# Novel and Known Gene-Smoking Interactions With cIMT Identified as Potential Drivers for Atherosclerosis Risk in West-African Populations of the AWI-Gen Study

**DOI:** 10.3389/fgene.2019.01354

**Published:** 2020-02-07

**Authors:** Palwende Romuald Boua, Jean-Tristan Brandenburg, Ananyo Choudhury, Scott Hazelhurst, Dhriti Sengupta, Godfred Agongo, Engelbert A. Nonterah, Abraham R. Oduro, Halidou Tinto, Christopher G. Mathew, Hermann Sorgho, Michèle Ramsay

**Affiliations:** ^1^ Clinical Research Unit of Nanoro, Institut de Recherche en Sciences de la Santé, Nanoro, Burkina Faso; ^2^ Faculty of Health Sciences, Sydney Brenner Institute for Molecular Bioscience (SBIMB), University of the Witwatersrand, Johannesburg, South Africa; ^3^ Division of Human Genetics, National Health Laboratory Service and School of Pathology, Faculty of Health Sciences, University of the Witwatersrand, Johannesburg, South Africa; ^4^ School of Electrical and Information Engineering, University of the Witwatersrand, Johannesburg, South Africa; ^5^ Navrongo Health Research Centre, Ghana Health Service, Navrongo, Ghana; ^6^ Julius Center for Health Sciences and Primary Care, University Medical Center Utrecht, Utrecht University, Utrecht, Netherlands; ^7^ Department of Medical and Molecular Genetics, Faculty of Life Sciences and Medicine, King's College London, London, United Kingdom

**Keywords:** GWIS, atherosclerosis, smoking, carotid intima-media thickness, gene-environment interactions

## Abstract

**Introduction:**

Atherosclerosis is a key contributor to the burden of cardiovascular diseases (CVDs) and many epidemiological studies have reported on the effect of smoking on carotid intima-media thickness (cIMT) and its subsequent effect on CVD risk. Gene-environment interaction studies have contributed towards understanding some of the missing heritability of genome-wide association studies. Gene-smoking interactions on cIMT have been studied in non-African populations (European, Latino-American, and African American) but no comparable African research has been reported. Our aim was to investigate smoking-SNP interactions on cIMT in two West African populations by genome-wide analysis.

**Materials and methods:**

Only male participants from Burkina Faso (Nanoro = 993) and Ghana (Navrongo = 783) were included, as smoking was extremely rare among women. Phenotype and genotype data underwent stringent QC and genotype imputation was performed using the Sanger African Imputation Panel. Smoking prevalence among men was 13.3% in Nanoro and 42.5% in Navrongo. We analyzed gene-smoking interactions with PLINK after adjusting for covariates: age and 6 PCs (Model 1); age, BMI, blood pressure, fasting glucose, cholesterol levels, MVPA, and 6 PCs (Model 2). All analyses were performed at site level and for the combined data set.

**Results:**

In Nanoro, we identified new gene-smoking interaction variants for cIMT within the previously described *RCBTB1* region (rs112017404, rs144170770, and rs4941649) (Model 1: p = 1.35E-07; Model 2: p = 3.08E-08). In the combined sample, two novel intergenic interacting variants were identified, rs1192824 in the regulatory region of *TBC1D8* (p = 5.90E-09) and rs77461169 (p = 4.48E-06) located in an upstream region of open chromatin. In silico functional analysis suggests the involvement of genes implicated in biological processes related to cell or biological adhesion and regulatory processes in gene-smoking interactions with cIMT (as evidenced by chromatin interactions and eQTLs).

**Discussion:**

This is the first gene-smoking interaction study for cIMT, as a risk factor for atherosclerosis, in sub-Saharan African populations. In addition to replicating previously known signals for *RCBTB1*, we identified two novel genomic regions (*TBC1D8*, near *BCHE*) involved in this gene-environment interaction.

## Introduction

During the last two decades, the burden of cardiovascular diseases (CVDs) has increased considerably, and low- and middle-income countries are now experiencing about 80% of the worldwide burden. Sub-Saharan Africa (SSA) is undergoing a health and demographic transition that has shifted the major causes of death from communicable and nutritional diseases to noncommunicable diseases (NCDs). The mean age of death attributable to CVDs in SSA in 2010 was 64.9 years (95% CI, 64.4–65.4) compared with 67.6–81.2 years for the rest of the world (Roth et al., 2015), making it one of the youngest affected populations globally. Populations of African descent have been under-represented in genomic studies, representing only about 3% of the participants worldwide used for genome-wide association studies up to 2016 (Popejoy and Fullerton, 2016). This is a gap that needs to be filled, considering that Africa is the continent with the highest genetic diversity owing to its deep evolutionary roots, and African genomes generally have lower linkage disequilibrium (LD). A previous study reported that African populations were more diverse and had significantly more genes and pathways involved in extreme allele frequency differences (EAFD) (Sulovari et al., 2017). The African genome is therefore highly relevant for the discovery of new genetic associations and a better understanding of human disease mechanisms (Tekola-Ayele and Rotimi, 2015; Choudhury et al., 2018; Martin et al., 2018).

Genetic understanding of complex traits has developed immensely over the past decade but remains hampered by the fact that genetic variants still explain only a fraction of the heritability of a trait, often referred to as the missing heritability. A contributor to this phenotypic variance is gene-environment interaction (GxE) (Duncan et al., 2014). GxE can be defined broadly as the interplay between the product of a genetic variant and an environmental factor as they affect a specific trait. GxE therefore refers to modification, by an environmental factor, of the effect of a genetic variant on a phenotypic trait. The phenotypic flexibility resulting from adjustments due to GxE could determine or modulate health or disease by modulating the adverse effects of a risk allele, or exacerbating the genotype-phenotype relationship to increase risk. Environmental stimuli, acting over hundreds of generations, have promoted adaptation that is reflected in allele frequency shifts observed in current populations for traits and disease risk.

Identifying GxE represents a cornerstone in “Precision Public Health” that will allow individuals to adjust exposure to a particular environmental factor involved in GxE interactions for the benefit of reducing disease risk in accordance with specific genotypes. However, if GxE testing represents an opportunity, challenges remain in interaction studies. The ability to detect interactions can be dependent on scale, SNP-based analyses can lack power, exposure measurements can be inconsistent and imperfect, and optimal software for efficient GxE analysis is lacking. Fortunately, recent advances in methodology development have boosted GxE interaction analysis and more studies are being published.

Smoking is an important risk factor for coronary heart disease (CHD) and CVD (Schroeder, 2013). Despite improved understanding, the pathophysiological mechanisms underpinning the association between smoking and CVD have yet to be elucidated fully. Nonetheless smoking is known to have an effect on endothelial cells, inflammatory states, platelet activation, procoagulant factors, and antifibrinolytic factors (Barua and Ambrose, 2013). Several studies have reported the effect of smoking on subclinical atherosclerosis (Liang et al., 2009; Yang et al., 2015; Hansen et al., 2016; Kianoush et al., 2017). Atherosclerosis is a complex, progressive disorder affecting large and medium-sized arteries. The disease has a silent progression, often with no clinical evidence until the occurrence of a vascular event.

Gene-smoking interactions for atherosclerosis have been reported. In the Bogalusa Heart Study, a variant located in the region of *EDNRA* was found to be associated with the status of the left cIMT (Li et al., 2015), and in the Northern Manhattan Study (NOMAS), *RCBTB1* was reported as a modifier of the smoking effect on cIMT, and *MXD1-JPH1* for carotid plaque burden in the presence of smoking (Wang et al., 2014; Della-Morte et al., 2014). Genes involved in inflammatory pathways mediated by the NF-kB axis (*TBC1D4* and *ADAMTS9*) have been identified as displaying gene-smoking interactions (Polfus et al., 2013). In 2017, a case-control study on CHD identified variants in *ADAMTS7* associated with a loss of cardio-protective effects resulting from gene-smoking interactions (Saleheen et al., 2017).

The Africa Wits-INDEPTH partnership for Genomic Studies (AWI-Gen), a Collaborative Centre of the Human Heredity and Health in Africa (H3Africa) Consortium, was developed to investigate the genomic and environmental risk factors for cardio-metabolic diseases in Africans. In this paper, we report on a genome-wide analysis of gene-environment interactions to explore the role of smoking on cIMT (a measure of atherosclerosis) in male participants from Nanoro (Burkina Faso) and Navrongo (Ghana) in West-Africa, as part of the AWI-Gen study.

## Methods

### Study Population

AWI-Gen is a cross-sectional study of adults (40 to 60 years of age) and in this study we used a subset of participants from the AWI-Gen study (Derra et al., 2012; Oduro et al., 2012; Ramsay et al., 2016; Ali et al., 2018), including male participants from the two study sites in West Africa. The participants for this study included 1,776 West African men from two rural settings, Nanoro (Burkina Faso) and Navrongo (Ghana). Only men were included in this study as smoking rates in women are very low in these communities. Participants completed a questionnaire with questions on demography, health history and behaviour. The reason for only performing the analysis on the two West African study sites, and not the other AWI-Gen study sites, is that they were comparable in terms of environmental exposures, genetic background, and prevalence of HIV infection (Derra et al., 2012; Oduro et al., 2012).

### cIMT Measurement

cIMT was measured using Dual B-mode ultrasound images of the carotid tree showing a typical double line for the arterial wall. cIMT is best visible in the measurement segment of the distal common carotid artery with lowest measurement variability. The measurement is most reliable over a one centimeter segment and was performed by semi-automatic reading methods, which minimise reading errors. The far wall of both the left and right common carotid artery was imaged using a linear-array 12L-RS transducer with a GE Healthcare B-mode LOGIQe ultrasound machine (GE, Healthcare, CT, USA). The participant was in a supine position for the measurements, head turned towards the left at a 45-degree angle to measure the right carotid. Operators used anatomical landmarks to identify the common carotid artery (CCA) on a longitudinal plane and the image was frozen. The operator then identified a continuous one-centimeter segment (10 mm) of the CCA far wall and placed a cursor between two points (10 mm apart) on this identified segment with the proximal starting point 1 cm from the bulb of the CCA. The inbuilt software then automatically detected the intima-lumen and the media-adventitia interfaces and calculated the minimum, maximum and mean common cIMT in mm and to two decimal places. To measure the left carotid, the participant's head was turned to the opposite side, and the process was repeated. The cIMT values were QCed according to the Mannheim Consensus defining the use of cIMT in population-based studies. We generated the mean cIMT as the average of the mean common right and left cIMT and used this variable for all analyses.

### Smoking Status and Other Variables

Smokers (current) and nonsmokers (never and former) were classified based on self-reporting. A dichotomous categorization of smoking status was chosen over a quantitative measure (e.g., pack-years) owing to the inherent high dimensionality of GWAS analysis (13.98 million SNPs with two-fold main effects and interaction variables per SNP). Additionally, previous studies reported the reversal of the effect of smoking after several years of cessation. Smoking intensity was assessed using pack/years calculated by multiplying the number of packs of cigarettes smoked per day by the number of years the person has smoked. The number of cigarettes or times a tobacco product were consumed was recorded at intervals of days (everyday, 5-6 days, 1-4 days, 1-3 days/month, less than once a month).

Other variables (height (m), weight (kg), BMI (kg/m^2^), blood pressure, fasting glucose, total cholesterol and physical activity) were recorded as previously described (Ali et al., 2018).

### Association and Follow-Up Analysis

#### Genotype Data and Imputation

The H3Africa genotyping array^1^, designed as an African-common-variant-enriched GWAS array (Illumina) with ~2.3 million SNPs, was used to genotype genomic DNA using the Illumina FastTrack Sequencing Service^2^. The following preimputation QC steps were applied to the entire AWI-Gen genotype data set. Individuals with a missing SNP calling rate greater than 0.05 were removed. SNPs with a genotype missingness greater than 0.05, MAF less than 0.01, and Hardy-Weinberg equilibrium (HWE) P-value less than 0.0001 were removed. Nonautosomal and mitochondrial SNPs, and ambiguous SNPs that did not match the GRCh37 references alleles or strands were removed. Imputation was performed on the cleaned data set (with 1,729,661 SNPs and 10,903 individuals from the AWI-Gen study) using the Sanger Imputation Server and the African Genome Resources as reference panel. We selected EAGLE2 (Loh et al., 2016) for prephasing and the default PBWT algorithm was used for imputation. After imputation, poorly imputed SNPs with info scores less than 0.6, MAF less 0.01, and HWE P-value less than 0.00001 were excluded. The final QC-ed imputed data had 13.98 M SNPs, and data from male participants from the Nanoro and Navrongo study sites were extracted for analysis.

#### Data Analysis

Descriptive statistics were used to summarise the population characteristics. Continuous variables were reported in median and interquartile ranges and categorical variables were reported in percentages. All the data were analyzed per site before reporting for the combined group. We examined group differences using the Mann-Whitney test for continuous variables and Pearson Chi-square test for categorical variables. Analyses were performed using mean cIMT and SNPs with a MAF of 0.05 or above.

#### Gene-Environment Interaction (GxE) Analysis

Linear regression of mean cIMT was performed with covariates using R^3^. Residuals were extracted from the linear regression analyses and used for the GWAS analysis. Model 1 used the following covariates: age and six principal components (PCs) computed on genetics data (to account for genetic structure). To check the consistency of our association, Model 2 included further adjustment [Model 1 + BMI + systolic blood pressure + diastolic blood pressure + fasting glucose + total cholesterol + physical activity (moderate to vigorous physical activity in minutes per week (MVPA)] to analyze SNPs with p-values < 1 E-06 in Model 1.

GxE testing was performed using the PLINK “-gxe” option (Purcell et al., 2007; Chang et al., 2015) on the “awigen” branch of the automated workflow^4^ (Baichoo et al., 2018). We screened the output for a genome-wide significance threshold (p-values < 5 E-08).

To assess genomic inflation, the observed distribution of −log10(*P*) values was compared to that expected in the absence of association (lambda) and illustrated in QQ plots. We used a cross-replication approach between the two sites, suggestive signals (p-values < 1E-04) in one site were checked in the other site and vice versa.

Power calculations were performed with Quanto^5^ (Version 1.2.4). The study was powered at 80% to identify SNPs with MAF ≥0.05 and interaction effect size (OR) of >4, given our sample size and smoking prevalence in Nanoro. The power would be higher for Navrongo and the combined data set because of the increased number of smokers.

#### Functional Analysis of Associated Variants

The FUMA online platform^6^ (Watanabe et al., 2017) was used to annotate, prioritize, visualize, and interpret GWAS results. From GWAS summary statistics as an input, it provided extensive functional annotation for all SNPs in genomic areas identified by lead SNPs. From the list of gene IDs (as identified by SNP2GENE option in FUMA) FUMA annotated genes in biological context (Watanabe et al., 2017). We selected all candidate SNPs in the associated genomic region having r^2^ ≥ 0.6 with one of the independently significant SNPs, with a suggestive P-value (P < 1E-05) and MAF ≥ 0.05 for annotation. Predicted functional consequences for these SNPs were obtained by matching the SNP's chromosome base-pair position, and reference and alternate alleles, to databases containing known functional annotations, including ANNOVAR (Wang et al., 2010), combined annotation-dependent depletion (CADD) scores (Kircher et al., 2014), and RegulomeDB (RDB) (Boyle et al., 2012) scores.

#### Functional Annotation of Mapped Genes

Genes implicated by mapping of significant GWAS SNPs were further investigated using the GENE2FUNC procedure in FUMA (Watanabe et al., 2017), which provides hypergeometric tests of enrichment of the list of mapped genes in 53 GTEx tissue-specific gene expression sets (The GTExArd Consortium et al., 2015; GTex Consortium et al., 2017), 7,246 MSigDB gene sets^7^, and chromatin states (Ernst and Kellis, 2013; Consortium Roadmap Epigenomics et al., 2015).

#### GWAS Catalog Lookup

The GWAS Catalog database was downloaded from the website^8^ (Accessed on 12 Jul 2018) and a subset the data set generated using the following key words relevant to our study: genome-wide interaction, gene-environment interactions, atherosclerosis, coronary artery diseases, carotid atherosclerosis, cIMT, coronary artery calcification, abdominal artery aneurism. Since our data set was in build 37, we performed a lift-over prior to comparison. In order to assess whether our study was replicating previous findings, we searched for the same marker or any markers within 100 kb of all suggestive index SNPs (p-value ≤ 1E-04) found in this study and further filtered the list using key words pertaining to coronary artery diseases and gene-environment interactions.

### Ethics and Consent

This study received the approval of the Human Research Ethics Committee (Medical), University of the Witwatersrand, South Africa (M121029), the approval of the Centre Muraz Institutional Ethics Committee, Burkina Faso (015-2014/CE- CM) and the approval of the National Ethics Committee For Health Research, Burkina Faso (2014-08-096), the Ghana Health Service Ethics Review Committee (ID No: GHS-ERC:05/05/2015), and the Navrongo Institutional Review Board (ID No: NHRCIRB178). All the participants signed an Informed Consent Form before any study procedure was performed.

## Results

### Characteristics of Participants

The participant characteristics are presented for Nanoro (n = 993), Navrongo (n = 783), and for the combined data (n = 1776) in **Table 1**. Only males were included. The prevalence of current smokers was 13.3%, 42.5% and 26.2%, respectively for Nanoro, Navrongo, and the combined data set. Smokers were younger than nonsmokers in Nanoro. Interestingly, although the prevalence of current smokers was lower, the smoking intensity was much higher (in packs/year) in Nanoro, 6.6 (3.7-11.6) vs 1.7 (0-4.2) in Navrongo. Moreover, although there were no differences in mean cIMT values between smokers and nonsmokers, significant differences were observed for the following risk factors, BMI, systolic, and diastolic blood pressure, for both sites and overall. Total cholesterol and low density lipid cholesterol (LDL-C) were lower in smokers compared to nonsmokers in Nanoro and the combined sample, whereas fasting glucose was lower for smokers in Navrongo and the combined sample. In spite of the geographic and genetic proximity of the two groups, the differences in the prevalence and smoking intensity, as well as that of other risk factors, suggest that the GxE interaction mechanism might not be the same in the two study centres.

**Table 1:** Descriptive characteristics for participants from Nanoro (Burkina Faso) and Navrongo (Ghana) and the combined sample.

	Nanoro			Navrongo			All		
	Nonsmokers (861)	Smokers (132)	p-values⌖	Nonsmokers (450)	Smokers (333)	p-values⌖	Nonsmokers (1311)	Smokers (465)	p-values⌖
**Age (year)**	50 (45–55)	48 (43–52)	**0.0024**	50 (45–56)	51 (46–55)	0.5053	50 (45–55)	50 (45–55)	0.6104
**BMI (kg/m^2^)**	21.31 (19.47–23.61)	19.50 (17.99–21.54)	**<0.001**	20.99 (19.42–22.95)	20.03(18.58–21.44)	**<0.001**	21.22 (19.46–23.37)	19.90 (18.38–21.45)	**<0.001**
**Systolic BP (mmHg)**	118 (109–132)	113 (101–124)	**<0.001**	123 (112–136)	119 (108–133)	**0.0033**	120 (110–133)	117 (107–131)	**0.0016**
**Diastolic BP (mmHg)**	76 (69–83)	72 (64–81)	**0.0012**	76 (68–85)	74 (66–83)	**0.0233**	76 (69–84)	74 (65–82)	**<0.001**
**Fasting Glucose (mmol/l)**	4.90 (4.46–5.35)	4.94 (4.52–5.42)	0.5672	4.49 (4.05–5.00)	4.29 (3.94–4.73)	**<0.001**	4.77 (4.30–5.24)	4.47 (4.06–4.96)	**<0.001**
**Total cholesterol (mmol/l)**	3.59 (2.96–4.26)	3.3 (2.88–3.84)	**0.0063**	3.10 (2.57–3.66)	3.18 (2.61–3.67)	0.4888	3.37 (2.78–4.10)	3.21 (2.67–3.70)	**<0.001**
**Triglycerides (mmol/l)**	0.70 (0.53–0.97)	0.70 (0.55–0.94)	0.7984	0.55 (0.42–0.73)	0.57 (0.43–0.73)	0.6104	0.64 (0.48–0.89)	0.60 (0.45–0.77)	**<0.001**
**LDL-C (mmol/l)**	1.98 (1.51–2.55)	1.82 (1.44–2.14)	**0.0031**	1.70 (1.22–2.19)	1.67 (1.28–2.06)	0.6141	1.88 (1.39–2.44)	1.72 (1.31–2.08)	**<0.001**
**HDL-C (mmol/l)**	1.12 (0.94–1.36)	1.13 (0.88–1.41)	0.8254	1.12 (0.89–1.34)	1.16 (0.94–1.45)	**0.0352**	1.12 (0.92–1.36)	1.16 (0.92–1.43)	0.1209
**MVPA (min/week)**	1,485 (80–3,055)	2,520 (420–3,155)	**0.0185**	2,408 (1,035–3,060)	2,475 (1,080–3,120)	0.7610	2,220 (250–3,060)	2,490 (720–3,120)	**<0.001**
**Cigarette (Pack/Year)^ϒ^**	—	6.6 (3.7–11.6)	—	—	1.7 (0–4.2)	—	—	2.8 (0–6)	—
**Mean cIMT (mm)**	0.673 (0.598–0.758)	0.656 (0.585–0.736)	0.0952	0.678 (0.610–0.768)	0.680 (0.607–0.753)	0.7332	0.675 (0.603–0.760)	0.675 (0.598–0.750)	0.4297

⌖Mann-Whitney rank-sum test, p-values at p < 0.05 are shown in bold.

^ϒ^Number of pack-years = (number of cigarettes smoked per day/20) × number of years smoked.

BMI, body mass index; BP, blood pressure; MVPA, moderate to vigorous physical activity.

### Gene-Smoking Interactions

**Figure 1:**
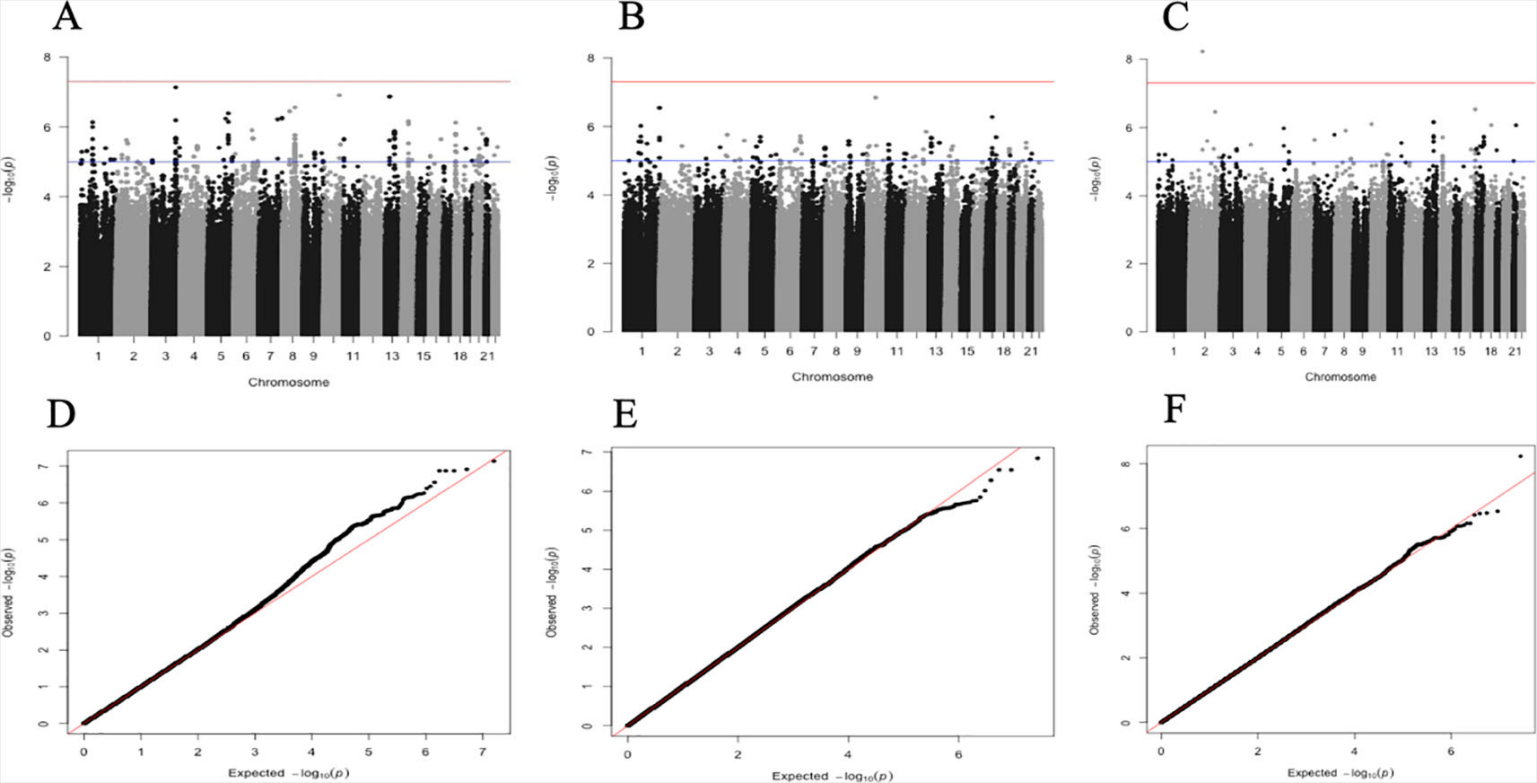
Manhattan plots showing the −log10-transformed two-tailed P-value of each SNP from the GWAS gene-smoking interaction for cIMT on the y-axis and base-pair positions along the chromosomes on the x-axis. The red line indicates Bonferroni-corrected genome-wide significance (p < 5E-08); the blue line indicates the threshold for suggestive association (p < 1E-04). Manhattan plot for Nanoro, 993 participants, 78289913 SNPs **(A)**. Manhattan plot for Navrongo, 783 participants, 7842446 SNPs **(B)**. Manhattan plot for combined set, 1776 participants, 7839440 SNPs **(C)**. QQ plot for Nanoro, GIF = 0.9944 **(D)**. QQ plot for Navrongo, GIF = 0.9934 **(E)**. QQ plot for combined set, GIF = 1.0079 **(F)**. GIF [genomic inflation factor (lambda)].

The association results of SNPs (MAF ≤ 0.05) are illustrated by Manhattan plots for each site and the combined data set in **Figures 1A–C**. Genomic inflation factors (GIFs) (lambda) were 0.994, 0.993, and 1.007, respectively for Nanoro (7,828,913 SNPs), Navrongo (7,842,446 SNPs), and the combined sample (7,839,440 SNPs) (**Figures 1D–F**).

**Figure 2:**
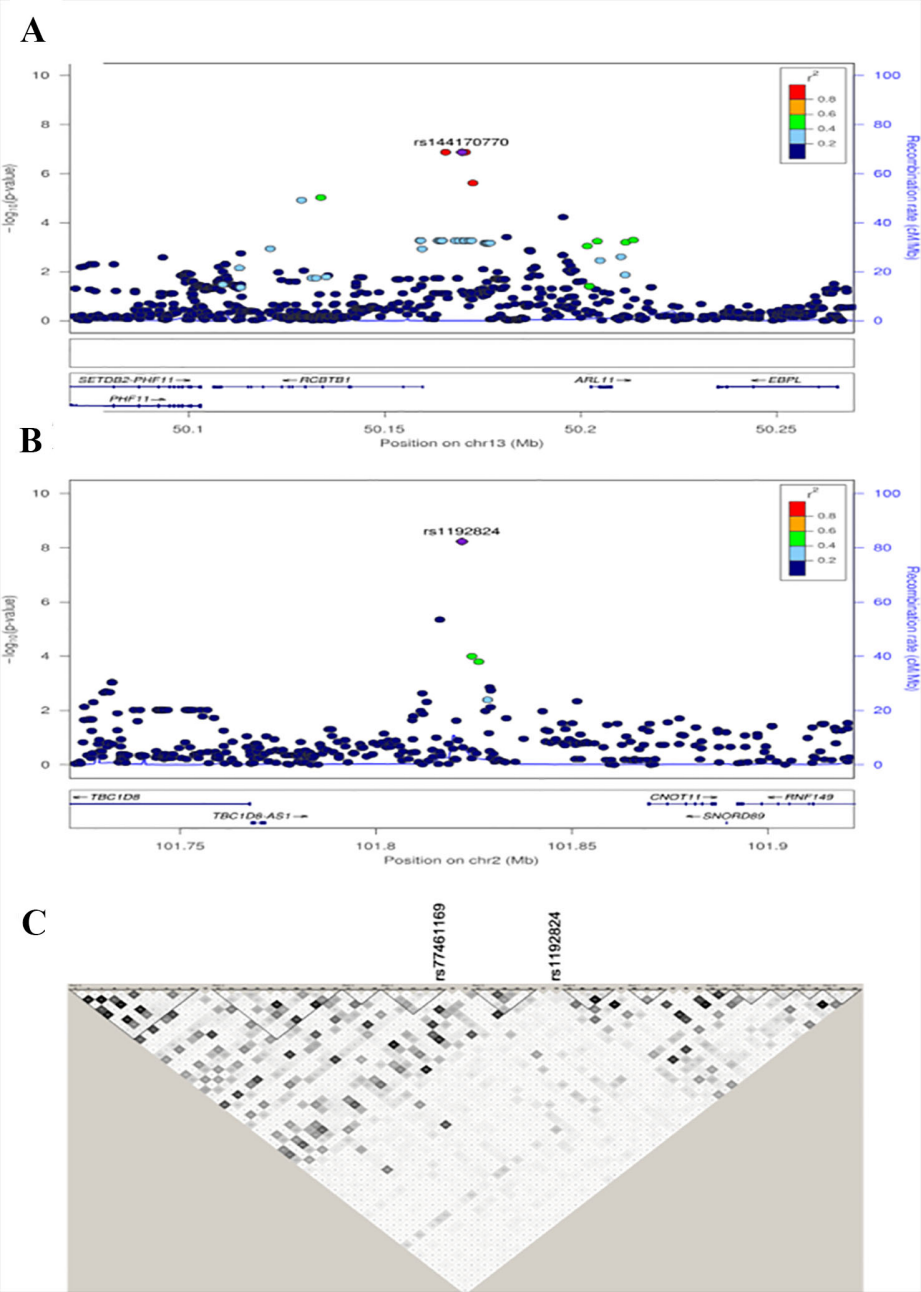
Regional association plots for the *RCBTB1* region in Nanoro **(A)**. Regional association plots of *TBC1D8* region in the combined data set **(B)**. Distinct genomic risk loci were defined as linkage disequilibrium (LD)-independent regions (r^2^) separated by 100 kb and containing one or more SNPs with a suggestive association (p-values < 1E-05). For each locus, the plots show the –log10 transformed value of each SNP on the y-axis and base pair positions along the chromosomes on the x-axis. Genes overlapping the locus are displayed below the plot. SNPs are colored by their LD value with the lead SNP in the region, and those LD values were generated from the study populations. Haplotype blocks show that rs1192824 and rs77461169 are not in LD. Haplotype blocks were built using Haploview with LD values calculated from the two study populations together **(C)**.

The strongest signal in the Nanoro sample was found for the GxE where the C allele of rs7649061 (allele frequency (AF) = 0.25, p-value = 7.37E-08) was associated with a decrease of mean-cIMT in the presence of smoking (**Table 2A**, **Supplementary Table 1**). This variant is located in an intergenic region between *BCHE* (butyrylcholinesterase) and *ZBBX* (zinc finger B-box domain containing). Four other regions had signals with p < 5E-07 in the analysis for Nanoro: rs7095209 (p-value = 1.23E-07), an intergenic SNP close to the *SORCS3* gene (sortilin related VPS10 domain containing receptor 3), the G allele of which (AF = 0.42) was associated with a decrease of mean-cIMT in the presence of smoking (**Supplementary Figures 1** and **2**); three chromosome 13 variants in high LD (rs112017404, rs144170770 and rs4941649), with the lowest p-value at 1.35E-07 (AF = 0.08), associated with an increase of mean-cIMT in smokers, and located between *RCBTB1* (Regulator of chromosome condensation (RCC1) and BTB (POZ) domain containing protein 1) and *ARL11* (ADP-ribosylation factor-like 11) (**Figure 2A**); rs13268575 (p = 2.77E-07) in the *CNBD1* (cyclic nucleotide binding domain containing 1) region which was associated with an increase of mean-cIMT in smokers for the A allele (AF = 0.23); and a missense variant, rs17844302 (p-value = 4.07E-07), in *PCDHA6* (protocadherin alpha 6), found to be associated with an increase of mean-cIMT in smokers.

**Table 2A:** Selected risk loci (p ≤ 1E-05) for SNP-smoking interactions on cIMT in Nanoro.

rsID	CHR	POS	A1^Ψ^	A2	BETA Nonsmokers	SE Nonsmokers	Allele Freq Nonsmokers	BETA Smokers	SE Smokers	Allele Freq Smokers	Z_GxE*	P_GXE	Funcrefgene	Generefgene
rs205482	1	10813675	G	T	−0,016	0,007	0,799	0,063	0,016	0,842	−4,558	5,16E-06	intronic	CASZ1
rs11161464	1	85298111	G	A	−0,015	0,008	0,86	0,073	0,016	0,877	−4,953	7,34E-07	intronic	LPAR3
rs1057305	1	175126223	A	T	−0,006	0,006	0,25	0,063	0,014	0,261	−4,608	4,08E-06	UTR3	KIAA0040
rs79936010	1	210977179	G	C	−0,011	0,008	0,114	0,081	0,019	0,11	−4,451	8,55E-06	intronic	KCNH1
rs148751085	2	38310007	T	G	0,021	0,006	0,216	−0,055	0,016	0,247	4,518	6,27E-06	intergenic	CYP1B1,CYP1B1-AS1
rs11126201	2	69084017	A	G	−0,019	0,005	0,508	0,042	0,012	0,492	−4,716	2,39E-06	intergenic	ARHGAP25,BMP10
rs6747064	2	78141022	T	C	−0,008	0,007	0,163	0,074	0,016	0,161	−4,673	2,98E-06	ncRNA_intronic	LOC101927967
rs4684453	3	4975879	T	A	0,015	0,006	0,277	−0,052	0,014	0,249	4,441	9,01E-06	ncRNA_intronic	BHLHE40-AS1
rs7649061	3	166457671	A	C	0,019	0,006	0,231	−0,058	0,013	0,255	5,381	7,37E-08	intergenic	BCHE,ZBBX
rs779272	3	191506748	T	A	−0,025	0,01	0,125	0,074	0,019	0,085	−4,615	3,94E-06	intergenic	LINCR-0002,FGF12
rs79243722	4	93634354	G	A	−0,005	0,01	0,068	0,109	0,023	0,071	−4,49	7,08E-06	intronic	GRID2
rs6819524	4	116593891	G	T	0,011	0,007	0,14	−0,081	0,018	0,156	4,642	3,44E-06	intergenic	NDST4,MIR1973
rs73764616	5	76294836	T	C	0,016	0,011	0,068	−0,108	0,025	0,069	4,613	3,96E-06	intergenic	CRHBP,AGGF1
rs79414964	5	107950880	T	C	−0,009	0,011	0,049	0,127	0,029	0,063	−4,446	8,74E-06	intergenic	FBXL17,LINC01023
rs2407218	5	121909381	G	A	−0,001	0,011	0,045	0,154	0,029	0,06	−4,998	5,78E-07	intergenic	MGC32805,LOC101927379
5:140208927	5	140208927	A	C	−**0**,008	0,01	0,098	0,108	0,021	0,091	−5,064	4,07E-07	exonic	PCDHA6
rs180973	5	165988671	A	G	0,03	0,01	0,932	−0,086	0,024	0,926	4,463	8,08E-06	intergenic	LOC102546299,CTB-7E3.1
5:171912171	5	171912171	T	C	0,003	0,006	0,549	−0,057	0,012	0,586	4,489	7,15E-06	intergenic	SH3PXD2B,NEURL1B
rs6934349	6	6899063	C	G	0,016	0,006	0,33	−0,047	0,013	0,305	4,529	5,89E-06	intergenic	LY86,RREB1
rs699402	6	122588540	C	T	−0,015	0,01	0,072	0,111	0,024	0,074	−4,847	1,24E-06	intergenic	GJA1,HSF2
rs59322395	6	128914510	G	A	0,012	0,008	0,117	−0,087	0,019	0,116	4,74	2,14E-06	intergenic	PTPRK,LAMA2
rs7450411	6	161010354	A	C	−0,011	0,009	0,125	0,075	0,017	0,097	−4,428	9,52E-06	intronic	LPA
rs4728159	7	128856192	C	T	−0,018	0,006	0,735	0,052	0,013	0,709	−4,989	6,07E-07	intergenic	SMO,AHCYL2
rs6978311	7	158665920	A	G	−0,012	0,009	0,064	0,122	0,025	0,093	−5,009	5,47E-07	intronic	WDR60
rs28890775	8	52207622	T	C	0,005	0,01	0,076	0,126	0,021	0,078	−5,091	3,56E-07	intergenic	SNTG1,PXDNL
rs13268575	8	88391237	C	A	−0,005	0,006	0,216	0,078	0,015	0,229	−5,139	2,77E-07	intronic	CNBD1
rs74815096	8	128577675	C	T	−0,006	0,006	0,242	0,063	0,014	0,192	−4,499	6,79E-06	intergenic	CASC8,CASC11
rs28687781	9	78496674	G	A	0,013	0,006	0,216	−0,061	0,015	0,238	4,552	5,33E-06	intergenic	MIR548H3,PCSK5
rs58733878	9	126968674	C	G	−0,005	0,008	0,114	0,093	0,02	0,144	−4,542	5,59E-06	intergenic	LHX2,NEK6
rs112473634	9	136780781	T	C	−0,007	0,01	0,098	0,093	0,02	0,084	−4,428	9,52E-06	intronic	VAV2
rs7095209	10	106781929	A	G	0,009	0,005	0,428	−0,064	0,013	0,425	5,289	1,23E-07	intronic	SORCS3
rs55729345	10	115390091	C	T	−0,007	0,005	0,367	0,057	0,013	0,407	−4,657	3,18E-06	intronic	NRAP
rs112017404	13	50165471	T	C	−0,016	0,01	0,061	0,12	0,024	0,085	−5,273	1,35E-07	intergenic	RCBTB1,ARL11
rs2819241	13	83709519	C	T	0,026	0,008	0,152	−0,062	0,016	0,123	4,837	1,33E-06	intergenic	NONE,SLITRK1
rs2357001	14	64352814	G	A	0,014	0,005	0,663	−0,052	0,012	0,625	4,96	7,01E-07	intronic	SYNE2
rs11851487	14	88284233	A	T	−0,021	0,006	0,349	0,042	0,013	0,334	−4,447	8,78E-06	intergenic	LOC283585,GALC
rs77506474	16	6068251	G	A	−0,009	0,008	0,114	0,086	0,019	0,121	−4,504	6,68E-06	upstream	RBFOX1
rs74032742	16	78725080	G	A	−0,02	0,009	0,076	0,096	0,023	0,085	−4,729	2,26E-06	intronic	WWOX
rs11663276	18	8653957	T	G	−0,01	0,008	0,136	0,083	0,017	0,144	−4,948	7,53E-07	intergenic	RAB12,GACAT2
rs60637140	18	13808717	G	A	−0,025	0,01	0,072	0,097	0,025	0,073	−4,551	5,36E-06	intergenic	RNMT,MC5R
rs67256262	19	1778023	A	G	−0,018	0,006	0,201	0,059	0,015	0,237	−4,601	4,20E-06	intergenic	ONECUT3,ATP8B3
rs36010337	19	44530696	C	A	−0,021	0,009	0,114	0,076	0,02	0,09	−4,43	9,41E-06	intronic	ZNF222
rs113096000	20	35308843	G	A	0,012	0,006	0,258	−0,063	0,014	0,27	4,87	1,12E-06	intronic	NDRG3
rs6025645	20	56157341	A	G	0,009	0,005	0,394	−0,054	0,012	0,44	4,803	1,56E-06	intergenic	PCK1,ZBP1
rs9982779	21	20459913	T	G	−0,008	0,006	0,284	0,06	0,013	0,293	−4,727	2,28E-06	intergenic	LOC101927797,LINC00320
rs9680265	21	22433521	T	C	−0,007	0,007	0,208	0,062	0,014	0,203	−4,418	9,94E-06	intronic	NCAM2
rs13058149	22	27831347	T	C	0,006	0,005	0,515	−0,053	0,012	0,473	4,51	6,49E-06	intergenic	LINC01422,MN1
rs73425956	22	48984237	A	G	−0,007	0,009	0,102	0,099	0,021	0,099	−4,623	3,79E-06	intronic	FAM19A5

*Z_GxE shows Beta interaction. For beta interaction, positive values indicate association of allele A1 and increased cIMT; negative values indicate association of allele A1 with decreased cIMT.

^Ψ^A1 is reflecting the alternative allele compared to the reference genome allele, all frequencies are reported for A1.

The associations for the analysis of the Navrongo data were less significant with none reaching p < 1E-07 (**Table 2B**, **Supplementary Table 1**). The strongest association signal was for rs4869800 (p-value = 1.92E-06, AF = 0.92), an intergenic variant between *RGS17* (regulator of G-protein signaling 17) and *OPRM1* (opioid receptor, mu 1). Other suggestive signals included genes from the olfactory receptor (OR) family. While there could indeed be true signals in the OR gene family, given the high false positive variant discovery rate in this gene family (Chen et al., 2017), it is difficult to assess the robustness of this association using imputed data sets, therefore we excluded these variants from the downstream analyses.

**Table 2B:** Selected risk loci (p ≤ 1E-05) for SNP-smoking interactions on cIMT in Navrongo.

rsID	CHR	POS	A1^Ψ^	A2	BETA Nonsmokers	SE Nonsmokers	Allele Freq Nonsmokers	BETA Smokers	SE Smokers	Allele Freq Smokers	Z_GxE*	P_GXE	Funcrefgene	Generefgene
rs12743305	1	102039804	T	G	−0,032	0,011	0,153	0,040	0,011	0,126	−4,691	2,72E-06	intergenic	LINC01307,OLFM3
rs452108	1	158716549	A	C	−0,054	0,015	0,937	0,045	0,015	0,940	−4,661	3,17E-06	intergenic	OR6K3,OR6K6
rs59483727	4	108724898	C	T	0,026	0,012	0,110	−0,052	0,013	0,097	4,442	8,92E-06	intergenic	PAPSS1,SGMS2
rs62352637	5	38912972	C	T	0,027	0,010	0,129	−0,042	0,012	0,130	4,440	9,02E-06	intronic	OSMR
rs6908852	6	130781638	C	T	0,014	0,007	0,538	−0,034	0,008	0,528	4,583	4,59E-06	intergenic	TMEM200A,SMLR1
rs4869800	6	154002533	G	A	−0,039	0,013	0,922	0,054	0,014	0,922	−4,762	1,92E-06	intergenic	RGS17,OPRM1
rs2849519	6	163132372	T	C	0,030	0,010	0,833	−0,036	0,011	0,838	4,518	6,23E-06	intronic	PARK2
rs79668927	7	154114710	C	T	−0,025	0,012	0,087	0,058	0,013	0,092	−4,605	4,15E-06	intronic	DPP6
rs11793060	9	133407956	C	T	−0,055	0,013	0,098	0,028	0,013	0,079	−4,517	6,28E-06	intergenic	ASS1,LOC100272217
rs10964835	9	21099991	A	G	0,024	0,007	0,419	−0,025	0,008	0,387	4,493	7,03E-06	intergenic	IFNB1,IFNW1
rs76252315	10	367258	C	T	0,035	0,014	0,086	−0,053	0,014	0,072	4,493	6,98E-06	intronic	DIP2C
rs11043124	11	11184095	T	C	0,013	0,008	0,267	−0,043	0,009	0,274	4,648	3,36E-06	intergenic	ZBED5-AS1,GALNT18
rs891290	11	119245010	A	T	−0,044	0,014	0,081	0,045	0,014	0,069	−4,432	9,33E-06	intronic	USP2
rs12797802	11	132031224	C	T	−0,019	0,008	0,338	0,030	0,008	0,307	−4,518	6,24E-06	intronic	NTM
rs11067175	12	115013760	A	T	−0,031	0,010	0,131	0,041	0,012	0,131	−4,581	4,64E-06	intergenic	TBX5-AS1,TBX3
rs7358623	12	59151544	G	A	−0,023	0,007	0,332	0,026	0,008	0,379	−4,482	7,42E-06	ncRNA_intronic	LOC100506869,LOC101927653
rs201818410	14	47817368	G	A	−0,018	0,008	0,273	0,037	0,009	0,254	−4,482	7,39E-06	intronic	MDGA2
rs11158732	14	68825191	C	A	−0,021	0,007	0,386	0,027	0,008	0,477	−4,624	3,77E-06	intronic	RAD51B
rs10873486	14	98256752	C	T	0,033	0,007	0,338	−0,017	0,009	0,342	4,419	9,90E-06	intergenic	LOC100129345,LINC01550
rs116720528	17	40505531	A	T	−0,035	0,013	0,083	0,055	0,014	0,072	−4,600	4,24E-06	intronic	STAT3
rs7245961	19	4843612	G	A	0,034	0,008	0,707	−0,019	0,009	0,714	4,492	7,04E-06	intronic	PLIN3

*For beta interaction, positive values indicate association of allele A1 and increased cIMT; negative values indicate association of allele A1 with decreased cIMT.

^Ψ^A1 is reflecting the alternative allele compared to the reference genome allele, all frequencies are reported for A1.

**Figure 3:**
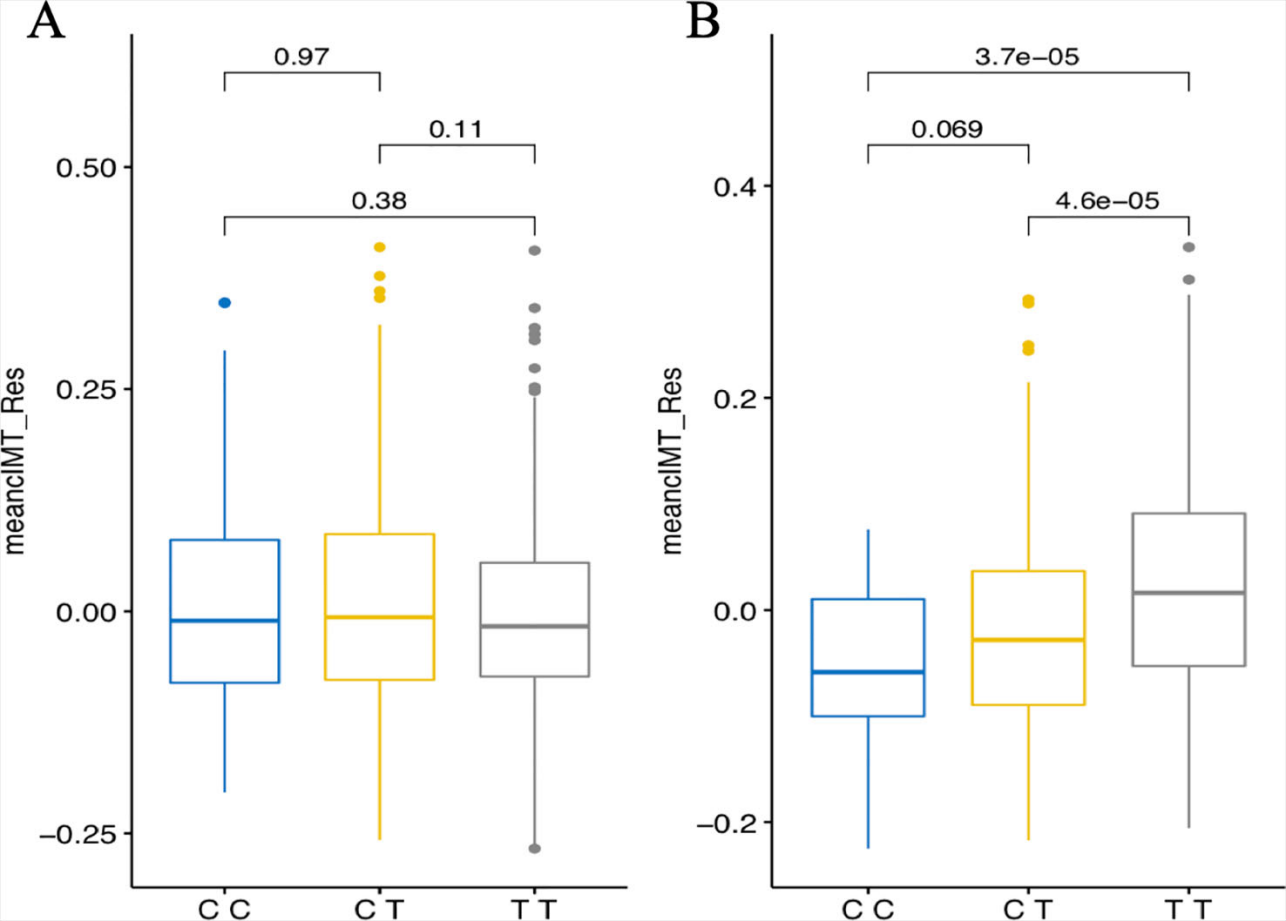
Genotypes plots of rs1192824 in the combined set showing distributions of mean cIMT residuals [adjusted for age and six principal components (PCs) for population structure] in nonsmokers **(A)** and smokers **(B)**. Mean_cIMT_Res are in mm. Displayed p-values report comparisons between genotype groups performed using the Kruskal-Wallis test.

In the combined sample, one SNP (rs1192824) reached the genome-wide significance level (**Table 2C**, **Supplementary Table 1**). The C allele of rs1192824 (AF = 0.69), located in the intergenic region between *TBC1D8* (TBC1 domain family, member 8) and *CNOT11* (CCR4-NOT Transcription Complex Subunit 11), showed a SNP-smoking interaction associated with a lower cIMT in smokers compared to the T carriers (p = 5.90E-09) (**Figure 2B**). Another variant in the promoter flanking region of *TBC1D8,* rs77461169, 5648 bp away from rs1192824, showed a suggestive interaction (p = 4.48E-06), and was located in an open chromatin region. The two variants were not in LD (**Figure 2C**). The distribution of mean cIMT for the three rs1192824 genotypes showed no difference in the nonsmokers, but there was a significant decrease of mean-cIMT for homozygote (C/C) and heterozygote (C/T) carriers among the smokers, when compared to the T/T genotype (**Figure 3**). This suggests a recessive mode of action for the risk allele (T). The second strongest signal was observed for rs12444312 (p-value = 1.27E-07) located in a noncoding RNA exon of *LOC440390*. This SNP is a regulatory region variant located in a CTCF binding site. A signal was found with rs11695675 (p-value = 3.48E-07) near *FTCDNL1* (formiminotransferase cyclodeaminase N-terminal like). Near *PCSK9*, rs1158815 was found suggestive of the GxE interaction for cIMT in the combined sample at p-value = 6.22E-06 (**Supplementary Figures 1** and **2**).

**Table 2C:** Selected risk loci (p ≤ 1E-05) for SNP-smoking interactions in combined sample.

rsID	CHR	POS	A1^Ψ^	A2	BETA Nonsmokers	SE Nonsmokers	Allele Freq Nonsmokers	BETA Smokers	SE Smokers	AlleleFreq Smokers	Z_GxE*	P_GXE	Funcrefgene	Generefgene
rs6685095	1	6502548	T	C	−0,005	0,006	0,131	0,047	0,010	0,135	−4,519	6,23E-06	intronic	ESPN
rs11588151	1	55487648	G	A	−0,024	0,008	0,087	0,040	0,012	0,083	−4,518	6,22E-06	intergenic	BSND,PCSK9
rs74115213	1	115054290	G	T	−0,012	0,007	0,081	0,051	0,012	0,095	−4,439	9,02E-06	upstream	TRIM33
rs1192824	2	101821934	T	C	0,012	0,005	0,674	−0,039	0,007	0,698	5,820	5,90E-09	intergenic	TBC1D8,CNOT11
rs73047627	2	189842214	A	G	0,018	0,009	0,062	−0,057	0,014	0,058	4,597	4,27E-06	intronic	COL3A1
rs11695675	2	200607333	A	G	−0,022	0,006	0,155	0,033	0,009	0,167	−5,095	3,48E-07	intergenic	LOC101927641,FTCDNL1
rs12465362	2	230587448	T	G	0,010	0,005	0,226	−0,033	0,008	0,238	4,475	7,63E-06	intergenic	DNER,TRIP12
rs7619102	3	12728045	A	G	0,017	0,006	0,150	−0,034	0,010	0,135	4,474	7,65E-06	intergenic	RAF1,TMEM40
rs869784	3	24348008	C	T	0,010	0,005	0,715	−0,029	0,007	0,698	4,507	6,57E-06	intronic	THRB
rs9834968	3	69552204	G	A	0,019	0,008	0,086	−0,044	0,012	0,082	4,481	7,45E-06	intergenic	FRMD4B,MITF
rs782444	3	127409941	C	T	0,009	0,004	0,473	−0,027	0,007	0,473	4,596	4,32E-06	UTR3	MGLL
rs76169119	4	40556609	G	C	0,022	0,009	0,063	−0,055	0,014	0,058	4,658	3,20E-06	intronic	RBM47
rs77655815	5	107952834	A	T	−0,016	0,008	0,056	0,068	0,015	0,068	−4,882	1,06E-06	intergenic	FBXL17,LINC01023
rs10454990	5	148838117	T	C	0,025	0,006	0,138	−0,029	0,010	0,129	4,558	5,17E-06	intergenic	MIR143HG,CSNK1A1
rs62435247	6	169725827	G	A	0,014	0,004	0,393	−0,024	0,007	0,387	4,726	2,30E-06	intergenic	THBS2,WDR27
rs62475193	7	154476372	A	G	−0,009	0,008	0,072	0,062	0,012	0,076	−4,792	1,65E-06	intronic	DPP6
rs16900766	8	126649041	G	A	−0,006	0,005	0,273	0,033	0,007	0,283	−4,458	8,30E-06	intergenic	TRIB1,LINC00861
rs74509340	10	480774	C	T	0,018	0,009	0,059	−0,065	0,014	0,066	4,936	7,97E-07	intronic	DIP2C
rs1854462	10	91991941	C	T	−0,016	0,007	0,102	0,042	0,011	0,103	−4,418	9,95E-06	intergenic	LINC01375,LOC101926942
rs112148169	10	133210477	A	T	−0,013	0,008	0,075	0,057	0,013	0,076	−4,518	6,26E-06	intergenic	TCERG1L,LINC01164
rs1939309	11	100421331	T	C	0,011	0,005	0,731	−0,032	0,008	0,740	4,681	2,84E-06	intergenic	CNTN5,ARHGAP42
rs7492237	13	84258581	T	C	0,015	0,005	0,263	−0,029	0,008	0,270	4,962	7,01E-07	intergenic	NONE,SLITRK1
rs1546993	14	40153203	A	G	0,013	0,004	0,512	−0,026	0,007	0,495	4,758	1,96E-06	intergenic	FBXO33,LOC644919
rs60889063	16	54371831	G	A	0,011	0,005	0,233	−0,032	0,008	0,235	4,593	4,39E-06	intergenic	IRX3,CRNDE
rs934166	16	84631300	G	A	−0,005	0,004	0,482	0,030	0,006	0,477	−4,581	4,63E-06	intronic	COTL1
rs11653290	17	6983529	C	A	−0,019	0,007	0,078	0,047	0,012	0,096	−4,599	4,22E-06	UTR5	CLEC10A
17:21509395	17	21509395	T	C	−0,015	0,008	0,090	0,049	0,012	0,086	−4,537	5,73E-06	intergenic	C17orf51,FAM27L
rs8067970	17	35145662	A	G	0,021	0,008	0,100	−0,042	0,011	0,092	4,638	3,52E-06	intergenic	MRM1,LHX1
rs10401037	17	52235347	G	C	0,007	0,005	0,261	−0,035	0,008	0,223	4,625	3,76E-06	intergenic	KIF2B,TOM1L1
rs4791039	17	64787600	A	G	−0,013	0,005	0,351	0,027	0,007	0,335	−4,764	1,90E-06	intronic	PRKCA
rs551160836	18	41413084	G	T	−0,014	0,009	0,078	0,058	0,012	0,069	−4,923	8,49E-07	intergenic	SYT4,LINC01478
rs8111212	19	6166665	T	C	−0,015	0,004	0,602	0,022	0,007	0,600	−4,581	4,65E-06	intronic	ACSBG2
rs389332	21	18270964	G	C	−0,018	0,008	0,939	0,056	0,014	0,934	−4,425	9,65E-06	intergenic	MIR99AHG,LINC01549
rs9974620	21	36097794	C	T	−0,006	0,005	0,713	0,037	0,007	0,687	−4,922	8,56E-07	ncRNA_intronic	LINC00160

*For beta interaction, positive values indicate association of allele A1 and increased cIMT; negative values indicate association of allele A1 with decreased cIMT.

^Ψ^A1 is reflecting the alternative allele compared to the reference genome allele, all frequencies are reported for A1.

When adjustment was applied for additional covariates, Model 1 + BMI + systolic blood pressure + diastolic blood pressure + fasting glucose + cholesterol + physical activity (MVPA), in Model 2, four variants in 2 loci reached genome-wide significance (rs7649061, p = 2.20E-08; rs112017404-rs144170770-rs4941649, p = 3.08E-08) in Nanoro. In the combined sample, rs1192824 (p = 2.70E-08) remained the single locus below the genome-wide significance threshold (**Table 3**). The direction of the allelic effect was the same in all cases where significant associations were identified in Nanoro and the combined sample.

**Table 3:** Comparison of gene-smoking association results for Nanoro and the combined sample (All), based on Model 1 and Model 2 for selected SNPs.

SNP	CHR	POS	A1	A2	All.P_GXE	MODEL 1				MODEL 2			
						Beta Interaction^*^	Nanoro.P_GXE	Beta Interaction^*^	All.P_GXE	Beta Interaction^*^	Nanoro.P_GXE	Beta Interaction^*^
rs11161464	1	85298111	G	A	3,29E-03	−2,94	7,34E-07	−4,95	4,61E-03	−2,83	2,14E-07	−5,19
**rs1192824**	**2**	**101821934**	**T**	**C**	**5,90E-09**	**5,82**	**5,01E-04**	**3,48**	**2,70E-08**	**5,56**	**3,10E-03**	**2,96**
rs11695675	2	200607333	A	G	3,48E-07	−5,10	5,48E-03	−2,78	3,27E-07	−5,11	9,66E-03	−2,59
**rs7649061**	**3**	**166457671**	**A**	**C**	**6,85E-04**	**3,40**	**7,37E-08**	**5,38**	**8,97E-04**	**3,32**	**2,20E-08**	**5,59**
rs7615817	3	166528151	G	A	1,70E-04	3,76	6,45E-07	4,98	2,01E-04	3,72	1,27E-06	4,84
rs2407218	5	121909381	G	A	4,05E-02	−2,05	5,78E-07	−5,00	3,20E-02	−2,14	3,58E-07	−5,09
5:140208927	5	140208927	A	C	7,98E-03	−2,65	4,07E-07	−5,06	1,08E-02	−2,55	4,18E-07	−5,06
5:140223827	5	140223827	A	G	1,15E-02	−2,53	7,16E-07	−4,96	1,71E-02	−2,39	1,23E-06	−4,85
rs4728159	7	128856192	C	T	1,84E-02	−2,36	6,07E-07	−4,99	2,66E-02	−2,22	4,96E-06	−4,57
rs115650684	7	158660582	T	C	7,74E-02	−1,77	5,77E-07	−5,00	1,49E-01	−1,44	4,04E-06	−4,61
rs6978311	7	158665920	A	G	7,49E-02	−1,78	5,47E-07	−5,01	1,46E-01	−1,45	3,93E-06	−4,61
rs28890775	8	52207622	T	C	9,83E-03	−2,58	3,56E-07	−5,09	8,92E-03	−2,62	1,27E-06	−4,84
rs13268575	8	88391237	C	A	2,96E-02	−2,18	2,77E-07	−5,14	4,14E-02	−2,04	1,54E-06	−4,81
rs74509340	10	480774	C	T	7,97E-07	4,94	1,04E-01	−1,63	2,05E-06	4,75	1,41E-01	1,47
rs7095209	10	106781929	A	G	5,46E-02	1,92	1,23E-07	−5,29	8,11E-02	1,74	1,90E-07	5,21
**rs112017404**	**13**	**50165471**	**T**	**C**	**5,95E-03**	−**2,75**	**1,35E-07**	−**5,27**	**8,83E-03**	−**2,62**	**3,08E-08**	−**5,54**
**rs144170770**	**13**	**50169651**	**G**	**A**	**5,95E-03**	−**2,75**	**1,35E-07**	−**5,27**	**8,83E-03**	−**2,62**	**3,08E-08**	−**5,54**
**rs4941649**	**13**	**50170562**	**C**	**A**	**6,64E-03**	−**2,71**	**1,35E-07**	−**5,27**	**1,06E-02**	−**2,56**	**3,08E-08**	−**5,54**
rs9546479	13	84256394	C	T	7,01E-07	4,96	1,45E-03	3,19	1,25E-06	4,85	1,10E-03	3,26
rs7492237	13	84258581	T	C	7,01E-07	4,96	1,45E-03	3,19	1,25E-06	4,85	1,10E-03	3,26
rs12435958	14	64352150	C	G	5,93E-03	2,75	7,01E-07	4,96	2,46E-03	3,03	1,86E-06	4,77
rs2357001	14	64352814	G	A	6,35E-03	2,73	7,01E-07	4,96	2,78E-03	2,99	1,86E-06	4,77
rs12895879	14	64353738	C	T	6,43E-03	2,72	7,19E-07	4,96	2,82E-03	2,99	1,90E-06	4,76
rs8004989	14	64354638	T	C	6,08E-03	2,74	8,43E-07	4,92	2,65E-03	3,00	2,16E-06	4,74
rs12444312	16	87097284	A	G	2,95E-07	−5,13	1,15E-03	−3,25	1,46E-06	−4,82	2,52E-03	−3,02
rs11663276	18	8653957	T	G	5,48E-02	−1,92	7,53E-07	−4,95	8,04E-02	−1,75	1,46E-06	−4,82
rs551160836	18	41413084	G	T	8,49E-07	−4,92	3,96E-02	−2,06	7,24E-07	−4,96	3,78E-02	−2,08
rs9974620	21	36097794	C	T	8,56E-07	−4,92	8,42E-04	−3,34	6,48E-07	−4,98	6,67E-04	−3,40

*For beta interaction, positive values indicate smoking interaction with allele A1 results in increased cIMT; negative values indicate smoking interaction with allele A1 results in decreased cIMT.

In bold are the variants reaching genome-wide significance threshold (p < 5E-08) in Model 2.

### Comparison of Association Signals Between Sites

We found eight SNPs from Nanoro with some evidence for interaction (p-values < 1E-04) which showed nominal replication (p-value ≤ 0.05) in Navrongo, with the strongest being rs77655815 (Nanoro p-value = 4.01E-05), replicated at p-value of 6.83E-05, and rs79419964 (Nanoro, p = 8.74E-06; Navrongo, p = 8.23E-04), and with p = 1.06E-06 (2.66E-06 for Model 2) in the combined analysis. From Navrongo, 19 SNPs (p < 1E-04) were nominally replicated in Nanoro, of which rs12444312 (Nanoro p = 1.15E-03), was found associated at a p-value of 2.95E-07 in the combined sample (**Supplementary Table 1**). When significant, the direction of the allelic effect was the same in all cases.

### GWAS Catalog Lookup for Gene-Environment Interaction

We replicated previously described gene-environment association loci for smoking interactions with body composition (BMI and waist circumference) (Justice et al., 2017), smoking or alcohol interaction with blood pressure (Taylor et al., 2016; Feitosa et al., 2018; Sung et al., 2018), coronary artery calcified plaque in type 2 diabetes (Divers et al., 2017) and peripheral arterial disease interaction with air pollution (Ward-Caviness et al., 2017) (**Supplementary Table 5**). No SNPs from these studies showed evidence of transference of the lead SNPs to African populations in our study, but none was specifically for cIMT as the main outcome. Interestingly, in Nanoro we identified 15 SNPs located within 100 kb of previously reported gene-environment interaction loci. The loci included a gene-alcohol interaction on blood pressure (Feitosa et al., 2018), gene-smoking interaction on waist circumference (Justice et al., 2017), gene-smoking interaction on lung cancer (Park et al., 2015) and gene-smoking interaction on blood pressure (Sung et al., 2018). In Navrongo, 13 SNPs replicated previous loci for gene-smoking interaction on BMI (Justice et al., 2017), lung cancer (McKay et al., 2017) and blood pressure (Taylor et al., 2016). Seventeen SNPs in the combined sample replicated previously reported interaction loci for gene-diabetes interaction for atherosclerotic plaque (Divers et al., 2017), gene-alcohol interaction for blood pressure (Feitosa et al., 2018), gene-smoking interaction for BMI (Justice et al., 2017) and gene-smoking interaction for blood pressure (Taylor et al., 2016; Sung et al., 2018).

### Functional Analysis

Functional annotation of SNPs with suggestive associations showed that these were mostly intronic or intergenic (**Supplementary Tables 2A–C**). 30 SNPs displayed a CADD score above 12.37 (17 in Nanoro; 3 in Navrongo; 10 in Combined), suspected to be deleterious. In the Nanoro sample, two SNPs (rs6701037, rs6677097), in high LD with rs10573305 (*KIAA0040* region), had a RDB score of 1f suggesting they were likely affecting binding sites and gene expression. Equally, for the combined sample two variants (rs10409209 and rs4807840), in LD with rs8111212, displayed a RDB score of 1f.

### Functional Annotation of Mapped Genes

**Figure 4:**
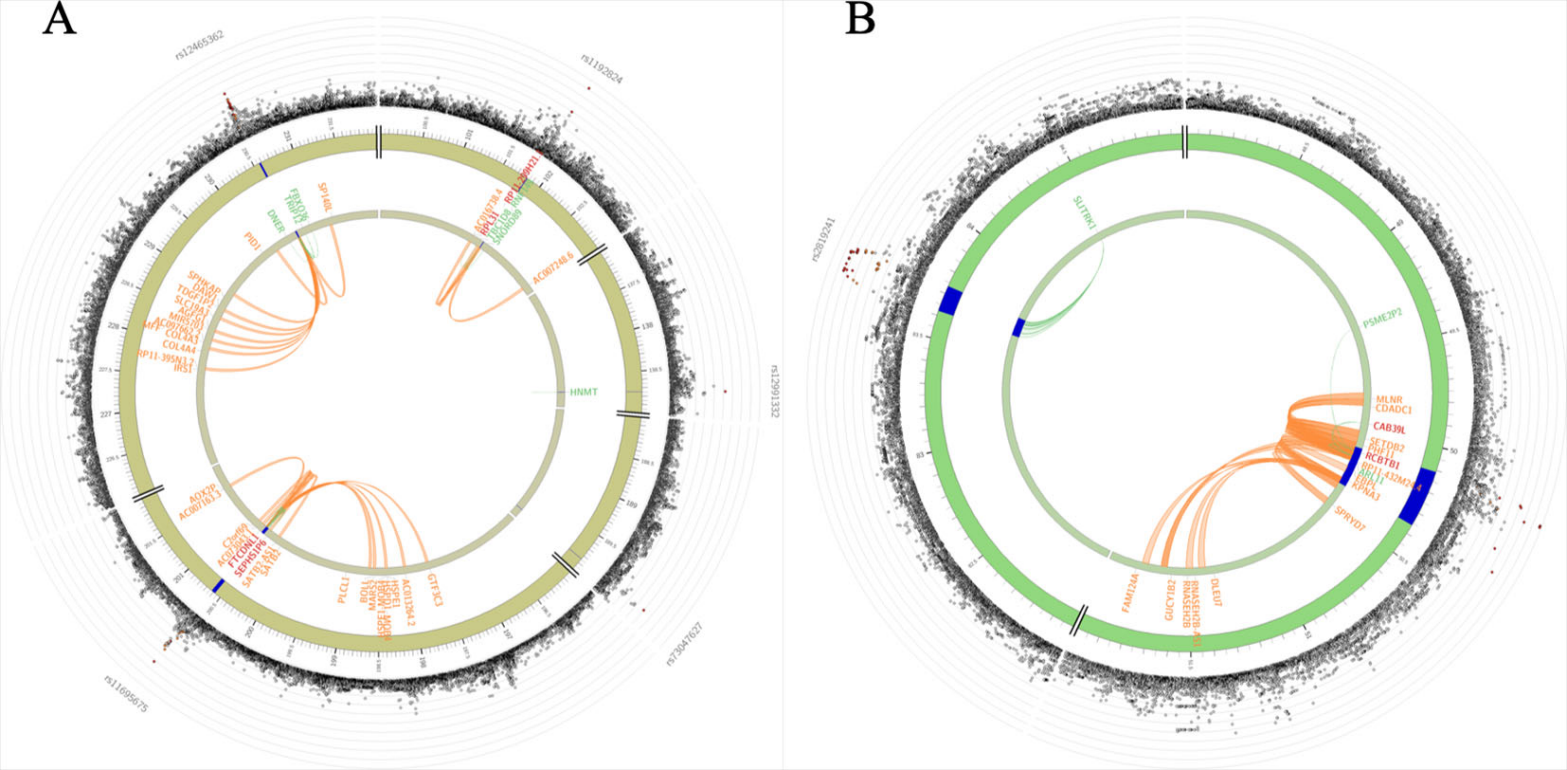
Circos plots showing genes on chromosomes 2 for combined sample **(A)** and on chromosomes 13 for Nanoro sample **(B)**. Blue regions are risk loci (P < 1E-05). Green lines are connecting an eQTL SNP to its associated gene. Orange lines are connecting two interacting regions for chromatin interactions. Genes implicated by eQTLs are shown in green, by chromatin interactions are shown in orange, and by both eQTLs and chromatin interactions are shown in red. The outer layer shows a Manhattan plot containing the –log^10^-transformed two-tailed p-value of each SNP from the gene-environment interaction analysis, with SNPs colored according to LD patterns with the lead SNP. Higher-resolution Circos plots for all chromosomes are provided in **Supplementary Figure 3**.

Genes implicated by mapping of significant SNPs were further investigated using the GENE2FUNC procedure in FUMA. Positional mapping, eQTL mapping (matched cis-eQTL SNPs) and chromatin interaction mapping (on the basis of 3D DNA–DNA interactions) are reported (**Supplementary Tables 3A–C**, **Supplementary Figure 3**). We found that rs1192824 and rs77461169 in *TBC1D8* were implicated as eQTLs influencing the expression of *TBC1D8*, *SNORD89,* and *RNF149* and also displaying chromatin interactions (**Figure 4A**). The *RCBTB1* locus SNPs were eQTLs for *RCBTB1*, *ARL11*, *CAB39L,* and *PSME2P2*; their chromatin interactions included surrounding genes such as *RCBTB1*, *KPNA3*, *CAB39L*, *SETDB2*, *MLNR,* and *CDADC1* (**Figure 4B**). A lookup into gene expression data sets, revealed expression of genes of interest in specific relevant tissues such as arteries (**Supplementary Figure 4**). We also analyzed the functional significance of the associated variants using FUN-LDA (Backenroth et al., 2018) and the results were largely similar to FUMA tissue-specific annotation.

### Gene Set Analysis

**Figure 5:**
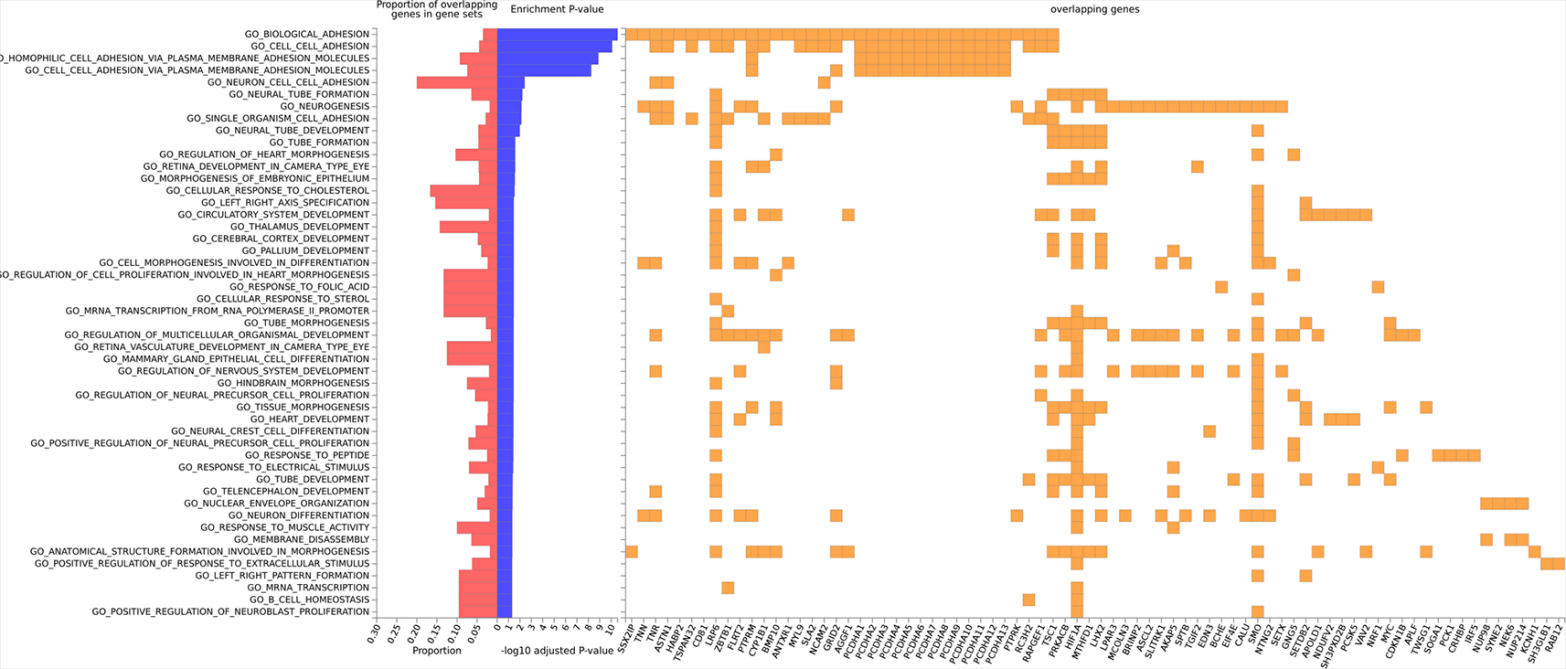
Gene ontology enrichment for biological processes for gene-smoking interaction on cIMT in Nanoro, with overlapping genes in gene sets.

Gene set analysis was only reported when at least five genes were implicated. In Nanoro, we found significant Gene Ontology Biological Processes for biological adhesion (AdjP = 2.69E-11), cell-cell adhesion (AdjP = 8.03E-11), homophilic cell adhesion *via* plasma membrane adhesion molecules (AdjP = 1.29E-09) and cell-cell adhesion *via* plasma membrane adhesion molecules (AdjP = 5.52E-09) (**Figure 5**; **Supplementary Tables 4A–C**).

## Discussion

Atherosclerosis is a low-grade chronic inflammatory condition characterized by aberrant lipid metabolism and a maladaptive inflammatory response. Biologically, the disease involves the formation of plaques in arterial walls and thickening that narrows the arterial passage, restricting blood flow and increasing the risk of occlusion resulting in a myocardial infarction and other events. Although environmental factors such as diet and/or smoking play an important role in the development of atherosclerosis, genetic factors represent important determinants of atherosclerotic CVD risk. Key gene-environment interactions may increase the risk for adverse outcomes by contributing to an increase of cIMT, and this was the aim of this study.

The gene-smoking interaction signals we identified for mean cIMT were with loci where the associated allele frequencies ranged from low to common, with most of them displaying effect sizes of over 4. This suggests that the effects were unlikely to be attributed to additive independent effects of genetic-association and smoking, but rather to the interaction. The high effects provided us with sufficient power to discover the gene-smoking interactions, given our sample size. Moreover, many previously reported loci for GxE with cardiovascular-related traits were replicated, and our study identified new GxE variants for cIMT. The loci identified are biologically relevant in terms of the pathophysiology of atherosclerosis and involve genes implicated in macrophage activation and recruitment in the endothelial layer, cholesterol metabolism at the cellular level, inflammation processes and signalling, and cell membrane activity.

Our study is the first to report an association of *TBC1D8* in the GxE interaction with cIMT and consequent risk for atherosclerosis. *TBC1D8* (also called Vascular Rab-GAP/TBC-containing protein) is a gene involved in blood circulation, intracellular protein transport and positive regulation of cell proliferation. The gene is regulated by vascular genes like *VEGF*, *ACKR1*, *VEGFA*, *SIRT1,* and *TNF*. Previous GWASs found variants in *TBC1D8* associated with bone mass (Kiel et al., 2007), cognitive decline (Li et al., 2015) and osteoporosis (Hsu et al., 2010) and found that *TBC1D8* expression was subject to change under environmental stress. A study on the effect of smoking on gene expression found that *TBC1D8* was differentially expressed in lymphocytes of smokers and nonsmokers (Charlesworth et al., 2010), as well as in macrophages from atherosclerotic plaques (Puig et al., 2011), depending on the inflammatory status of patients. Later Verdugo and colleagues (Verdugo et al., 2013) reported that *TBC1D8* expression in monocytes was subject to a gene interaction with smoking among atherosclerotic patients, and that *TBC1D8* was involved in one of the shortest gene paths between smoking and atherosclerotic plaques (smoking and plaques were separated by a relatively low number of genes). Their analysis of causality models provided evidence of gene expression partially mediating the relationship between smoking and atherosclerosis. Our study is therefore confirming the importance of *TBC1D8* gene-environment interaction in atherosclerosis pathophysiology.

*RCBTB1*, previously identified as a modifier for smoking on cIMT in multi-ethnic northern American populations, has been replicated in our study. The signal identified in our study is independent from the one previously described, although located in the same gene, the signal reported in the NOMAS study was led by the Hispanic population and located about 42 kb from our signal. But, the allele frequencies where highest in non-Hispanic blacks compared to Hispanic (rs3751383, MAF: 0.44 vs 0.25). Our data showed no LD (r^2^ < 0.02) between the lead SNPs from the two studies (rs3751383, rs112017404). The *RCBTB1* gene encodes a protein with an N-terminal RCC1 domain and a C-terminal BTB (broad complex, tramtrack, and bric-a-brac) domain. In rat, overexpression of this gene in vascular smooth muscle cells induced cellular hypertrophy. These results suggest that gene-smoking interaction for atherosclerosis might be acting through an intensification of monocyte activation and recruitment under the endothelial layers before their differentiation into macrophages, a process known to trigger foam cell formation and subsequent plaques (Jia et al., 2017). The results from the functional analyses suggest that the gene-smoking interaction for cIMT is likely acting through a regulatory process, explaining the involvement of multiple loci displaying chromatin interactions and acting as eQTLs. We were able, in our study, to reproduce a GxE interaction for markers in *RCBTB1*, albeit with an independent signal in the gene, demonstrating that the association is more generalizable. Our study is the first independent validation of the involvement of *RCBTB1* in gene-smoking interaction for cIMT.

There were differences in the association results between the two study sites with low replication. These differences may be partly explained by differences in the prevalence of smoking, sample sizes and smoking intensity. Effectively, the median smoking intensity in Nanoro was three times higher than in Navrongo (6.6 pack/year vs 1.7 pack/year) (**Table 1**), whereas the number of smokers in Nanoro (n = 132) was less than half of those in Navrongo (n = 333). A previous study using a systems biology approach revealed that cigarette smoke induced a concentration-dependent (direct and indirect) biological mechanism that promotes monocyte–endothelial cell adhesion (Poussin et al., 2015). Hence, the influence of smoking intensity on the detection of gene-smoking interaction was previously reported in a study of gene-smoking interaction for blood pressure in the Framingham Heart Study (Basson et al., 2015). They found different associated loci in the light smoker and the heavy smoker groups (>10 cigarettes per day). In the study by Wang et al. on gene-smoking interaction, the strongest association was among heavy smokers (≥20 pack/year) (Wang et al., 2014). This might explain why the *RCBTB1* region was only replicated in Nanoro, where the smoking intensity was higher than in Navrongo.

We report a substantial number of suggestive GxE signals that may be African-specific as they have not yet been observed in non-African studies with larger cohorts. Since African genetic diversity is generally higher, it is possible that there are more novel gene variants that are related to pathways involved in complex diseases like atherosclerosis (Sulovari et al., 2017). Our study is restricted to men and is limited by the sample size and relatively low prevalence of smokers in Nanoro. There was, however, sufficient power to detect the effect sizes we observed, and our sample size exceeded several previously published studies. In the design of the study, an inclusion criterion was that participants should not be closely related (Ali et al., 2018); however the genetic data revealed several individuals with first and second degree relatedness (204 in nonsmokers, 62 in smokers; 15%). To mitigate the effect of relatedness, we ran the analysis using GEMMA, a program that adjusts using the kinship matrix (Zhou et al., 2012). The comparison of the results from GEMMA (gxe option) and from PLINK (gxe option) showed that the outputs were highly correlated, indicating that relatedness had little effect on the outcomes.

Probable contributors to the heterogeneity of signals between the two geographical groups include differences in the patterns of smoking exposure and the simplistic measure of smoking status that we used in this study (current smokers vs nonsmokers), over the use of a continuous measure (pack/year).

## Conclusion

Our study provides the first report of gene-smoking interactions for cIMT in sub-Saharan African populations. We identified novel genome-wide significant variants in *TBC1D8* for interactions with smoking for cIMT. The replication of eight previous signals identified in non-African populations, demonstrates that these signals are transferable to West Africa. The discovery of the novel signals, on the other hand, indicates the possibility of African-specific associations. The strategies of functional annotation and gene mapping using biological data resources provided useful information on the likely consequences of relevant genetic variants and identified plausible gene targets and biological mechanisms for functional follow-up. Gene set analyses contributed novel insight into underlying pathways, confirming the importance of gene-environment interactions in atherosclerosis and pointing toward the involvement of specific cell types. Gene-environment GWASs will benefit from colocalization analyses for interpreting the biological and clinical relevance of the GWAS results. When variants associated with GxE are present at high frequency in target populations, this provides an opportunity for precision public health. Future studies based on populations from other African regions may provide validation of transferability to SSA more generally, identify further novel signals and to generate more insights into the relationship between these associations disease pathophysiology.

## Data Availability Statement

The raw data supporting the conclusions of this manuscript will be made available by the authors, without undue reservation, to any qualified researcher.

## Ethics Statement

This study received the approval of the Human Research Ethics Committee (Medical), University of the Witwatersrand/South Africa (M121029), the approval of the Centre Muraz Institutional Ethics Committee/Burkina Faso (015-2014/CE- CM) and the approval of the National Ethics Committee For Health Research/Burkina Faso (2014-08-096), the Ghana Health Service Ethics Review Committee (ID No: GHS-ERC:05/05/2015) and the Navrongo Institutional Review Board (ID No: NHRCIRB178). All the participants signed an Informed Consent Form before any study procedure was performed.

## Author Contributions

PB, HS, HT, AC, CM, and MR designed the study. PB and J-TB performed the analysis. DS performed the imputation. PB wrote the manuscript. PB, J-TB, AC, CM, HS, DS, SH, GA, EN, AO, HT and MR critically reviewed and approved the manuscript.

## Funding

This study was funded by the National Institutes of Health (NIH) through the H3Africa AWI-Gen project (NIH grant number U54HG006938) and the Wits Non-Communicable Disease Research Leadership Programme (NIH Fogarty International Centre grant number D43TW008330). AWI-Gen is supported by the National Human Genome Research Institute (NHGRI), Eunice Kennedy Shriver National Institute of Child Health & Human Development (NICHD), Office of the Director (OD) at the National Institutes of Health. PB is funded by the National Research Fondation/The World Academy of Sciences “African Renaissance Doctoral Fellowship” (Grant no. 100004).

## Conflict of Interest

The authors declare that the research was conducted in the absence of any commercial or financial relationships that could be construed as a potential conflict of interest.

## Footnotes

1
https://www.h3abionet.org/h3africa-chip

2
https://www.illumina.com/services/sequencing-services.html

3
https://www.R-project.org/

4
http://github.com/h3abionet/h3agwas/

5
http://biostats.usc.edu/Quanto.html

6
http://fuma.ctglab.nl/

7
http://software.broadinstitute.org/gsea/msigdb

8
https://www.ebi.ac.uk/gwas/
