# Supplementary material for: Novel and Known Gene-Smoking Interactions With cIMT Identified as Potential Drivers for Atherosclerosis Risk in West-African Populations of the AWI-Gen Study
Source: Front Genet. 2020 Feb 7;10:1354. doi: 10.3389/fgene.2019.01354 (PMC7025492; doi:10.3389/fgene.2019.01354)
Supplement: Supplementary file 2 [file Image_1.pdf]

Supplementary Figure 1a:

Regional association plots of selected loci (p-values) in Nanoro. Distinct genomic risk loci were defined as LD-independent regions (r 2 separated by 100 kb and containing one or more SNPs with a suggestive association (p-values<1E-05). For each locus , the plots shows the –log10 transformed –value of each SNP on the y-axis and base pair positions along the chromosomes on the x-axis. Genes overlapping the locus are displayed below the plot. SNPs are colored by their LD value with the lead SNP in the region, and those LD values has been generated from the study populations.

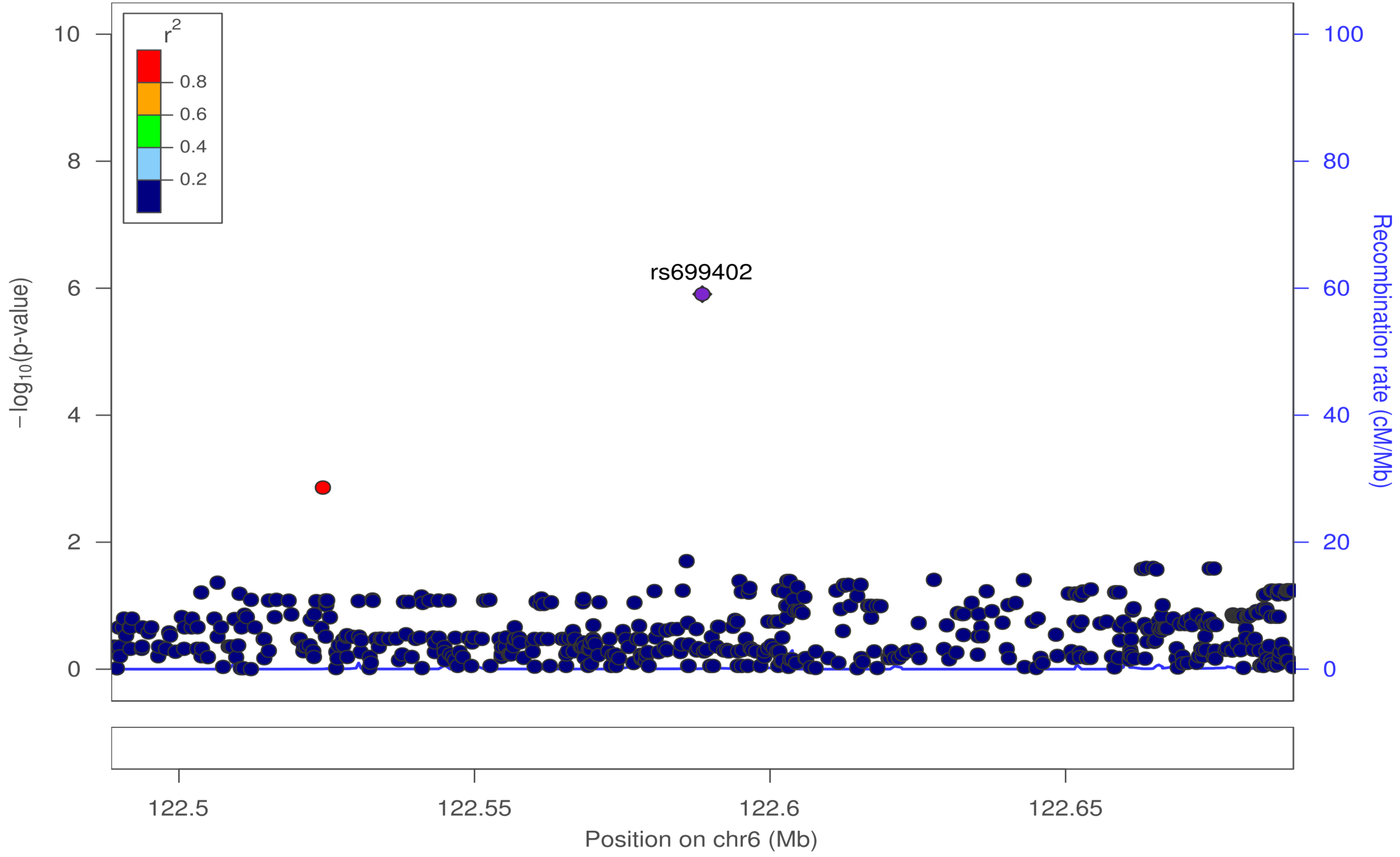
r2
0.8
0.6
0.4
0.2
rs699402
10
8
6
4
2
0
−log10(p-value)
100
80
60
40
20
0
Recombination rate (cM/Mb)
122.5
122.55
122.6
122.65
Position on chr6 (Mb)

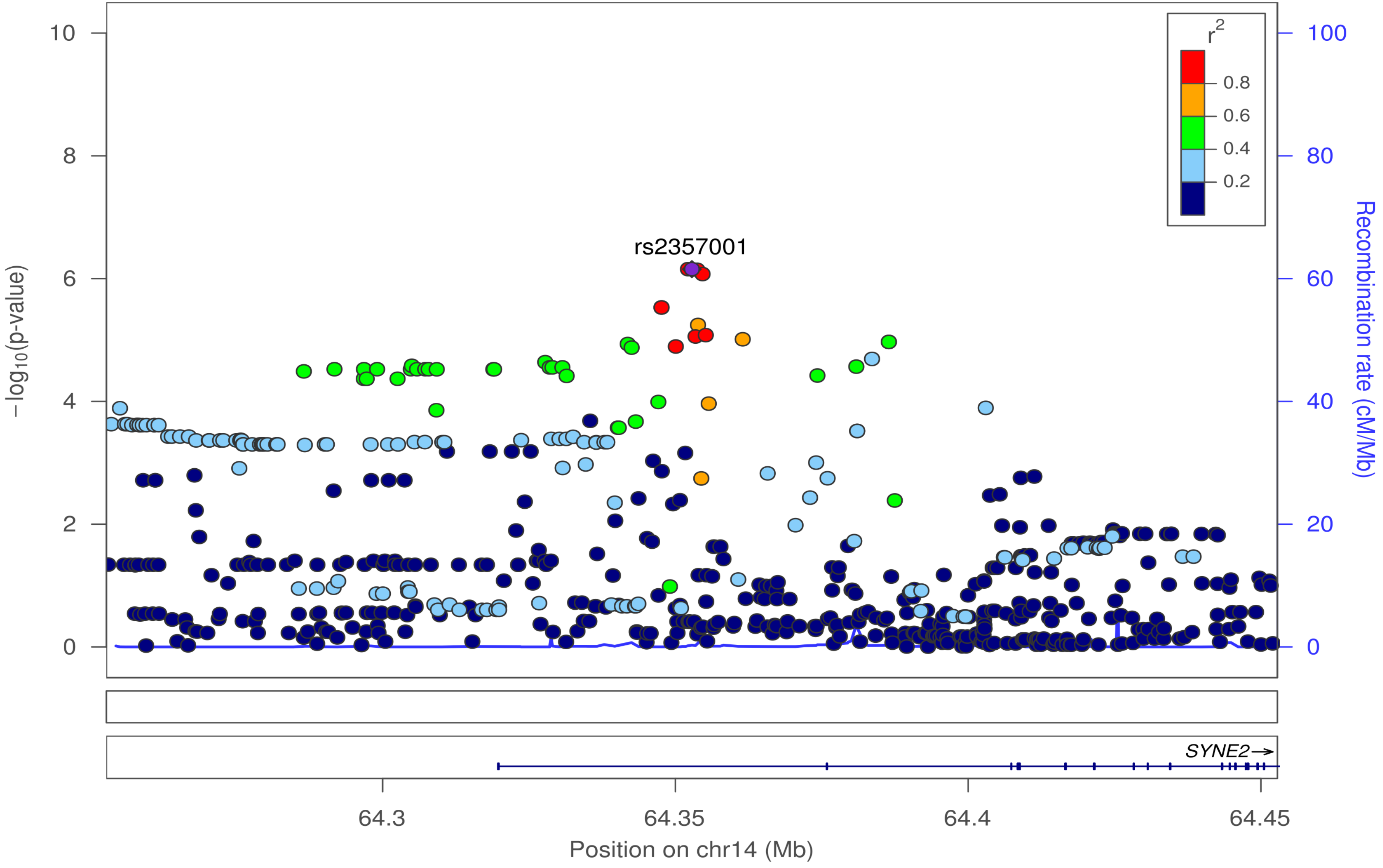
rs2357001
r²
0.8
0.6
0.4
0.2
−log10(p-value)
Recombination rate (cM/Mb)
0
2
4
6
8
10
0
20
40
60
80
100
SYNE2→
64.3
64.35
64.4
64.45
Position on chr14 (Mb)

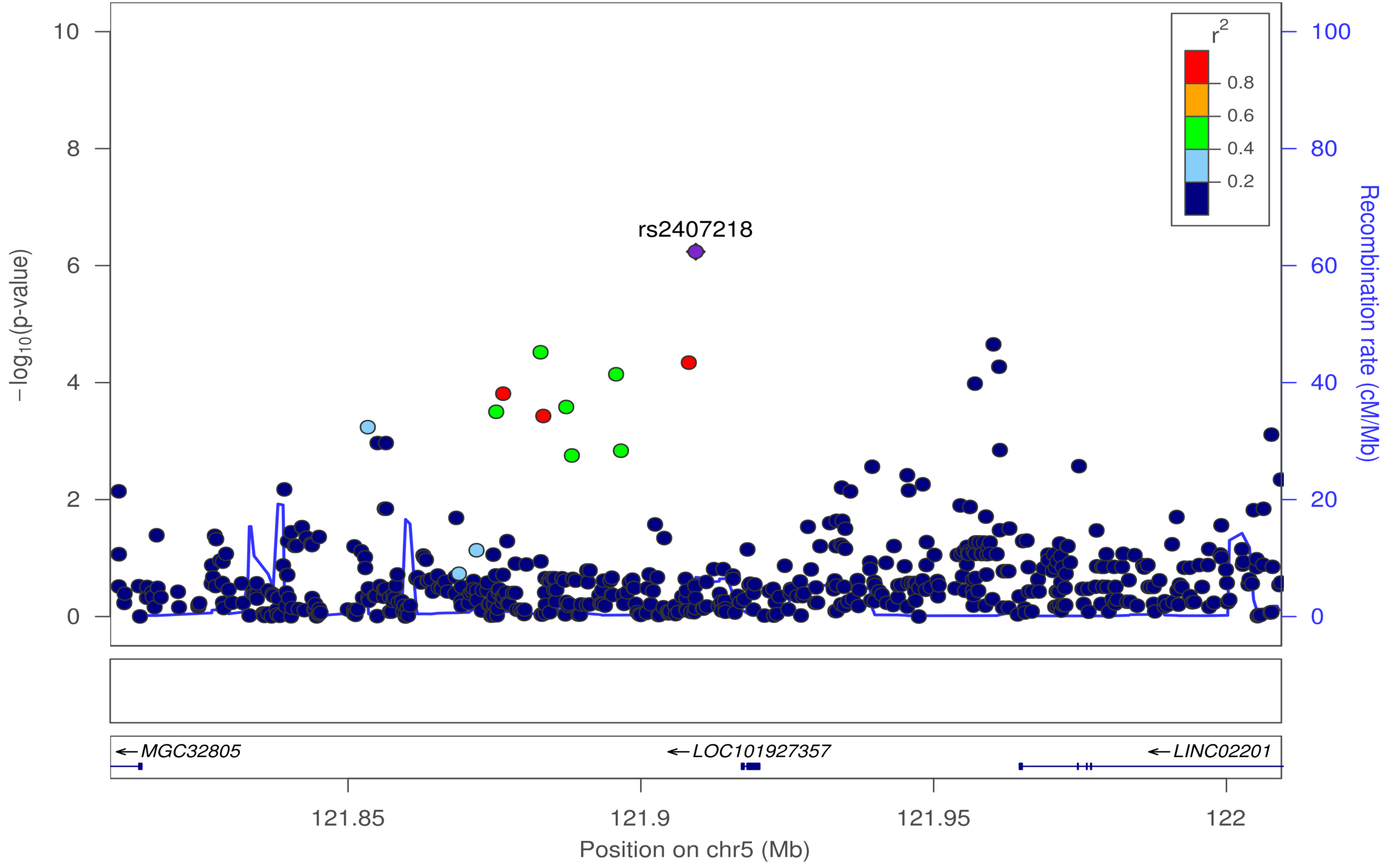

r2
0.8
0.6
0.4
0.2
rs2407218
−log10(p-value)
Recombination rate (cM/Mb)
0
2
4
6
8
10
0
20
40
60
80
100
MGC32805
LOC101927357
LINC02201
121.85
121.9
121.95
122
Position on chr5 (Mb)

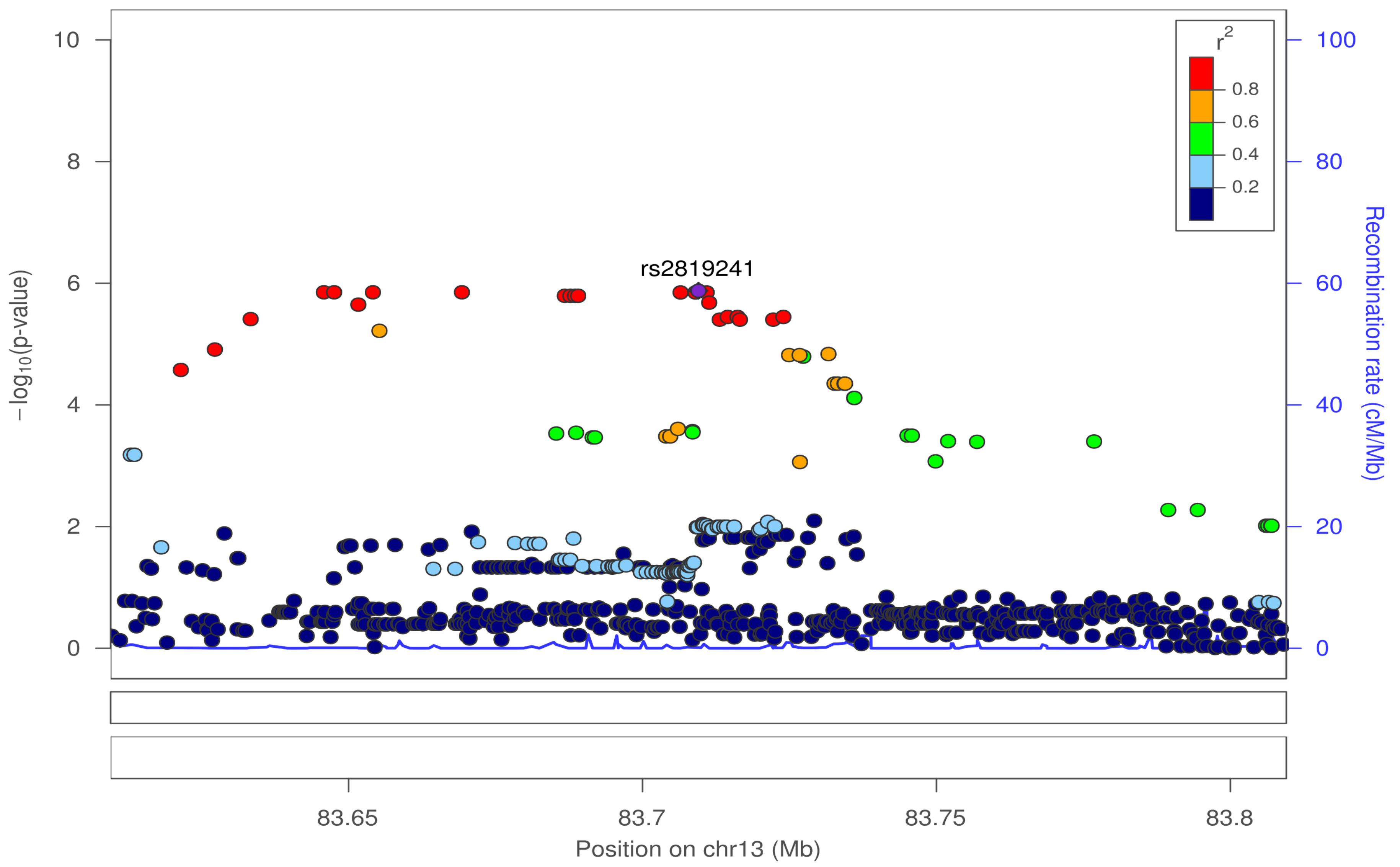
rs2819241
r2
0.8
0.6
0.4
0.2
−log10(p-value)
Recombination rate (cM/Mb)
Position on chr13 (Mb)
83.65
83.7
83.75
83.8
0
2
4
6
8
10
20
40
60
80
100

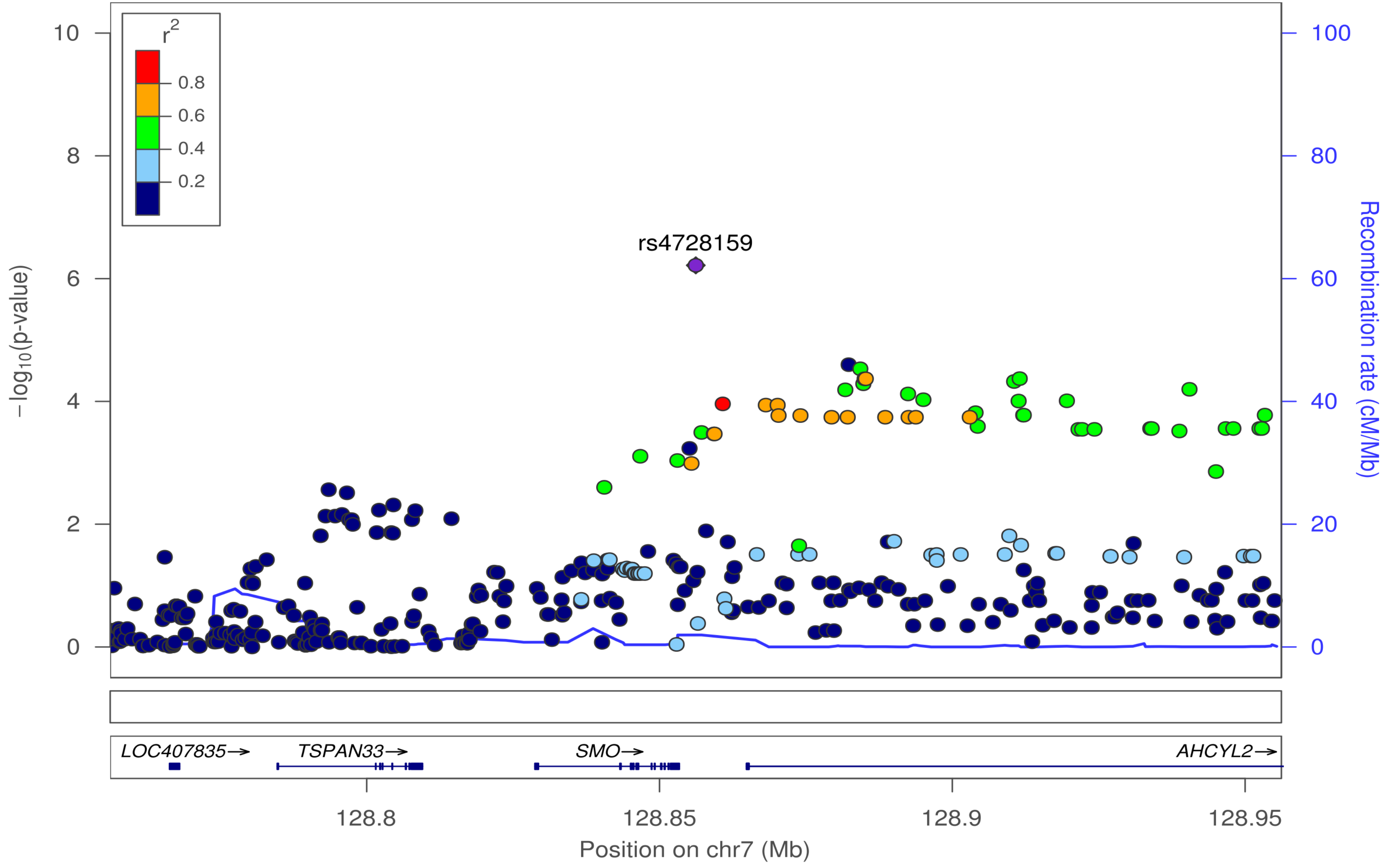

r2
0.8
0.6
0.4
0.2
rs4728159
−log10(p-value)
Recombination rate (cM/Mb)
10
8
6
4
2
0
100
80
60
40
20
0
LOC407835→
TSPAN33→
SMO→
AHCYL2→
128.8
128.85
128.9
128.95
Position on chr7 (Mb)

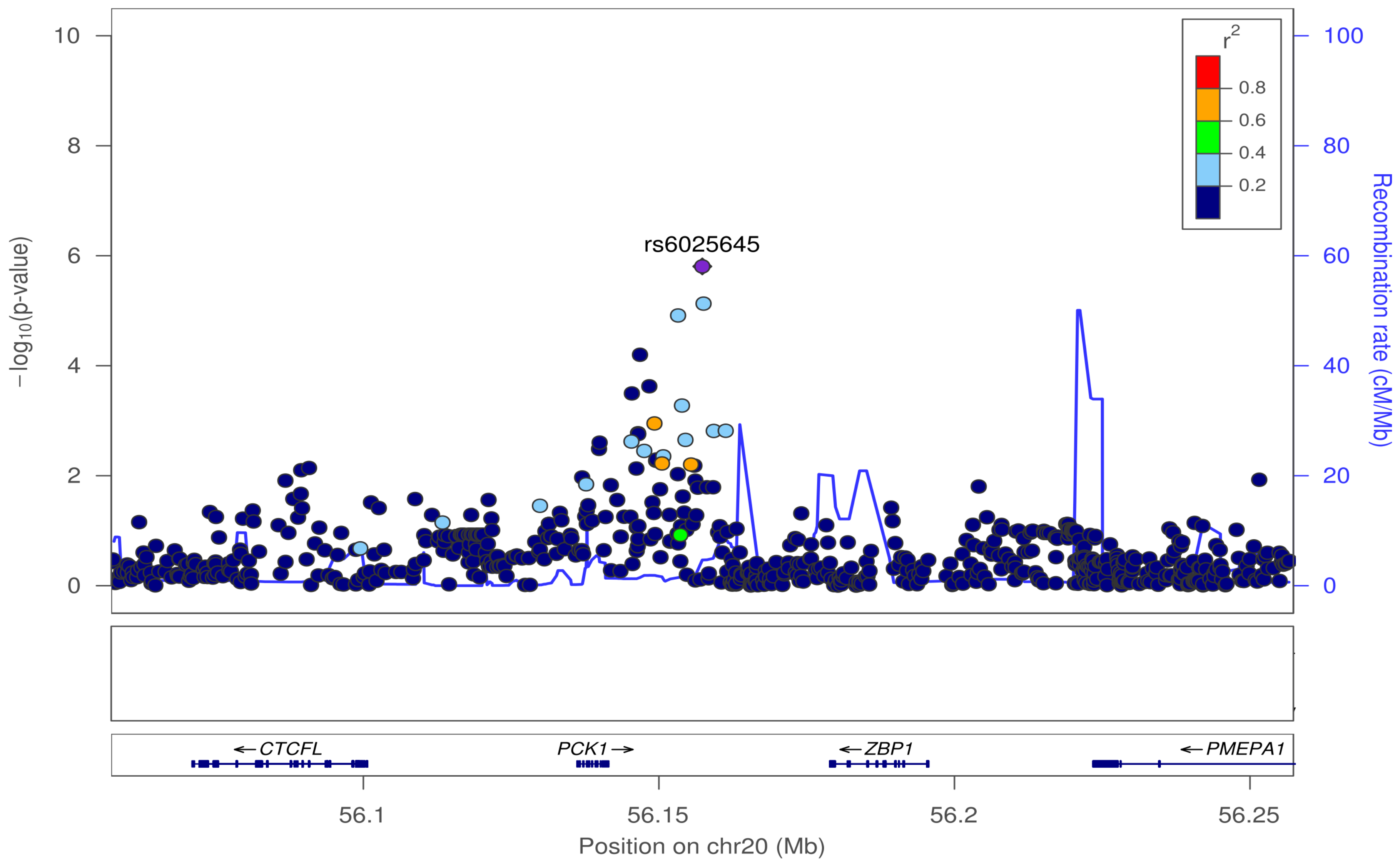

r2
0.8
0.6
0.4
0.2
rs6025645
−log10(p-value)
Recombination rate (cM/Mb)
CTCFL
PCK1
ZBP1
PMEPA1
56.1
56.15
56.2
56.25
Position on chr20 (Mb)

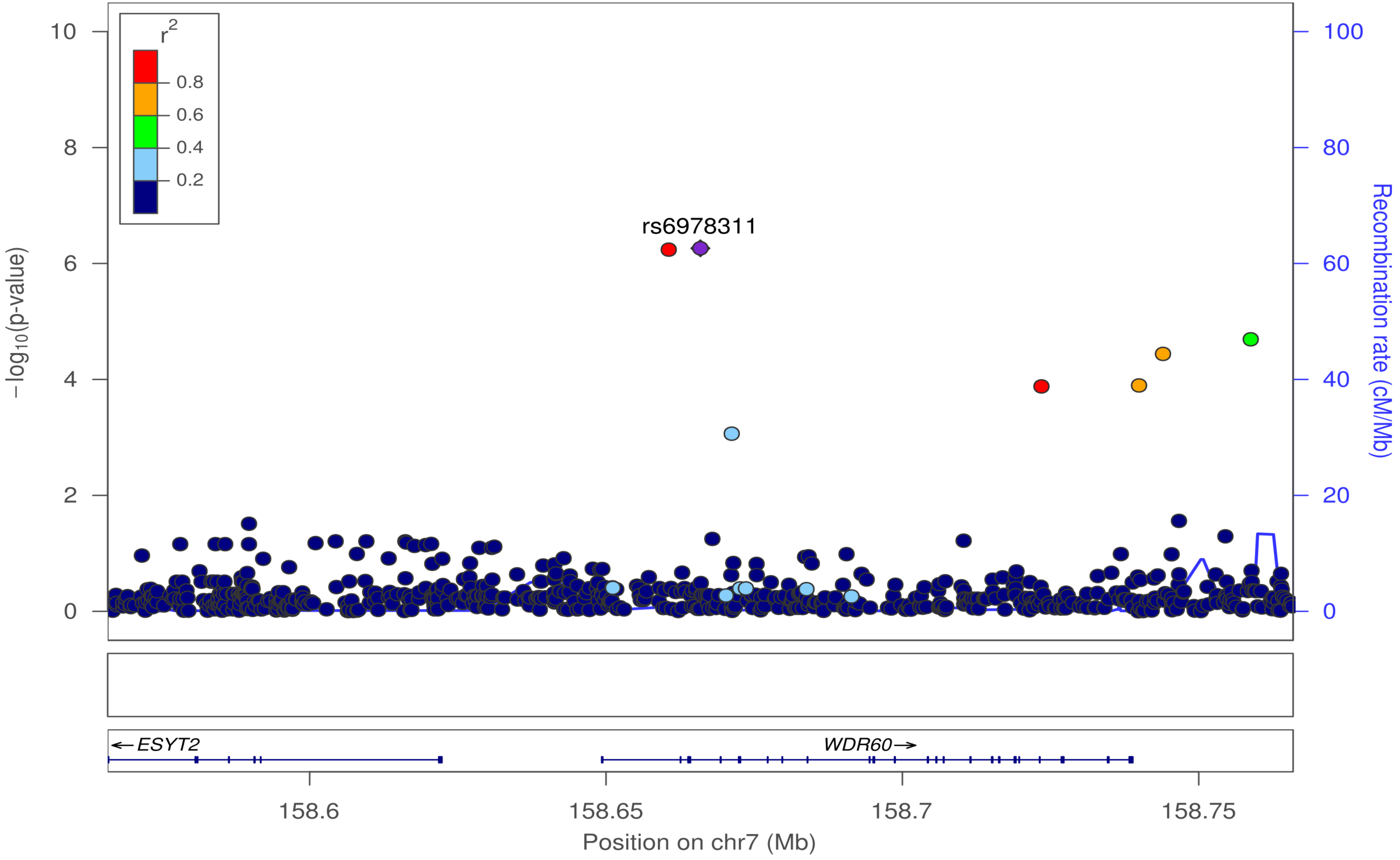

r2
0.8
0.6
0.4
0.2
rs6978311
−log10(p-value)
Recombination rate (cM/Mb)
ESYT2
WDR60
158.6
158.65
158.7
158.75
Position on chr7 (Mb)

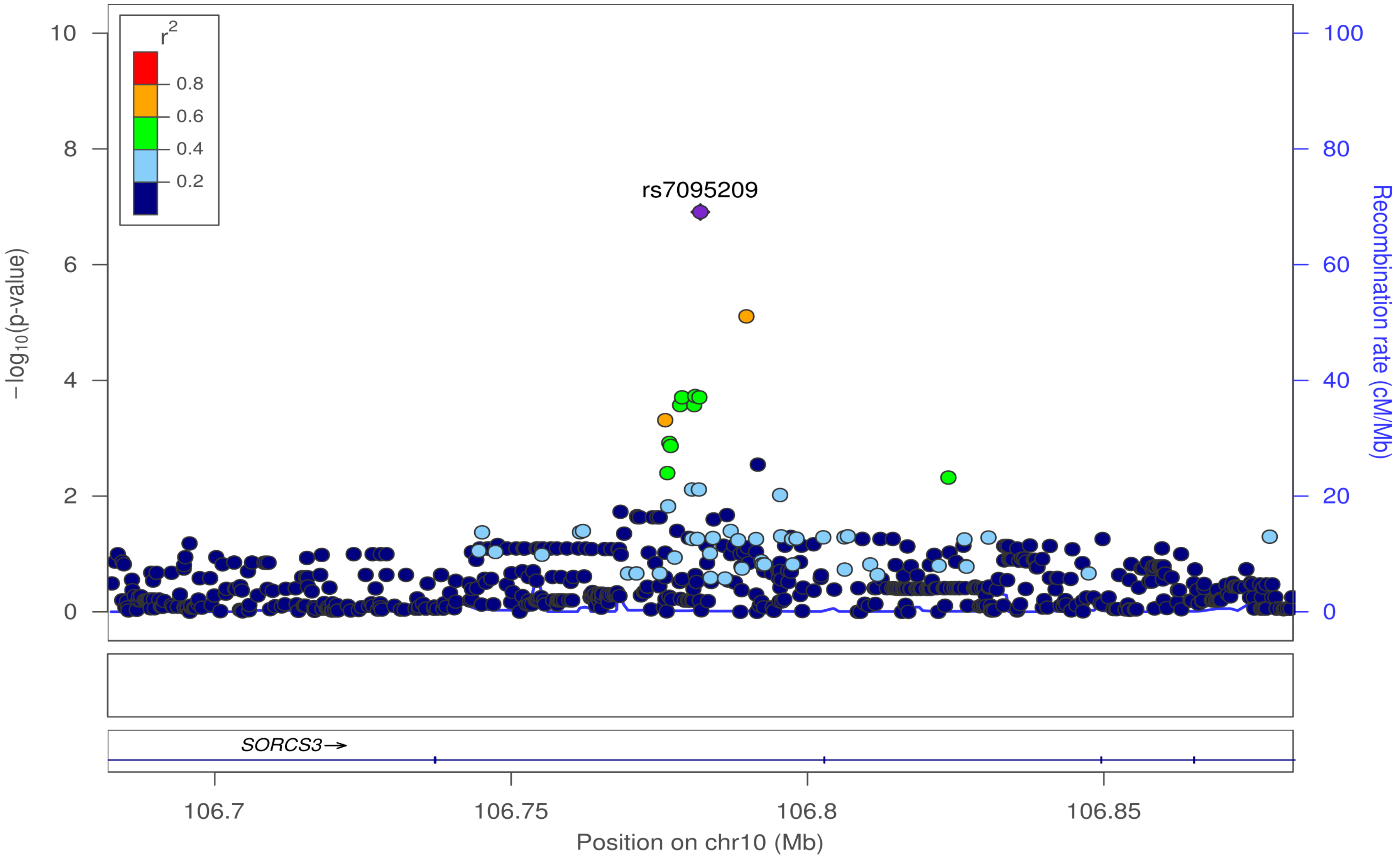

r2
0.8
0.6
0.4
0.2
rs7095209
10
8
6
4
2
0
−log10(p-value)
100
80
60
40
20
0
Recombination rate (cM/Mb)
SORCS3→
106.7
106.75
106.8
106.85
Position on chr10 (Mb)

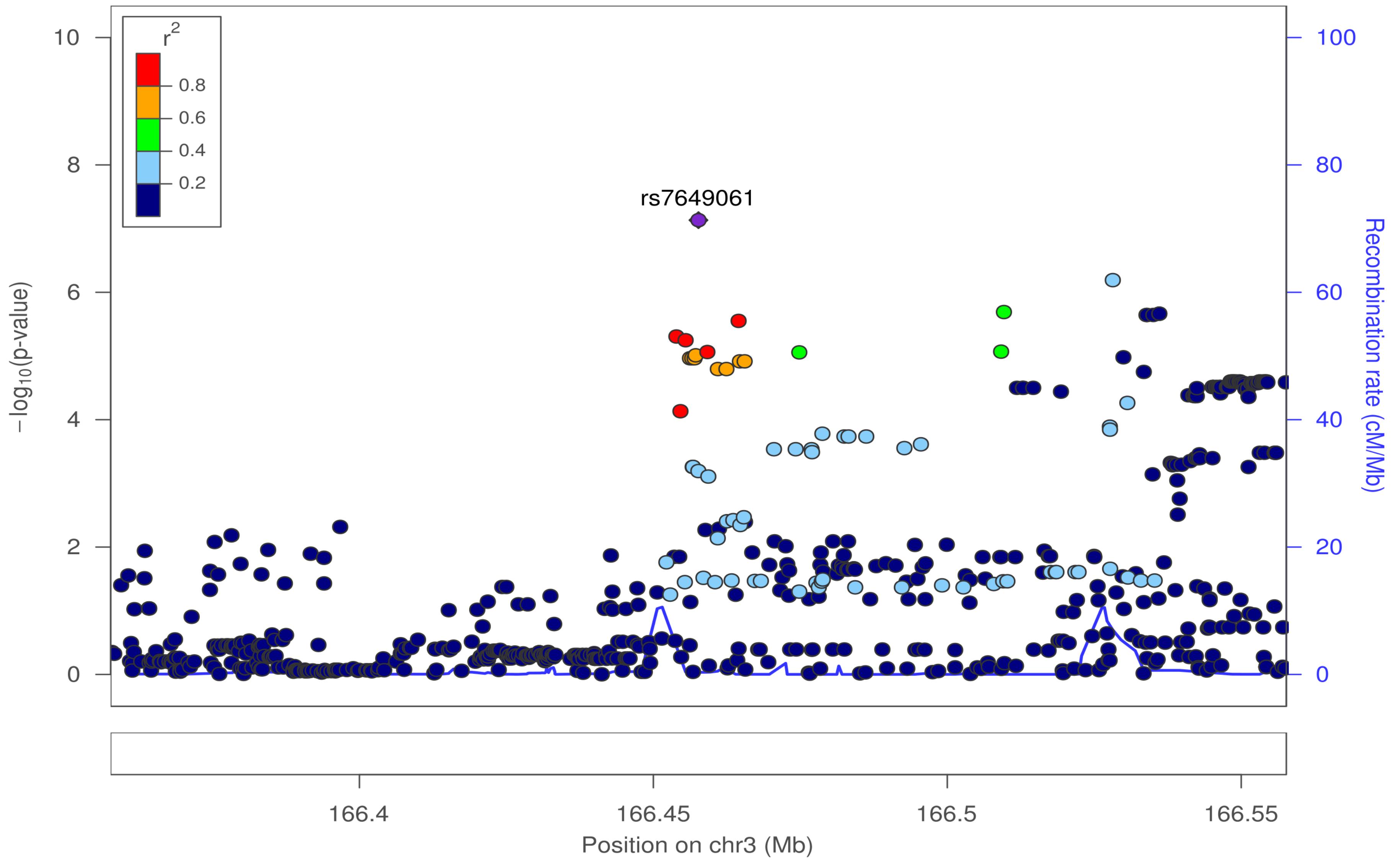

r2
0.8
0.6
0.4
0.2
rs7649061
−log10(p-value)
Recombination rate (cM/Mb)
10
8
6
4
2
0
100
80
60
40
20
0
166.4
166.45
166.5
166.55
Position on chr3 (Mb)

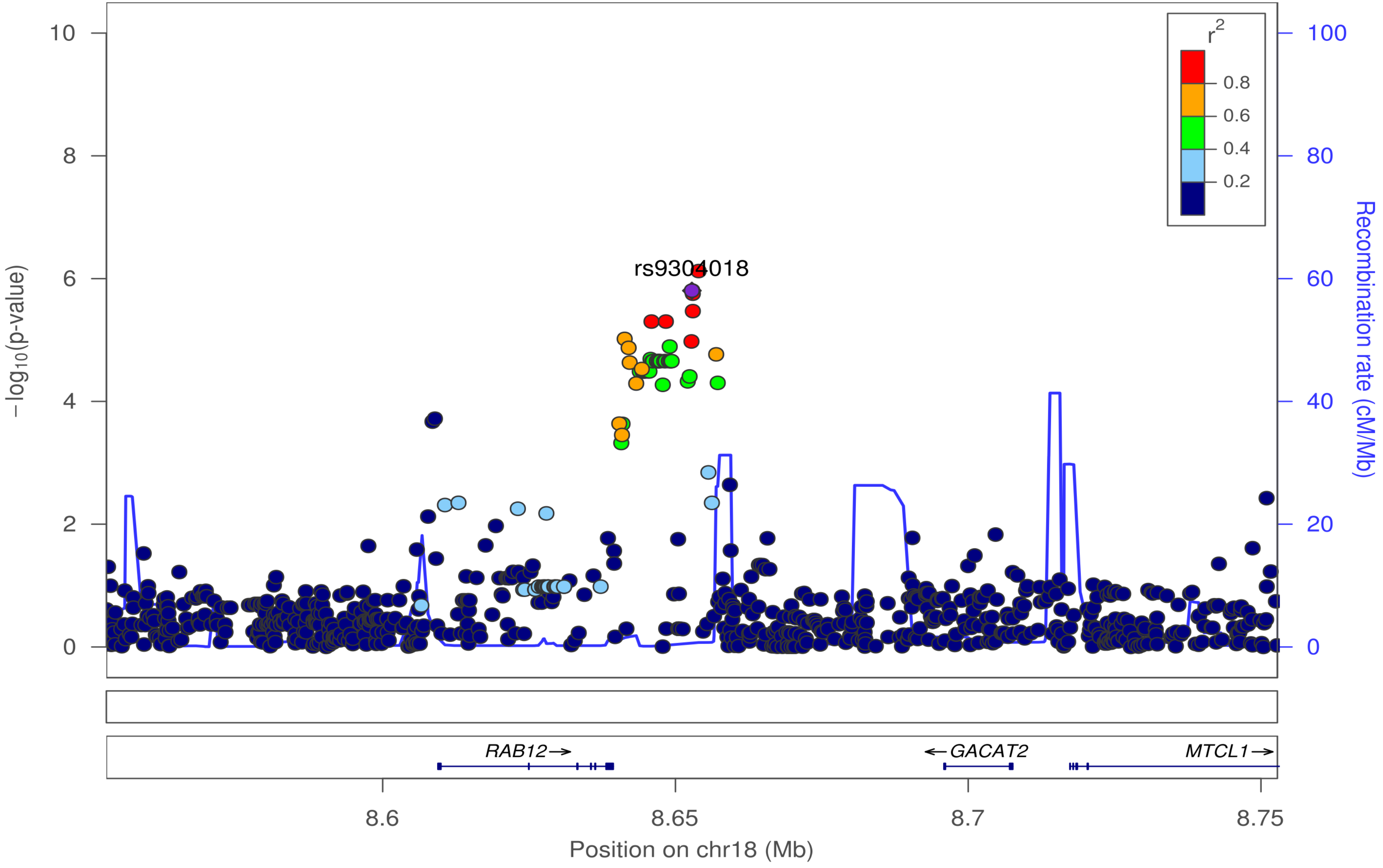

rs9304018
r2
0.8
0.6
0.4
0.2
10
8
6
4
2
0
−log10(p-value)
100
80
60
40
20
0
Recombination rate (cM/Mb)
RAB12→
←GACAT2
MTCL1→
8.6
8.65
8.7
8.75
Position on chr18 (Mb)

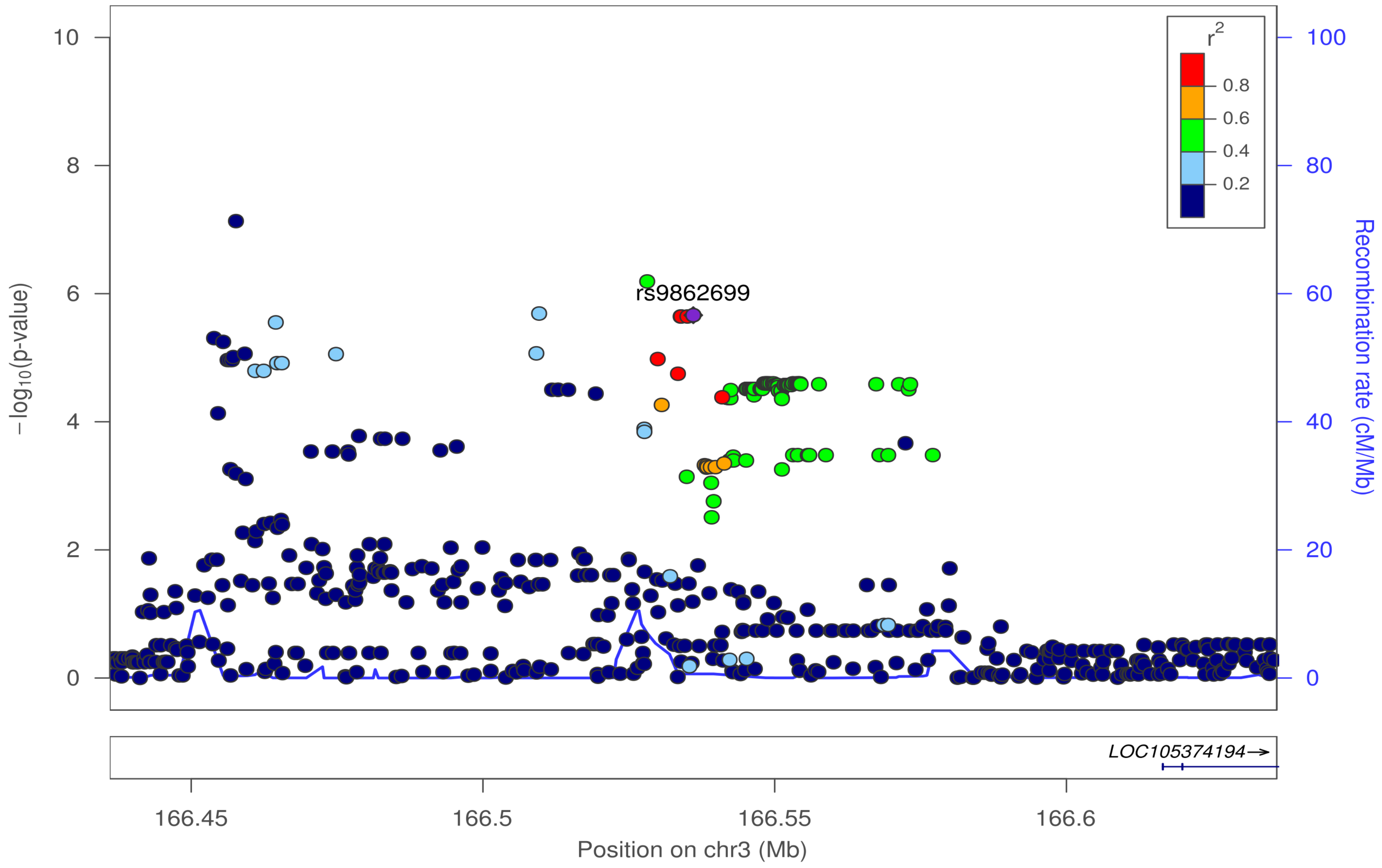

rs9862699
r²
0.8
0.6
0.4
0.2
−log10(p-value)
Recombination rate (cM/Mb)
LOC105374194→
166.45
166.5
166.55
166.6
Position on chr3 (Mb)

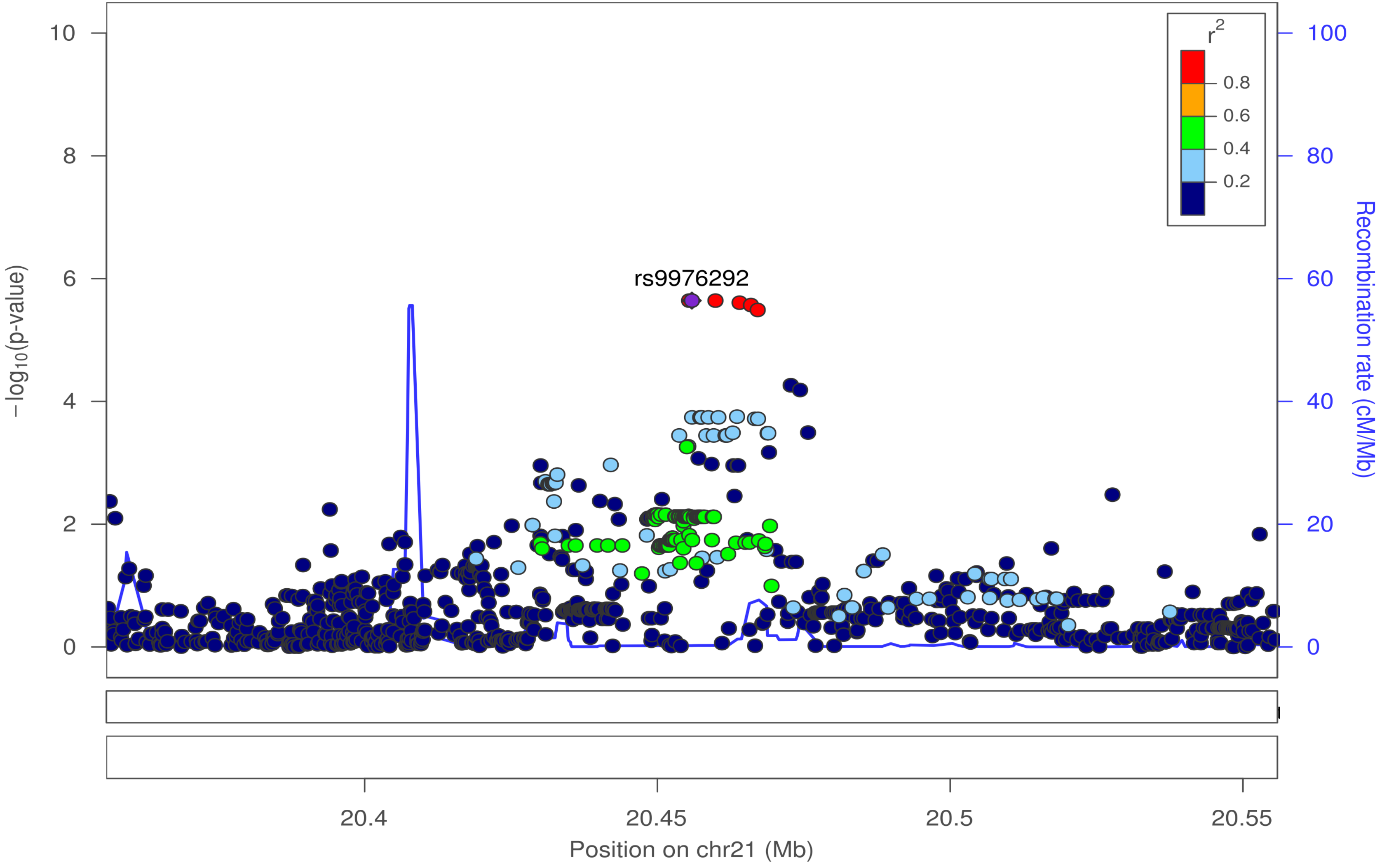

rs9976292
r2
0.8
0.6
0.4
0.2
10
8
6
4
2
0
−log10(p-value)
100
80
60
40
20
0
Recombination rate (cM/Mb)
20.4
20.45
20.5
20.55
Position on chr21 (Mb)

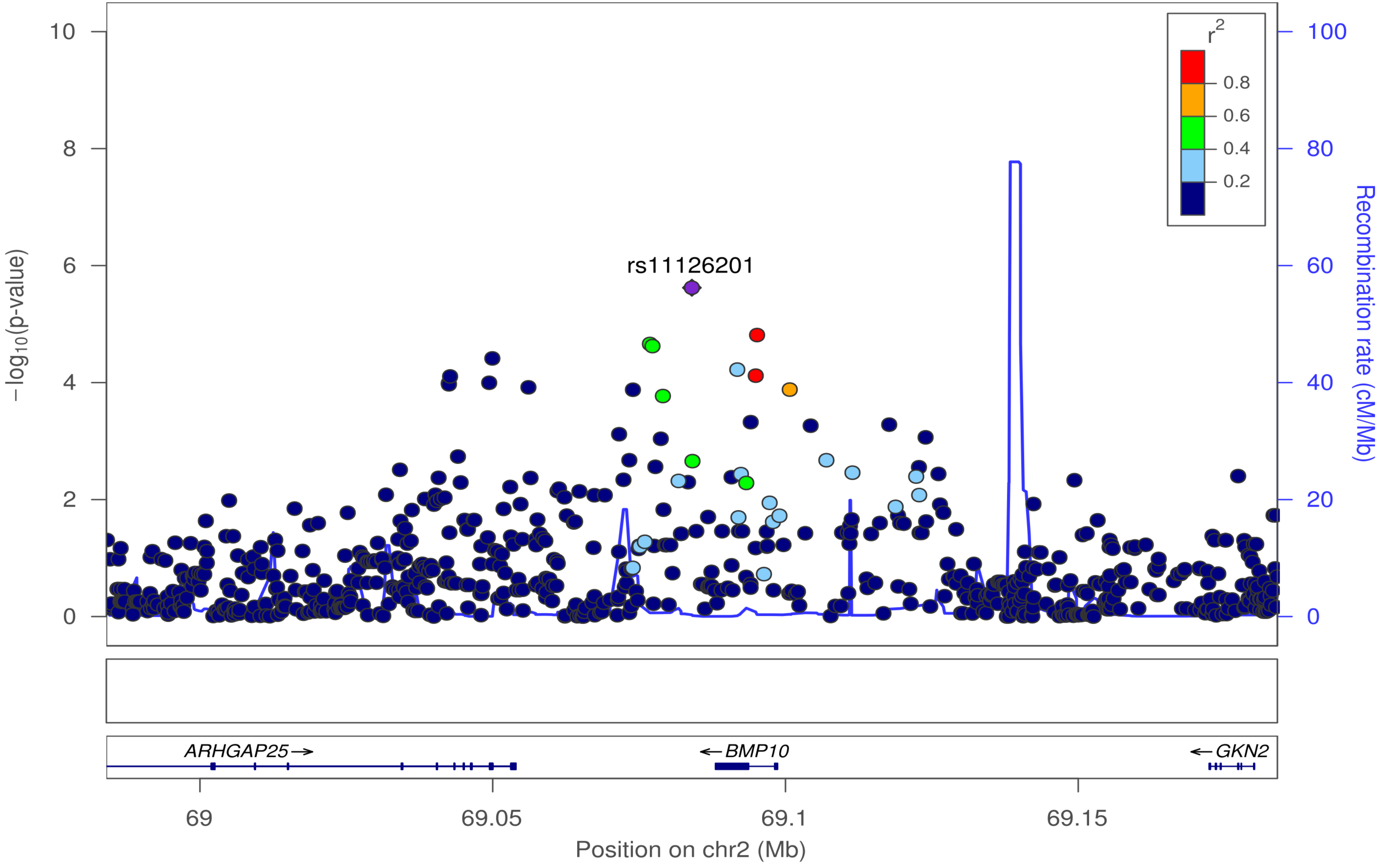

rs11126201
r2
0.8
0.6
0.4
0.2
10
8
6
4
2
0
−log10(p-value)
100
80
60
40
20
0
Recombination rate (cM/Mb)
ARHGAP25→
←BMP10
←GKN2
69
69.05
69.1
69.15
Position on chr2 (Mb)

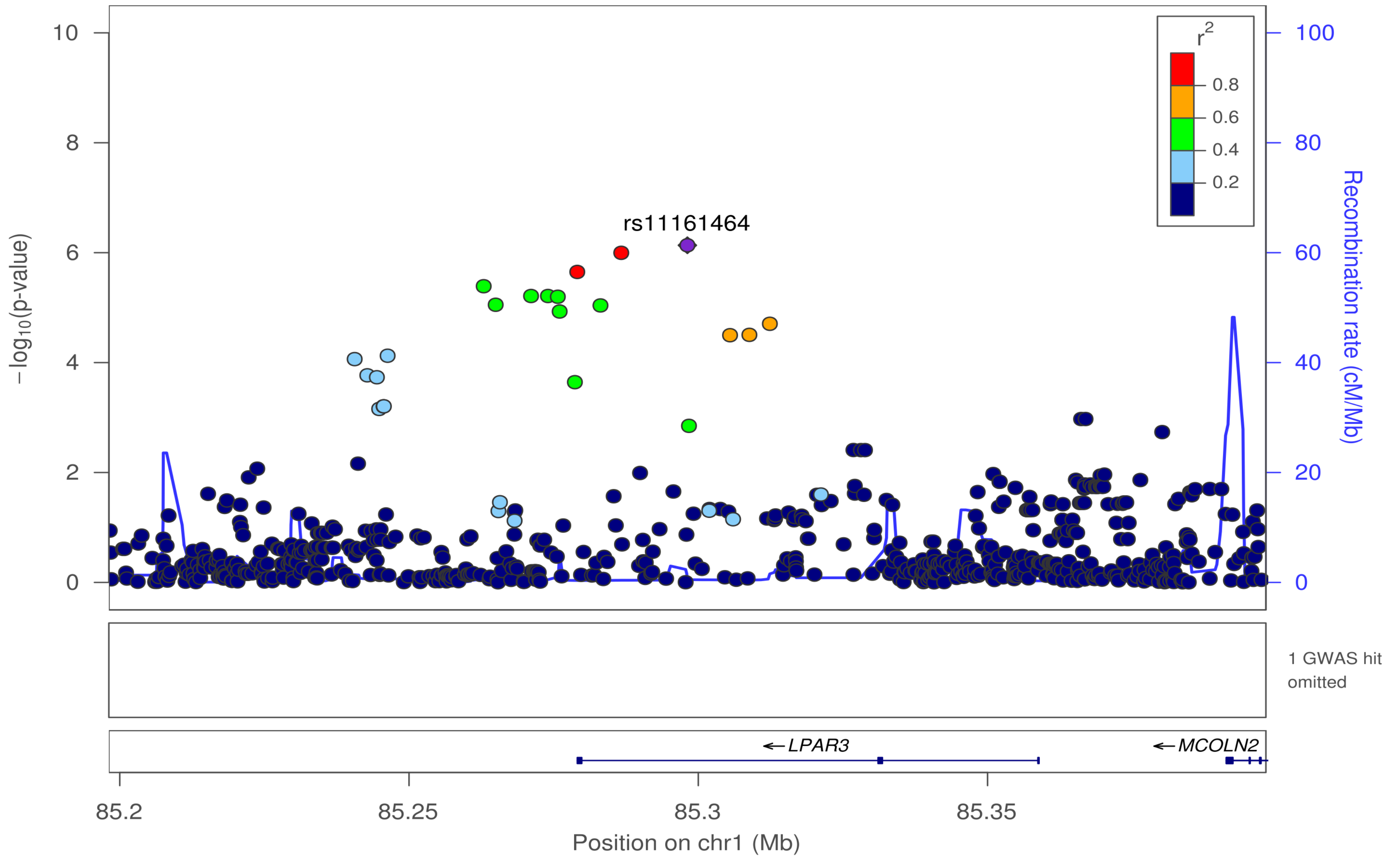
rs11161464
r2
0.8
0.6
0.4
0.2
−log10(p-value)
Recombination rate (cM/Mb)
1 GWAS hit omitted
LPAR3
MCOLN2
85.2
85.25
85.3
85.35
Position on chr1 (Mb)

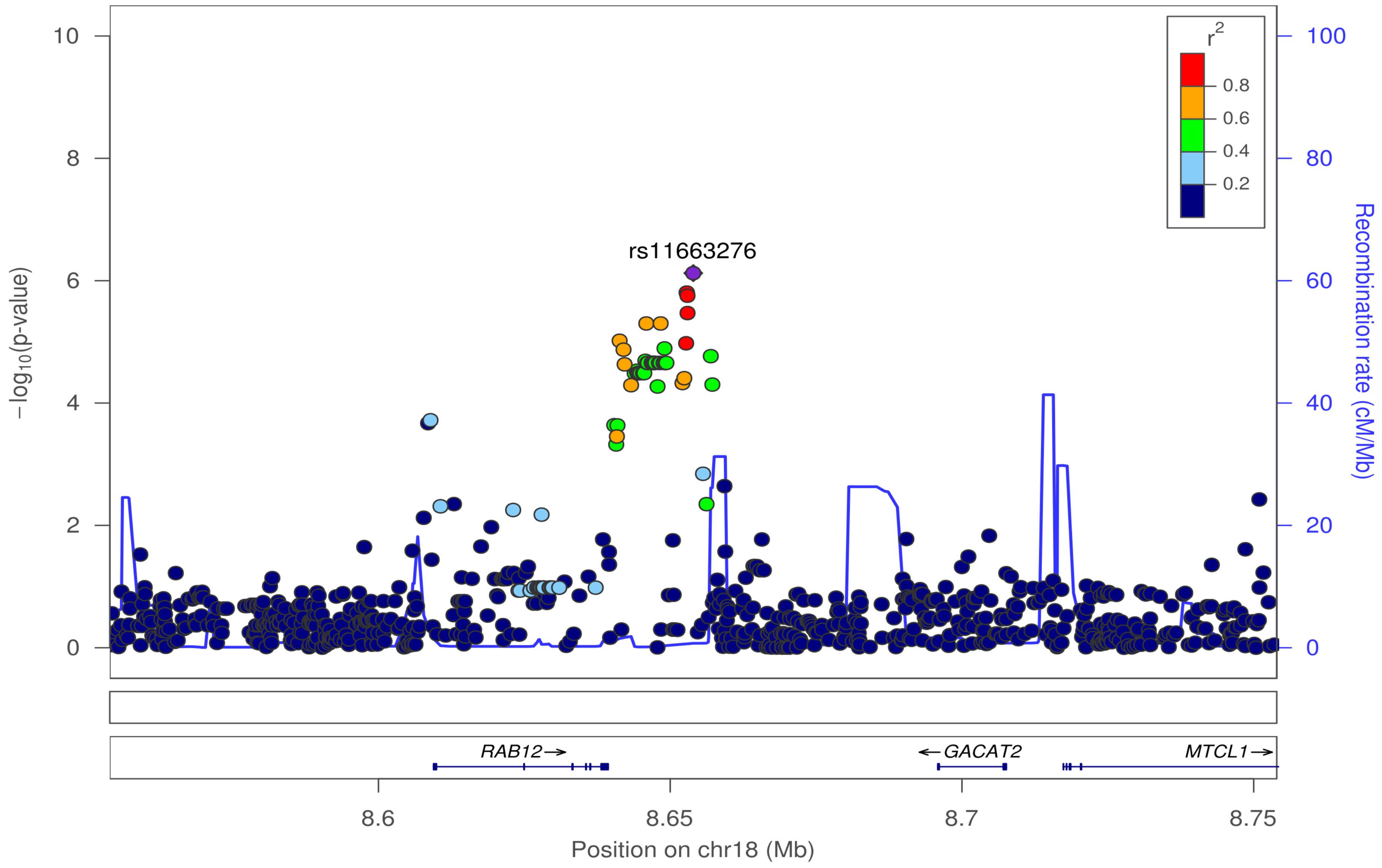

10
8
6
4
2
0
−log10(p-value)
100
80
60
40
20
0
Recombination rate (cM/Mb)
r2
0.8
0.6
0.4
0.2
rs11663276
RAB12→
←GACAT2
MTCL1→
8.6
8.65
8.7
8.75
Position on chr18 (Mb)

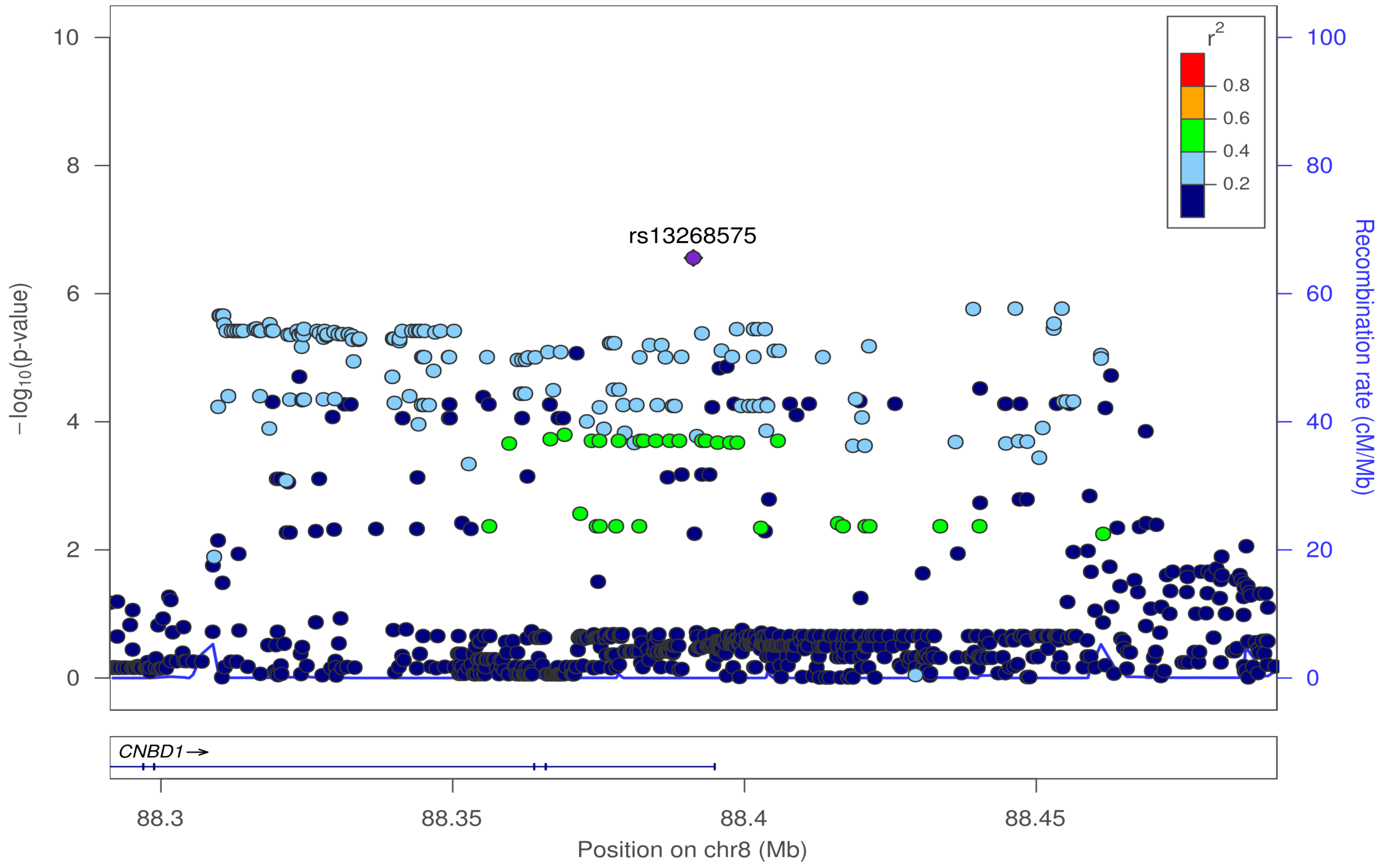

rs13268575
r2
0.8
0.6
0.4
0.2
10
8
6
4
2
0
−log10(p-value)
100
80
60
40
20
0
Recombination rate (cM/Mb)
CNBD1→
88.3
88.35
88.4
88.45
Position on chr8 (Mb)

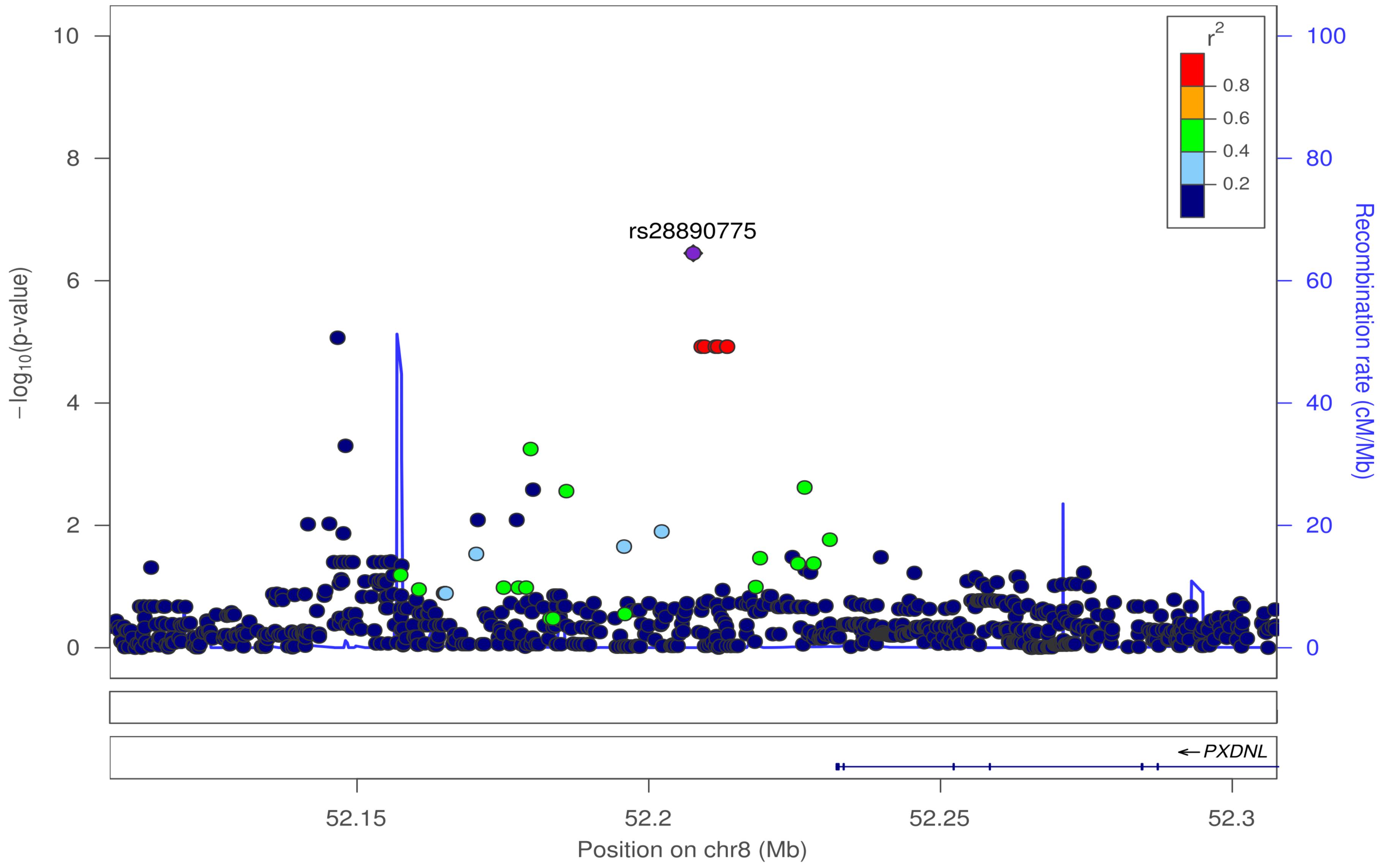

rs28890775
r2
0.8
0.6
0.4
0.2
0
2
4
6
8
10
−log10(p-value)
0
20
40
60
80
100
Recombination rate (cM/Mb)
← PXDNL
52.15
52.2
52.25
52.3
Position on chr8 (Mb)

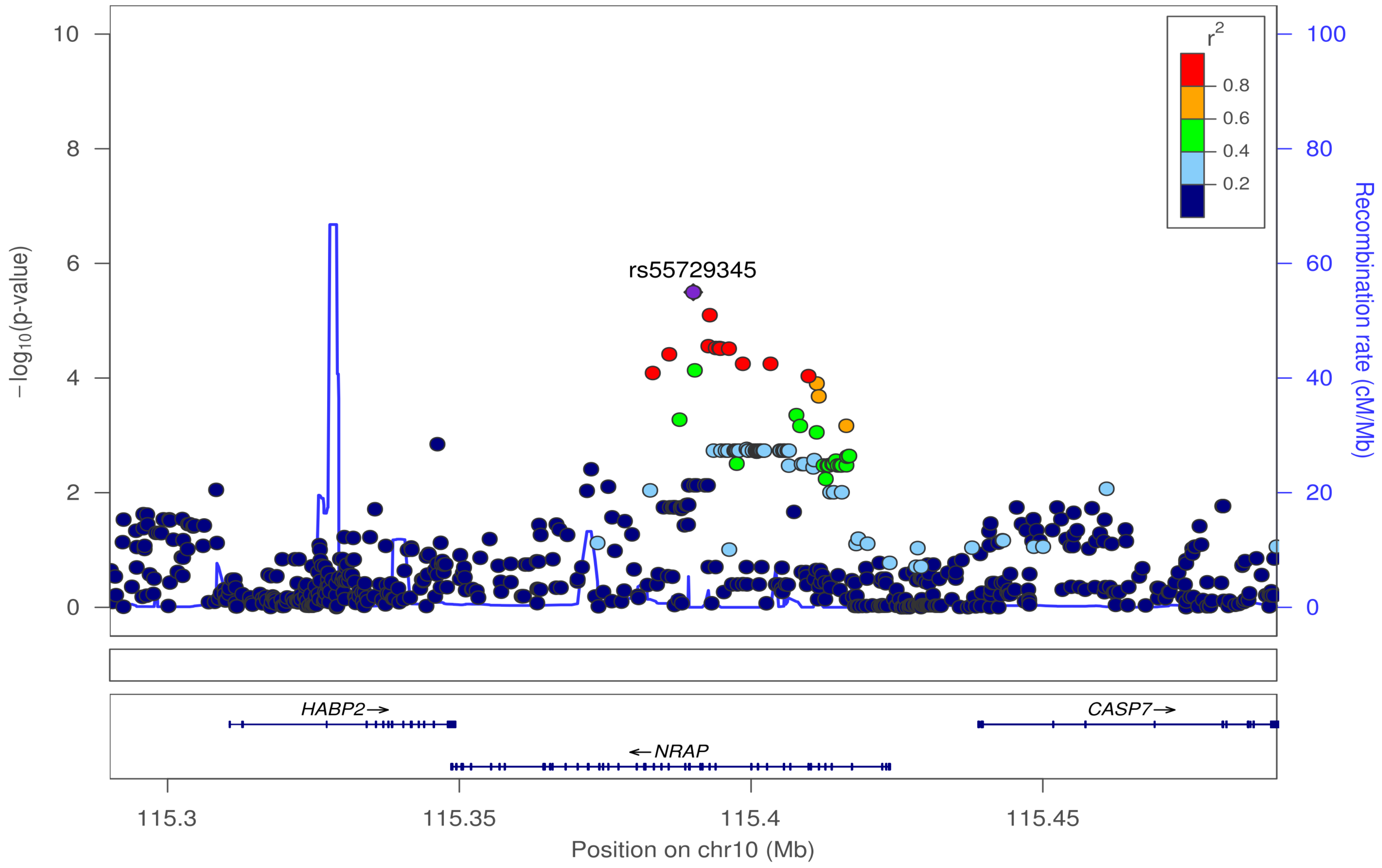
rs55729345
r2
0.8
0.6
0.4
0.2
−log10(p-value)
Recombination rate (cM/Mb)
HABP2→
←NRAP
CASP7→
Position on chr10 (Mb)
115.3
115.35
115.4
115.45

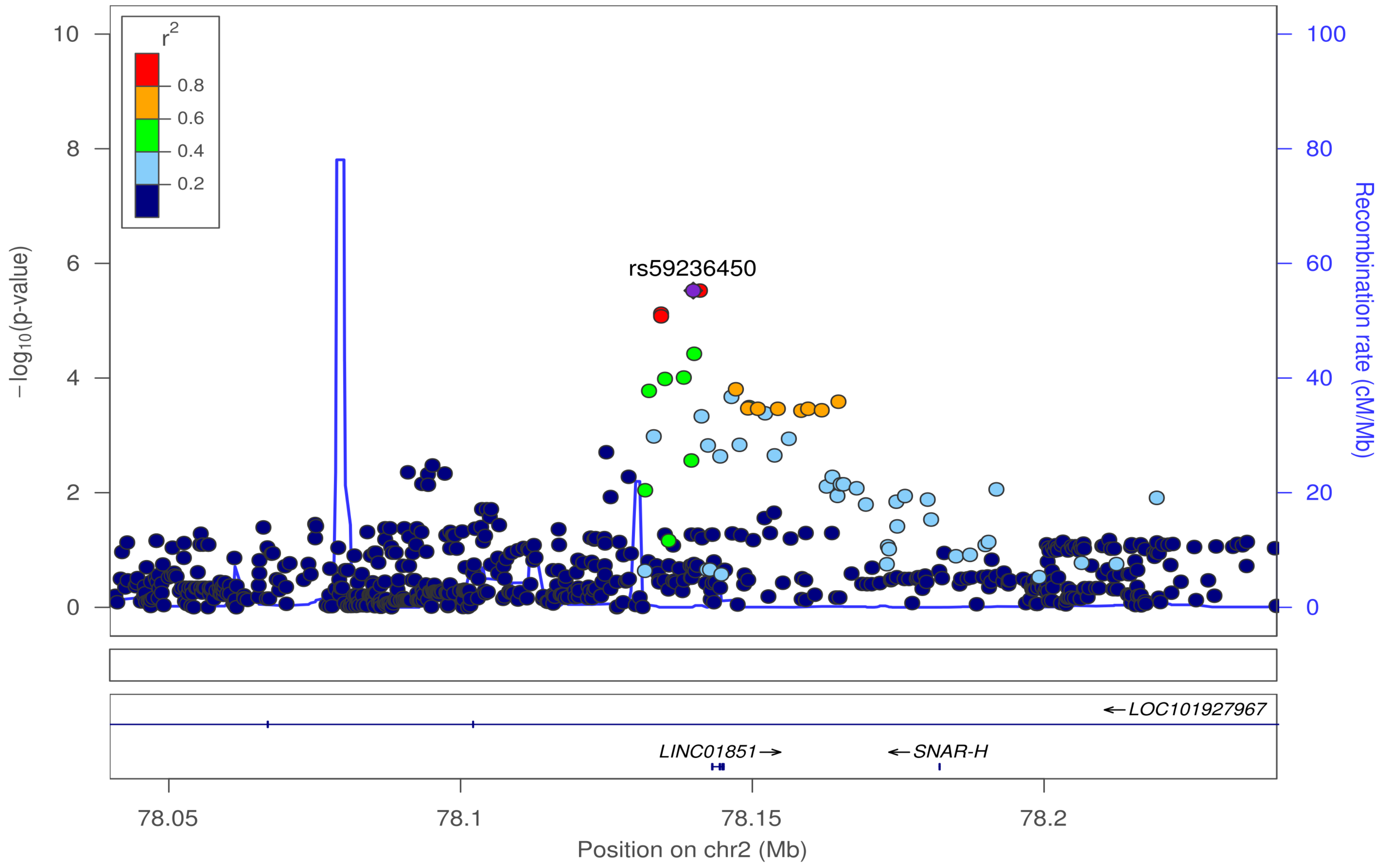
r2
0.8
0.6
0.4
0.2
rs59236450
10
8
6
4
2
0
100
80
60
40
20
0
−log10(p-value)
Recombination rate (cM/Mb)
←LOC101927967
LINC01851→
←SNAR-H
78.05
78.1
78.15
78.2
Position on chr2 (Mb)

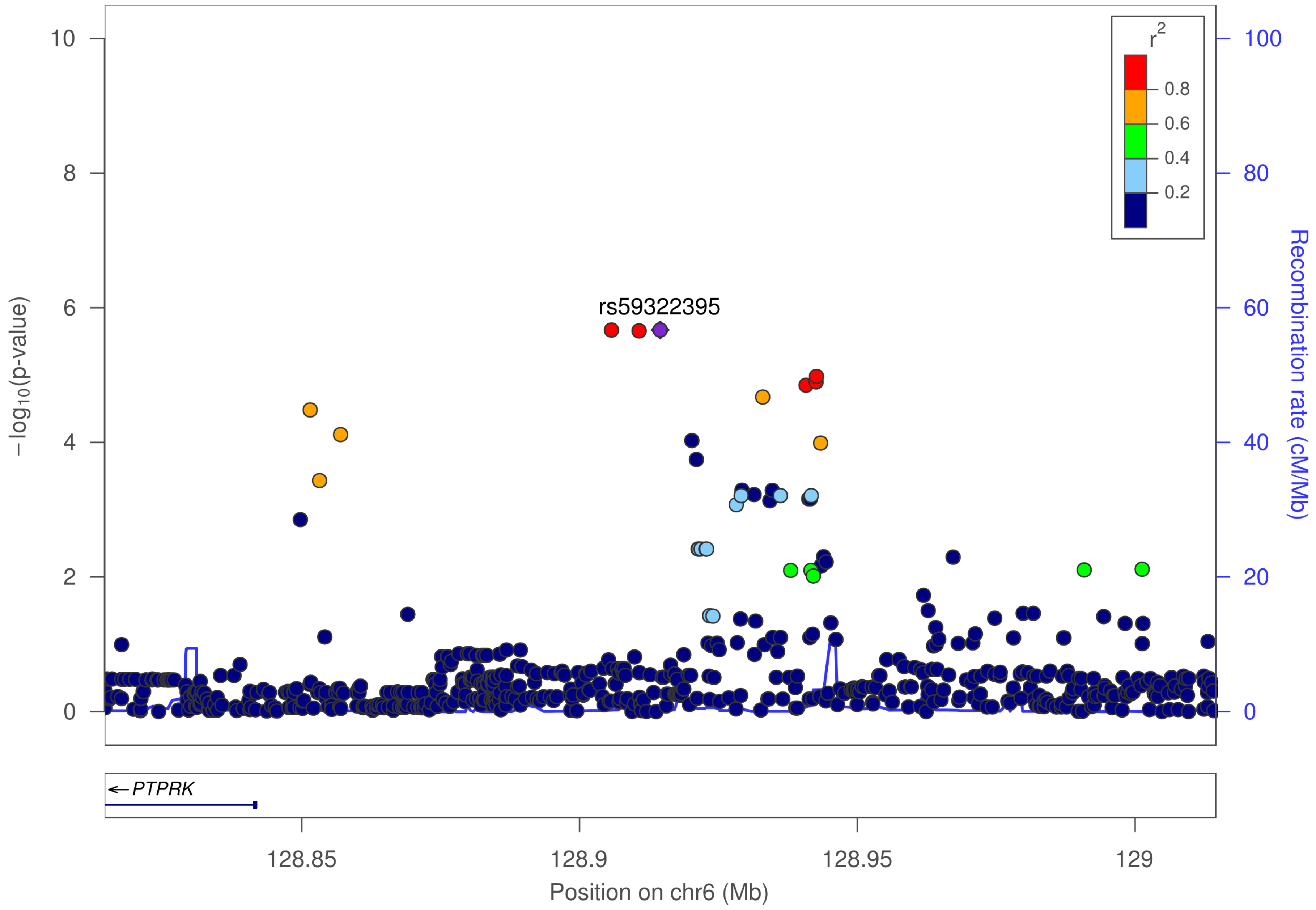

r2
0.8
0.6
0.4
0.2
rs59322395
10
8
6
4
2
0
100
80
60
40
20
0
−log10(p-value)
Recombination rate (cM/Mb)
PTPRK
128.85
128.9
128.95
129
Position on chr6 (Mb)

Locus …

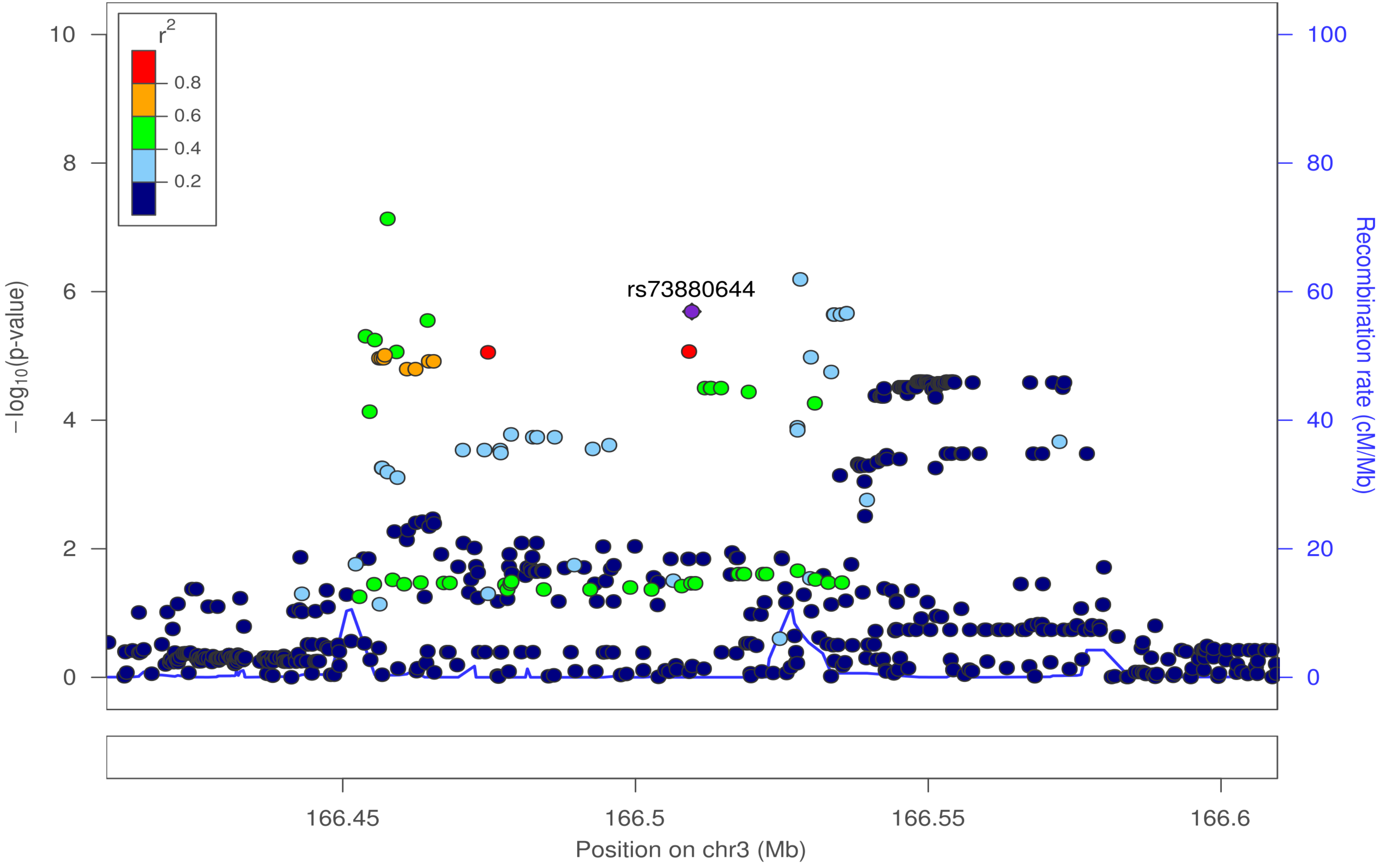

r2
0.8
0.6
0.4
0.2
rs73880644
10
8
6
4
2
0
−log10(p-value)
100
80
60
40
20
0
Recombination rate (cM/Mb)
166.45
166.5
166.55
166.6
Position on chr3 (Mb)

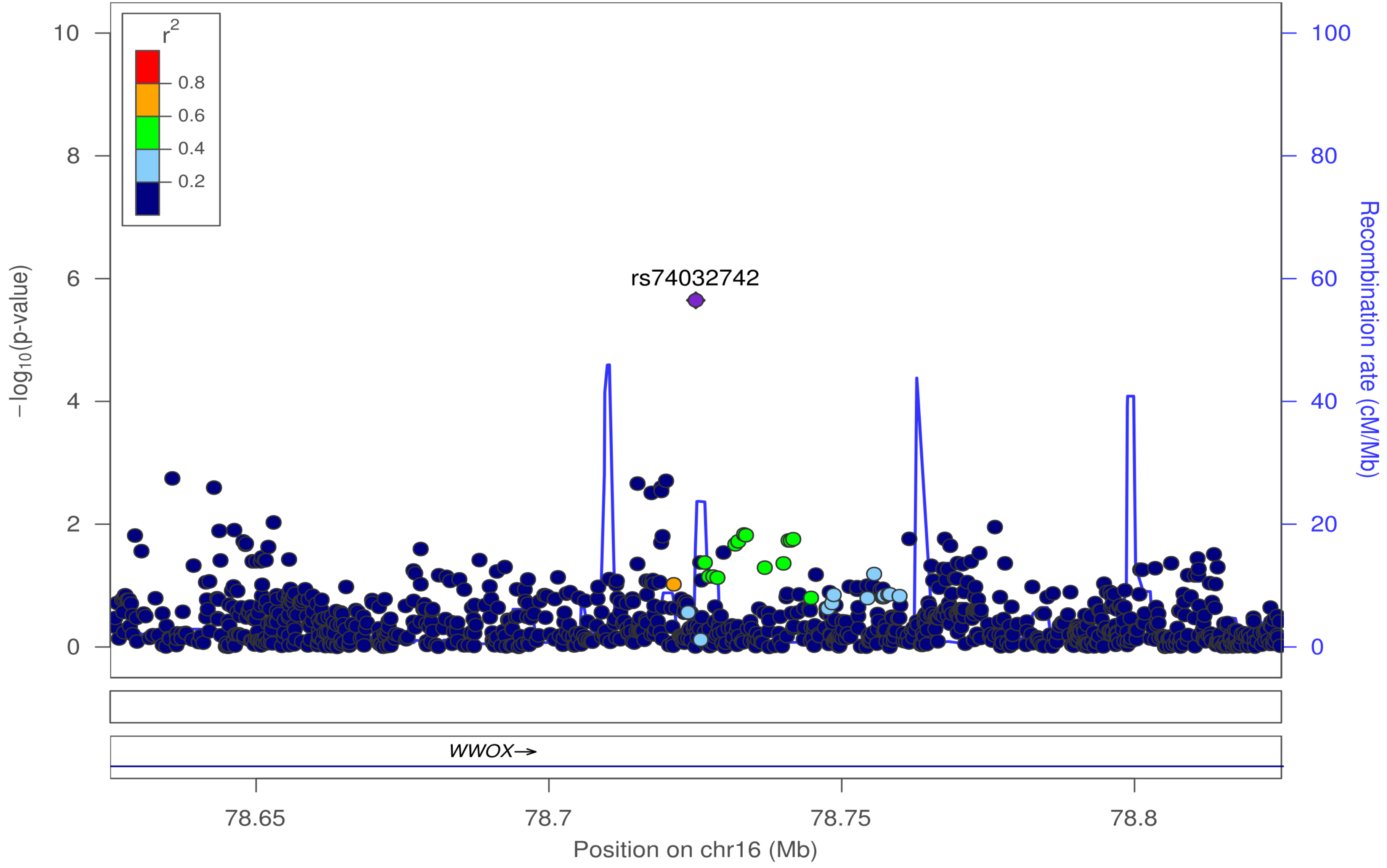

rs74032742
r2
0.8
0.6
0.4
0.2
−log10(p-value)
Recombination rate (cM/Mb)
0
2
4
6
8
10
0
20
40
60
80
100
WWOX→
78.65
78.7
78.75
78.8
Position on chr16 (Mb)

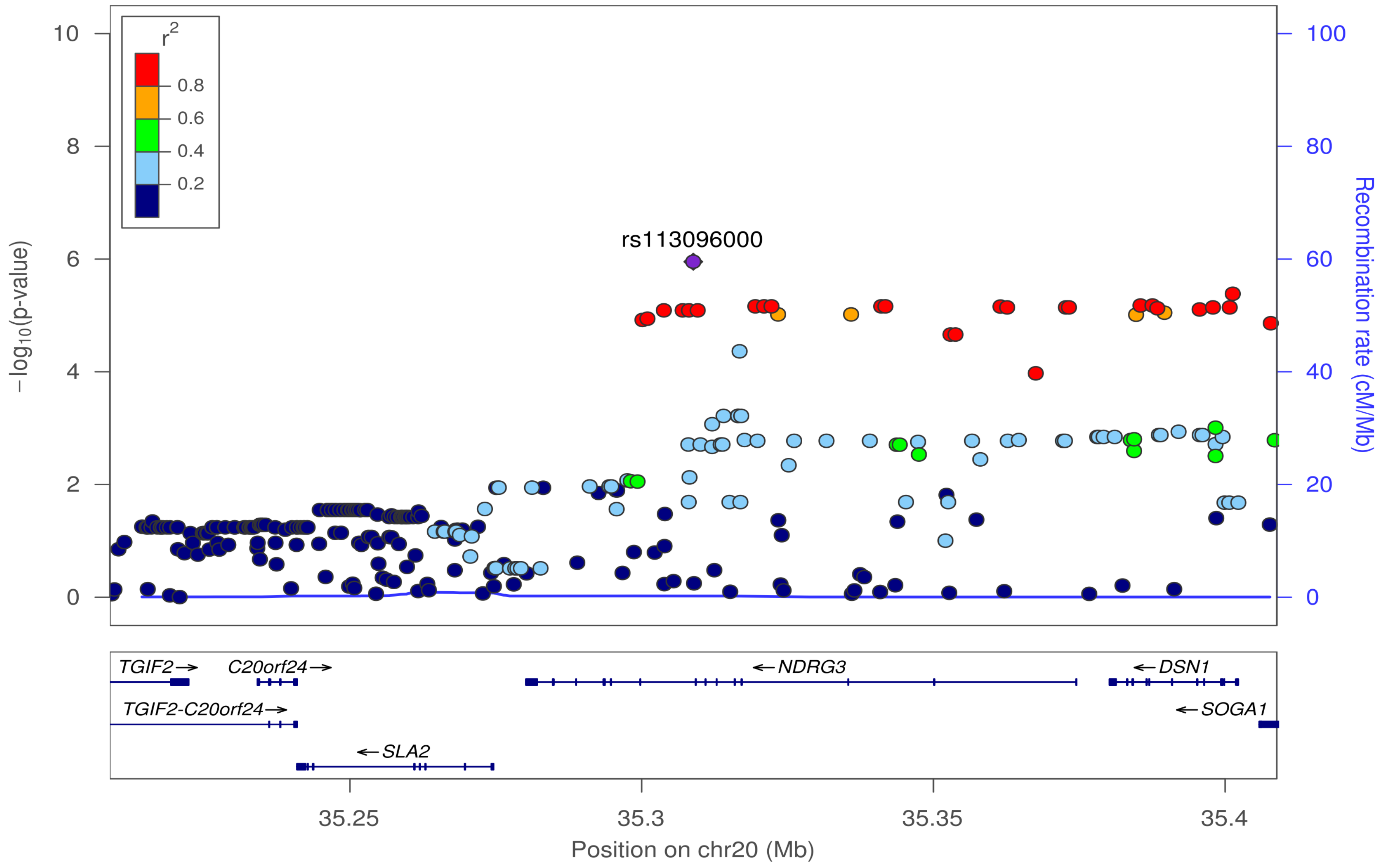

r2
0.8
0.6
0.4
0.2
rs113096000
−log10(p-value)
Recombination rate (cM/Mb)
10
8
6
4
2
0
100
80
60
40
20
0
TGIF2→
C20orf24→
←NDRG3
←DSN1
TGIF2-C20orf24→
←SOGA1
←SLA2
35.25
35.3
35.35
35.4
Position on chr20 (Mb)

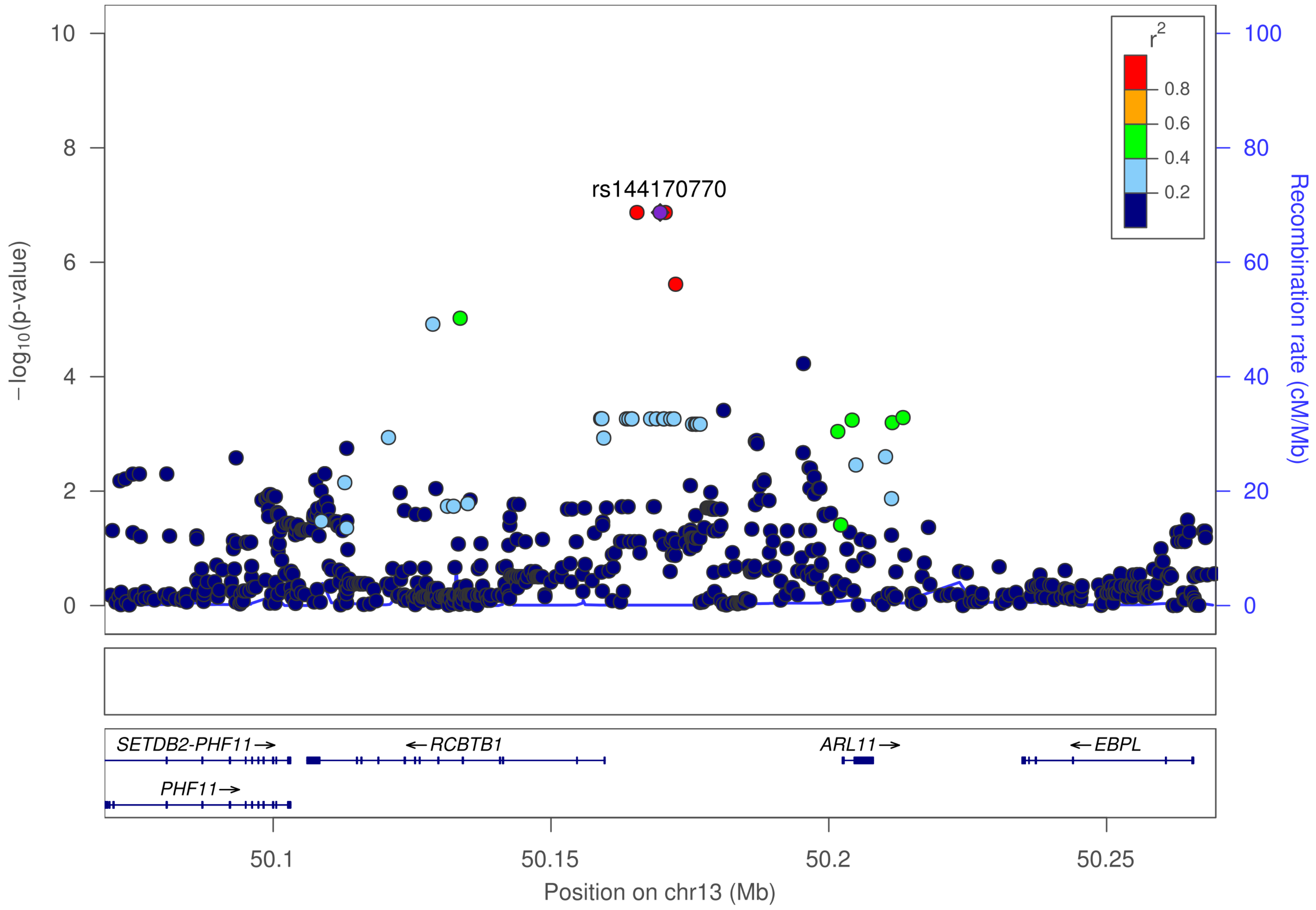

rs144170770
r2
0.8
0.6
0.4
0.2
−log10(p-value)
Recombination rate (cM/Mb)
SETDB2-PHF11→
←RCBTB1
ARL11→
←EBPL
PHF11→
50.1
50.15
50.2
50.25
Position on chr13 (Mb)

Supplementary Figure 1b:

Regional association plots of selected loci (p-values) in Navrongo. Distinct genomic risk loci were defined as LD-independent regions (r 2 separated by 100 kb and containing one or more SNPs with a suggestive association (p-values<1E-05). For each locus , the plots shows the –log10 transformed –value of each SNP on the y-axis and base pair positions along the chromosomes on the x-axis. Genes overlapping the locus are displayed below the plot. SNPs are colored by their LD value with the lead SNP in the region, and those LD values has been generated from the two study populations combined.

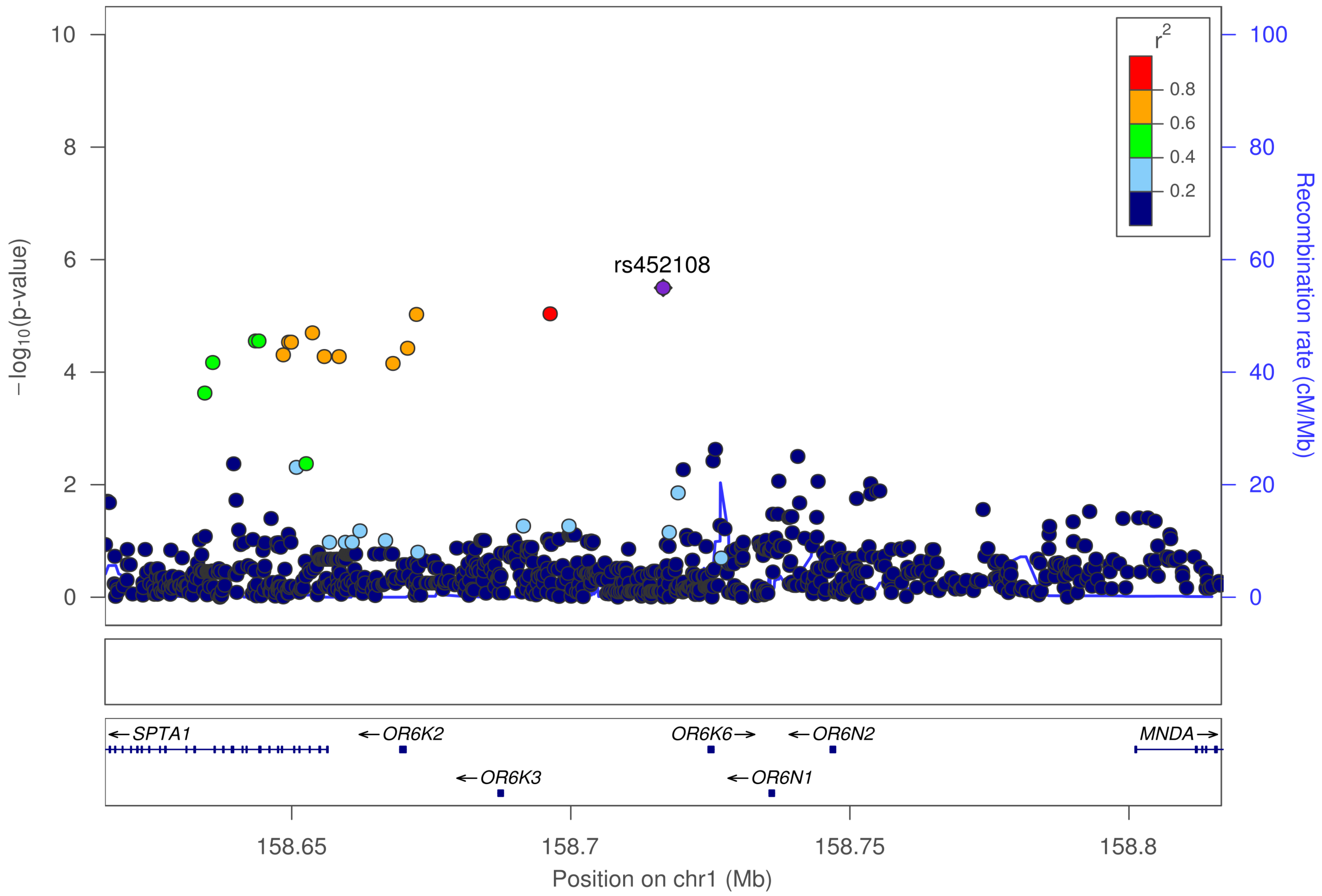

10
8
6
4
2
0
−log10(p-value)
rs452108
r2
0.8
0.6
0.4
0.2
100
80
60
40
20
0
Recombination rate (cM/Mb)
←SPTA1
←OR6K2
←OR6K3
OR6K6→
←OR6N2
←OR6N1
MNDA→
158.65
158.7
158.75
158.8
Position on chr1 (Mb)

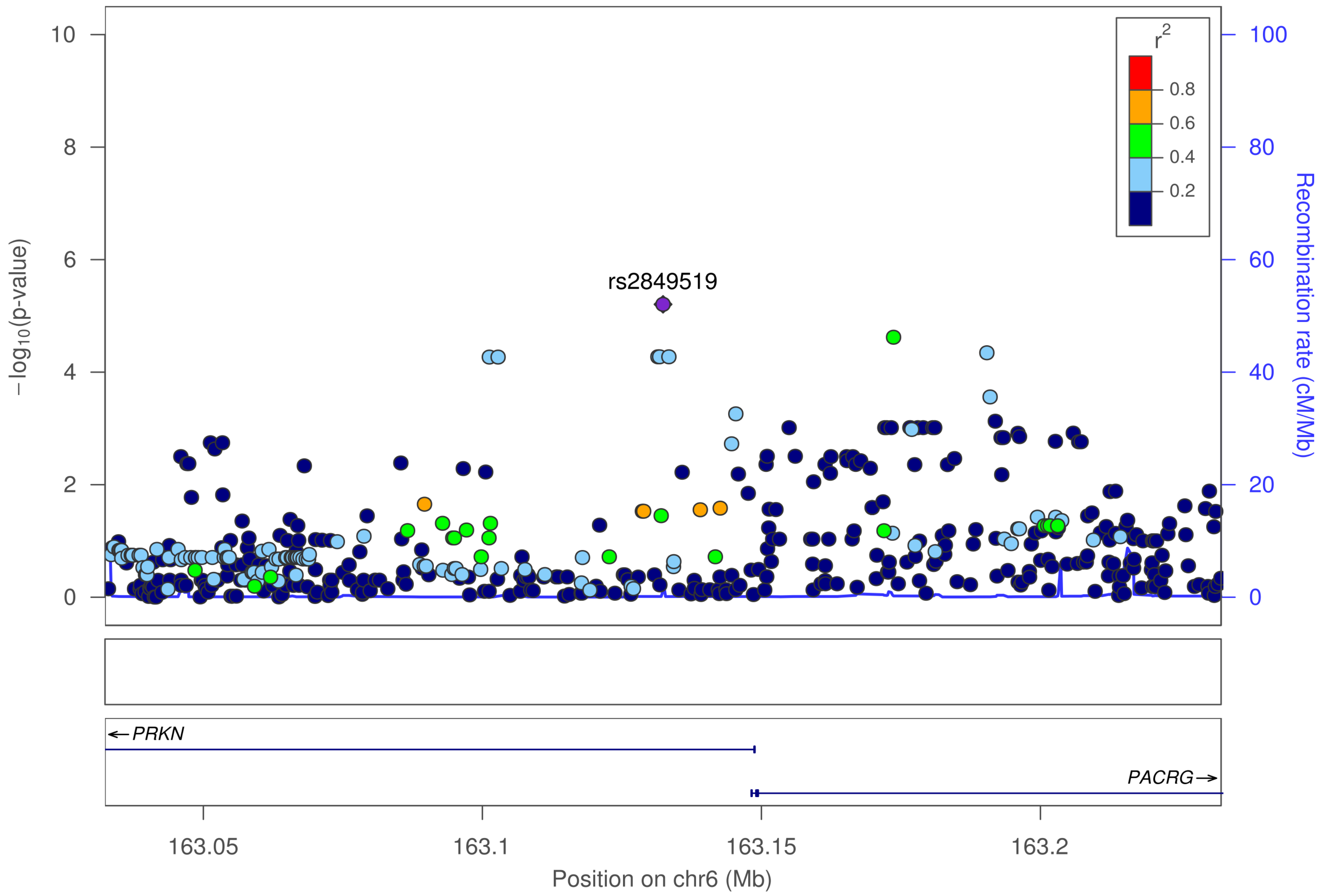

rs2849519
r2
0.8
0.6
0.4
0.2
10
8
6
4
2
0
−log10(p-value)
100
80
60
40
20
0
Recombination rate (cM/Mb)
← PRKN
PACRG →
163.05
163.1
163.15
163.2
Position on chr6 (Mb)

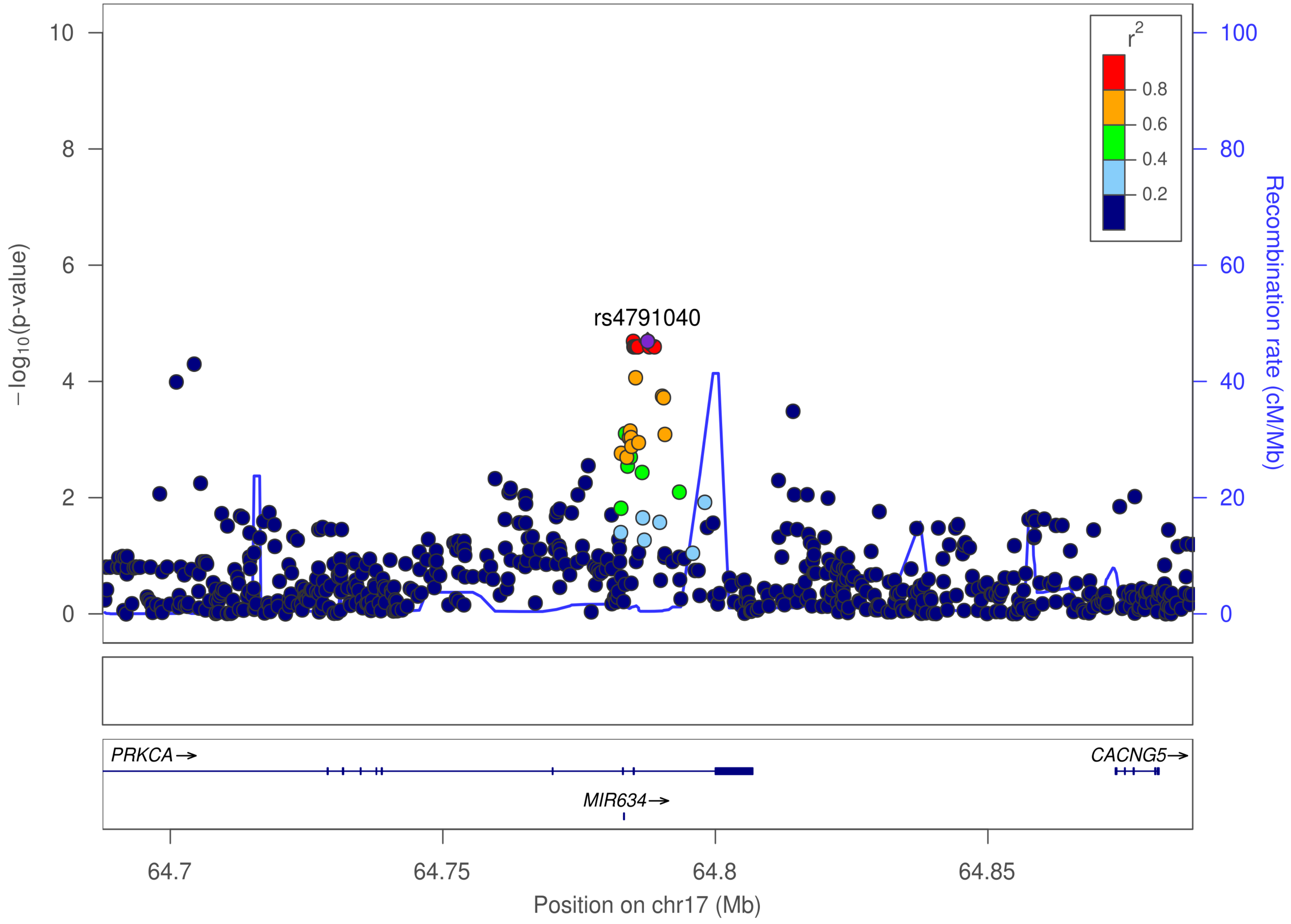

r2
0.8
0.6
0.4
0.2
rs4791040
−log10(p-value)
Recombination rate (cM/Mb)
10
8
6
4
2
0
100
80
60
40
20
0
PRKCA→
CACNG5→
MIR634→
64.7
64.75
64.8
64.85
Position on chr17 (Mb)

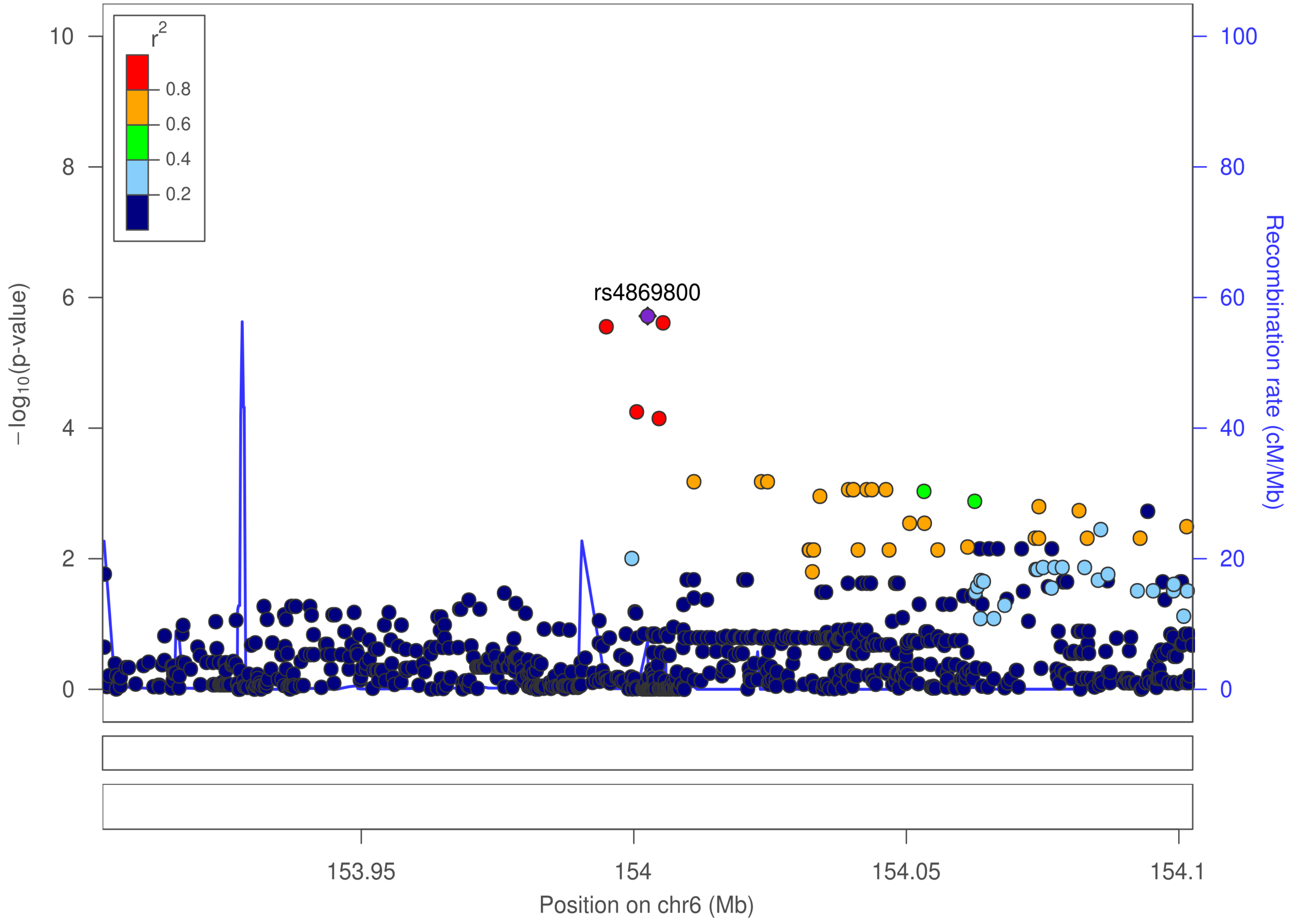

r2
0.8
0.6
0.4
0.2
rs4869800
10
8
6
4
2
0
−log10(p-value)
100
80
60
40
20
0
Recombination rate (cM/Mb)
153.95
154
154.05
154.1
Position on chr6 (Mb)

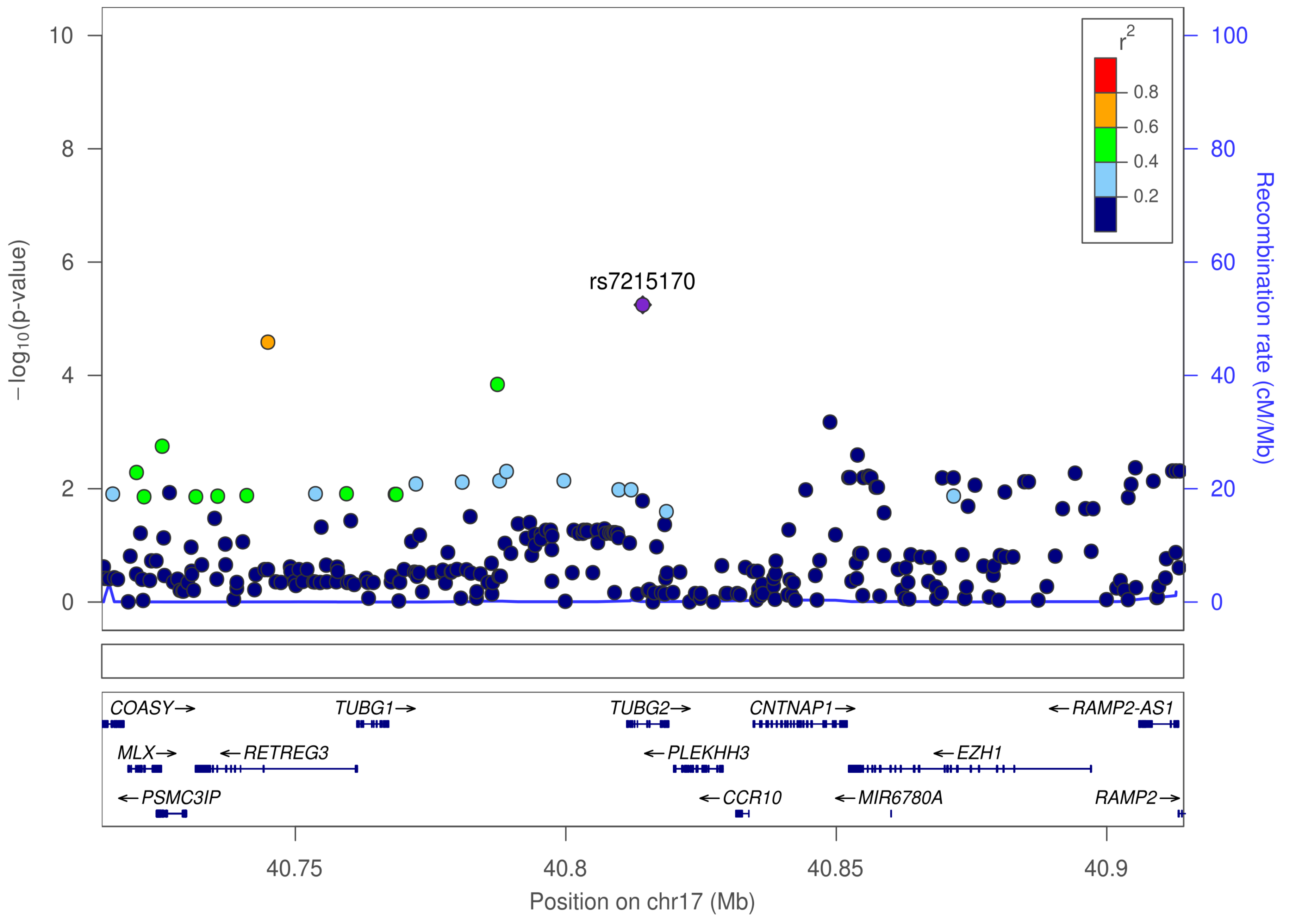

r2
0.8
0.6
0.4
0.2
rs7215170
−log10(p-value)
Recombination rate (cM/Mb)
0
2
4
6
8
10
0
20
40
60
80
100
COASY→
TUBG1→
TUBG2→
CNTNAP1→
←RAMP2-AS1
MLX→
←RETREG3
←PLEKHH3
←EZH1
←PSMC3IP
←CCR10
←MIR6780A
RAMP2→
40.75
40.8
40.85
40.9
Position on chr17 (Mb)

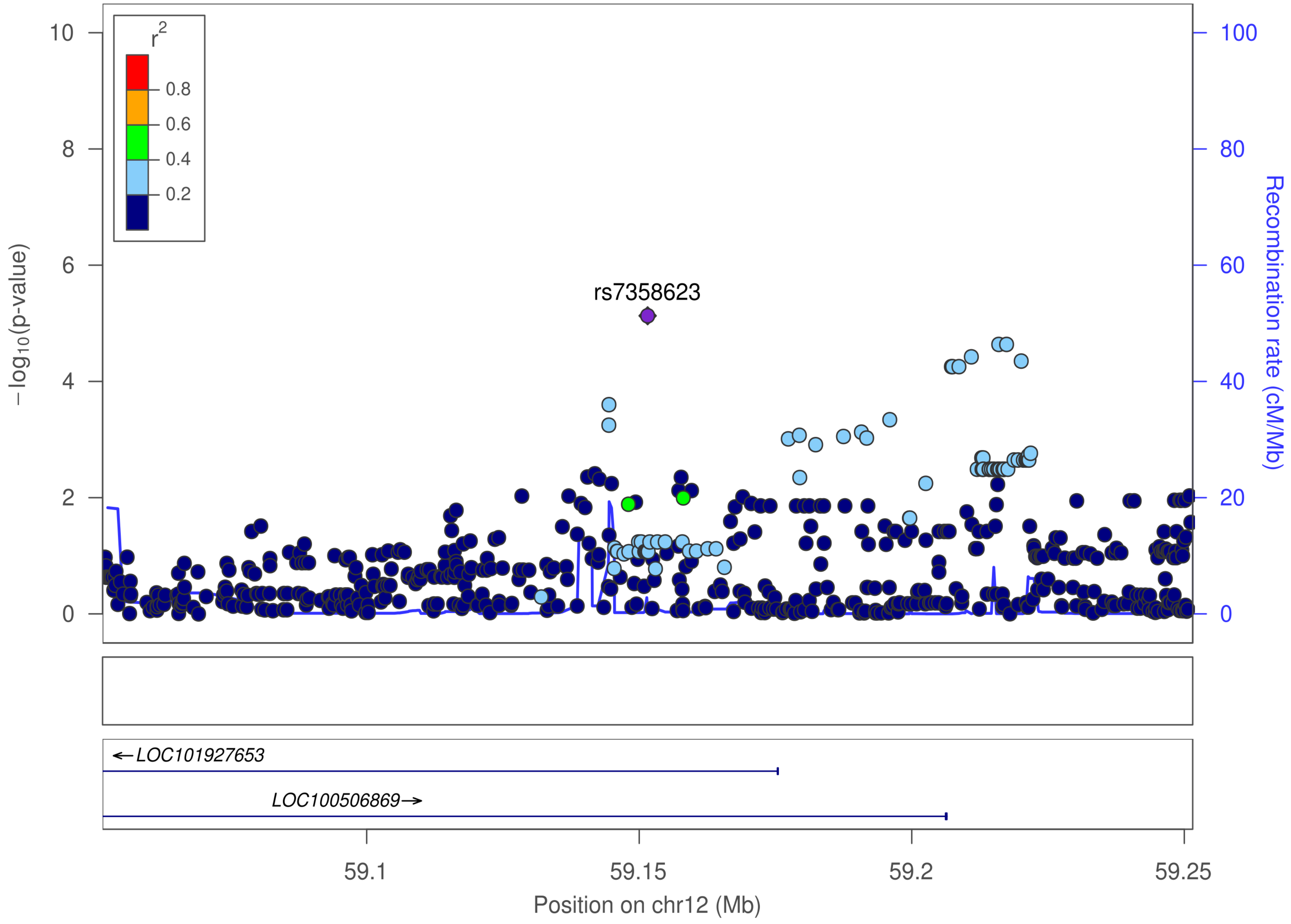

r²
0.8
0.6
0.4
0.2
rs7358623
−log10(p-value)
Recombination rate (cM/Mb)
0
2
4
6
8
10
0
20
40
60
80
100
←LOC101927653
LOC100506869→
59.1
59.15
59.2
59.25
Position on chr12 (Mb)

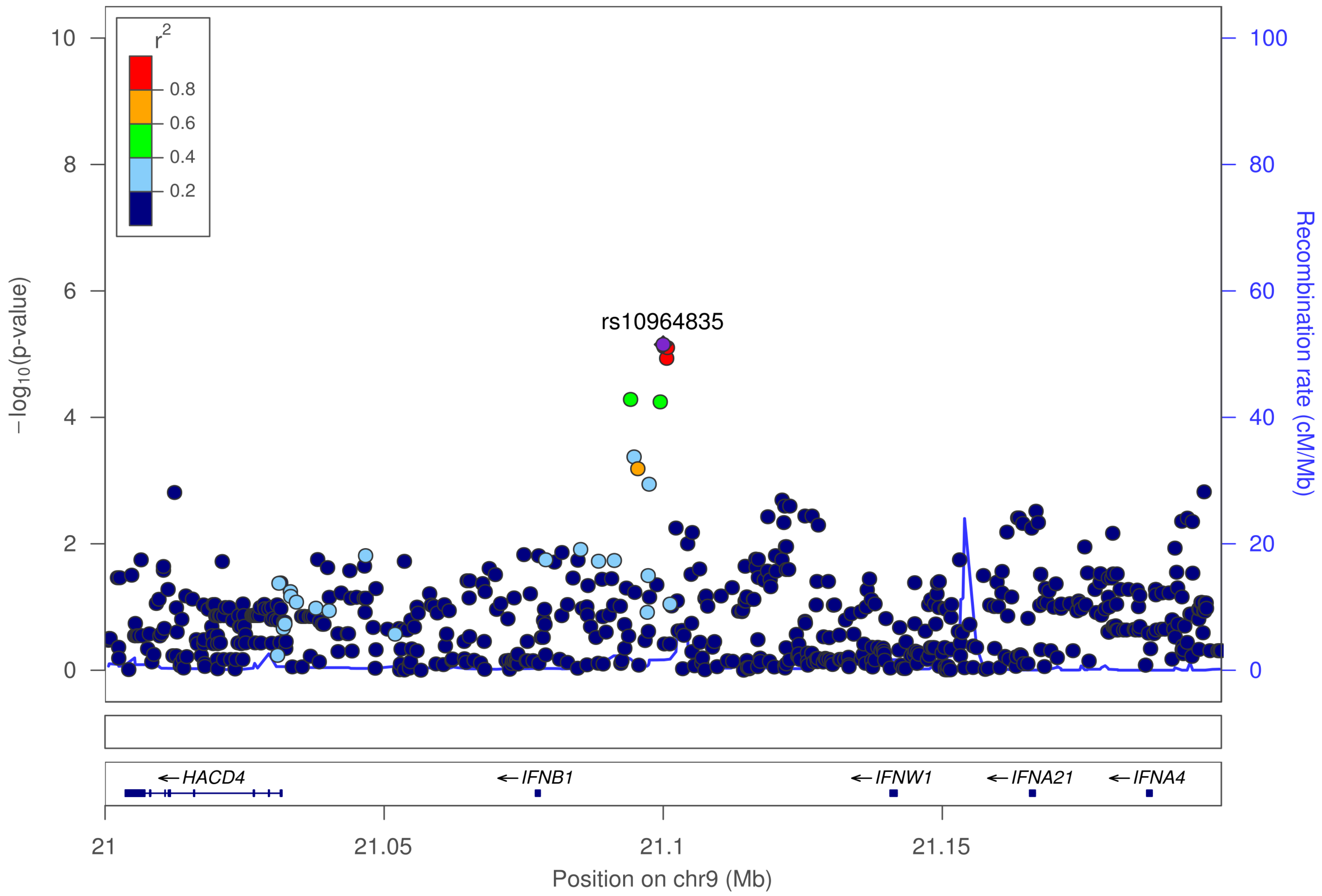
r2
0.8
0.6
0.4
0.2
rs10964835
−log10(p-value)
0
2
4
6
8
10
Recombination rate (cM/Mb)
0
20
40
60
80
100
HACD4
IFNB1
IFNW1
IFNA21
IFNA4
21
21.05
21.1
21.15
Position on chr9 (Mb)

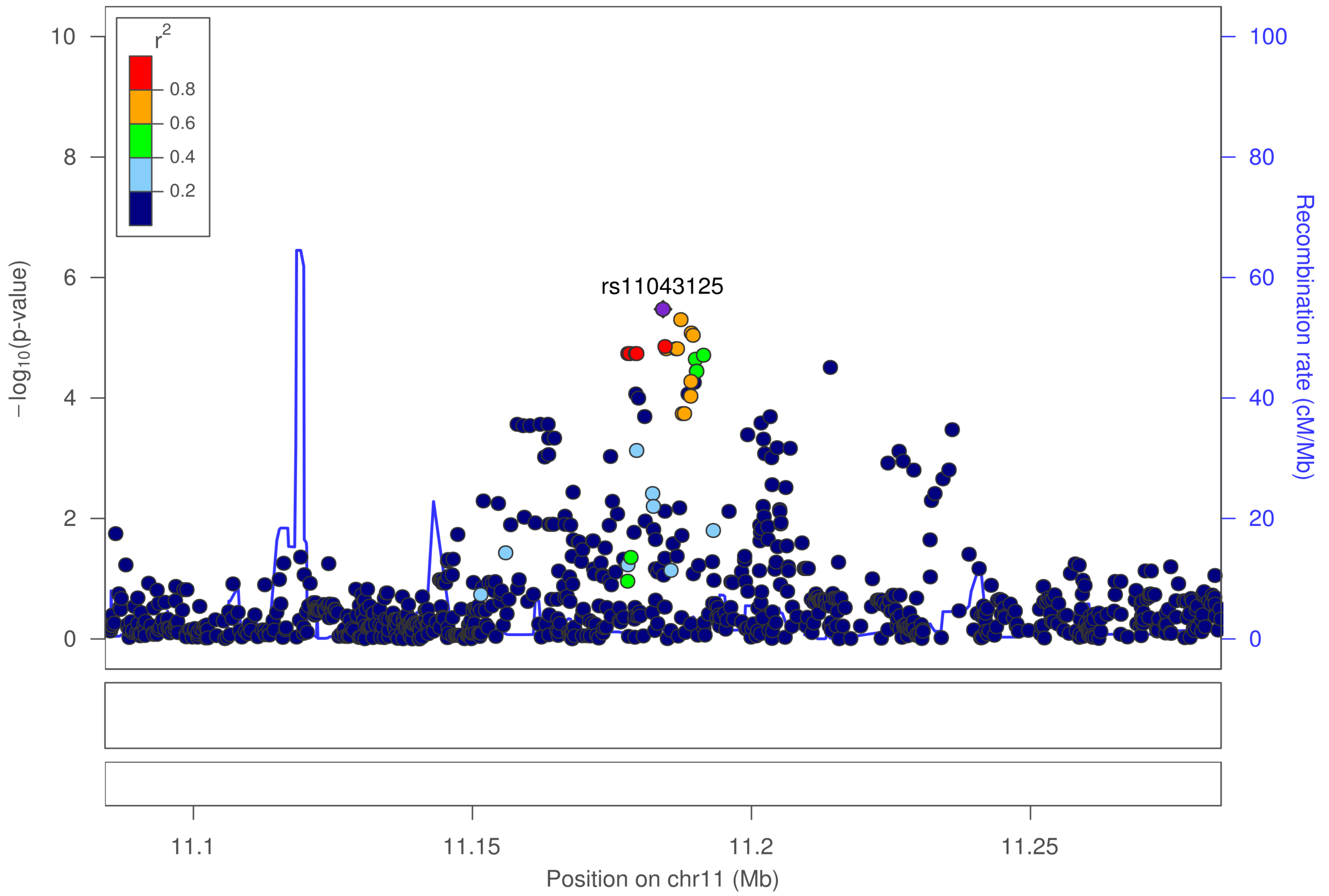

r2
0.8
0.6
0.4
0.2
rs11043125
−log10(p-value)
Recombination rate (cM/Mb)
0
2
4
6
8
10
0
20
40
60
80
100
11.1
11.15
11.2
11.25
Position on chr11 (Mb)

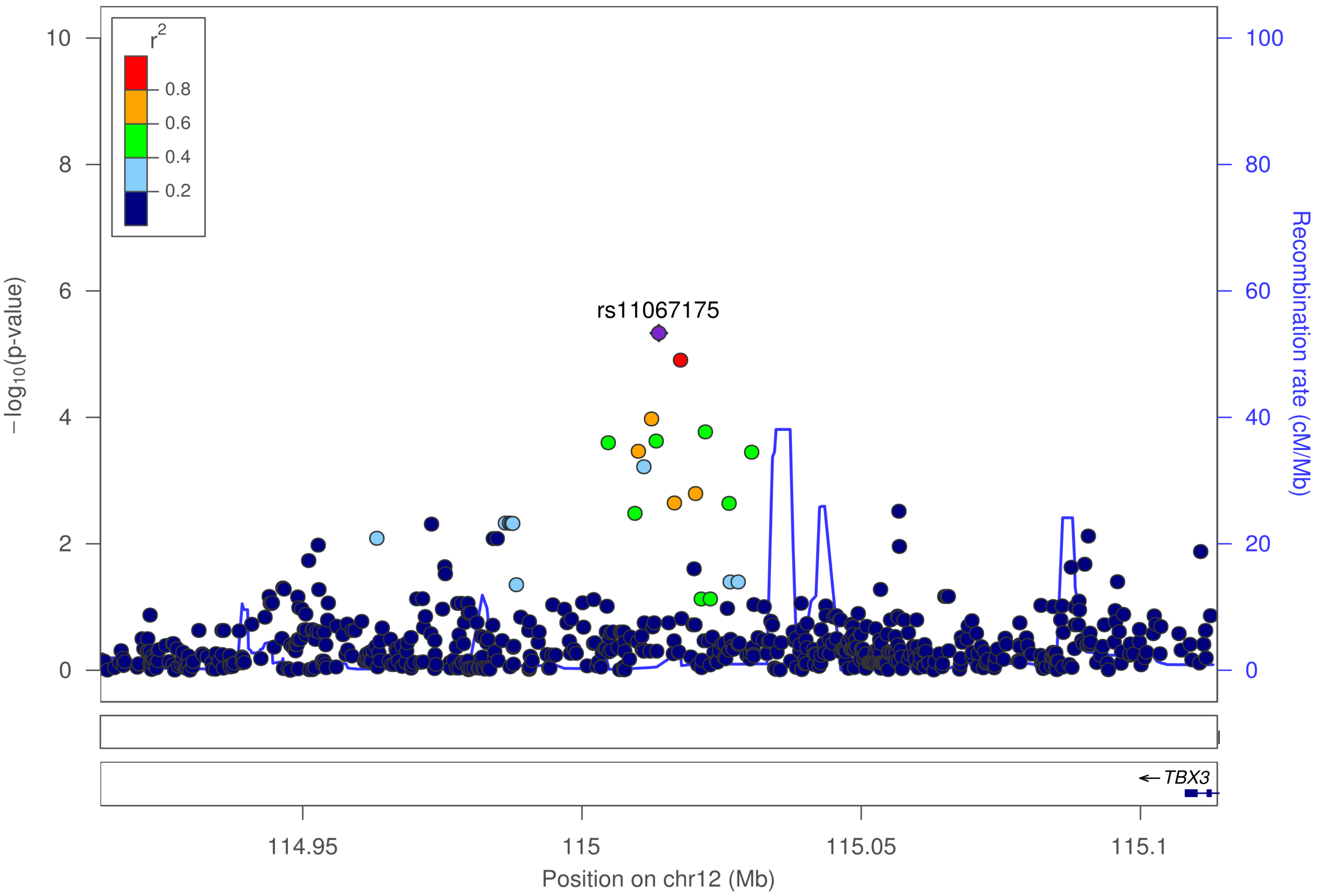
r2
0.8
0.6
0.4
0.2
10
8
6
4
2
0
−log10(p-value)
rs11067175
100
80
60
40
20
0
Recombination rate (cM/Mb)
← TBX3
114.95
115
115.05
115.1
Position on chr12 (Mb)

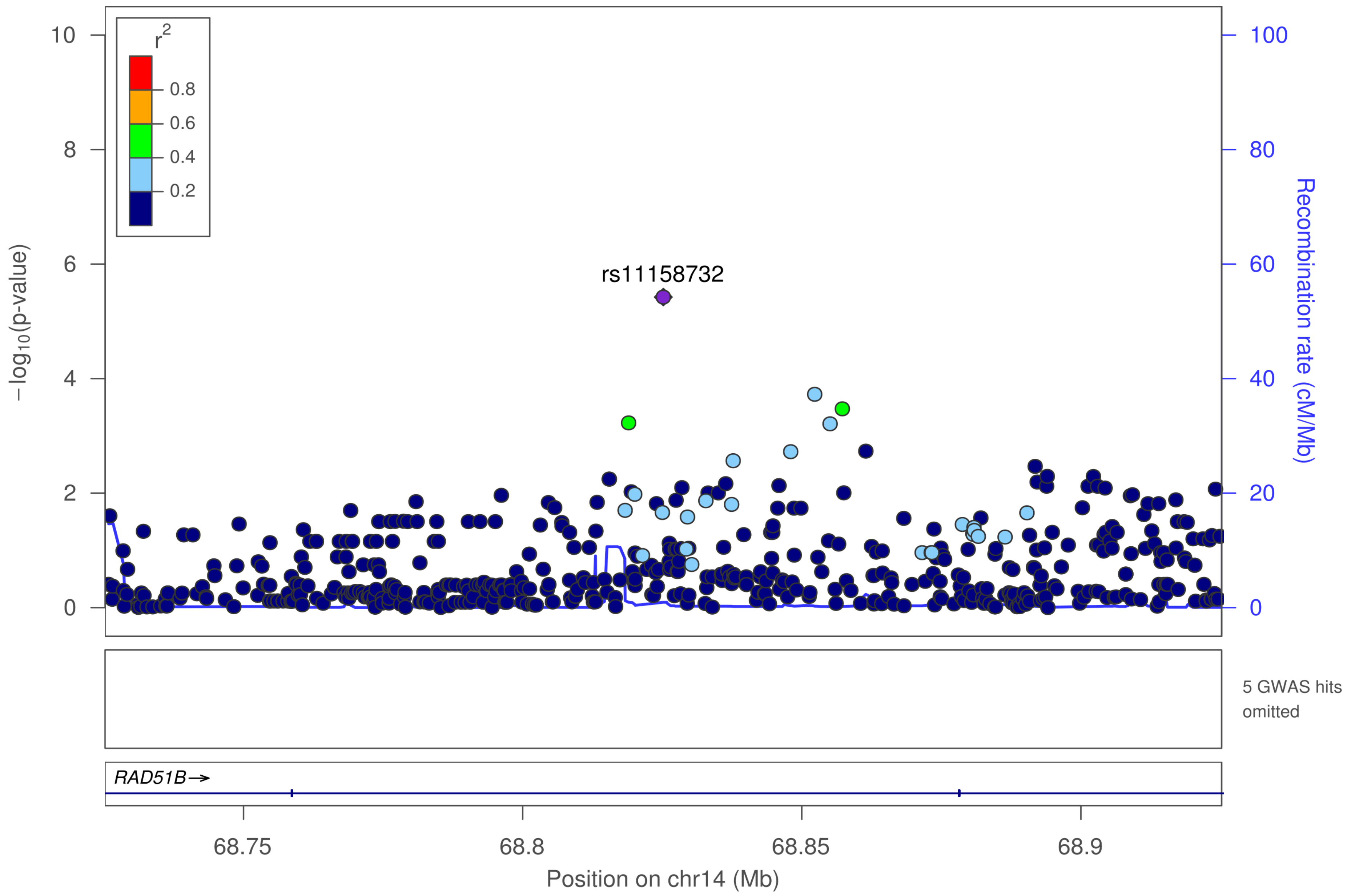
r2
0.8
0.6
0.4
0.2
10
8
6
4
2
0
−log10(p-value)
rs11158732
100
80
60
40
20
0
Recombination rate (cM/Mb)
5 GWAS hits omitted
RAD51B→
68.75
68.8
68.85
68.9
Position on chr14 (Mb)

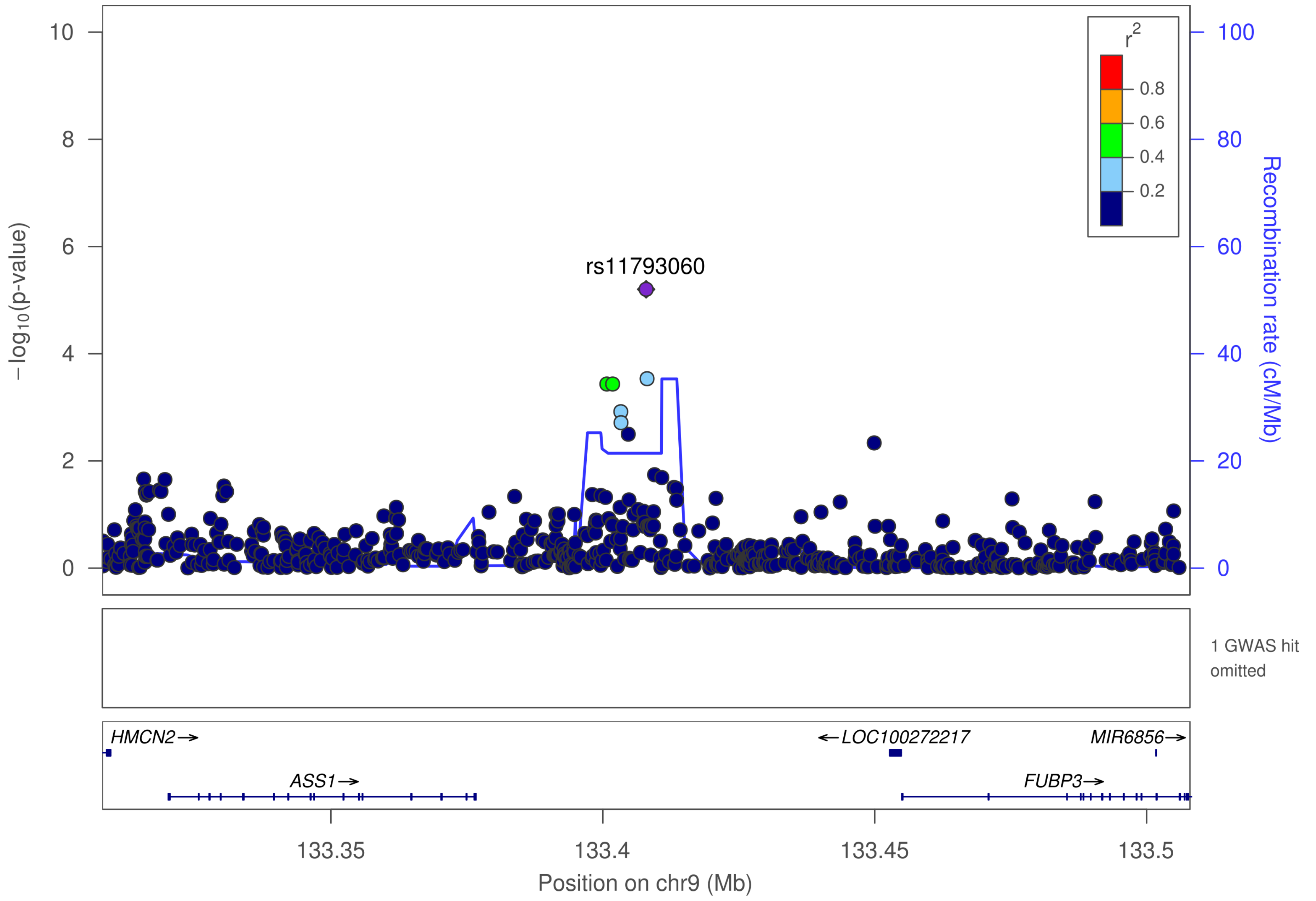

rs11793060
r2
0.8
0.6
0.4
0.2
10
8
6
4
2
0
100
80
60
40
20
0
−log10(p-value)
Recombination rate (cM/Mb)
1 GWAS hit omitted
HMCN2→
ASS1→
←LOC100272217
MIR6856→
FUBP3→
133.35
133.4
133.45
133.5
Position on chr9 (Mb)

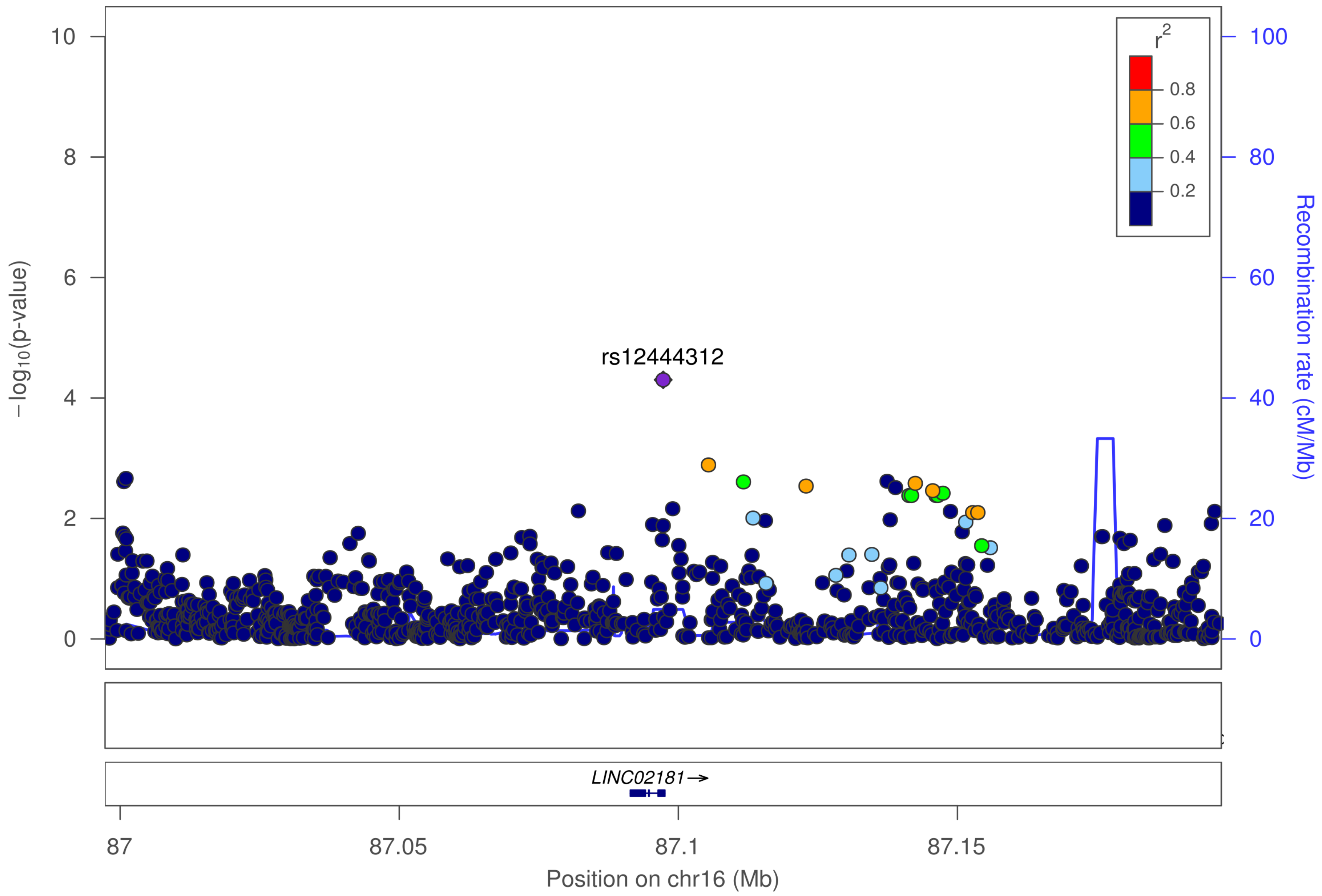

rs12444312
r2
0.8
0.6
0.4
0.2
−log10(p-value)
Recombination rate (cM/Mb)
LINC02181→
87
87.05
87.1
87.15
Position on chr16 (Mb)

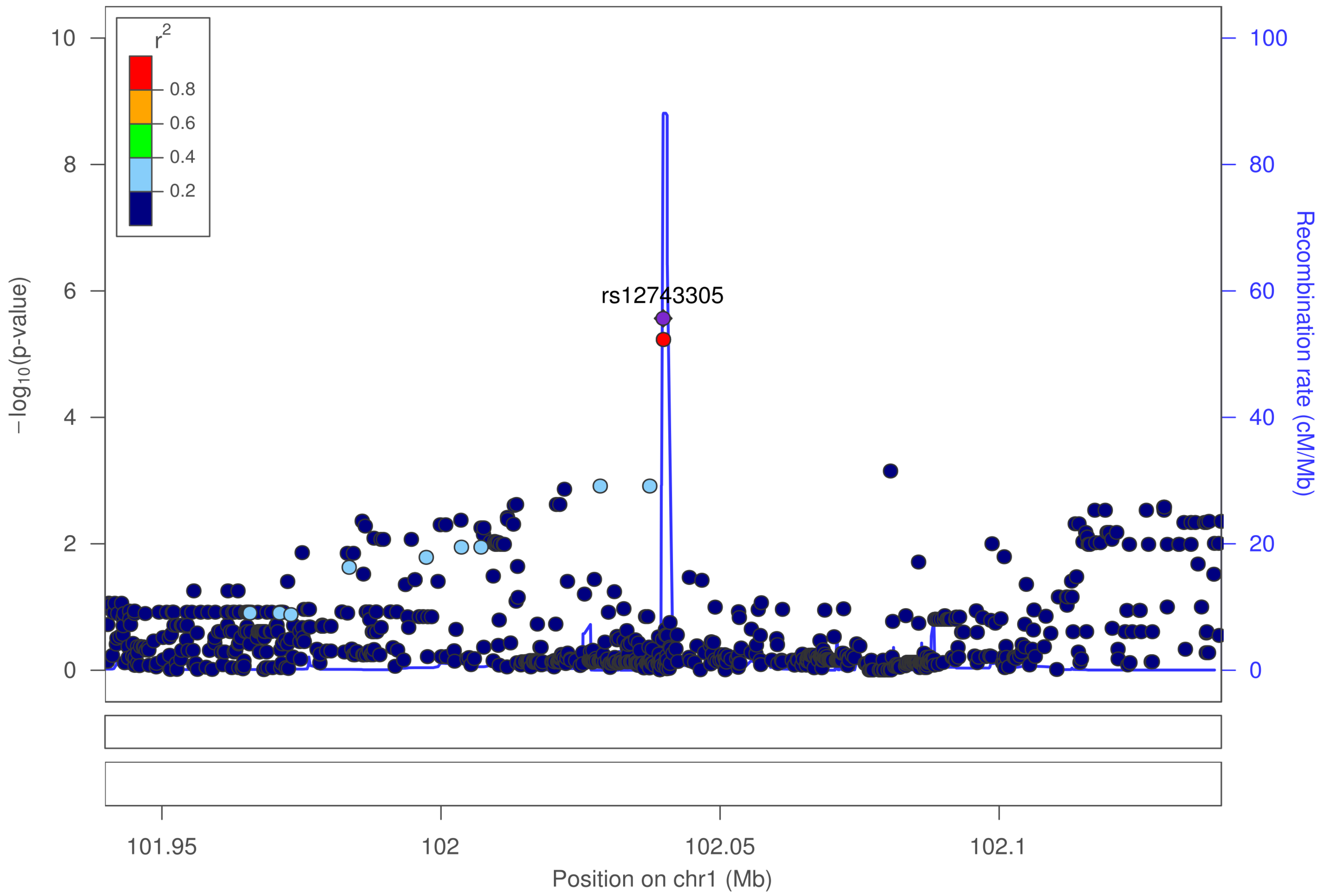

r2
0.8
0.6
0.4
0.2
rs12743305
10
8
6
4
2
0
−log10(p-value)
100
80
60
40
20
0
Recombination rate (cM/Mb)
101.95
102
102.05
102.1
Position on chr1 (Mb)

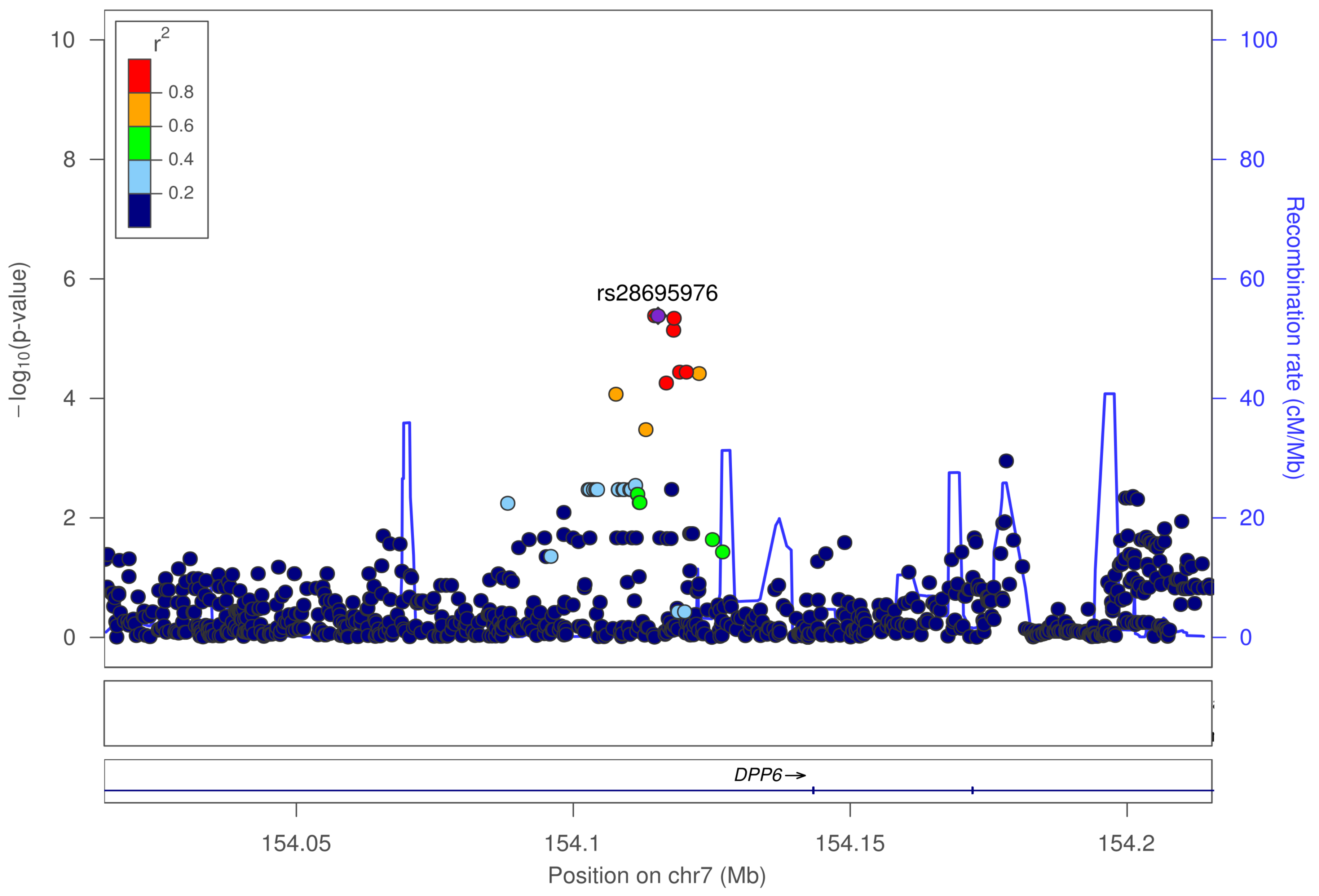

r²
0.8
0.6
0.4
0.2
rs28695976
−log10(p-value)
Recombination rate (cM/Mb)
0
2
4
6
8
10
0
20
40
60
80
100
DPP6→
154.05
154.1
154.15
154.2
Position on chr7 (Mb)

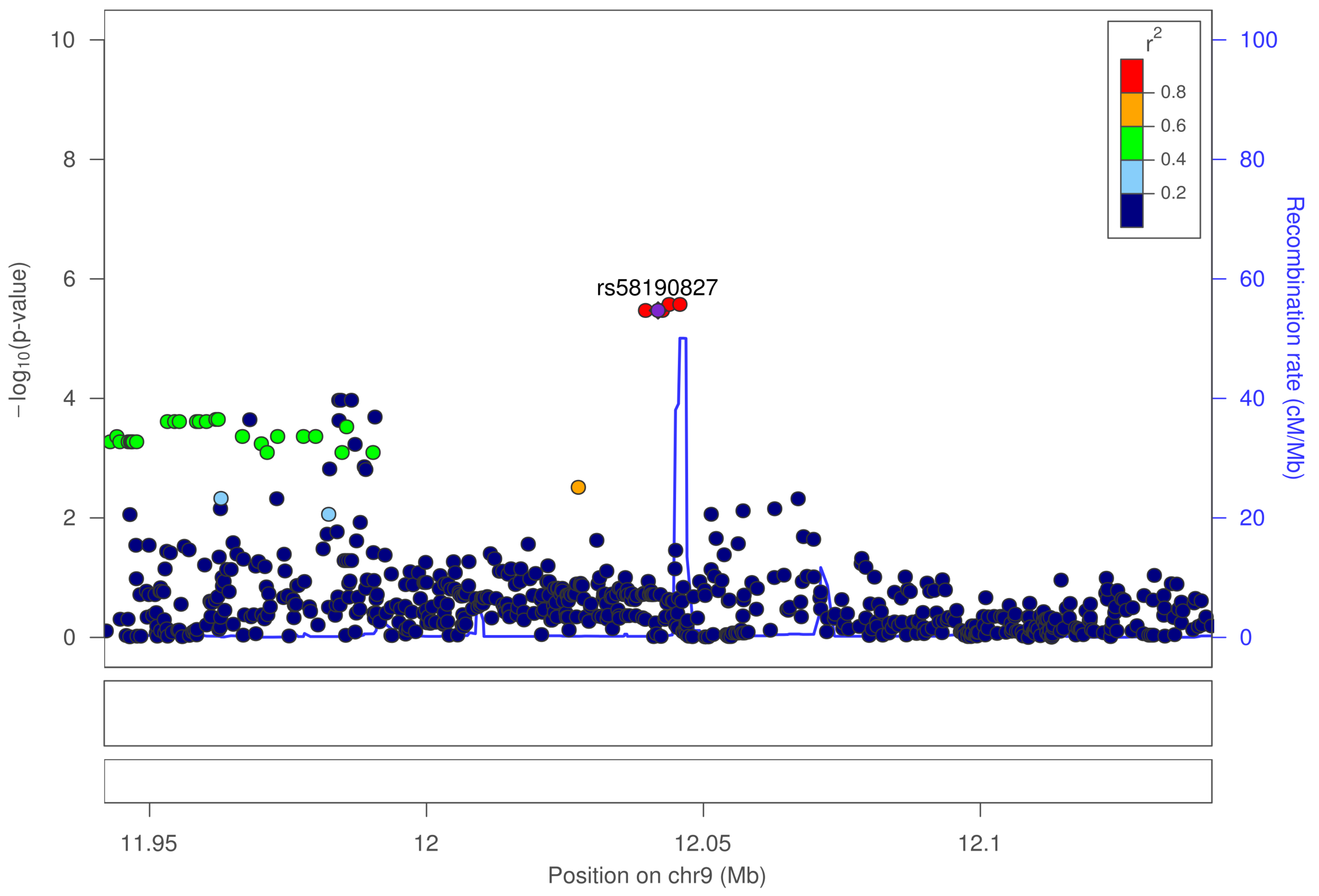

rs58190827
r2
0.8
0.6
0.4
0.2
10
8
6
4
2
0
100
80
60
40
20
0
−log10(p-value)
Recombination rate (cM/Mb)
11.95
12
12.05
12.1
Position on chr9 (Mb)

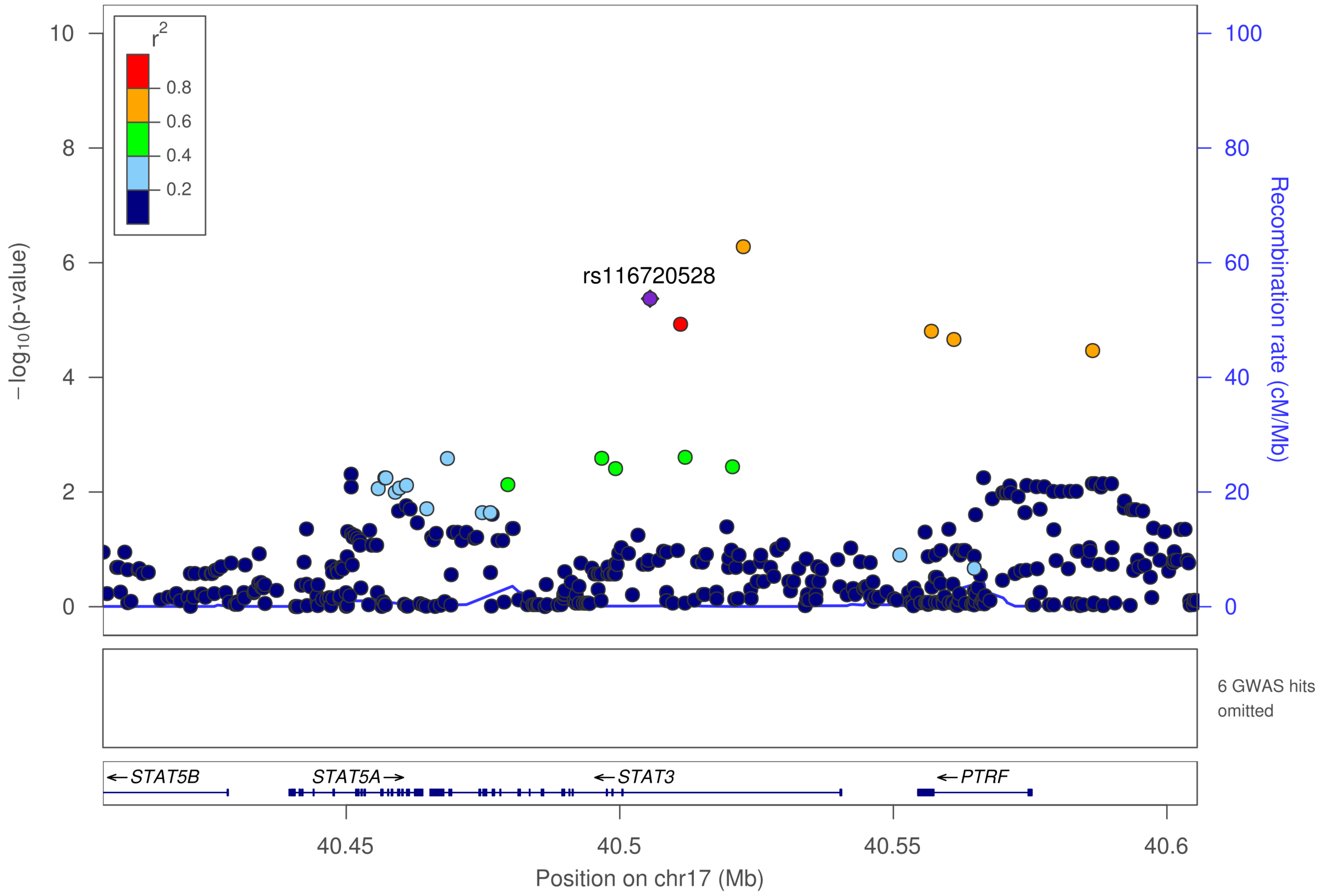

r2
0.8
0.6
0.4
0.2
rs116720528
−log10(p-value)
Recombination rate (cM/Mb)
10
8
6
4
2
0
100
80
60
40
20
0
6 GWAS hits
omitted
←STAT5B
STAT5A→
←STAT3
←PTRF
40.45
40.5
40.55
40.6
Position on chr17 (Mb)

Supplementary Figure 1c:

Regional association plots of selected loci (p-values) in Combined Sample. Distinct genomic risk loci were defined as LD-independent regions (r 2 separated by 100 kb and containing one or more SNPs with a suggestive association (p-values<1E-05). For each locus , the plots shows the –log10 transformed –value of each SNP on the y-axis and base pair positions along the chromosomes on the x-axis. Genes overlapping the locus are displayed below the plot. SNPs are colored by their LD value with the lead SNP in the region, and those LD values has been generated from the two study populations.

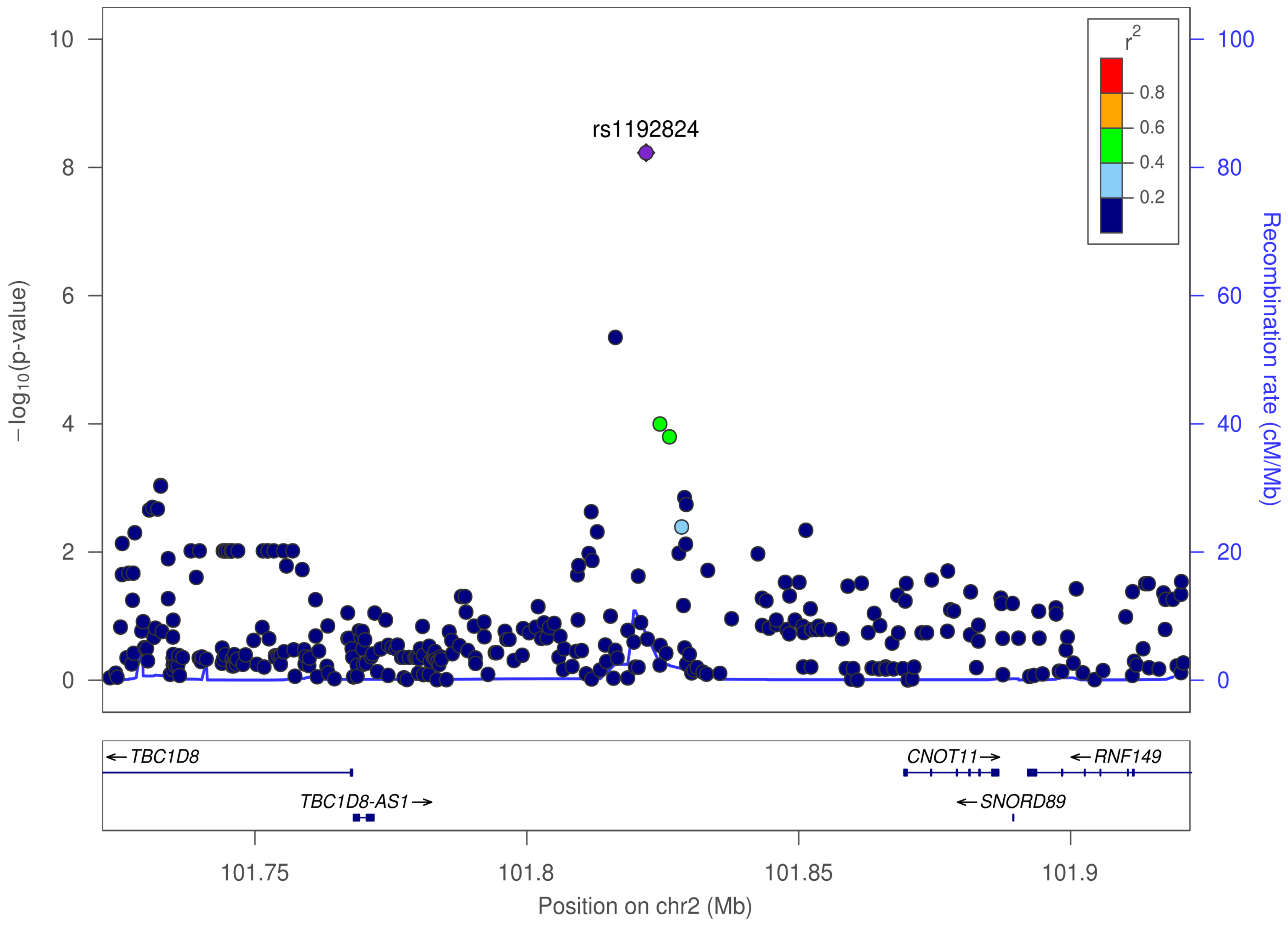

rs1192824
r2
0.8
0.6
0.4
0.2
−log10(p-value)
Recombination rate (cM/Mb)
0
2
4
6
8
10
0
20
40
60
80
100
←TBC1D8
TBC1D8-AS1→
CNOT11→
←RNF149
←SNORD89
101.75
101.8
101.85
101.9
Position on chr2 (Mb)

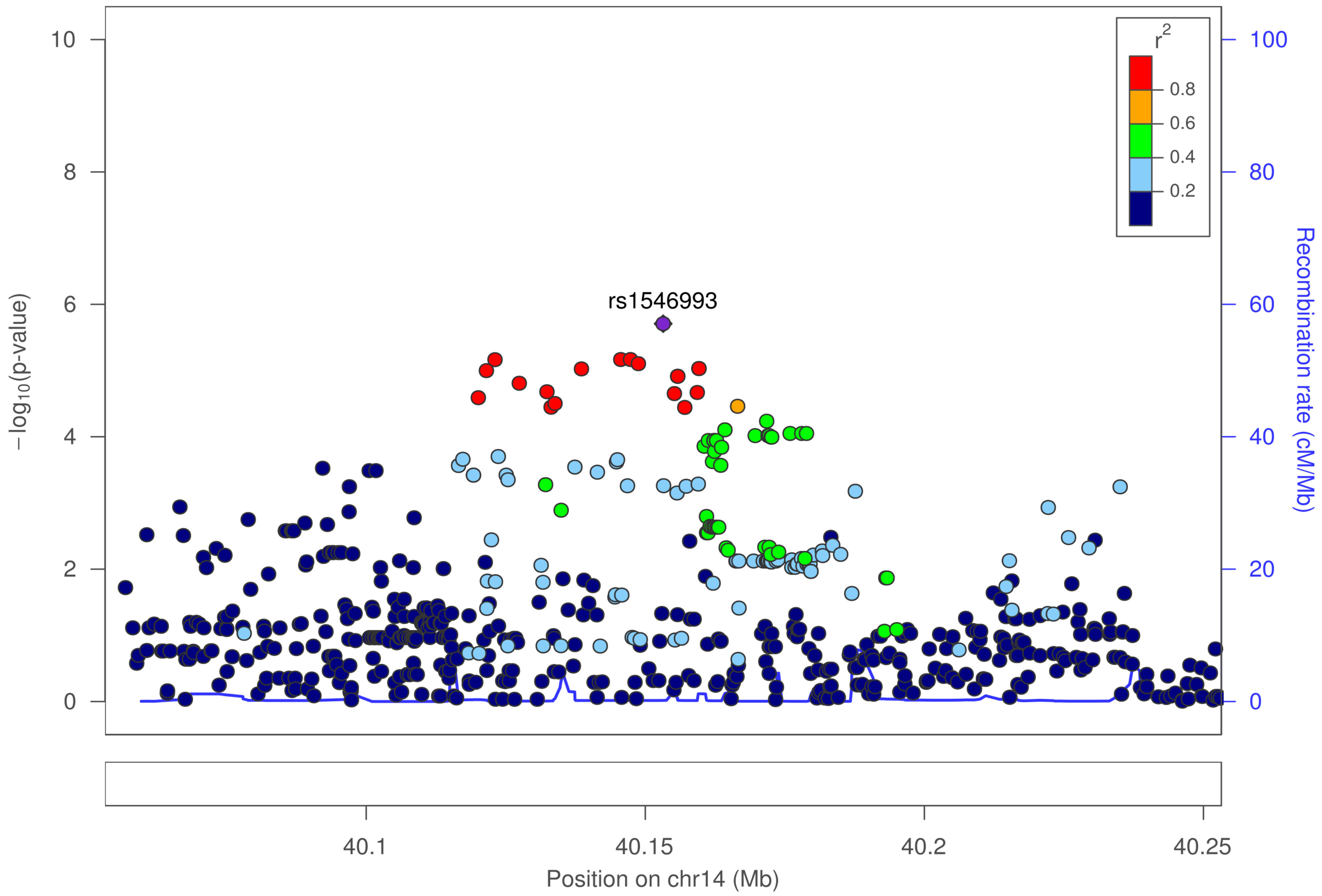

rs1546993
r2
0.8
0.6
0.4
0.2
10
8
6
4
2
0
100
80
60
40
20
0
−log10(p-value)
Recombination rate (cM/Mb)
40.1
40.15
40.2
40.25
Position on chr14 (Mb)

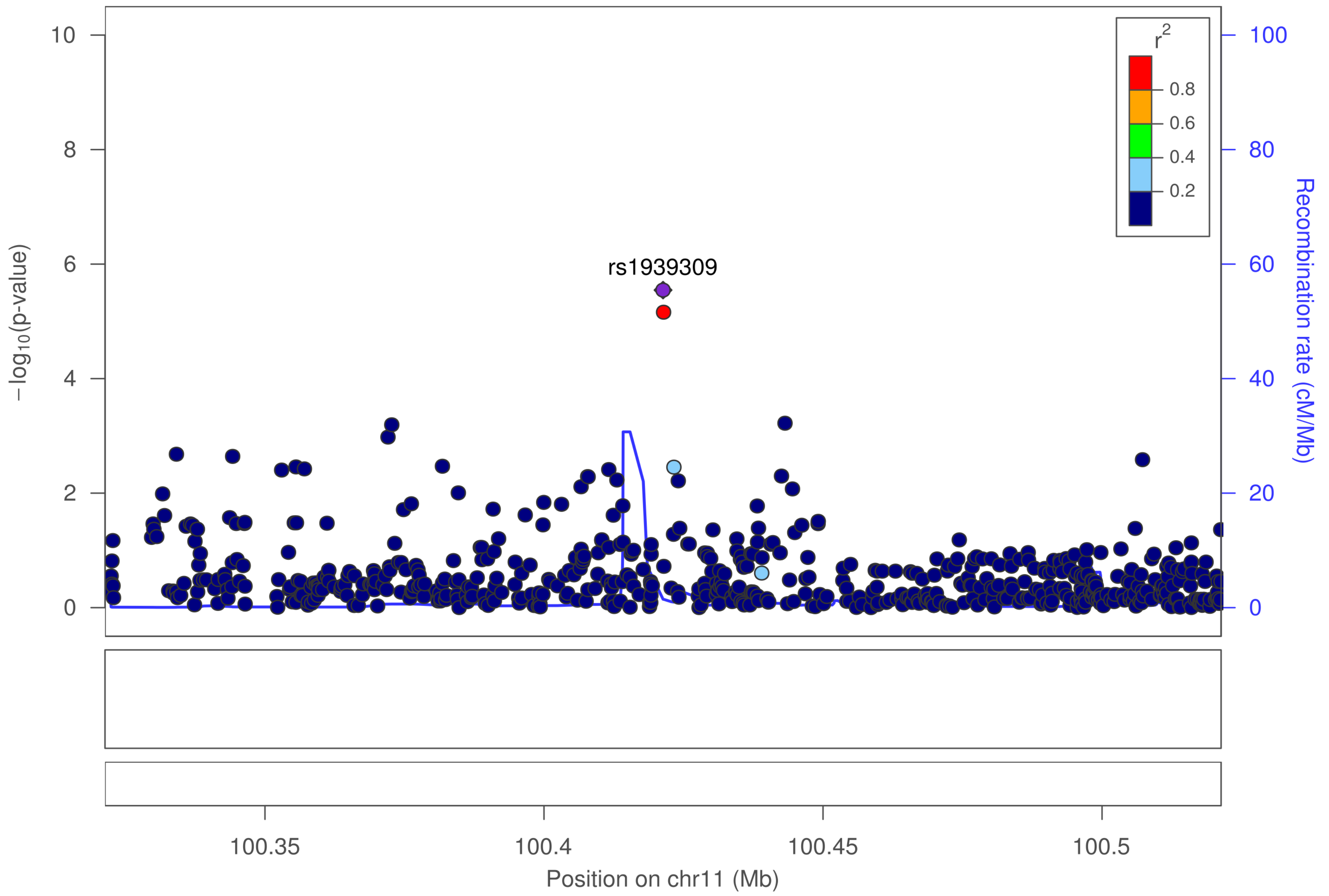

rs1939309
r2
0.8
0.6
0.4
0.2
−log10(p-value)
Recombination rate (cM/Mb)
0
2
4
6
8
10
0
20
40
60
80
100
100.35
100.4
100.45
100.5
Position on chr11 (Mb)

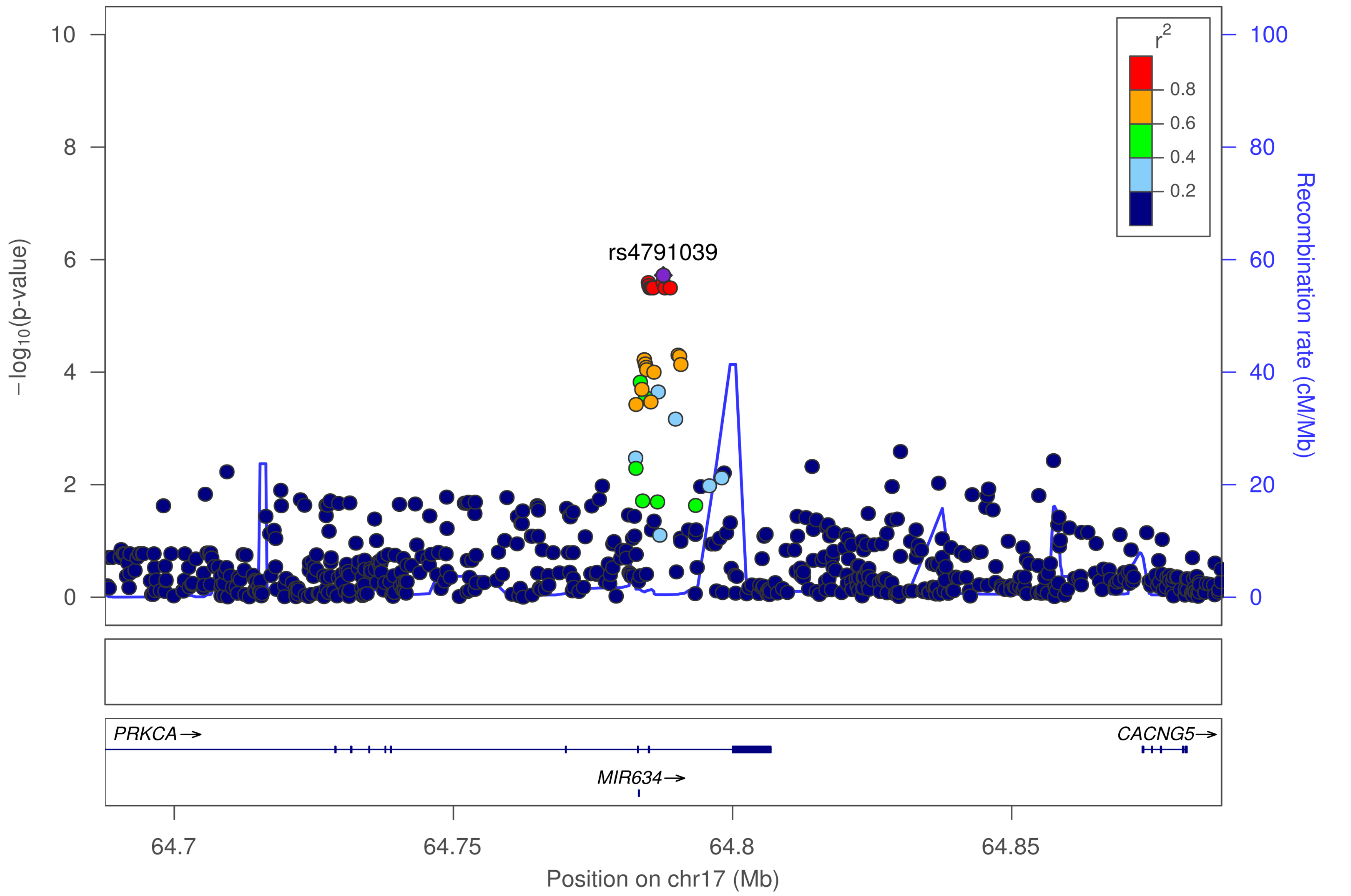

rs4791039
−log10(p-value)
Recombination rate (cM/Mb)
r2
0.8
0.6
0.4
0.2
10
8
6
4
2
0
100
80
60
40
20
0
PRKCA→
CACNG5→
MIR634→
64.7
64.75
64.8
64.85
Position on chr17 (Mb)

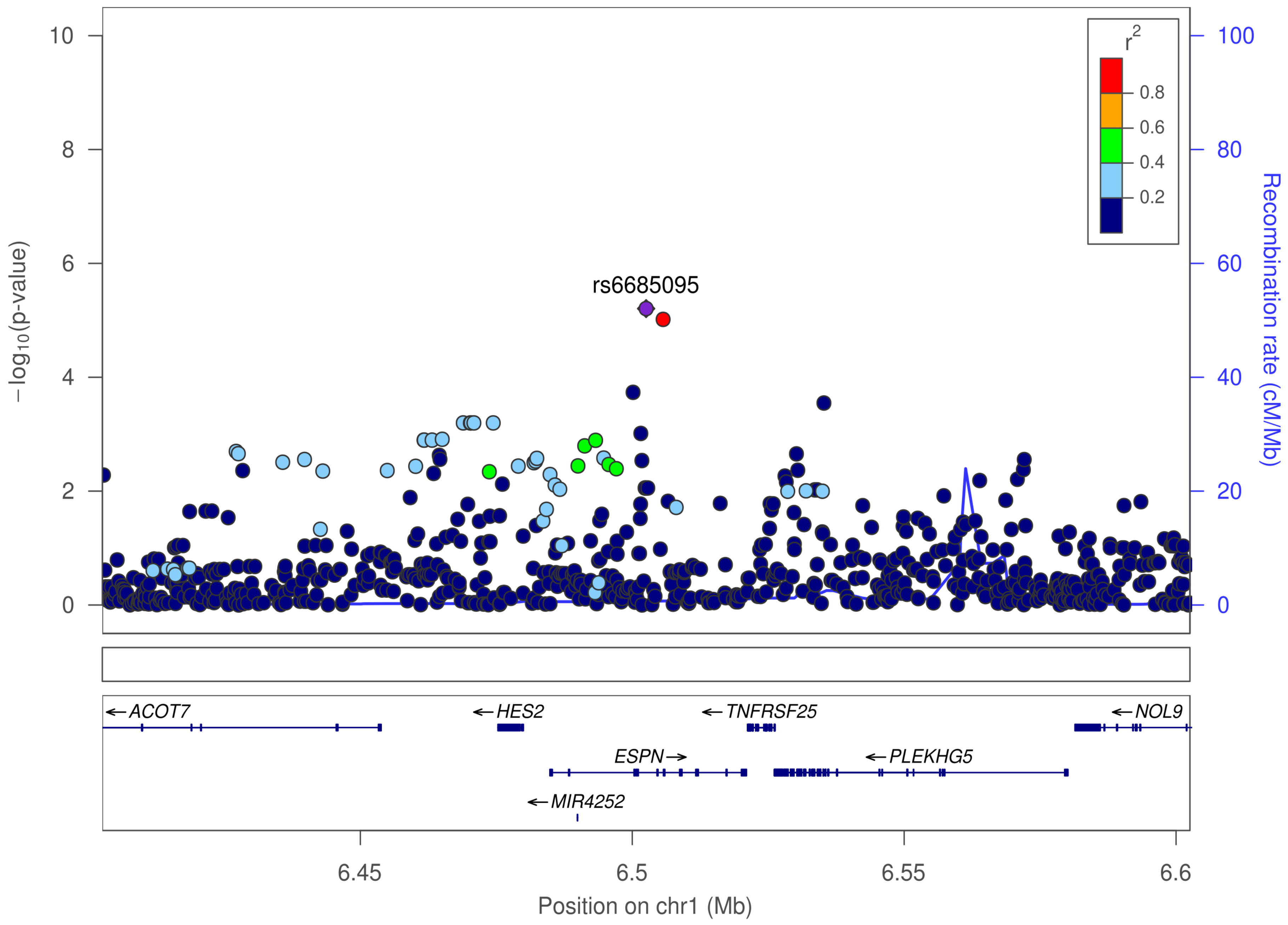

rs6685095
r2
0.8
0.6
0.4
0.2
10
8
6
4
2
0
−log10(p-value)
100
80
60
40
20
0
Recombination rate (cM/Mb)
ACOT7
HES2
TNFRSF25
NOL9
ESPN
PLEKHG5
MIR4252
6.45
6.5
6.55
6.6
Position on chr1 (Mb)

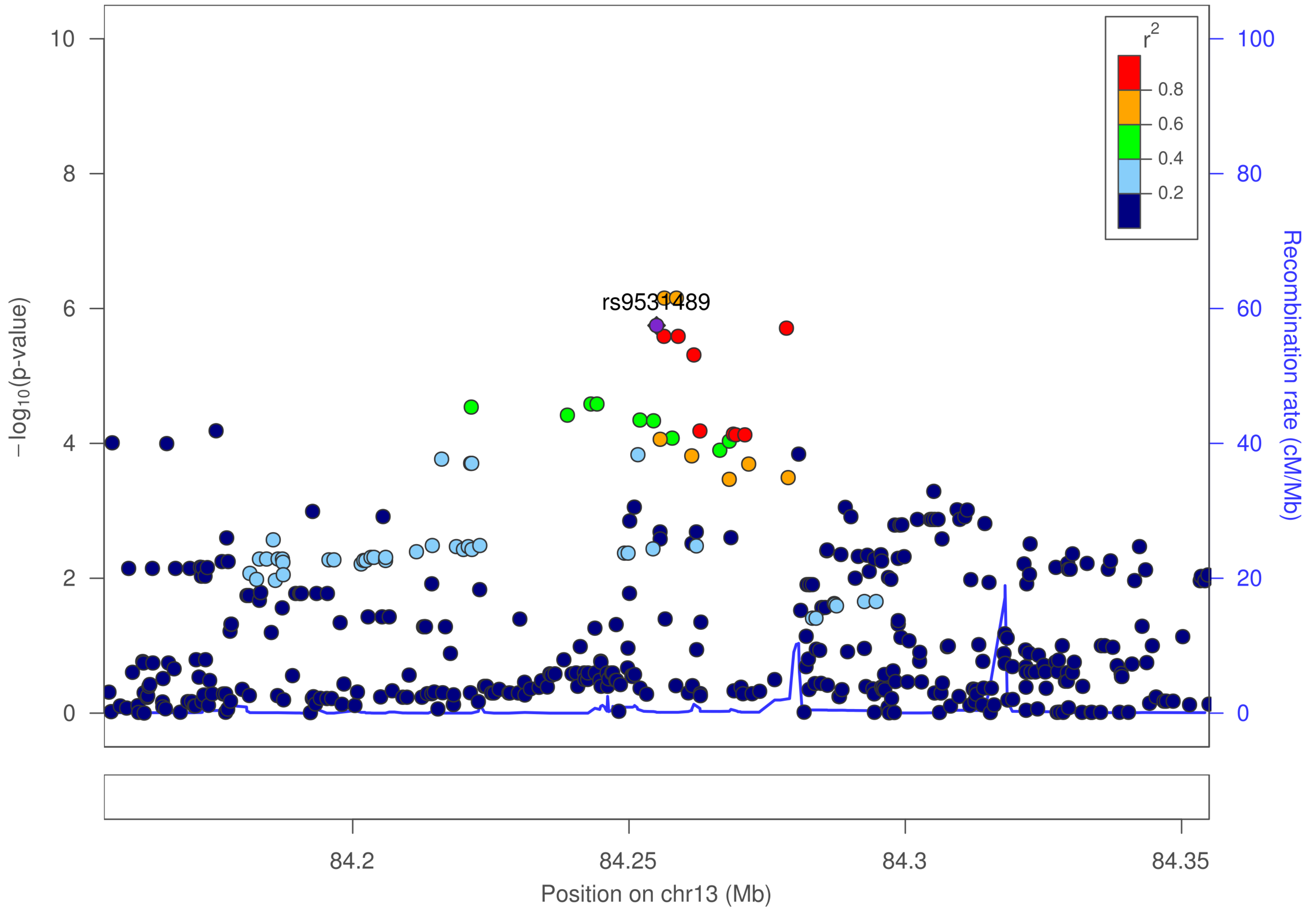

r2
0.8
0.6
0.4
0.2
rs9531489
−log10(p-value)
Recombination rate (cM/Mb)
10
8
6
4
2
0
100
80
60
40
20
0
84.2
84.25
84.3
84.35
Position on chr13 (Mb)

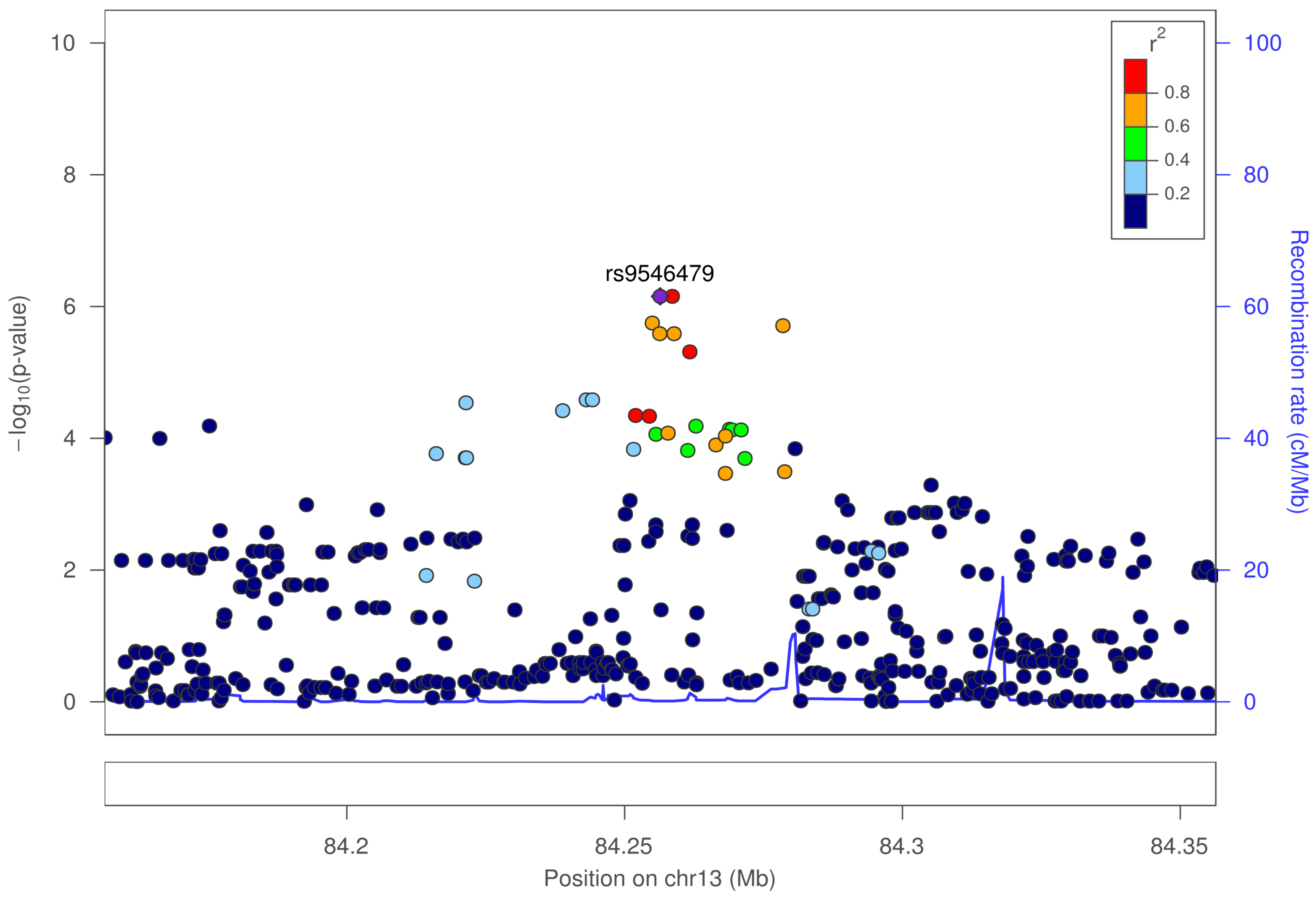
rs9546479
r2
0.8
0.6
0.4
0.2
10
8
6
4
2
0
−log10(p-value)
100
80
60
40
20
0
Recombination rate (cM/Mb)
84.2
84.25
84.3
84.35
Position on chr13 (Mb)

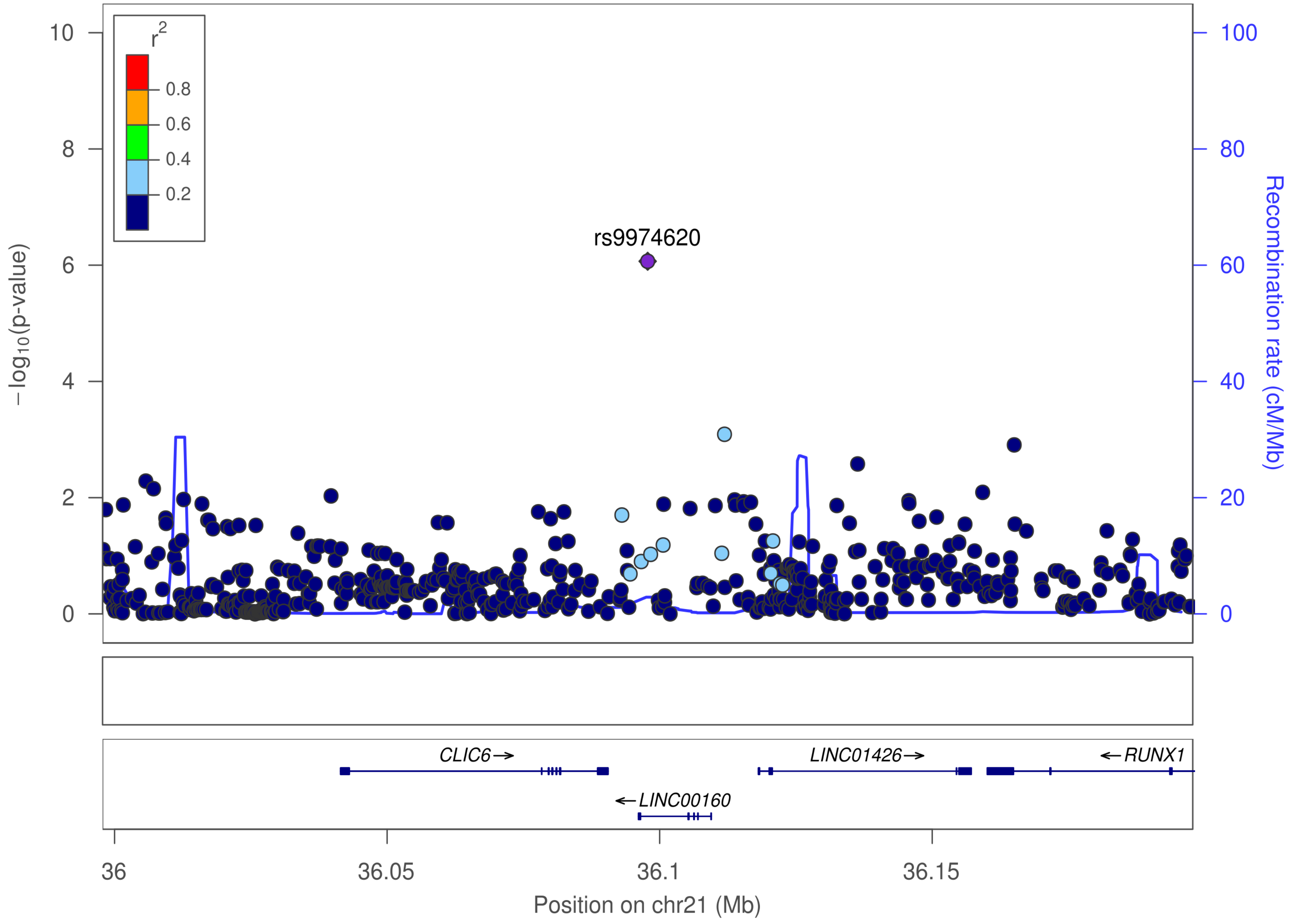
r2
0.8
0.6
0.4
0.2
rs9974620
−log10(p-value)
Recombination rate (cM/Mb)
10
8
6
4
2
0
100
80
60
40
20
0
CLIC6→
LINC01426→
←RUNX1
←LINC00160
36
36.05
36.1
36.15
Position on chr21 (Mb)

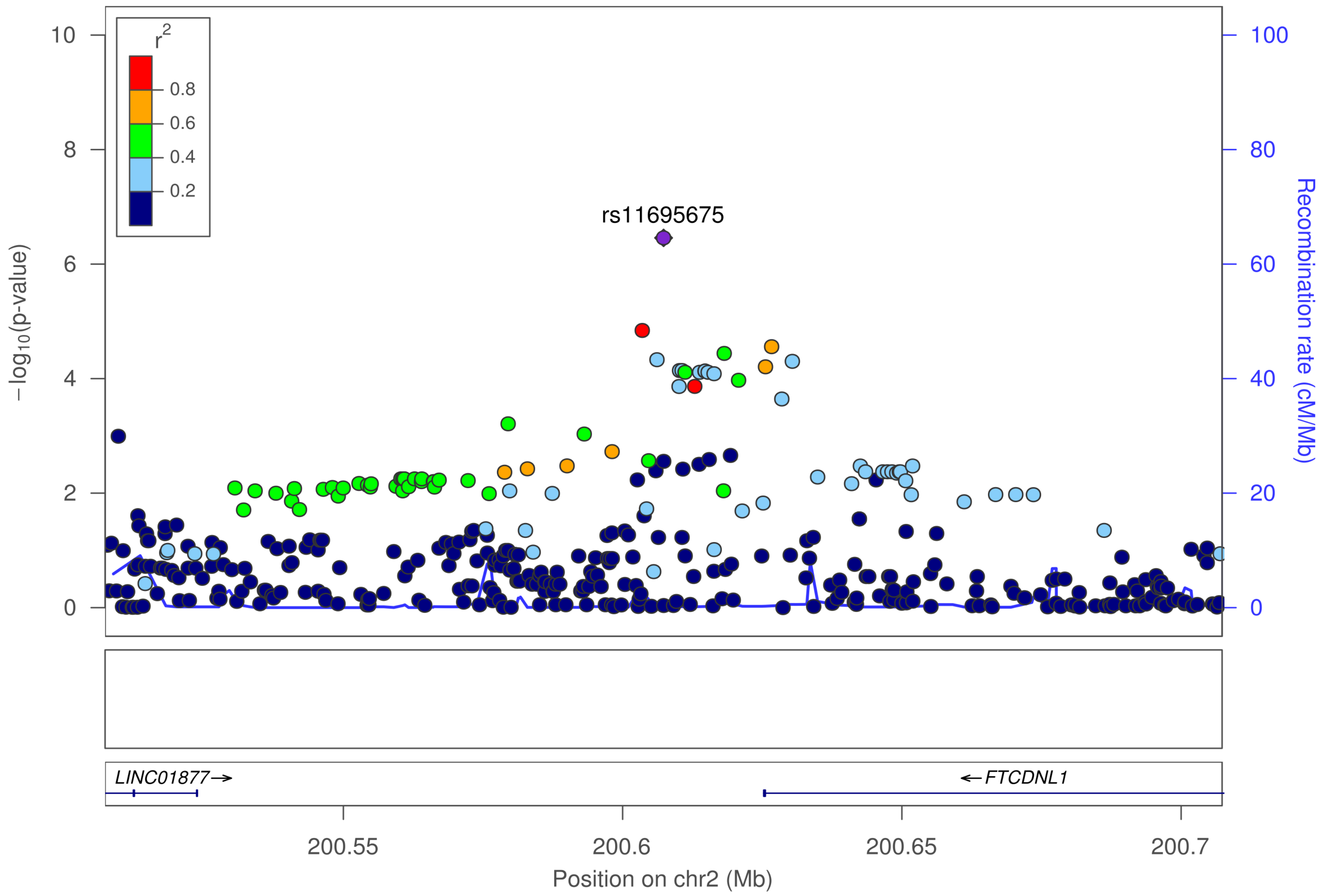
r2
0.8
0.6
0.4
0.2
rs11695675
−log10(p-value)
Recombination rate (cM/Mb)
10
8
6
4
2
0
100
80
60
40
20
0
LINC01877→
←FTCDNL1
200.55
200.6
200.65
200.7
Position on chr2 (Mb)

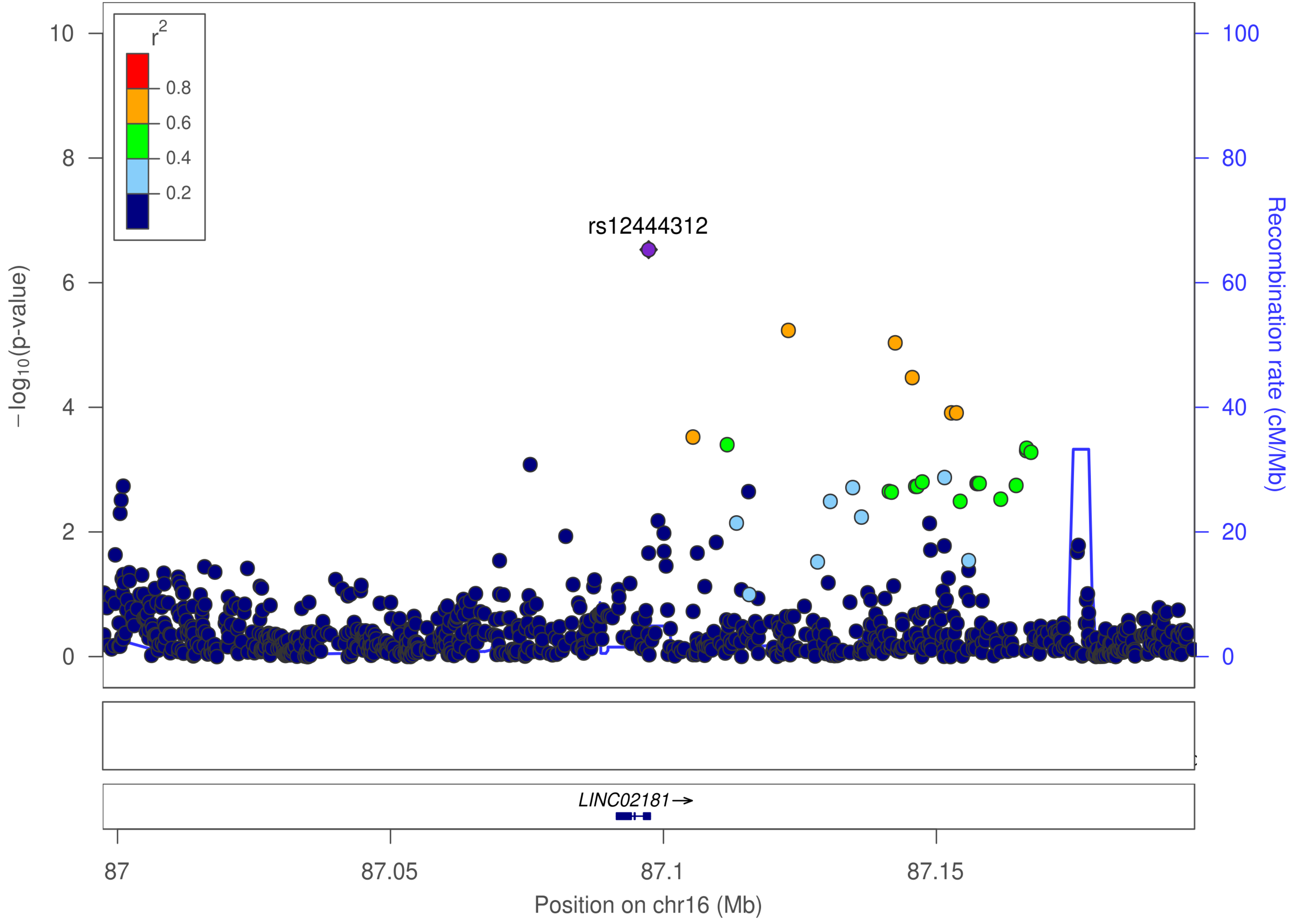
rs12444312
r2
0.8
0.6
0.4
0.2
10
8
6
4
2
0
−log10(p-value)
100
80
60
40
20
0
Recombination rate (cM/Mb)
LINC02181→
87
87.05
87.1
87.15
Position on chr16 (Mb)

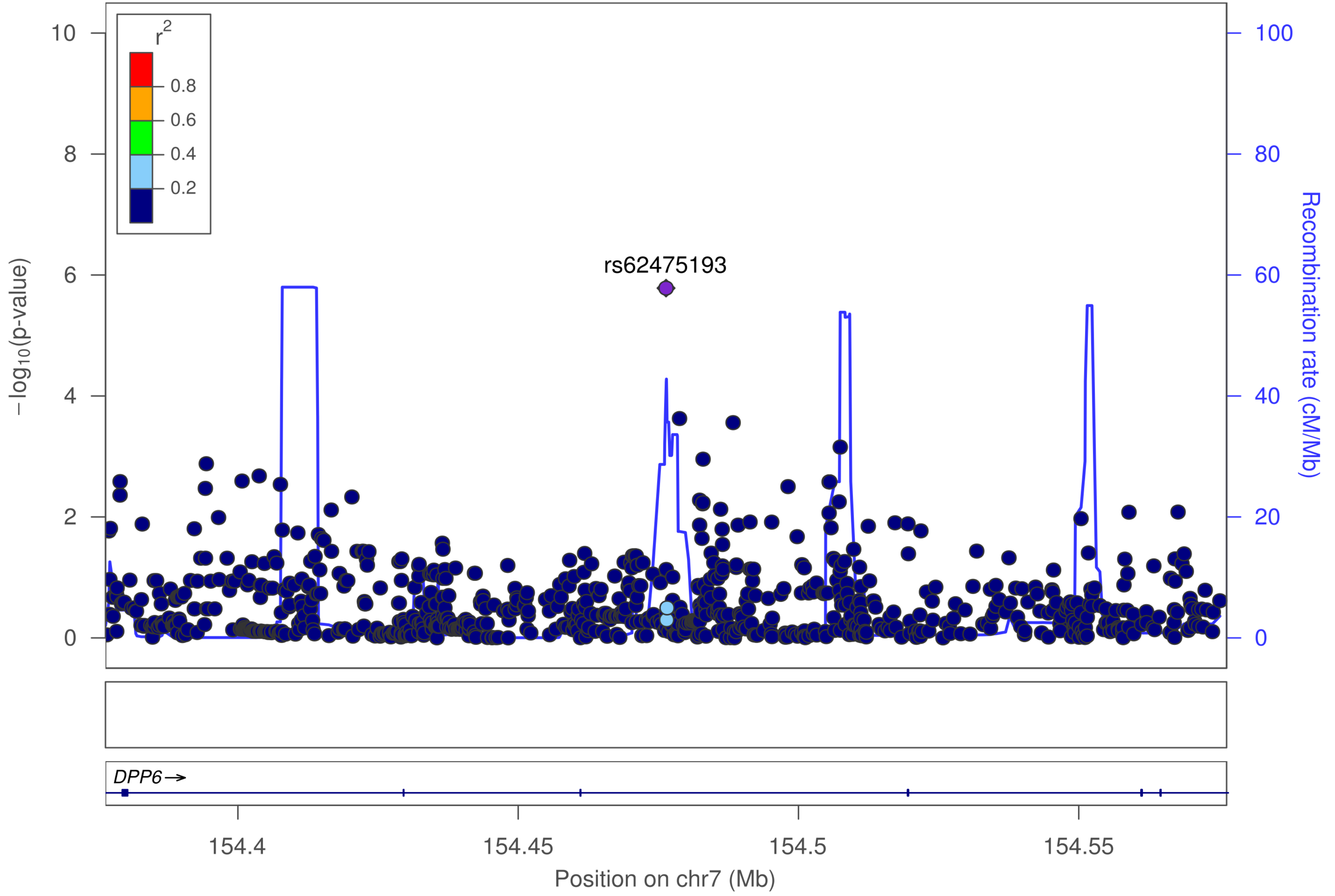

r2
0.8
0.6
0.4
0.2
rs62475193
−log10(p-value)
Recombination rate (cM/Mb)
10
8
6
4
2
0
100
80
60
40
20
0
DPP6→
154.4
154.45
154.5
154.55
Position on chr7 (Mb)

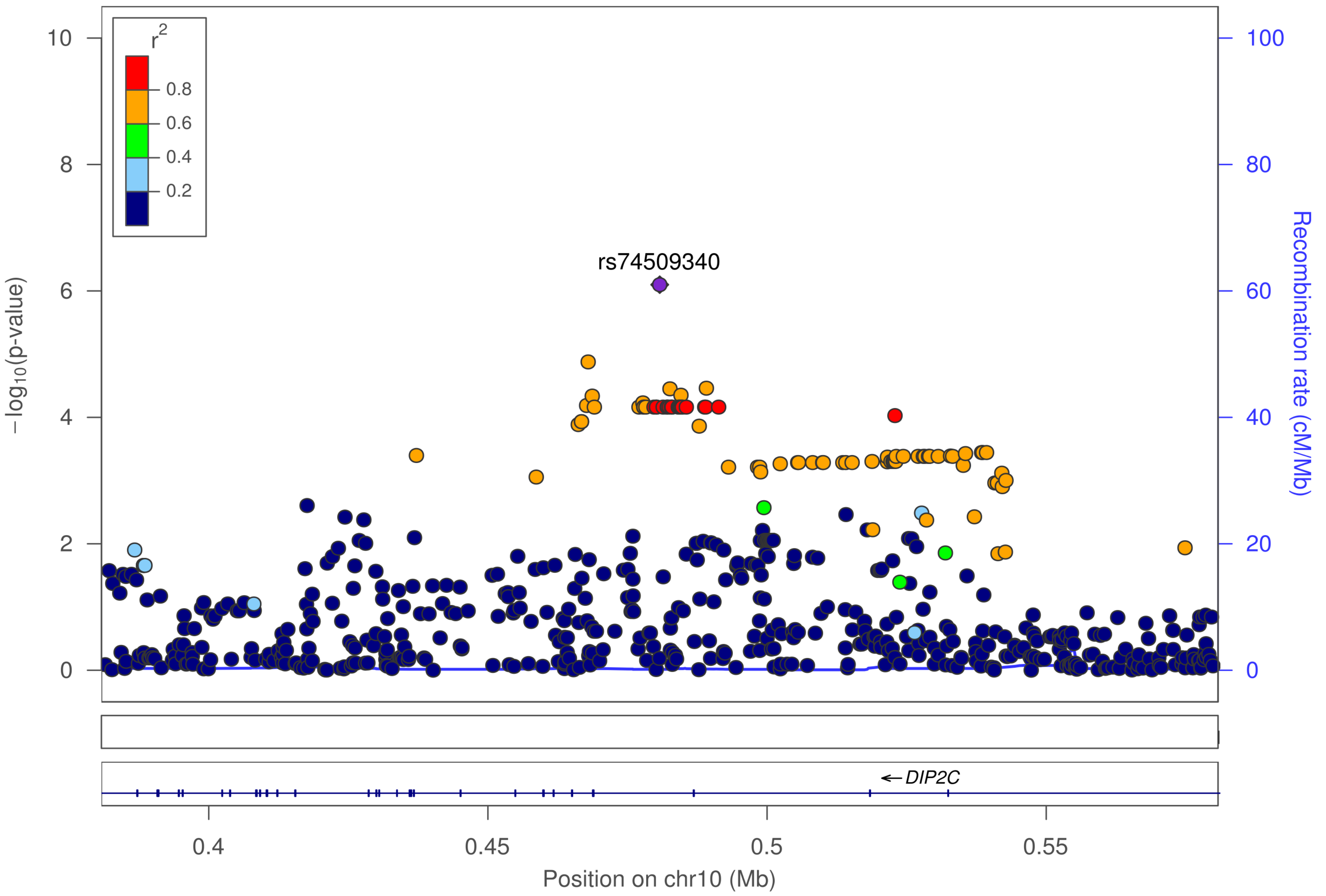
r2
0.8
0.6
0.4
0.2
rs74509340
−log10(p-value)
Recombination rate (cM/Mb)
DIP2C
Position on chr10 (Mb)
0
2
4
6
8
10
0
20
40
60
80
100
0.4
0.45
0.5
0.55

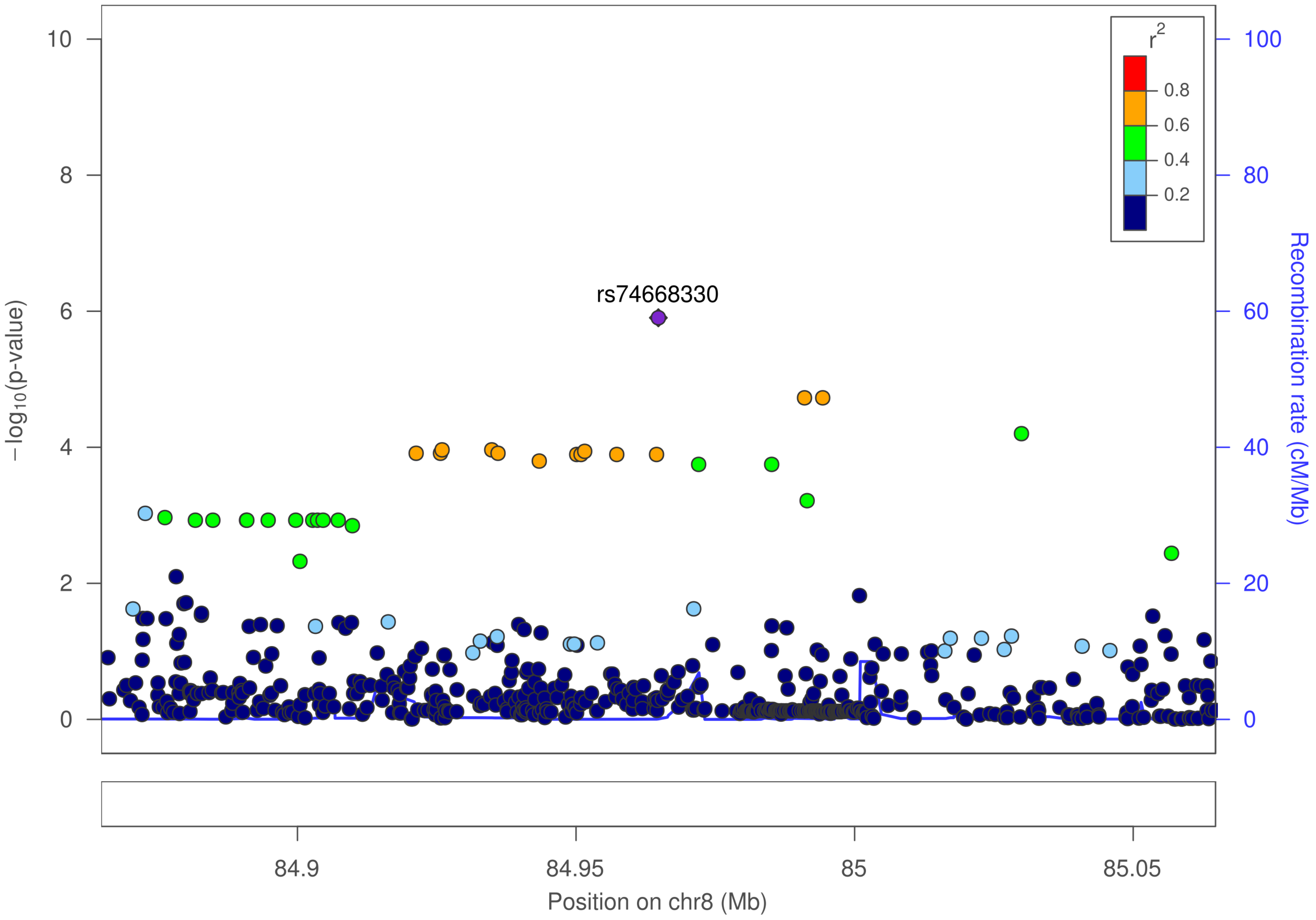

rs74668330
r2
0.8
0.6
0.4
0.2
10
8
6
4
2
0
100
80
60
40
20
0
−log10(p-value)
Recombination rate (cM/Mb)
84.9
84.95
85
85.05
Position on chr8 (Mb)

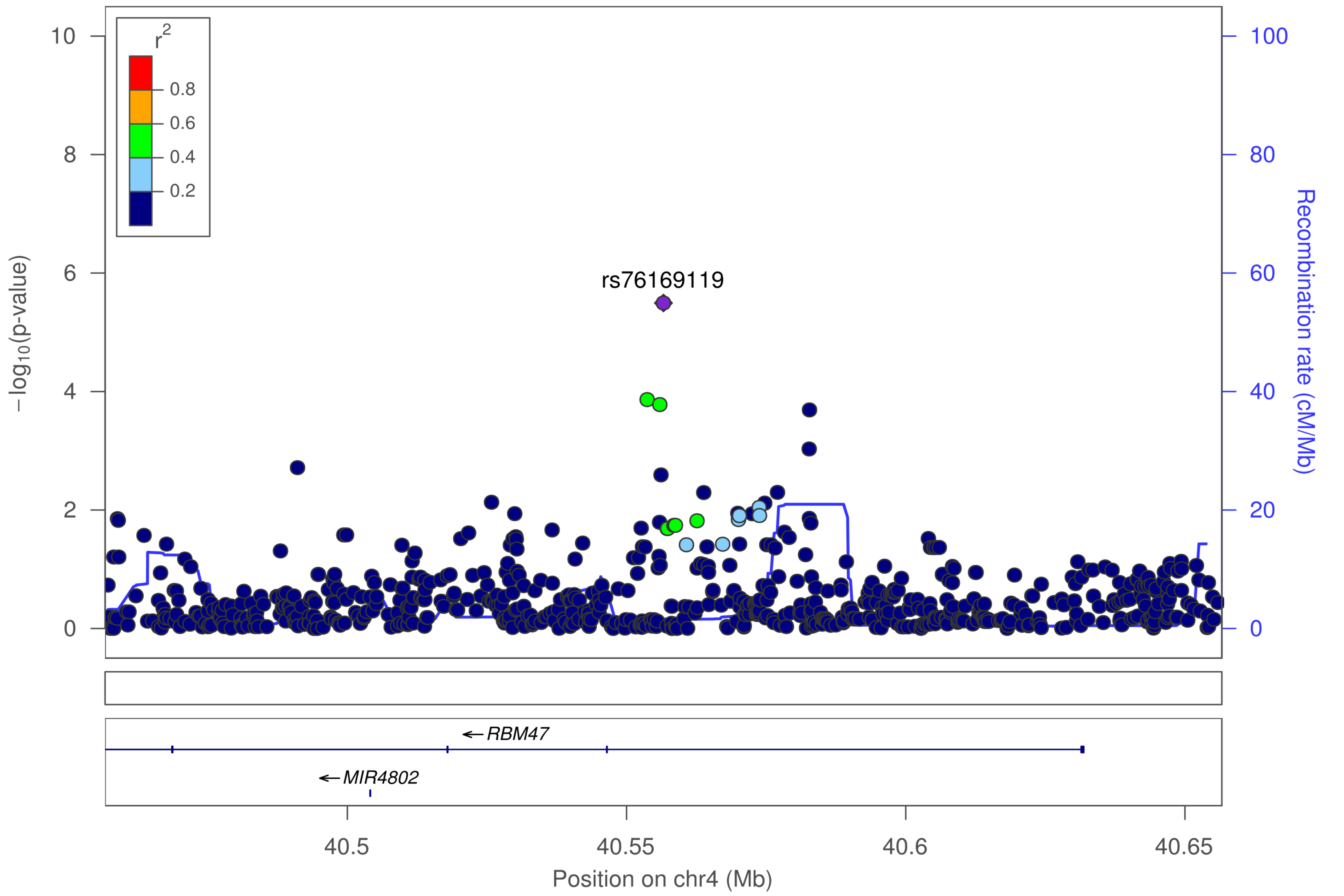
r2
0.8
0.6
0.4
0.2
rs76169119
−log10(p-value)
Recombination rate (cM/Mb)
10
8
6
4
2
0
100
80
60
40
20
0
RBM47
MIR4802
40.5
40.55
40.6
40.65
Position on chr4 (Mb)

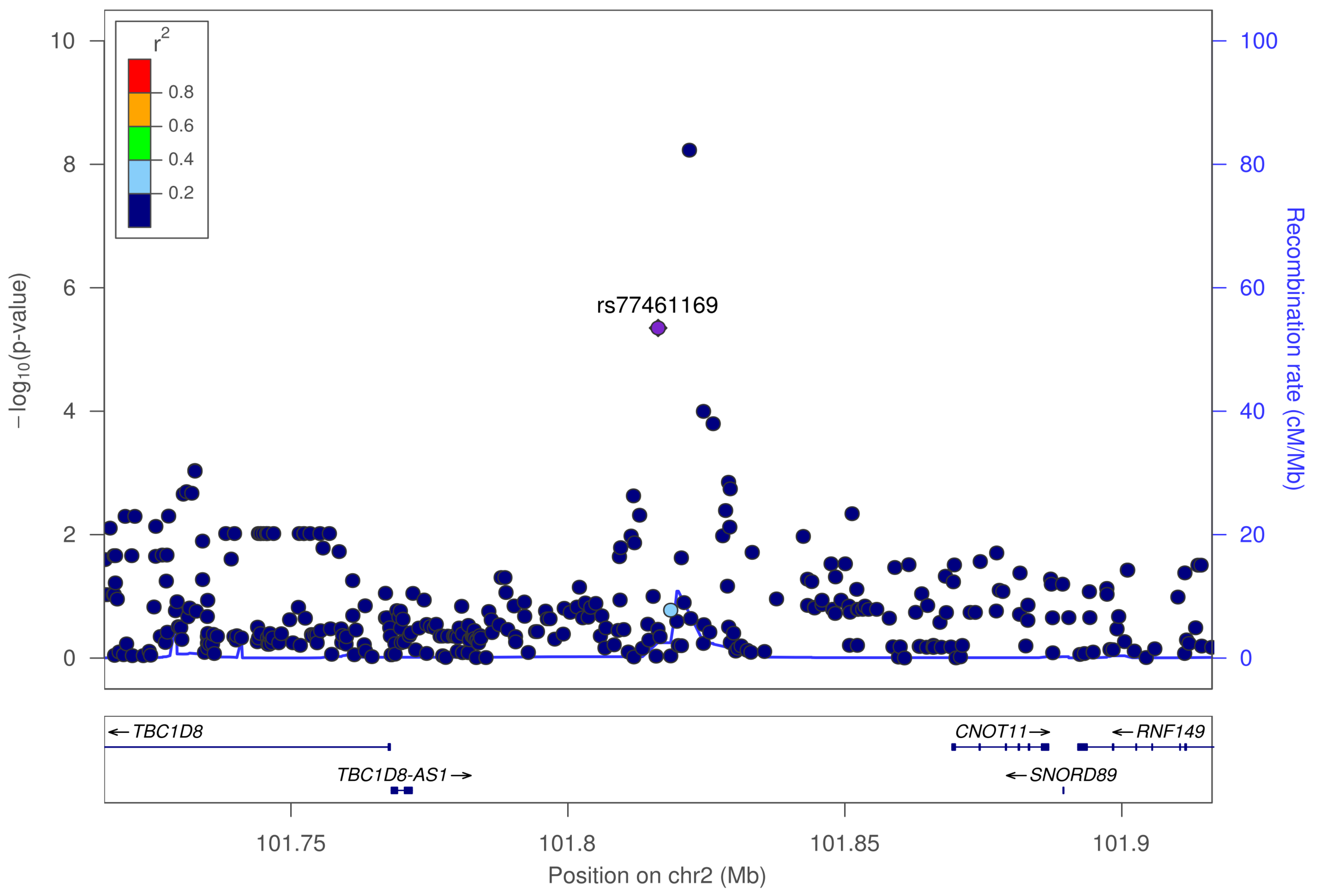

r2
0.8
0.6
0.4
0.2
rs77461169
10
8
6
4
2
0
−log10(p-value)
100
80
60
40
20
0
Recombination rate (cM/Mb)
← TBC1D8
TBC1D8-AS1 →
CNOT11 →
← RNF149
← SNORD89
101.75
101.8
101.85
101.9
Position on chr2 (Mb)

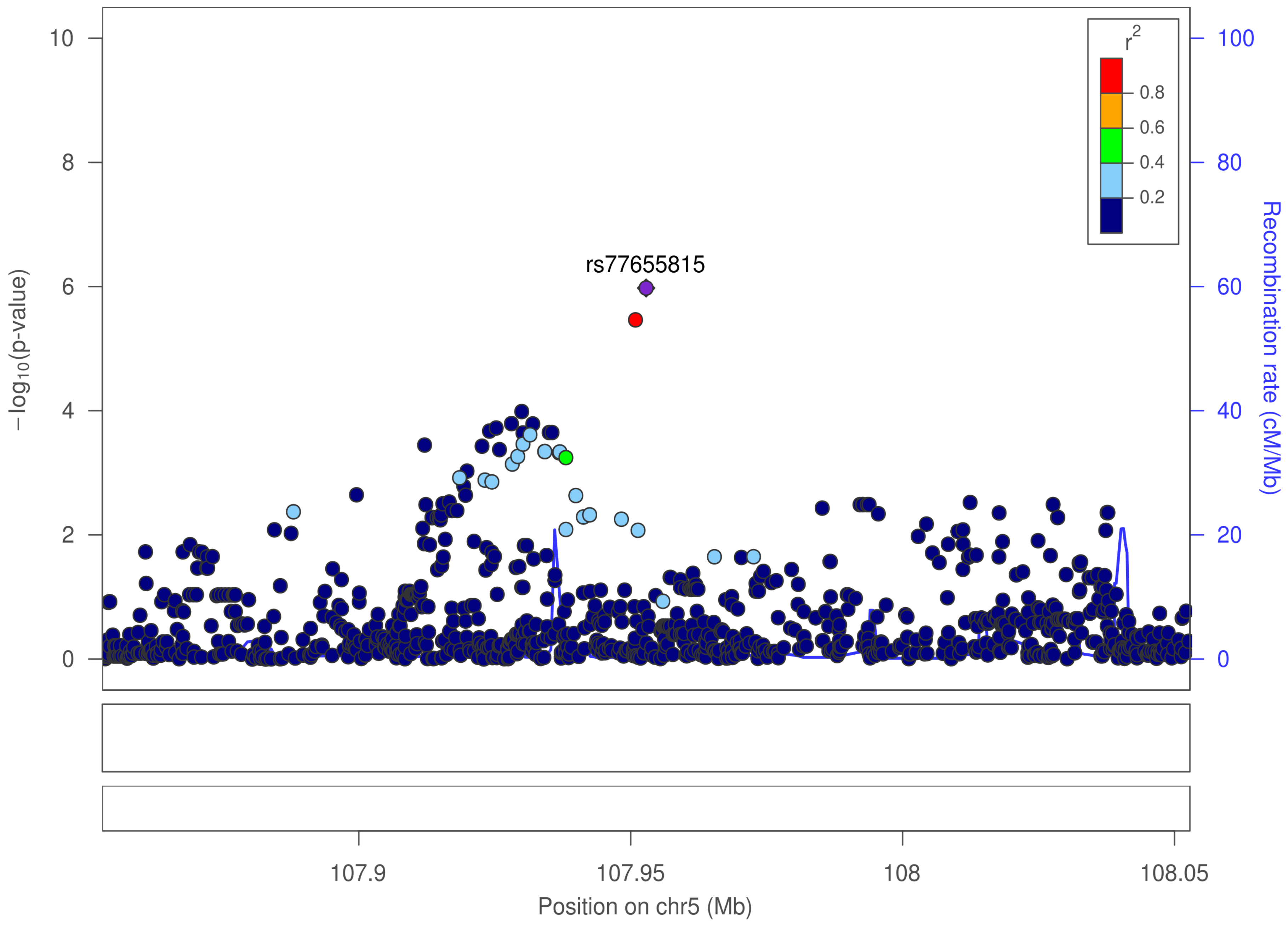
rs77655815
r2
0.8
0.6
0.4
0.2
10
8
6
4
2
0
100
80
60
40
20
0
−log10(p-value)
Recombination rate (cM/Mb)
107.9
107.95
108
108.05
Position on chr5 (Mb)

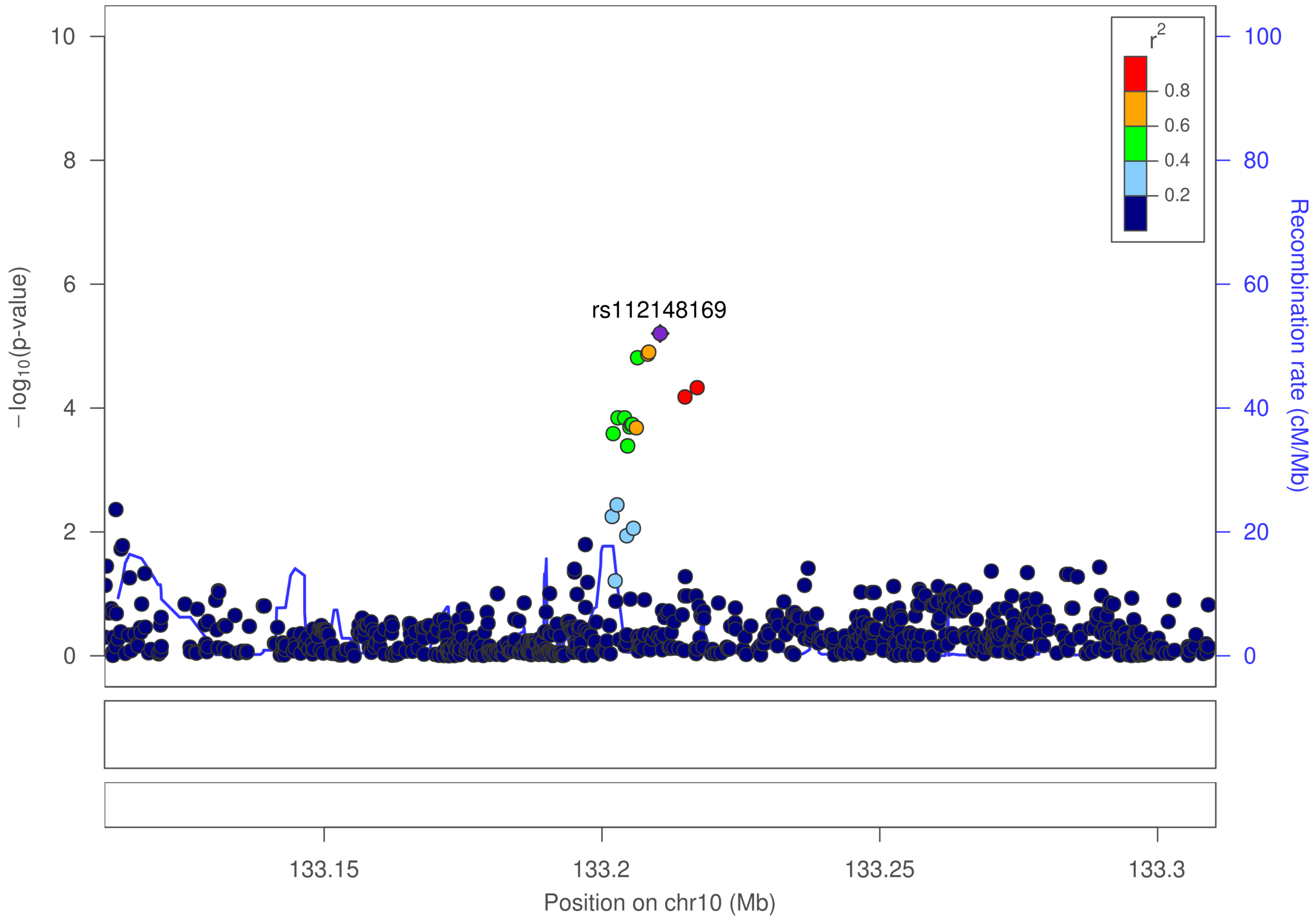

rs112148169
r2
0.8
0.6
0.4
0.2
−log10(p-value)
Recombination rate (cM/Mb)
10
8
6
4
2
0
100
80
60
40
20
0
133.15
133.2
133.25
133.3
Position on chr10 (Mb)

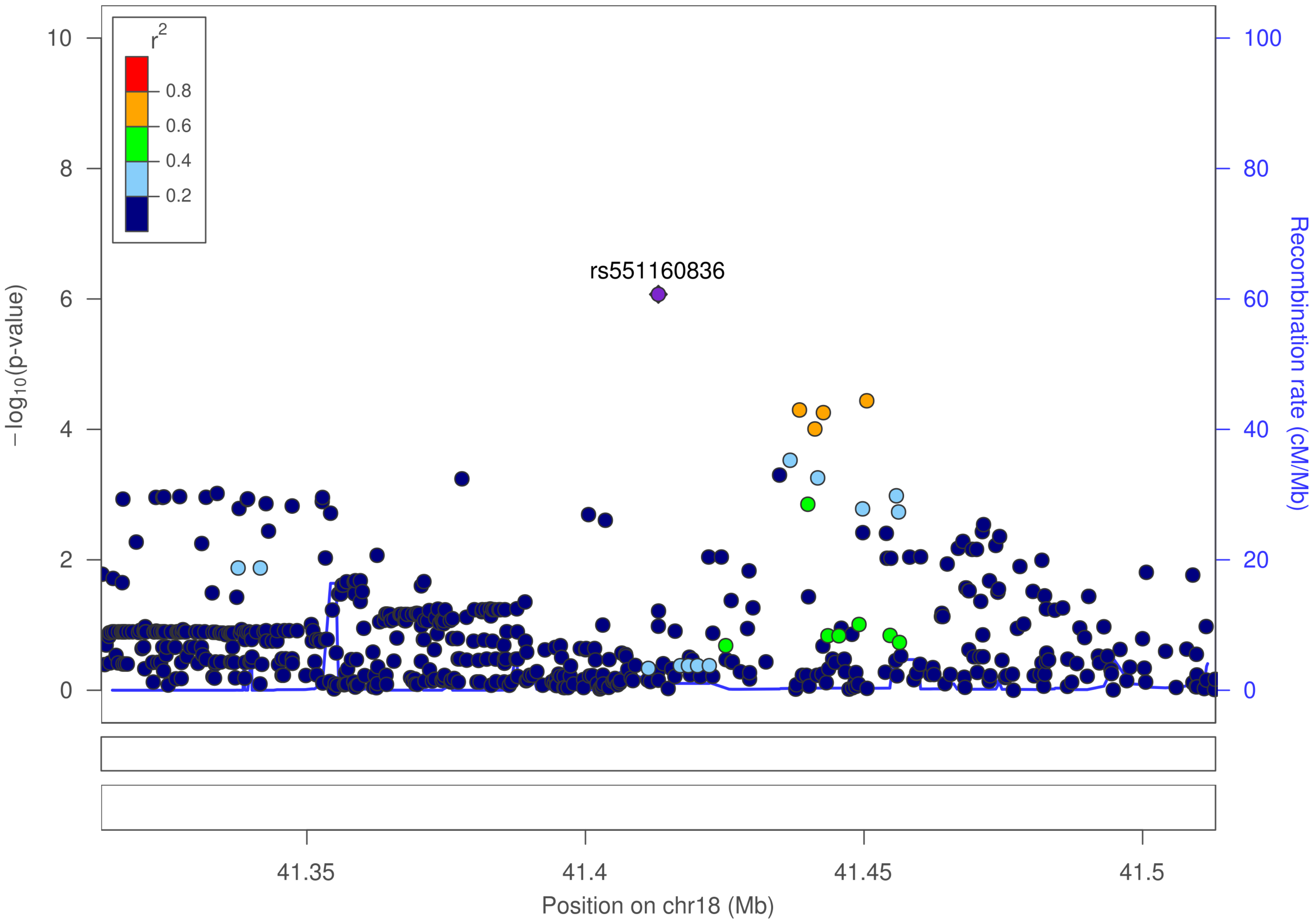
r2
0.8
0.6
0.4
0.2
rs551160836
10
8
6
4
2
0
−log10(p-value)
100
80
60
40
20
0
Recombination rate (cM/Mb)
41.35
41.4
41.45
41.5
Position on chr18 (Mb)
