# Supplementary material for: Novel and Known Gene-Smoking Interactions With cIMT Identified as Potential Drivers for Atherosclerosis Risk in West-African Populations of the AWI-Gen Study
Source: Front Genet. 2020 Feb 7;10:1354. doi: 10.3389/fgene.2019.01354 (PMC7025492; doi:10.3389/fgene.2019.01354)

Supplementary Figure 2a:

Genotypes plots of selected SNPs (p-values<1E-05) in Nanoro showing distibutions of mean cIMT residuals in smokers and non-smokers groups.
Mean_cIMT_Res are in mm.

rs699402

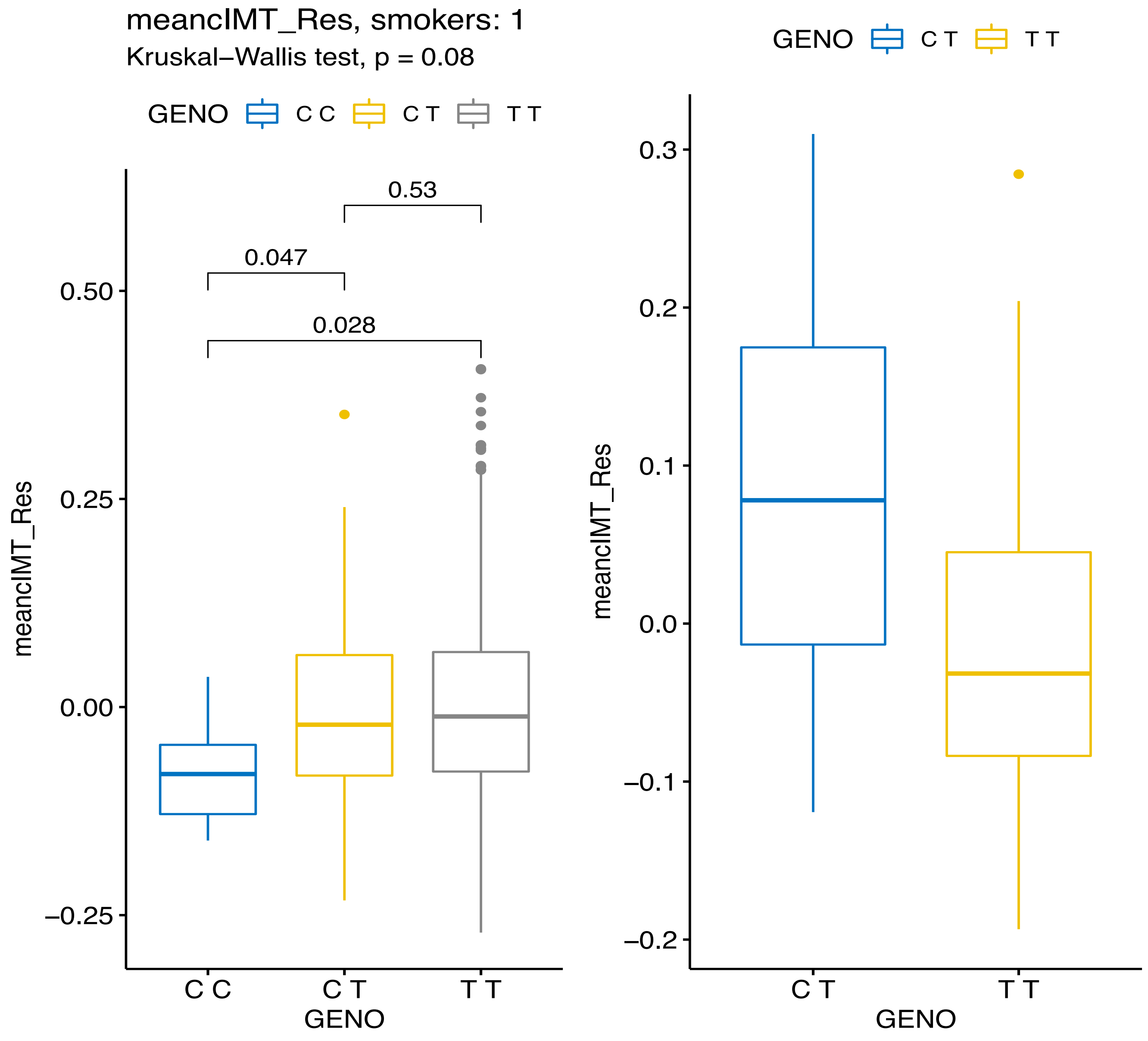
meancIMT_Res, smokers: 1
Kruskal–Wallis test, p = 0.08
GENO
C C
C T
T T
0.53
0.047
0.028
0.50
0.25
0.00
−0.25
meancIMT_Res
C C
C T
T T
GENO
GENO
C T
T T
0.3
0.2
0.1
0.0
−0.1
−0.2
meancIMT_Res
C T
T T
GENO

rs1161464

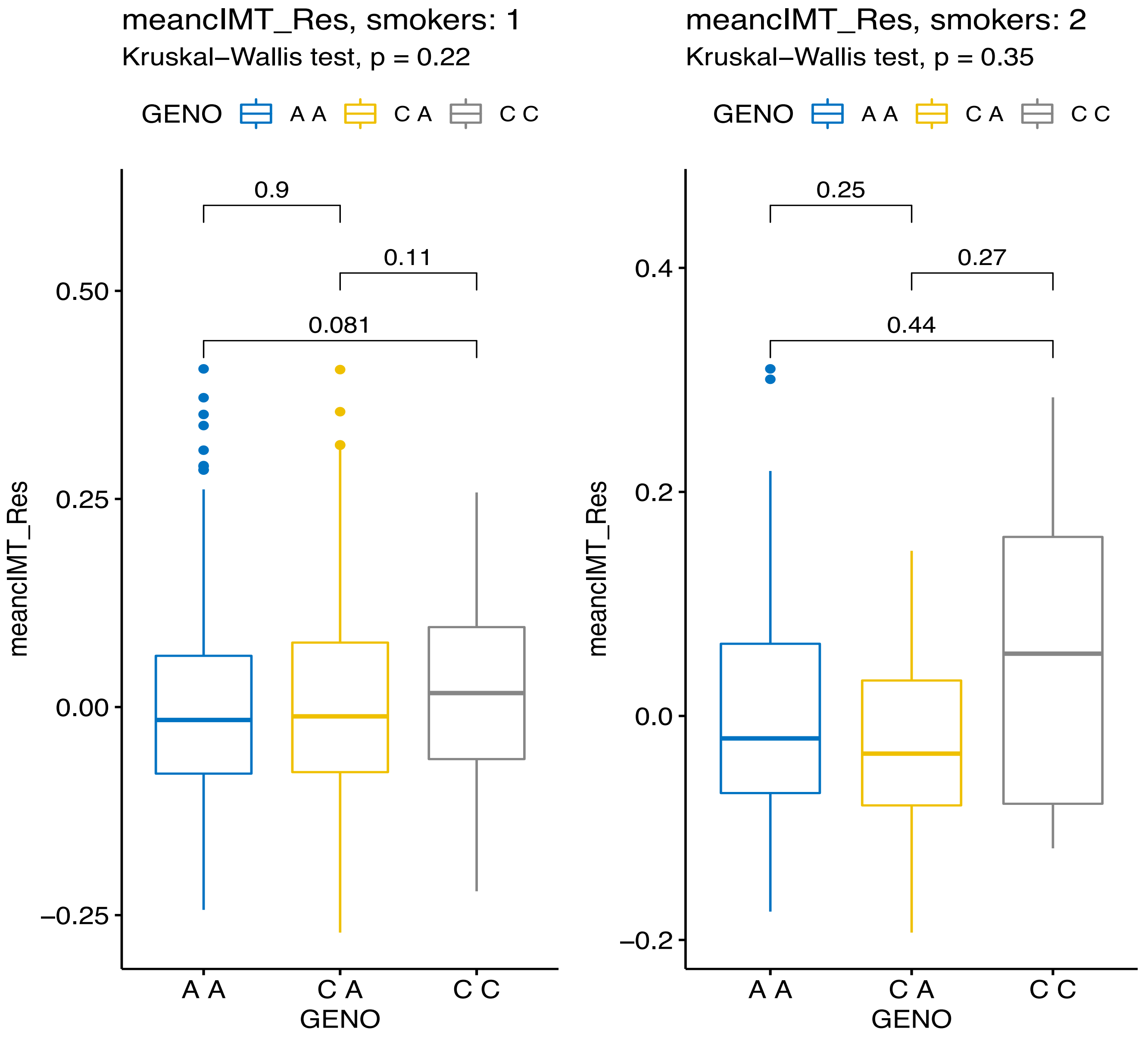
meancIMT_Res, smokers: 1
Kruskal–Wallis test, p = 0.22
GENO A A C A C C
0.9
0.11
0.081
0.50
0.25
0.00
−0.25
meancIMT_Res
A A C A C C
GENO
meancIMT_Res, smokers: 2
Kruskal–Wallis test, p = 0.35
GENO A A C A C C
0.25
0.27
0.44
0.4
0.2
0.0
−0.2
meancIMT_Res
A A C A C C
GENO

rs2357001

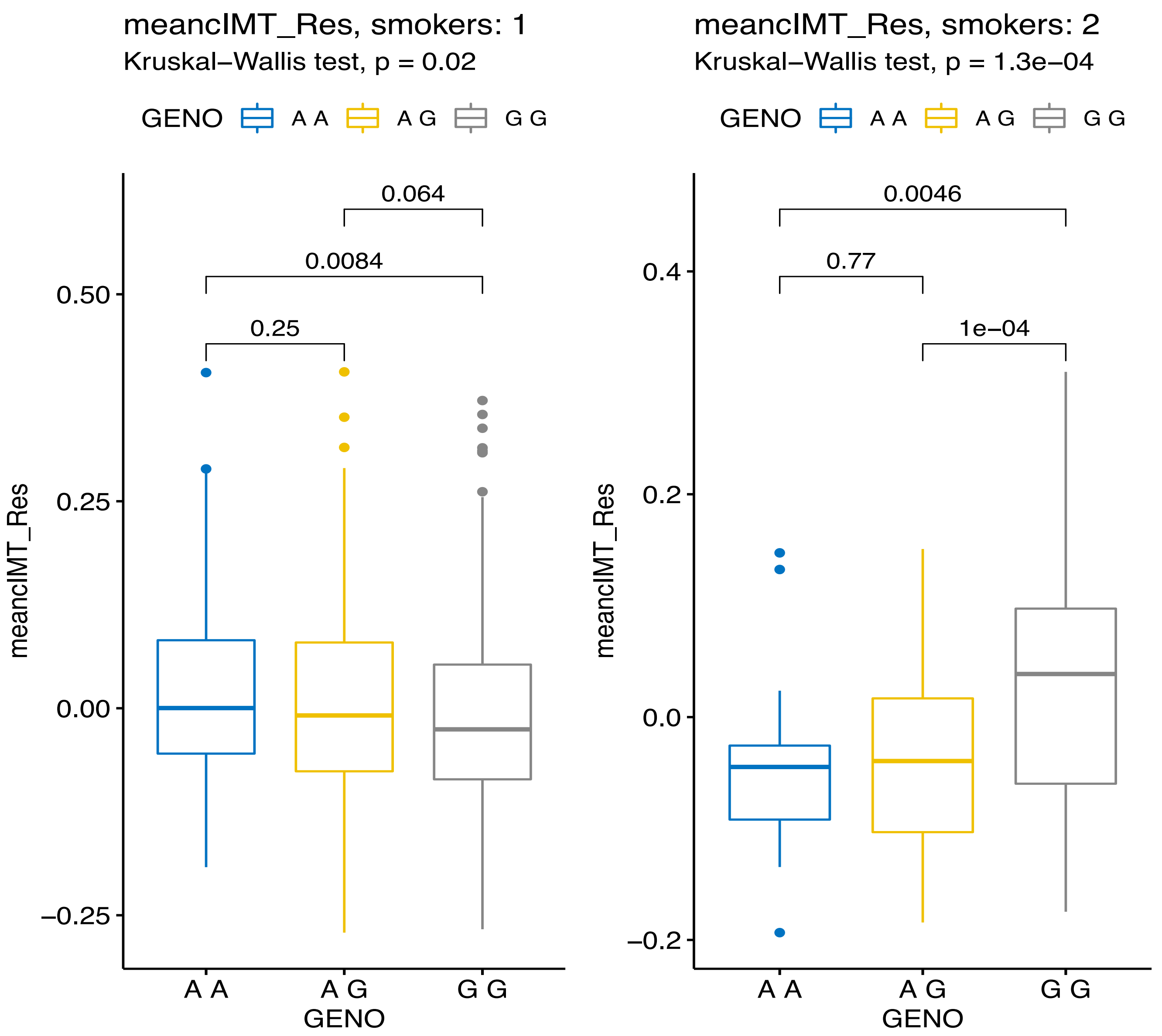
meancIMT_Res, smokers: 1
Kruskal–Wallis test, p = 0.02
GENO
A A
A G
G G
0.064
0.0084
0.25
0.50
0.25
0.00
−0.25
meancIMT_Res
GENO
meancIMT_Res, smokers: 2
Kruskal–Wallis test, p = 1.3e−04
GENO
A A
A G
G G
0.0046
0.77
1e−04
0.4
0.2
0.0
−0.2
meancIMT_Res
GENO

geno-rs2407218

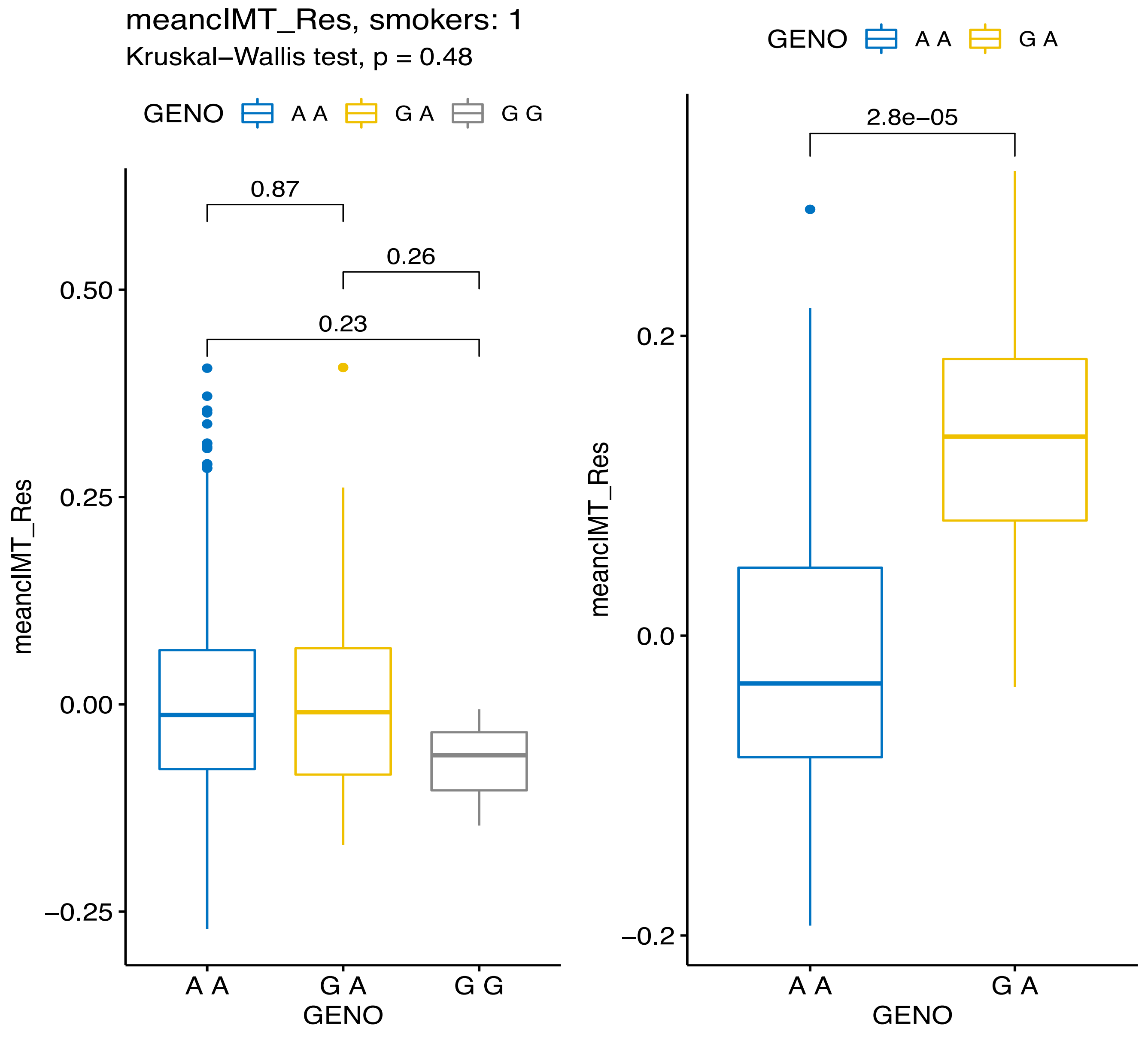

geno-rs2819241

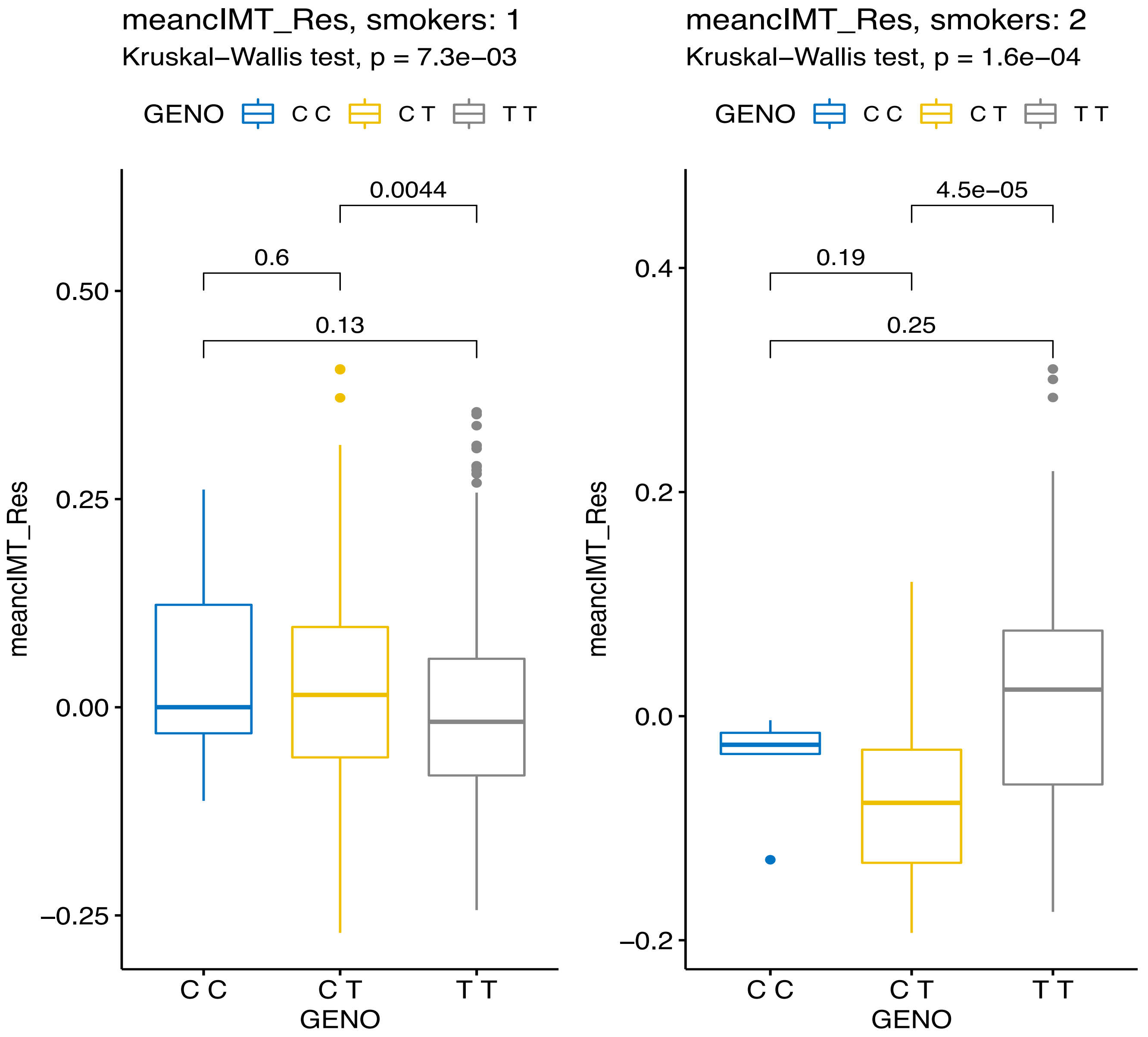

geno-rs4728159

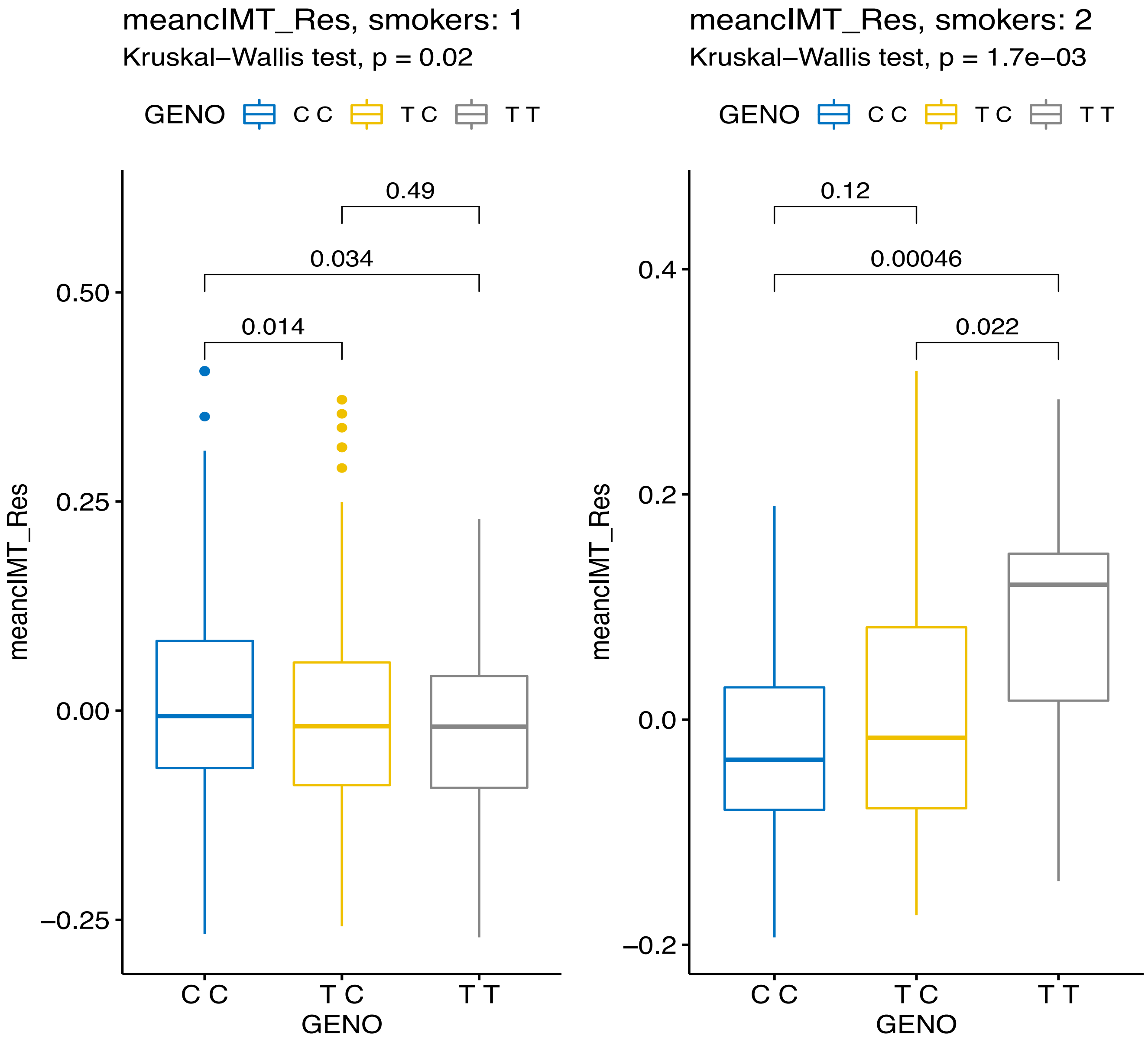

geno-rs6025645

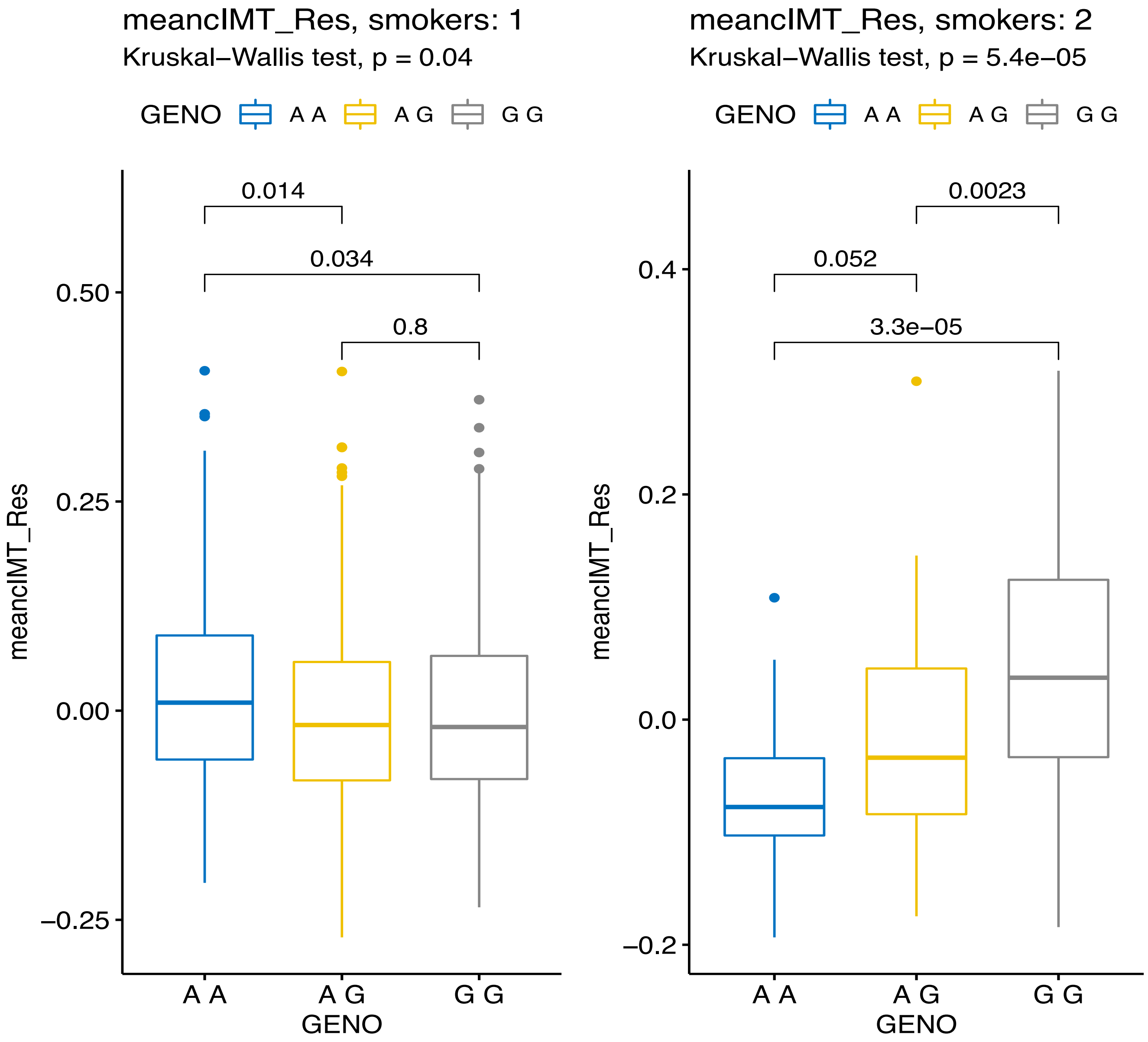

geno-rs6978311

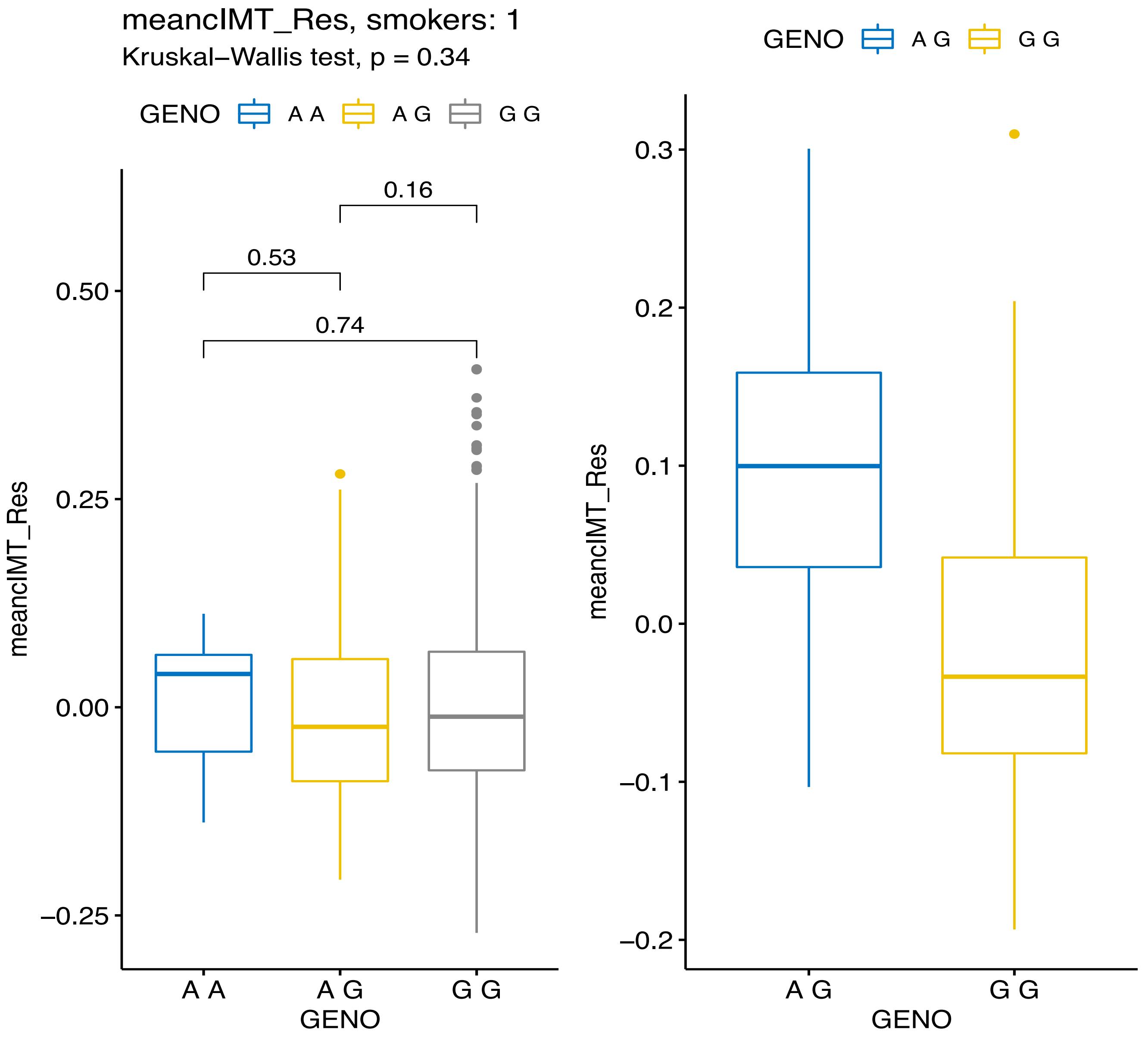

geno-rs7095209

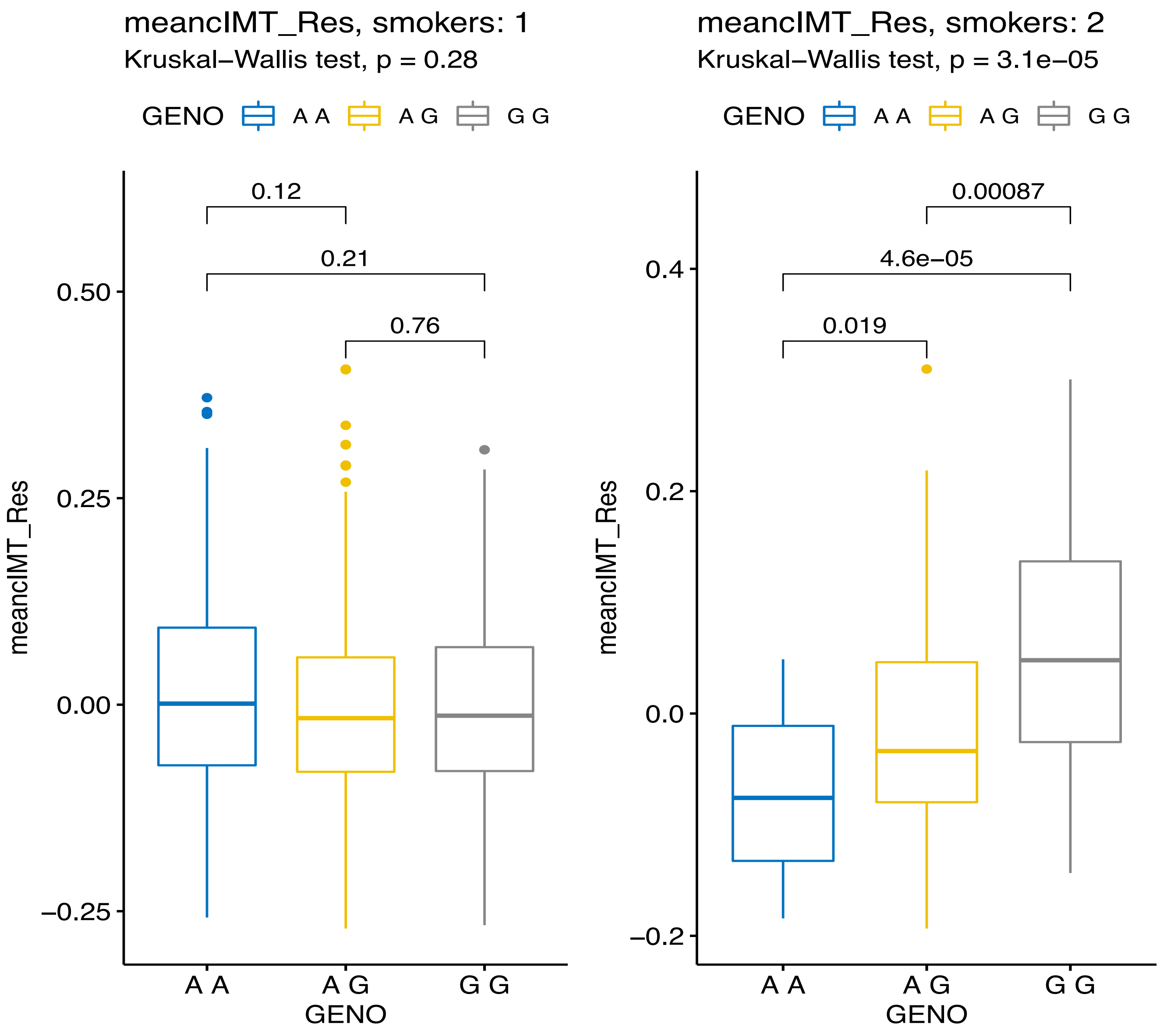

geno-rs7649061

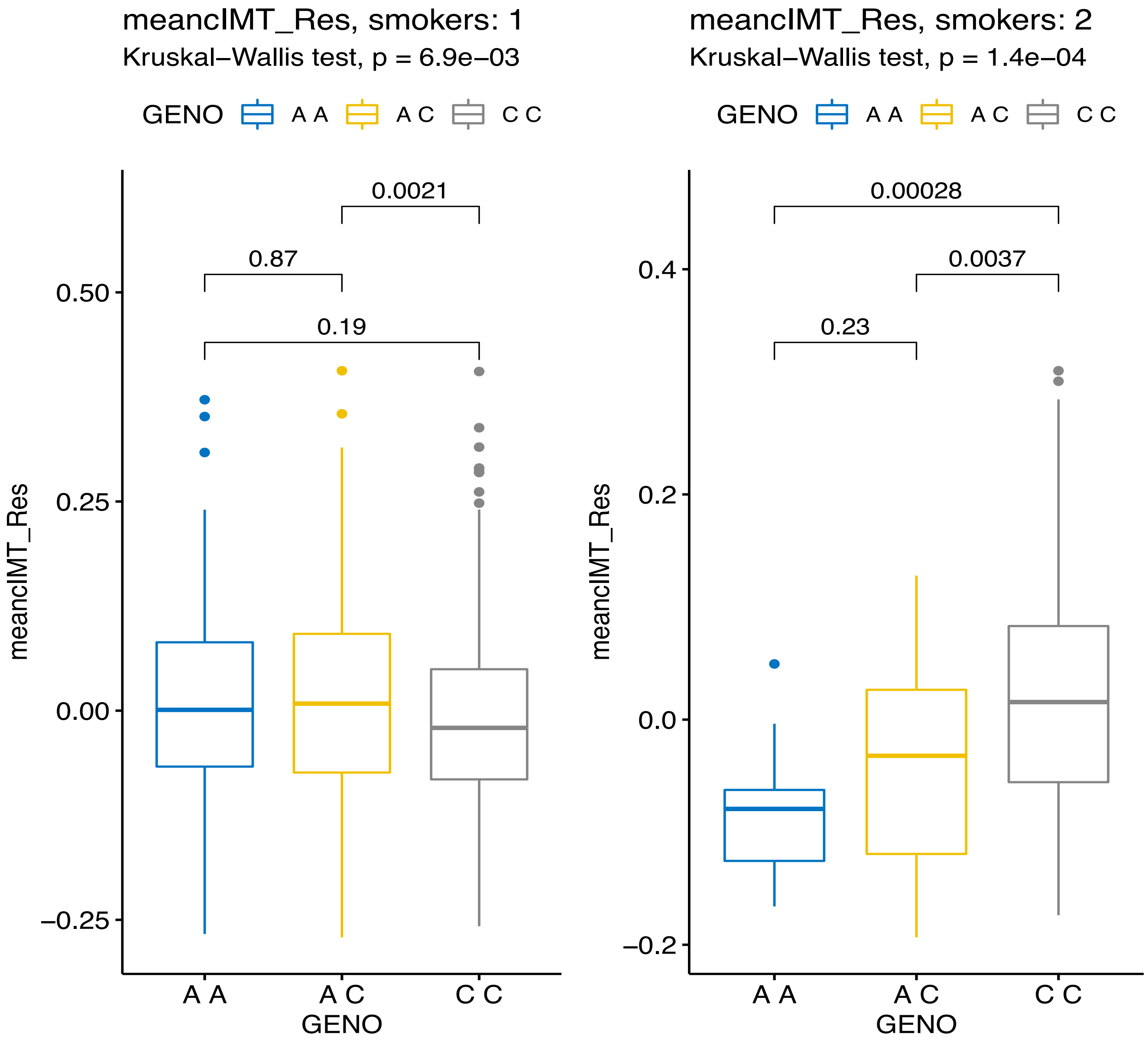

geno-rs9304018

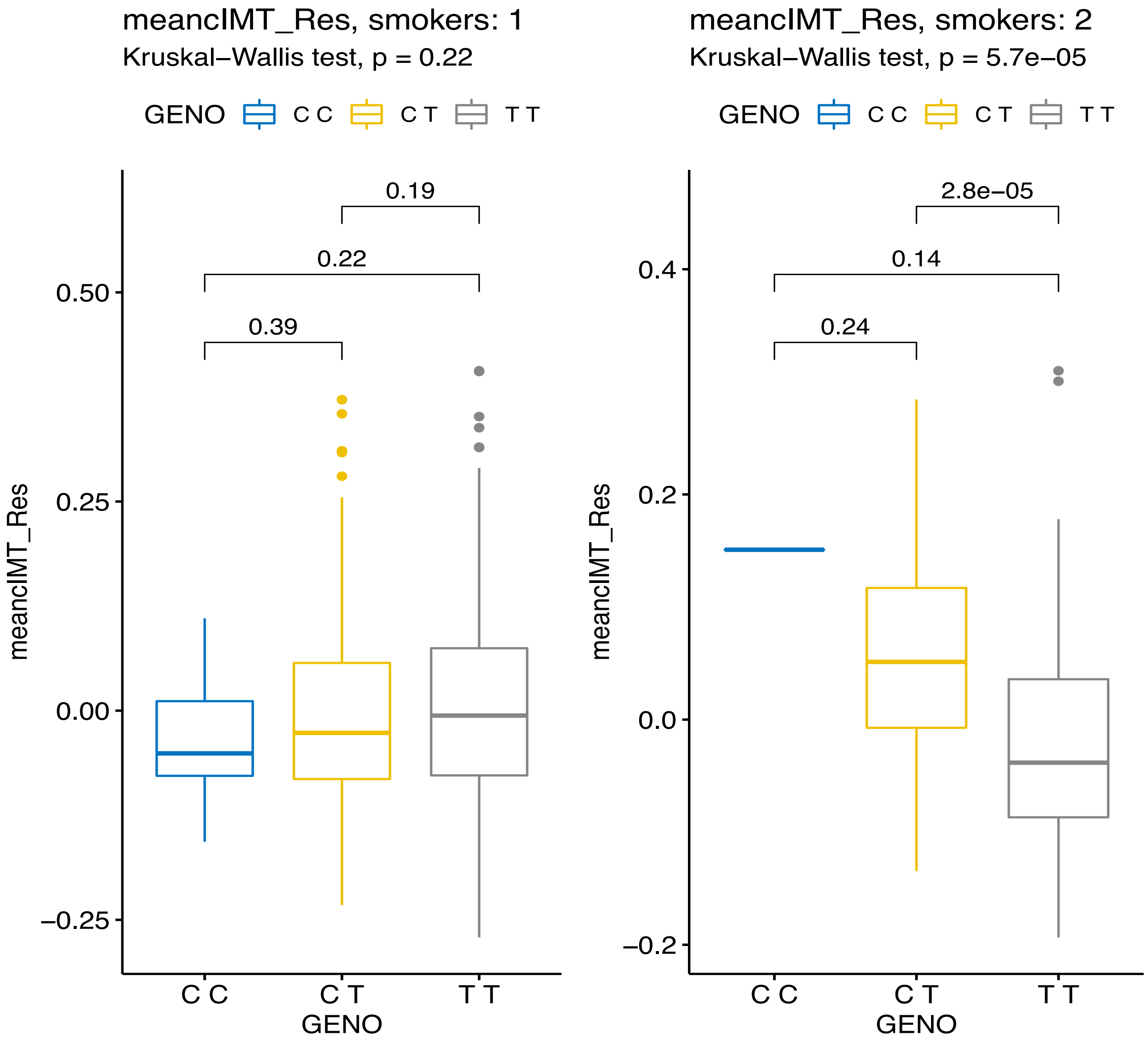

geno-rs9862699

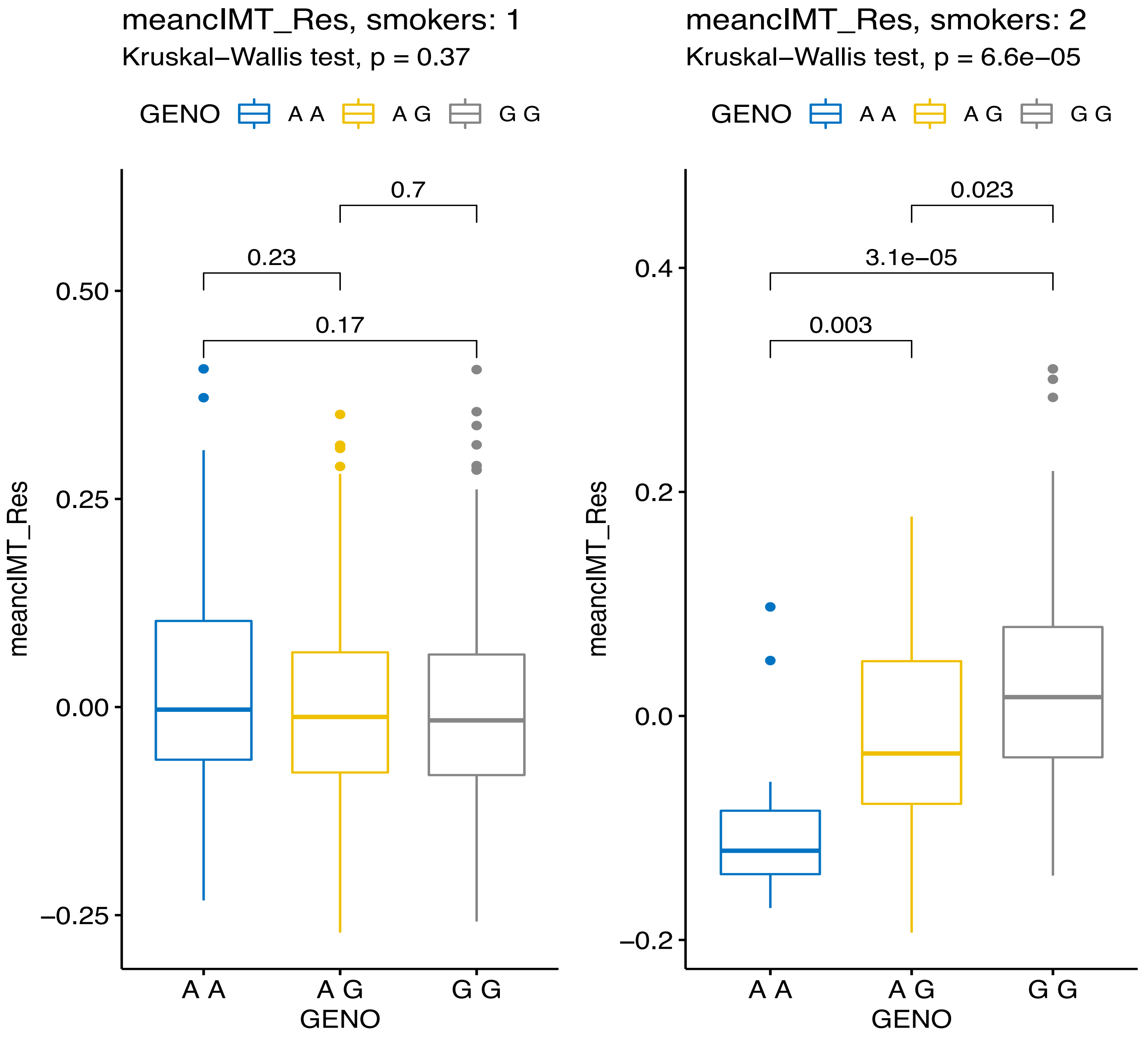

geno-rs9976292

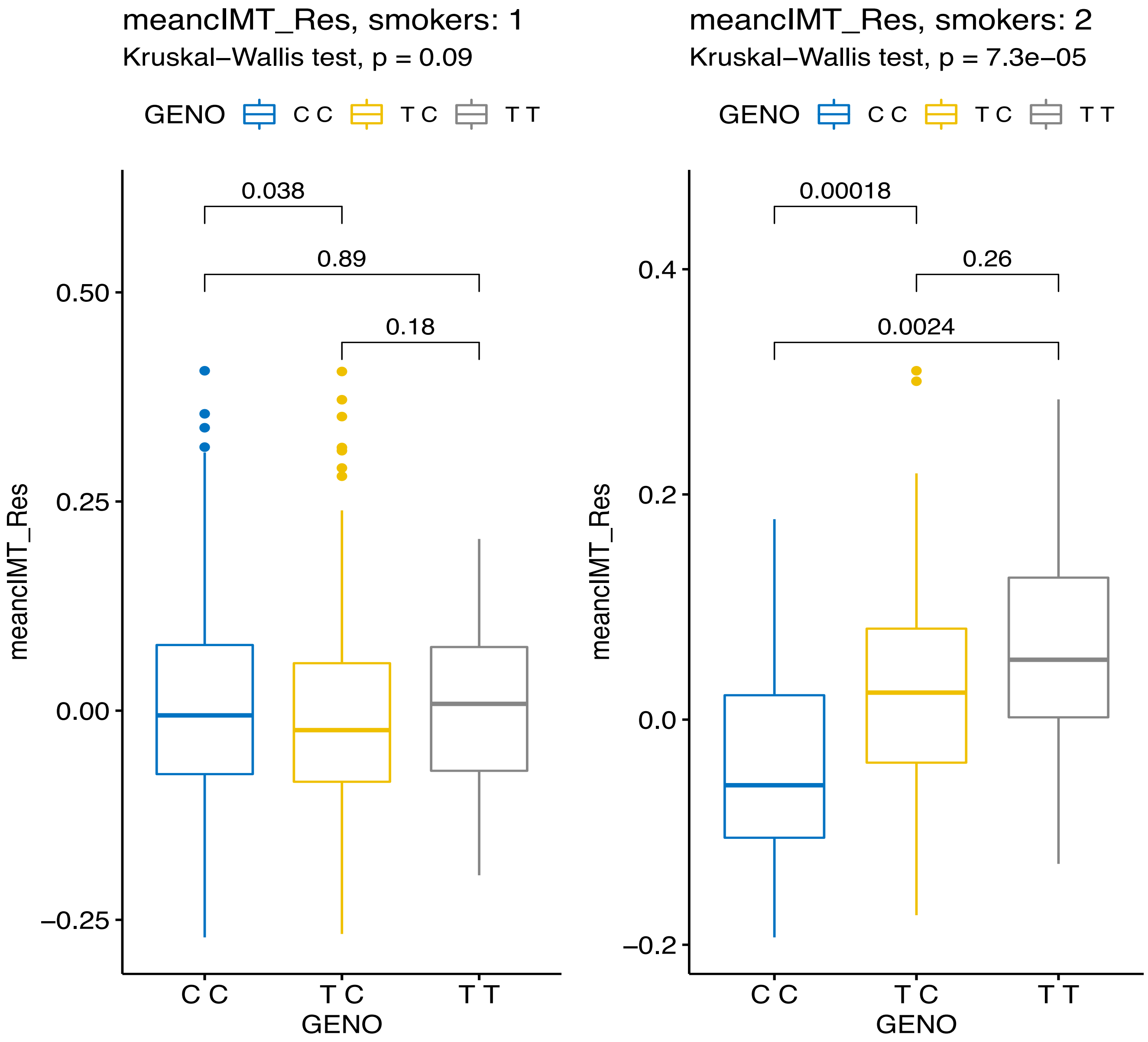

geno-rs11126201

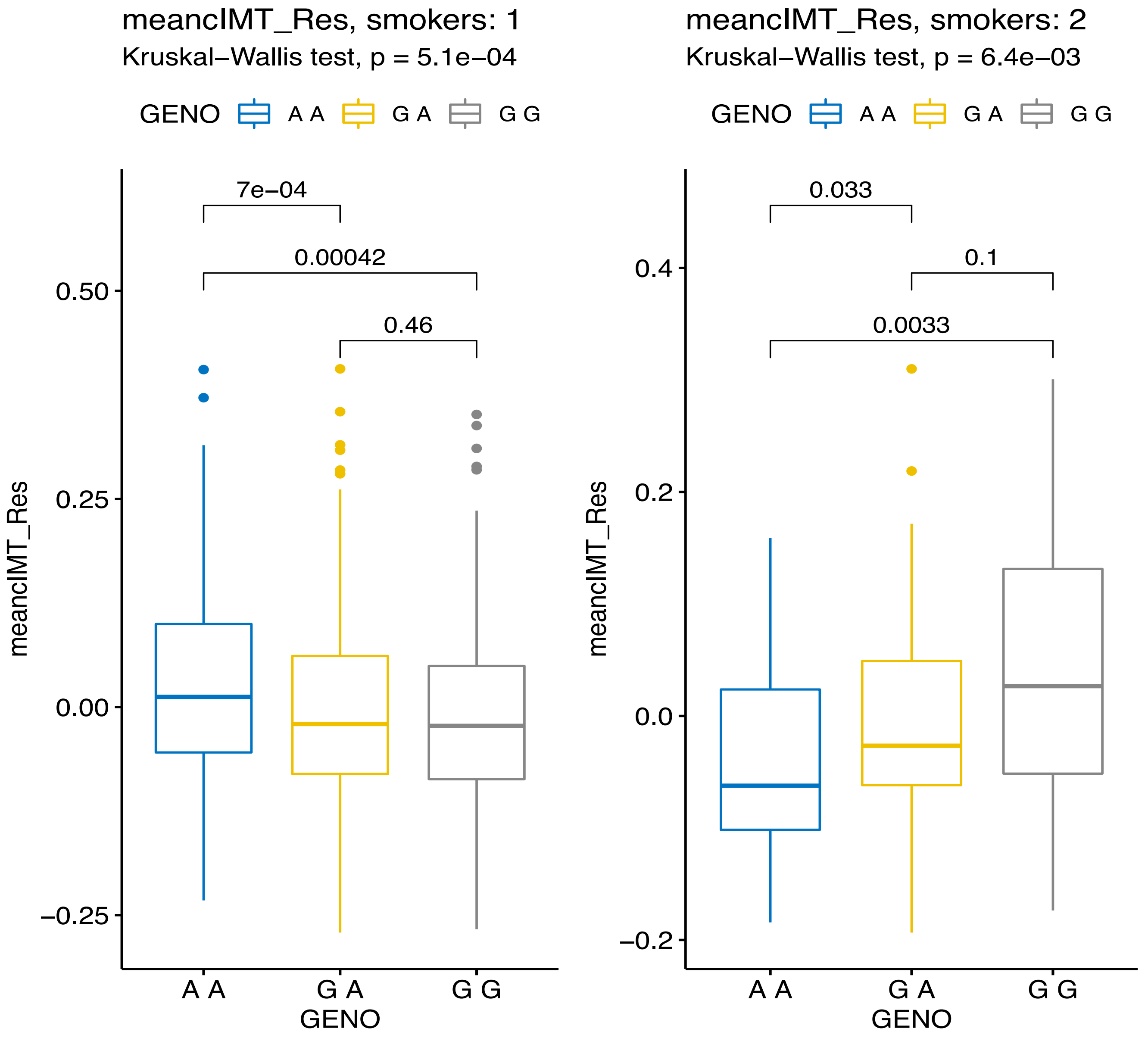

geno-rs11161464

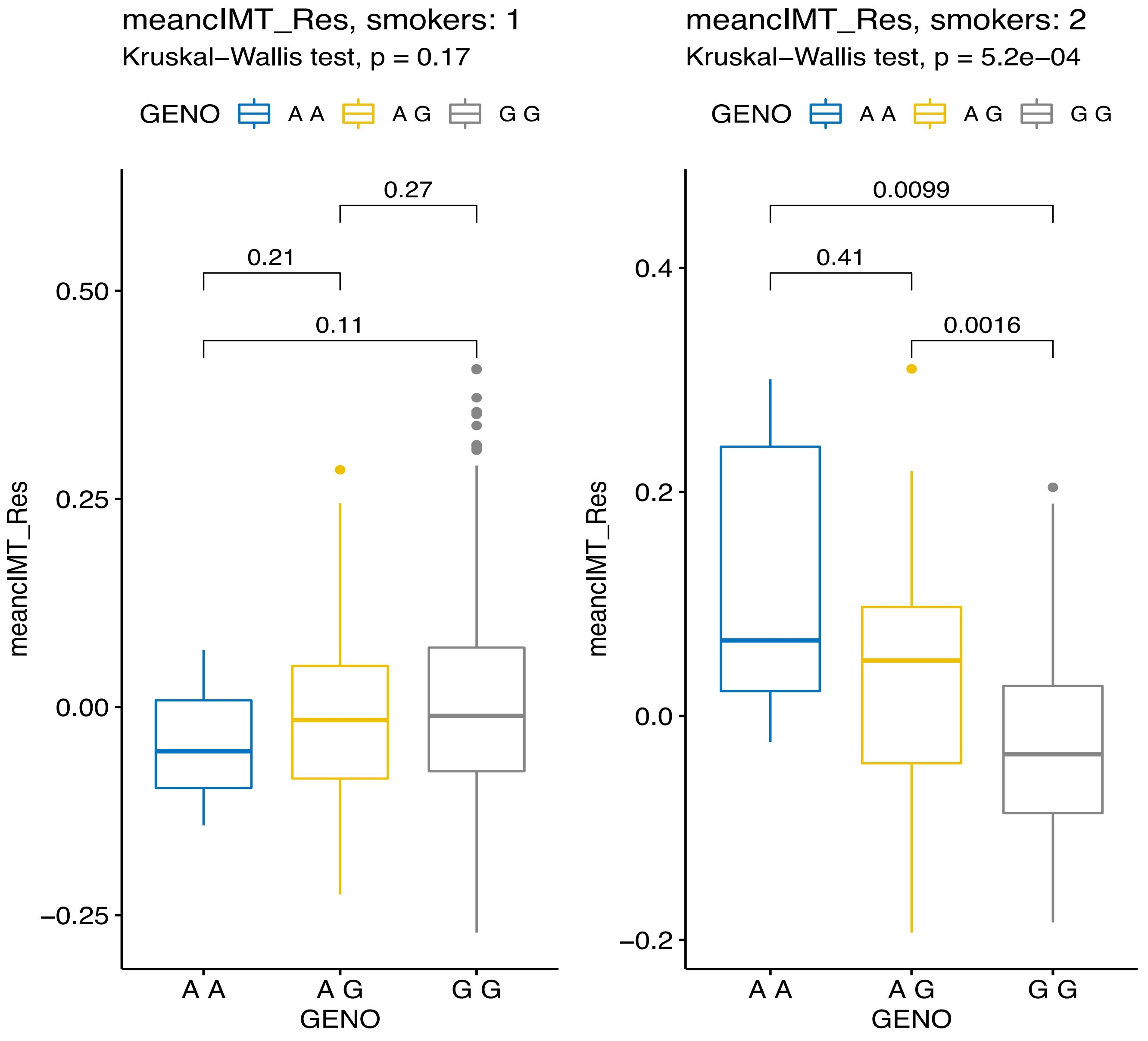

geno-rs11663276

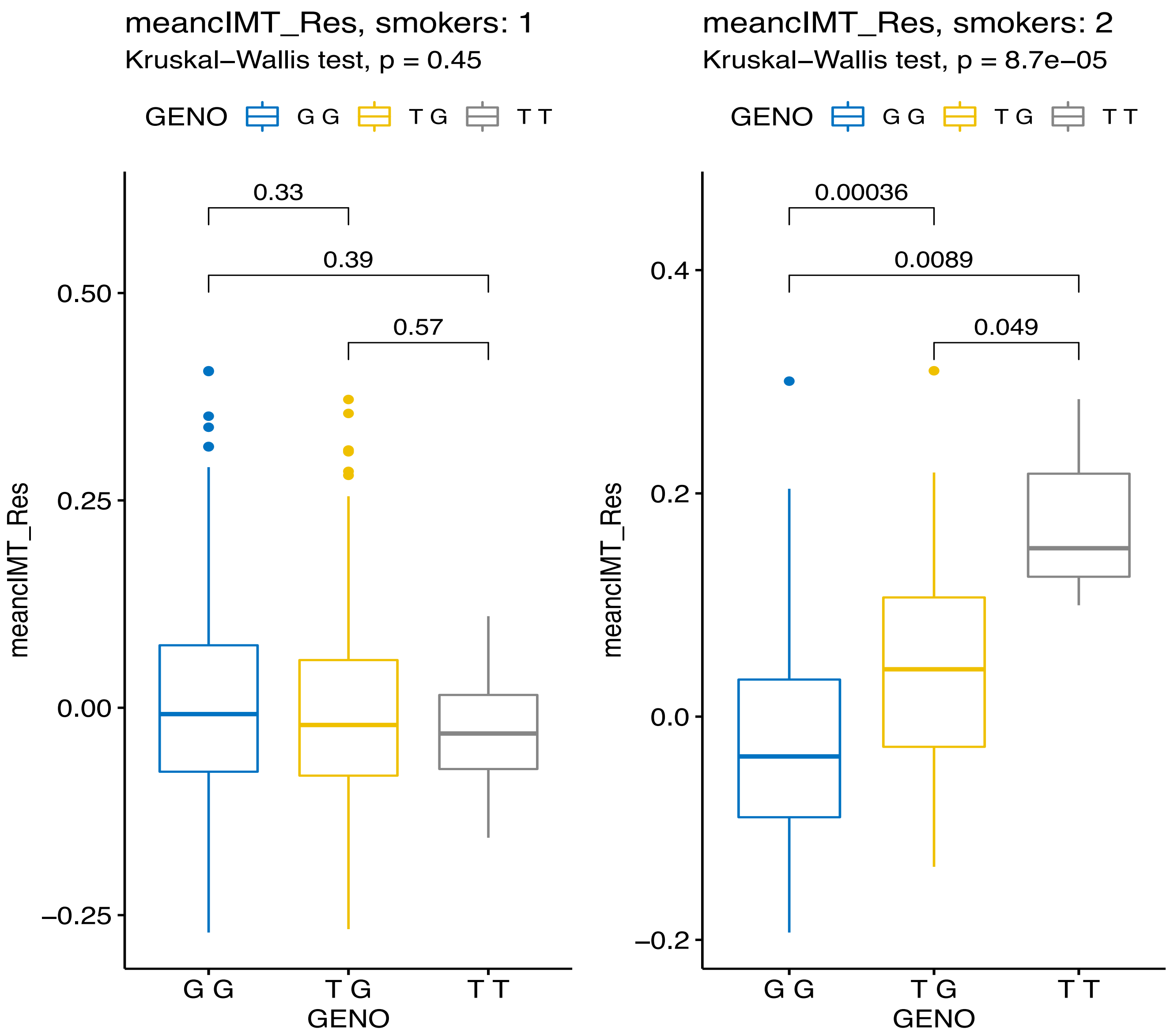

geno-rs13268575

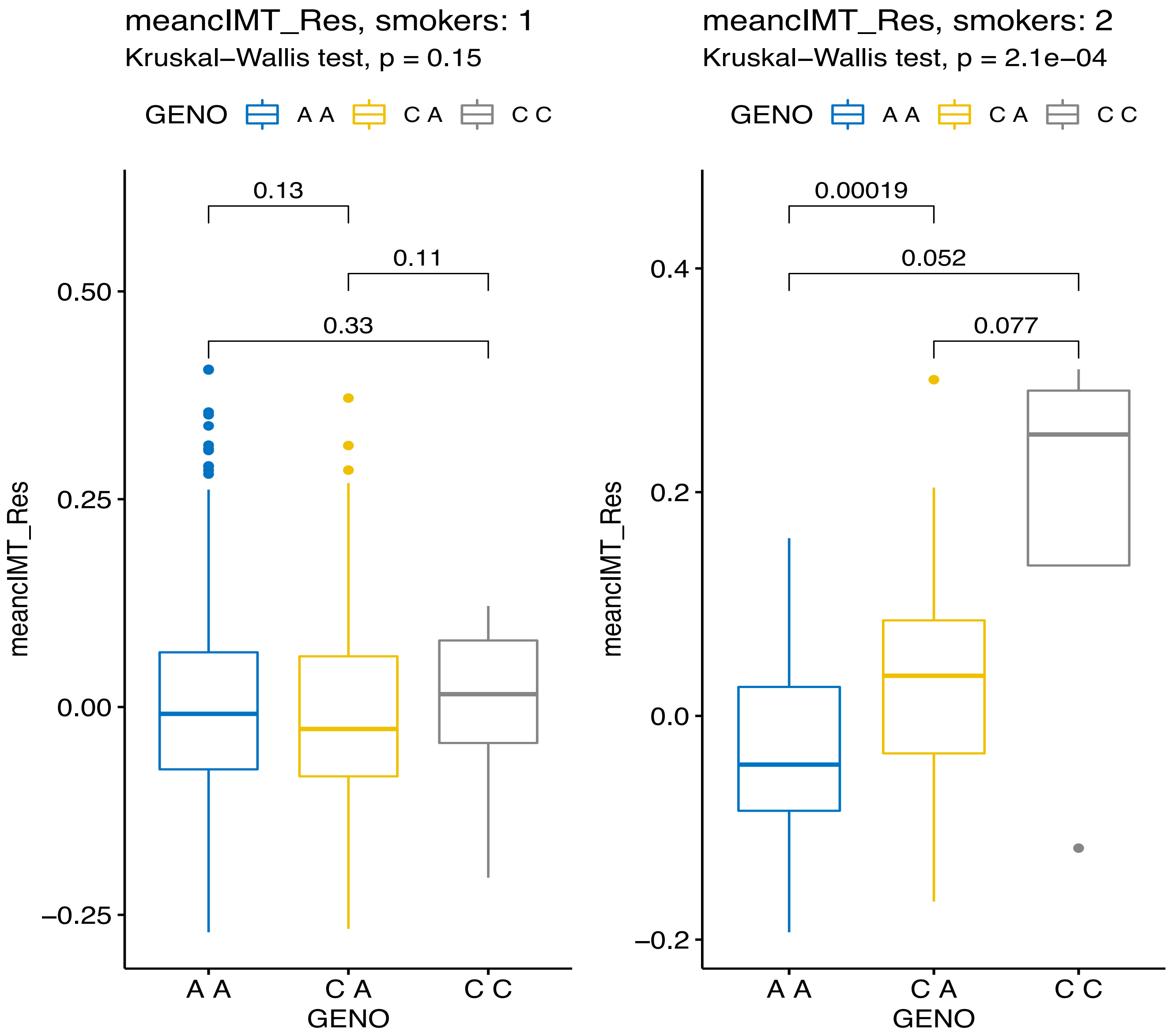

geno-rs28890775

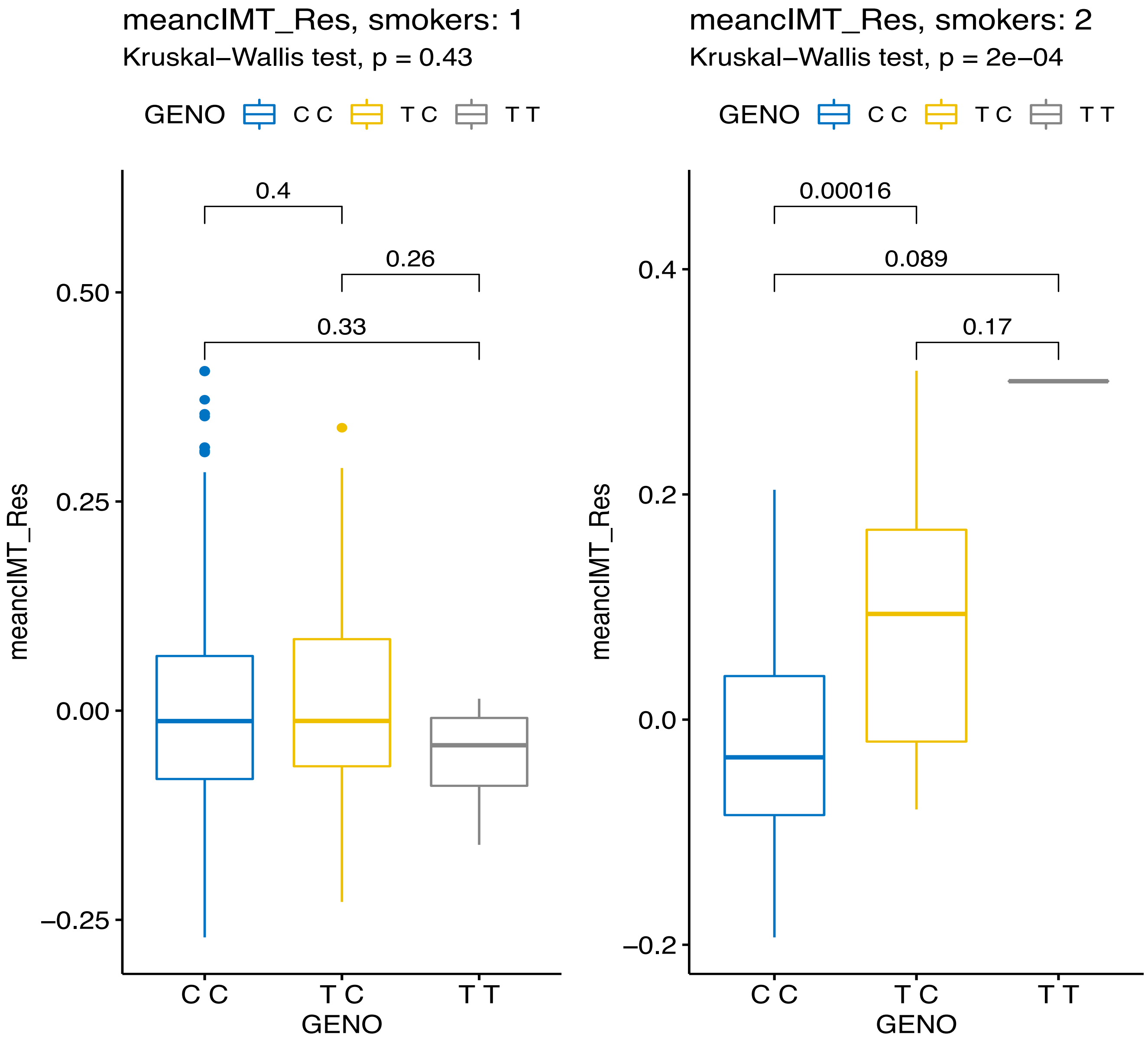

geno-rs55729345

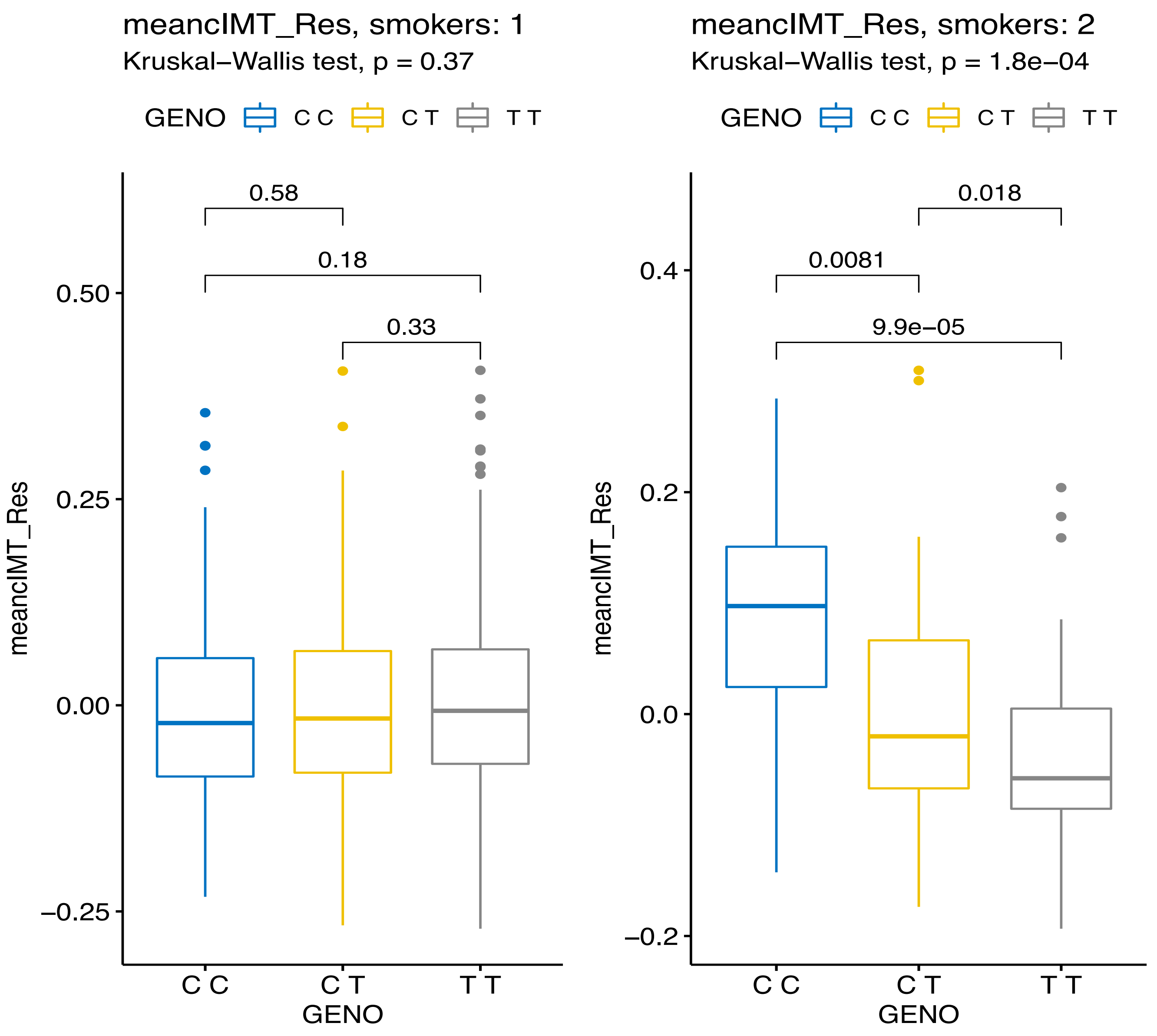

geno-rs59236450

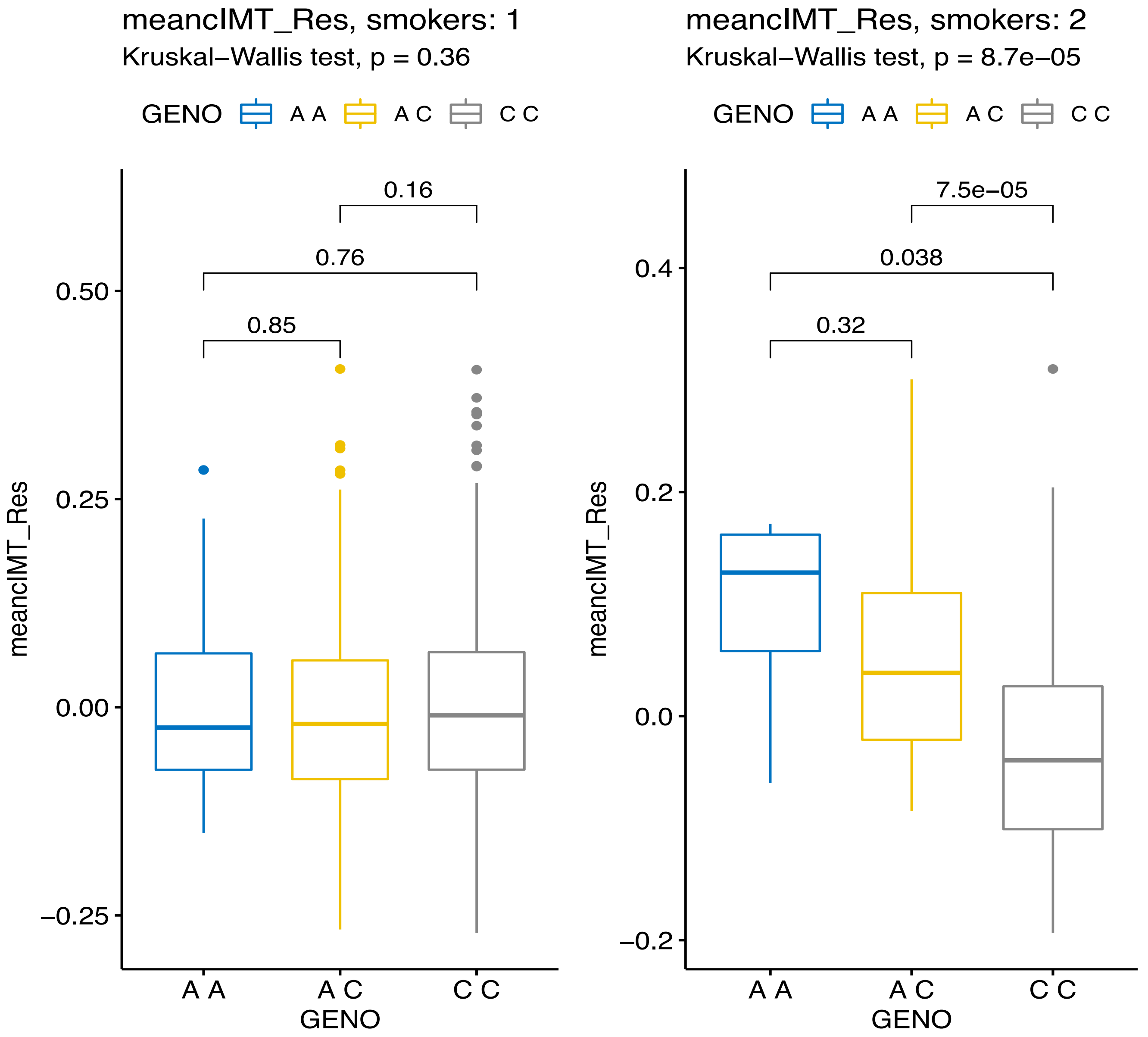

geno-rs59322395

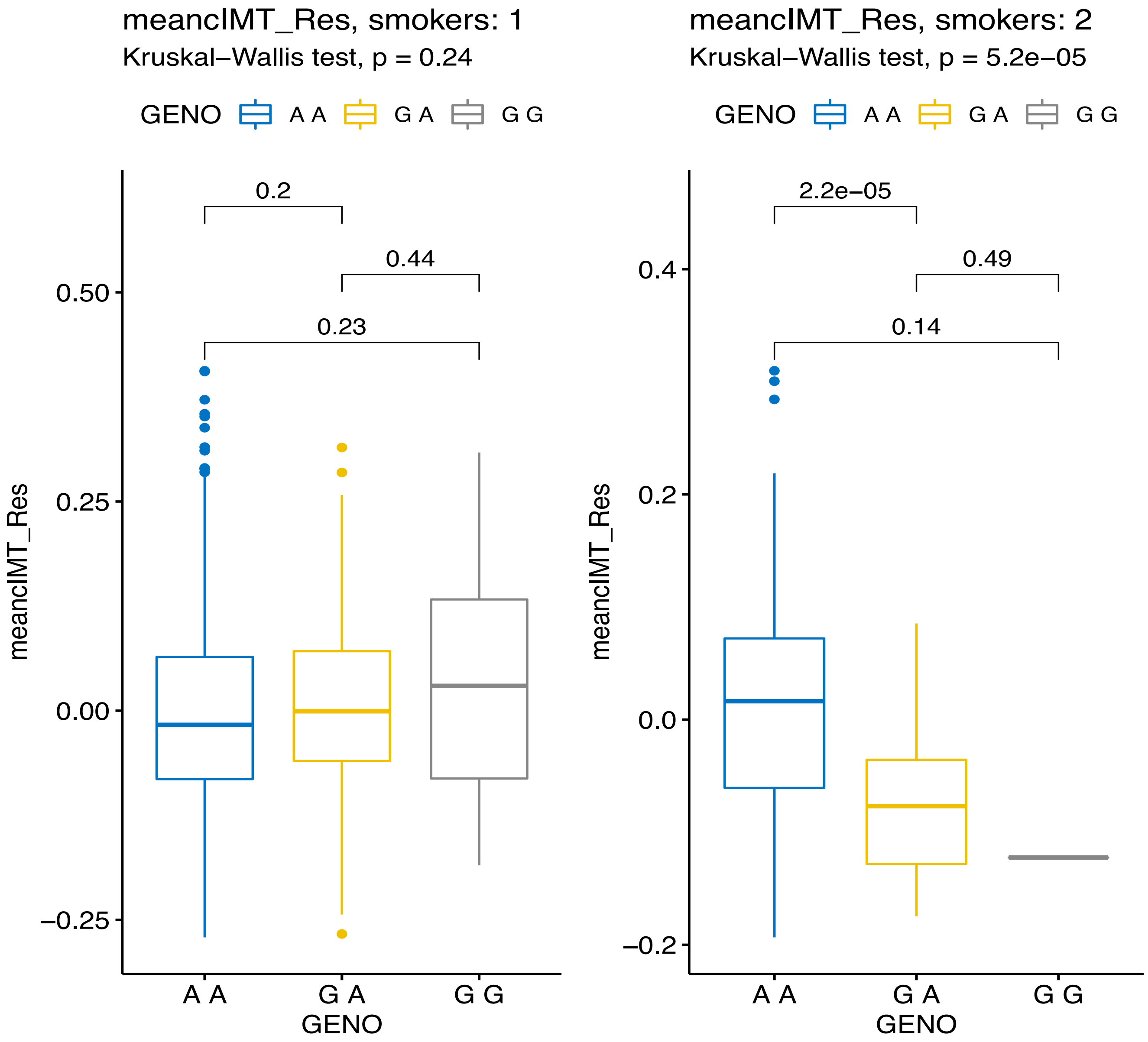

geno-rs73880644

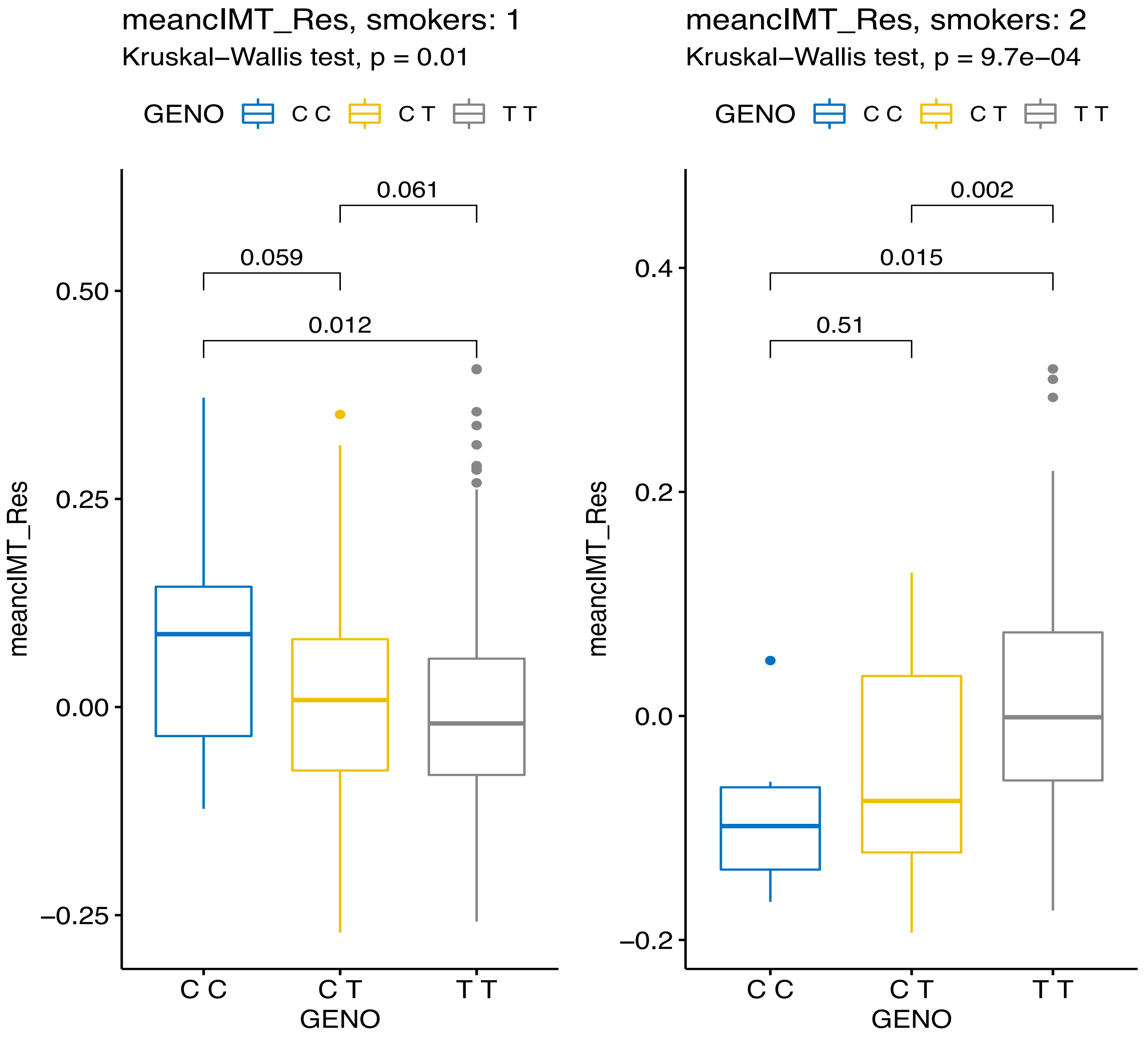

geno-rs74032742

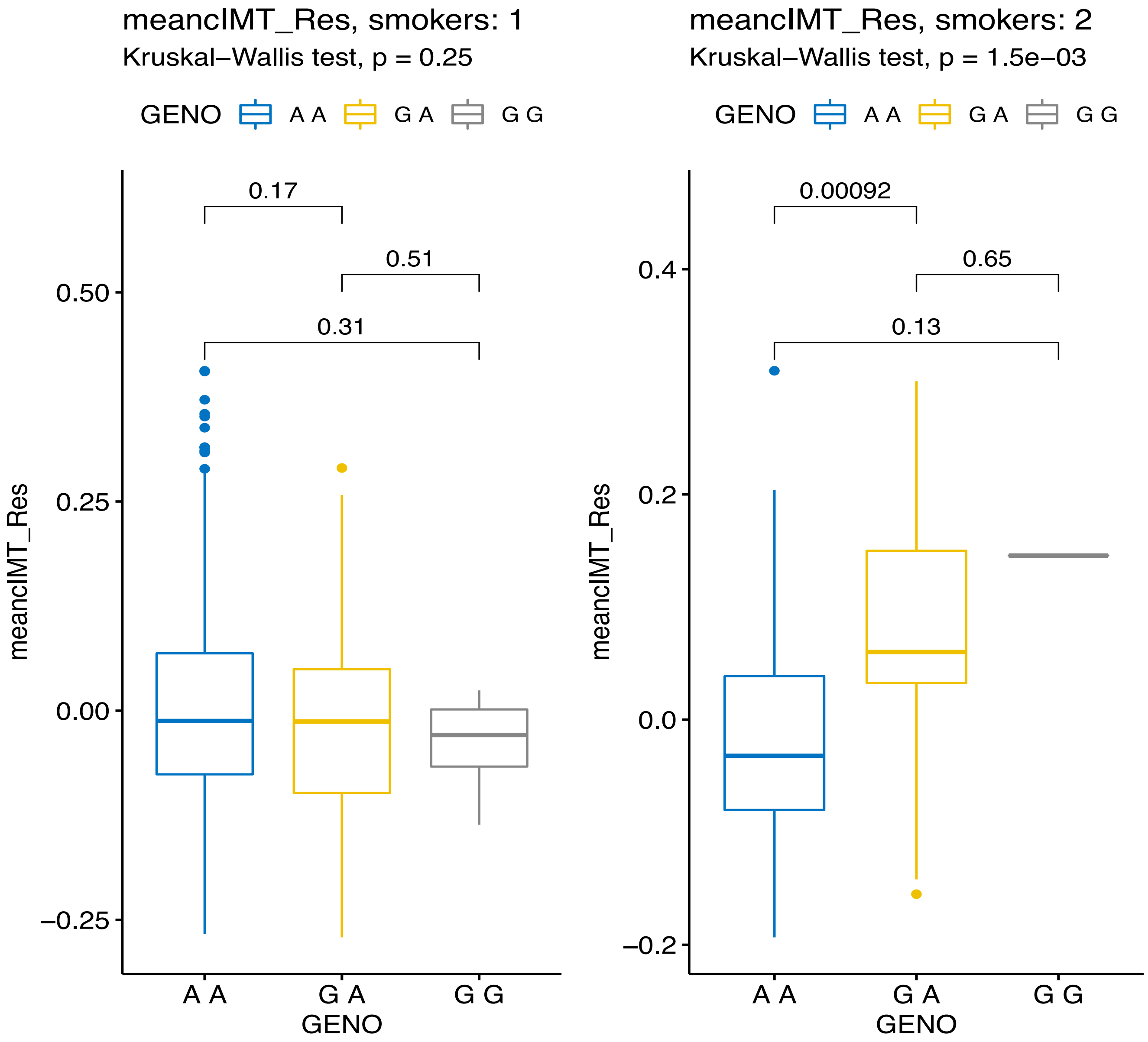

geno-rs113096000

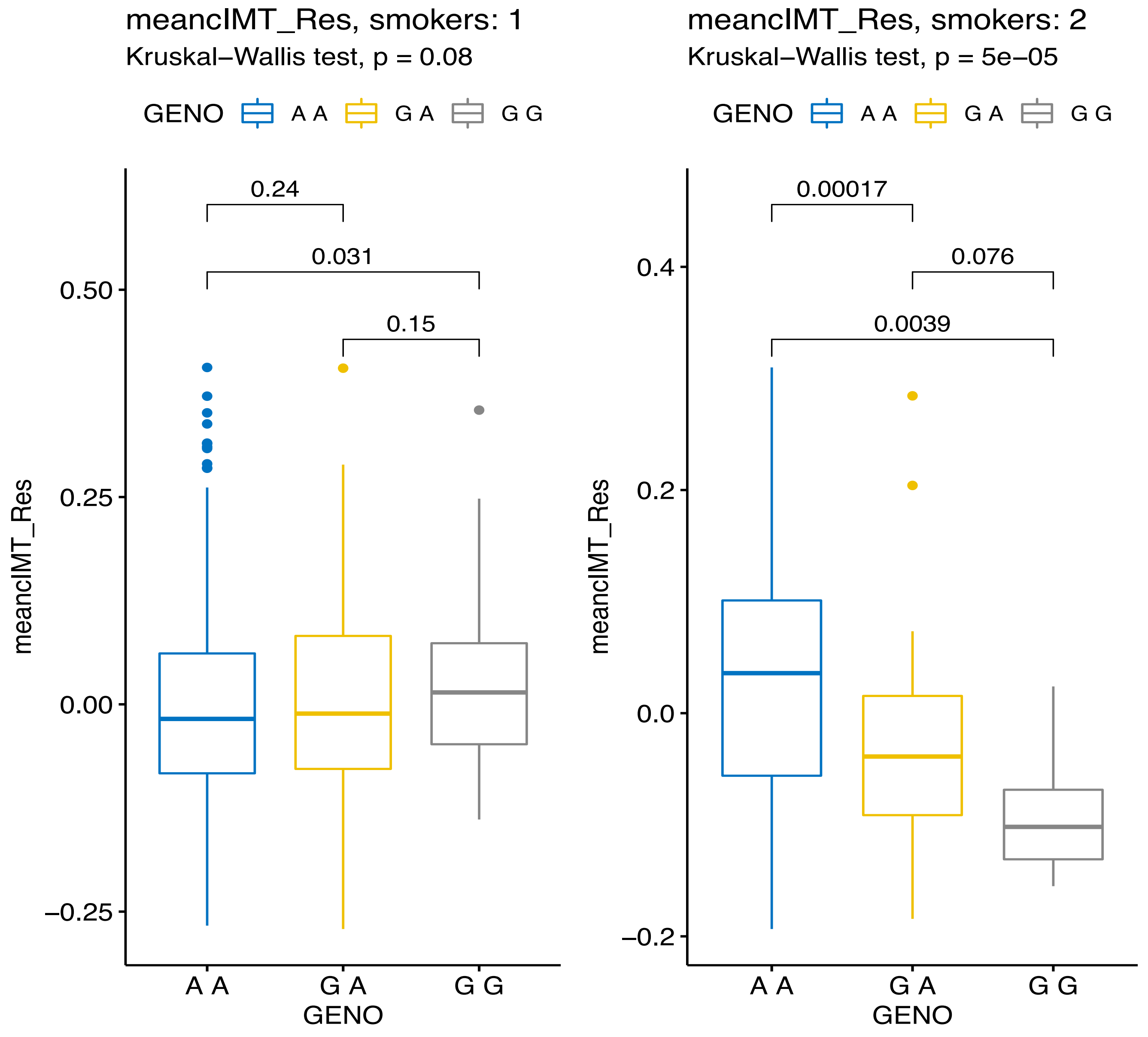

geno-rs144170770

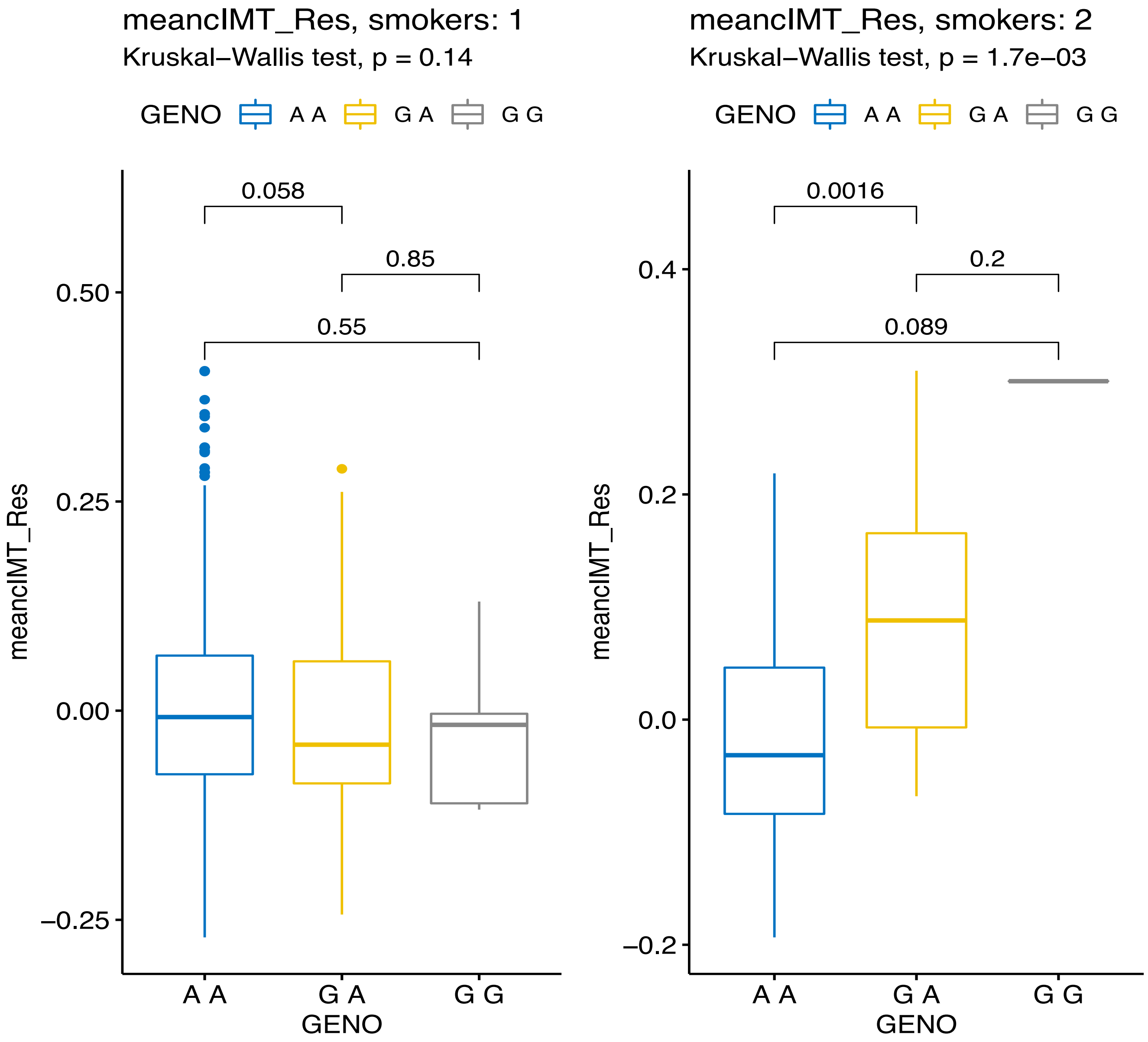

Supplementary Figure 2b:

Genotypes plots of selected SNPs (p-values<1E-05) in Navrongo, showing distibutions of mean cIMT residuals in somkers and non-smokers groups

geno-rs452108

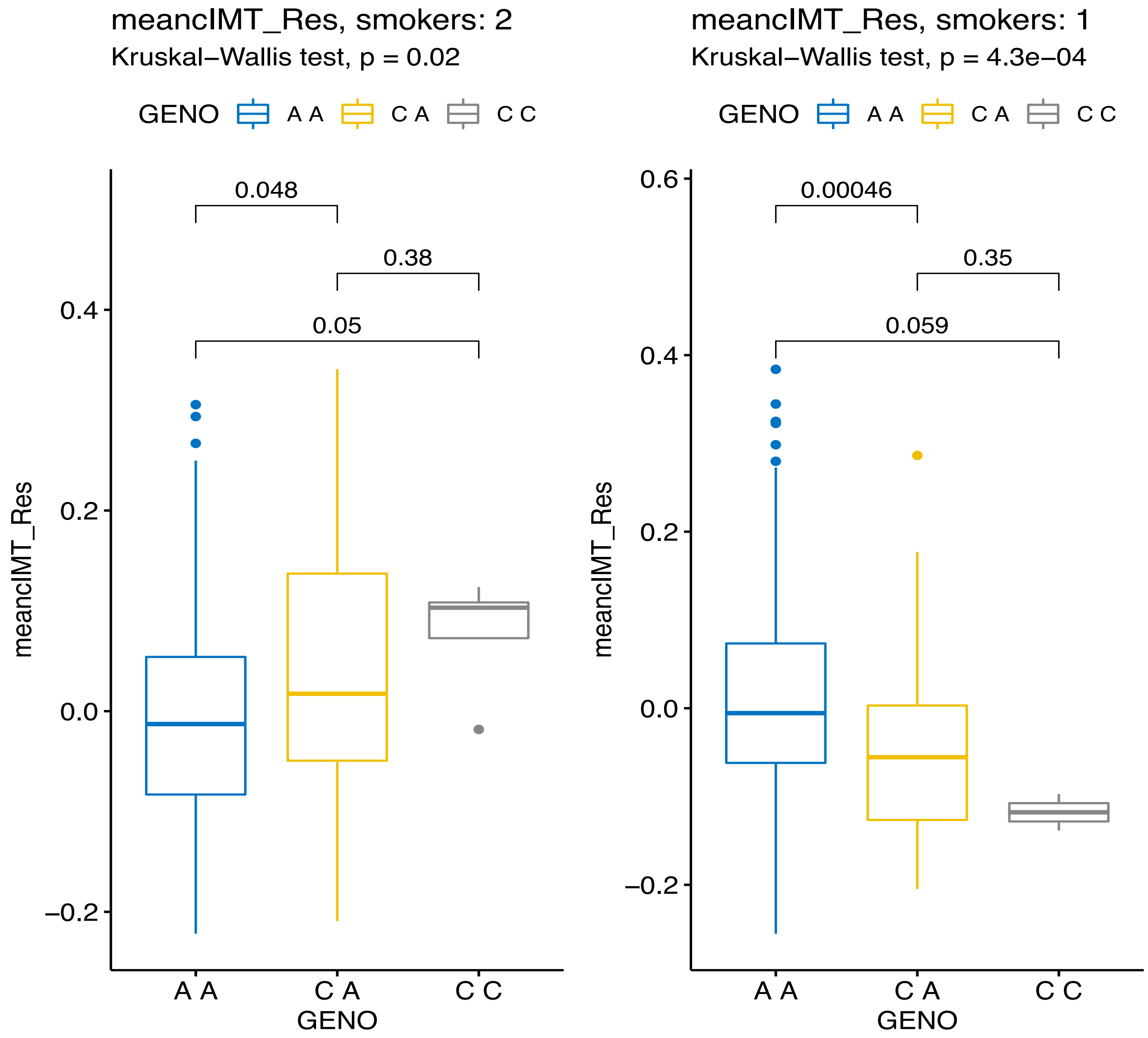

geno-rs2849519

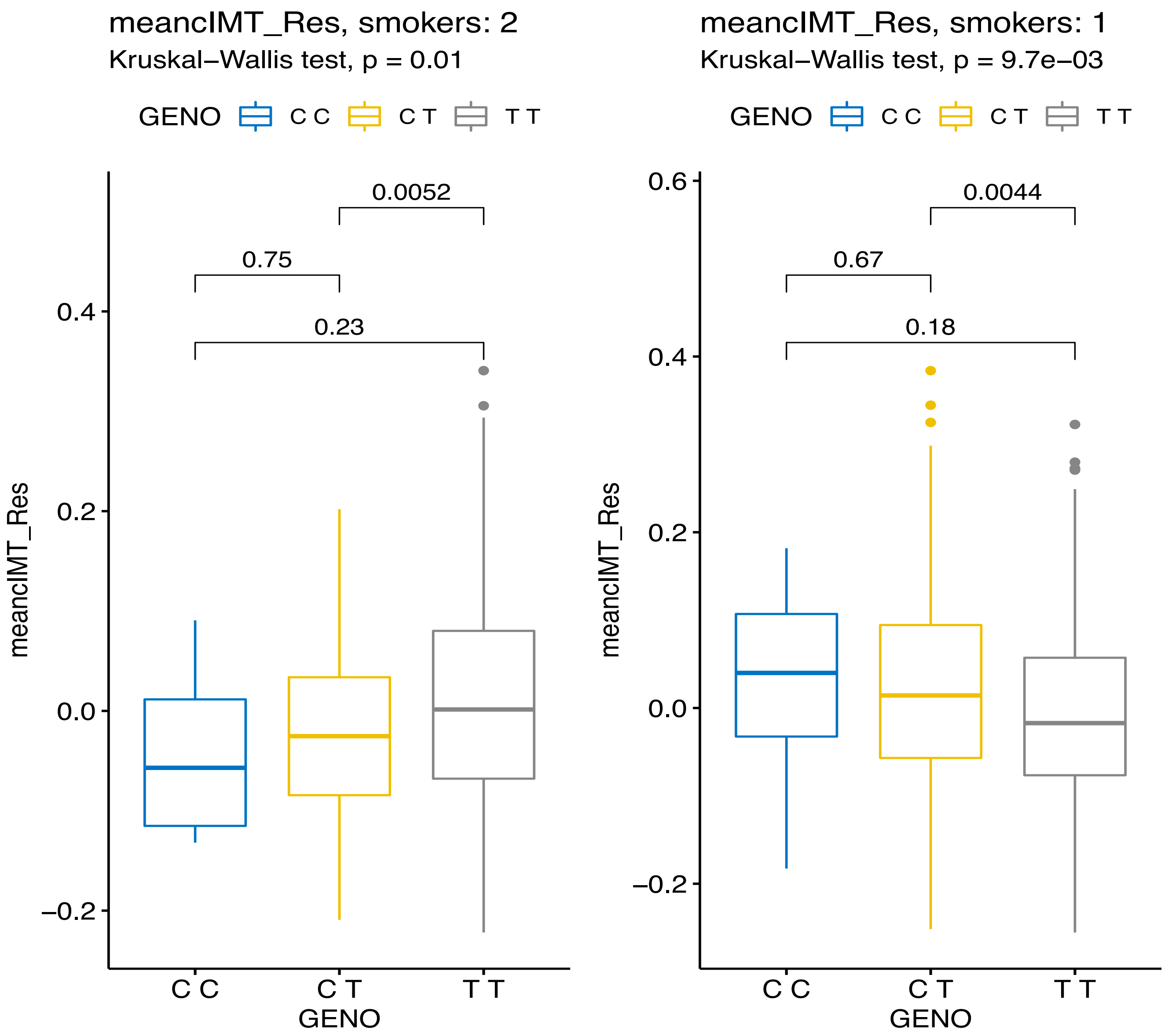

geno-rs4791040

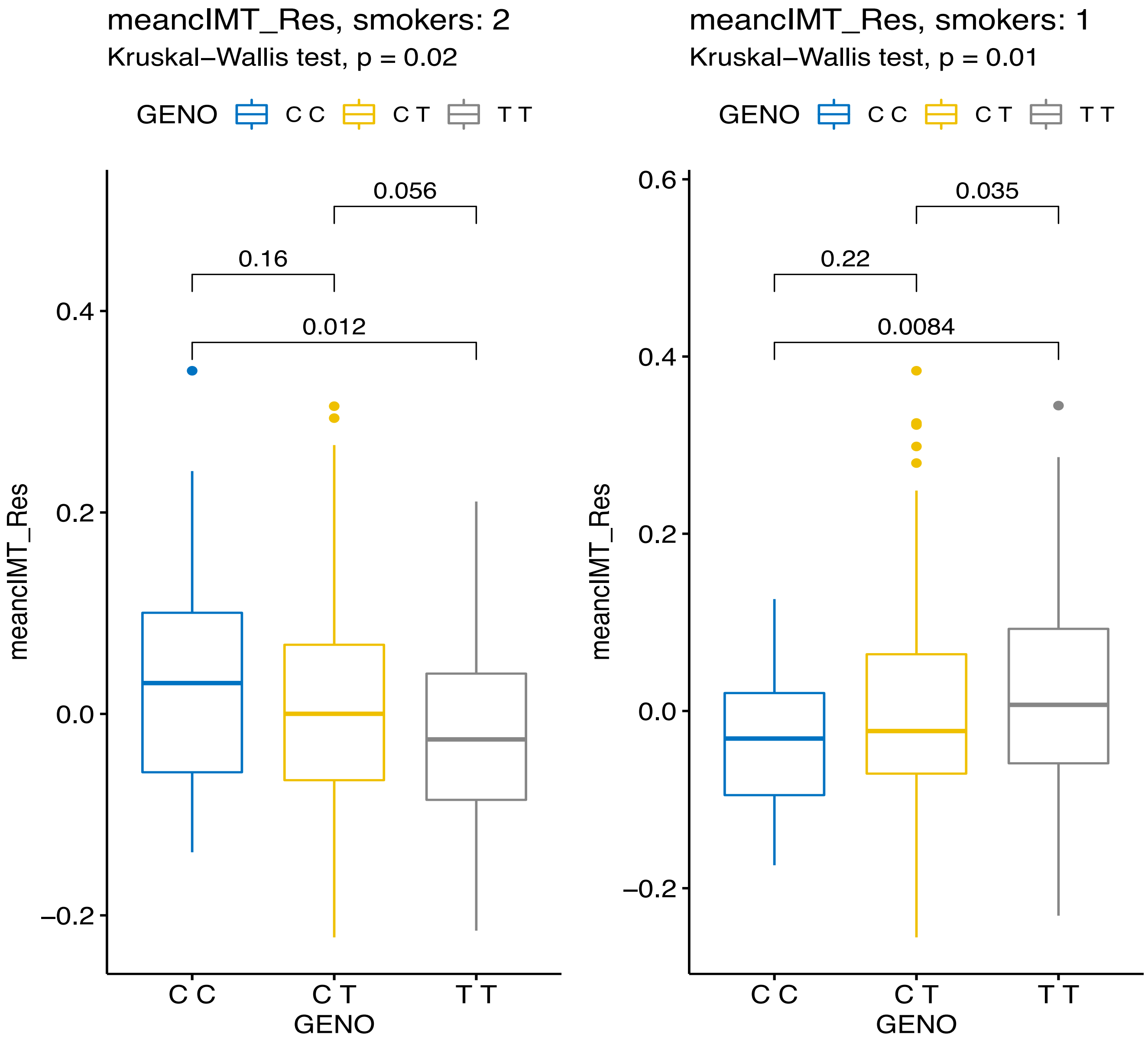

geno-rs4869800

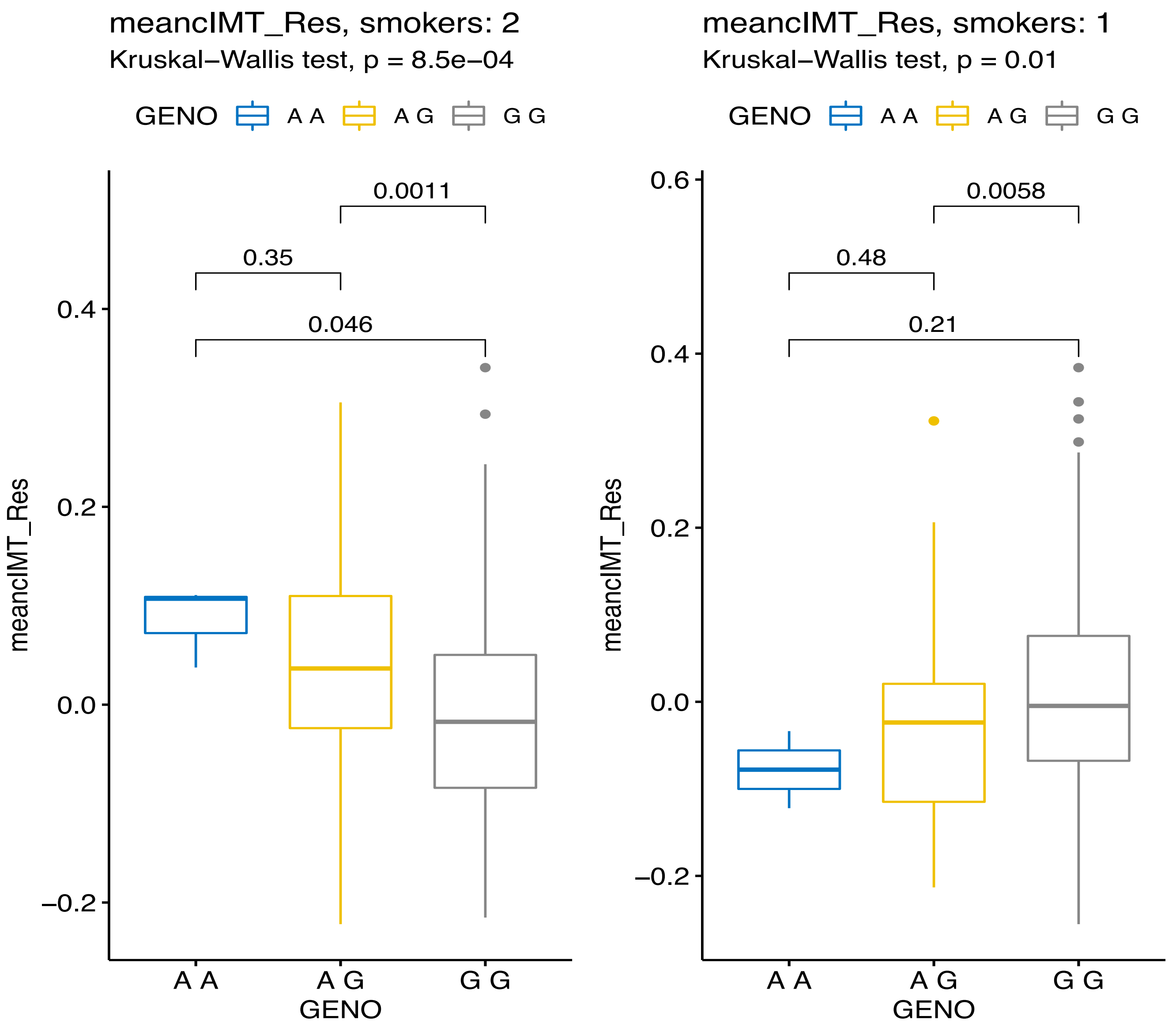

geno-rs7215170

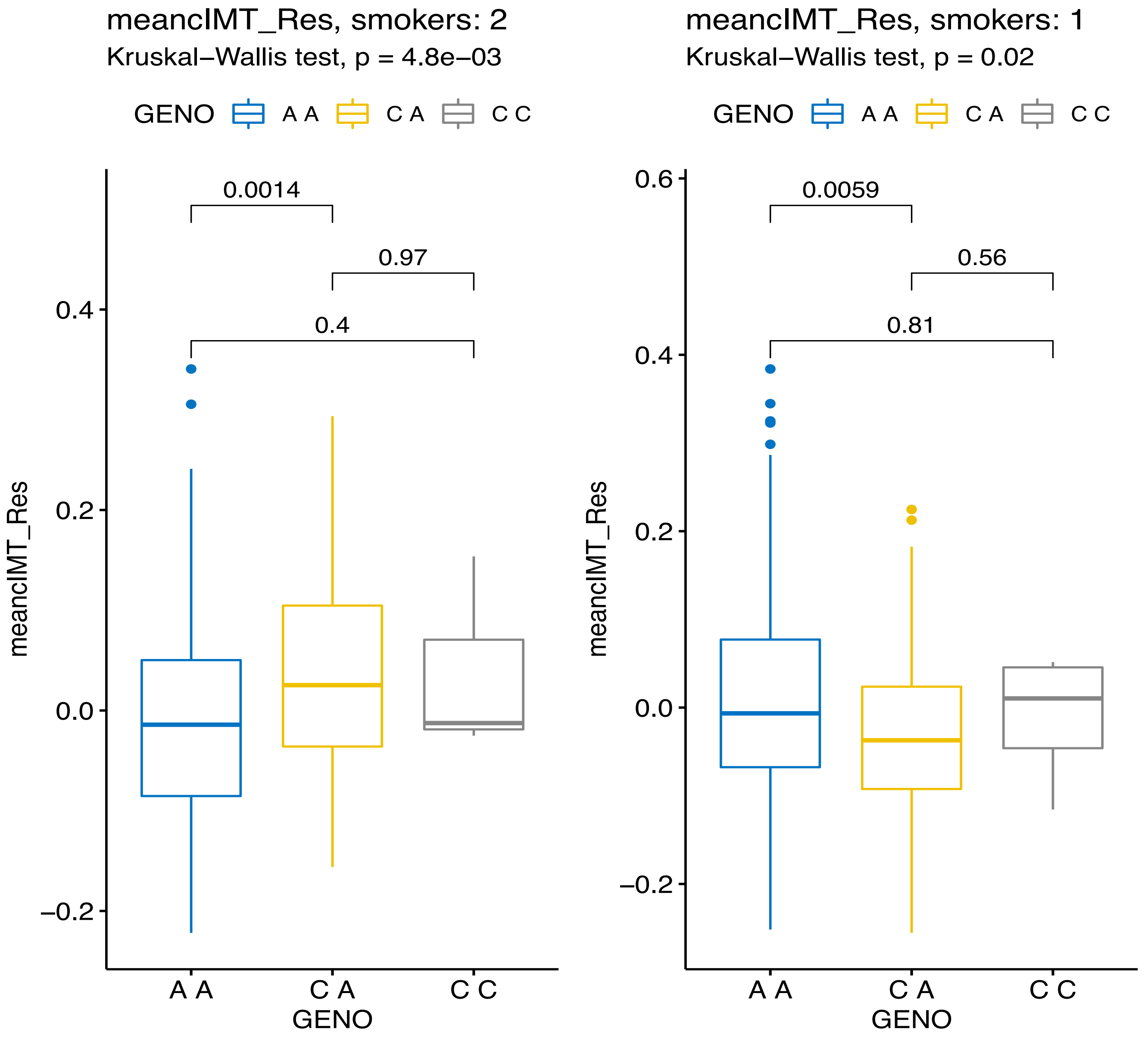

geno-rs7358623

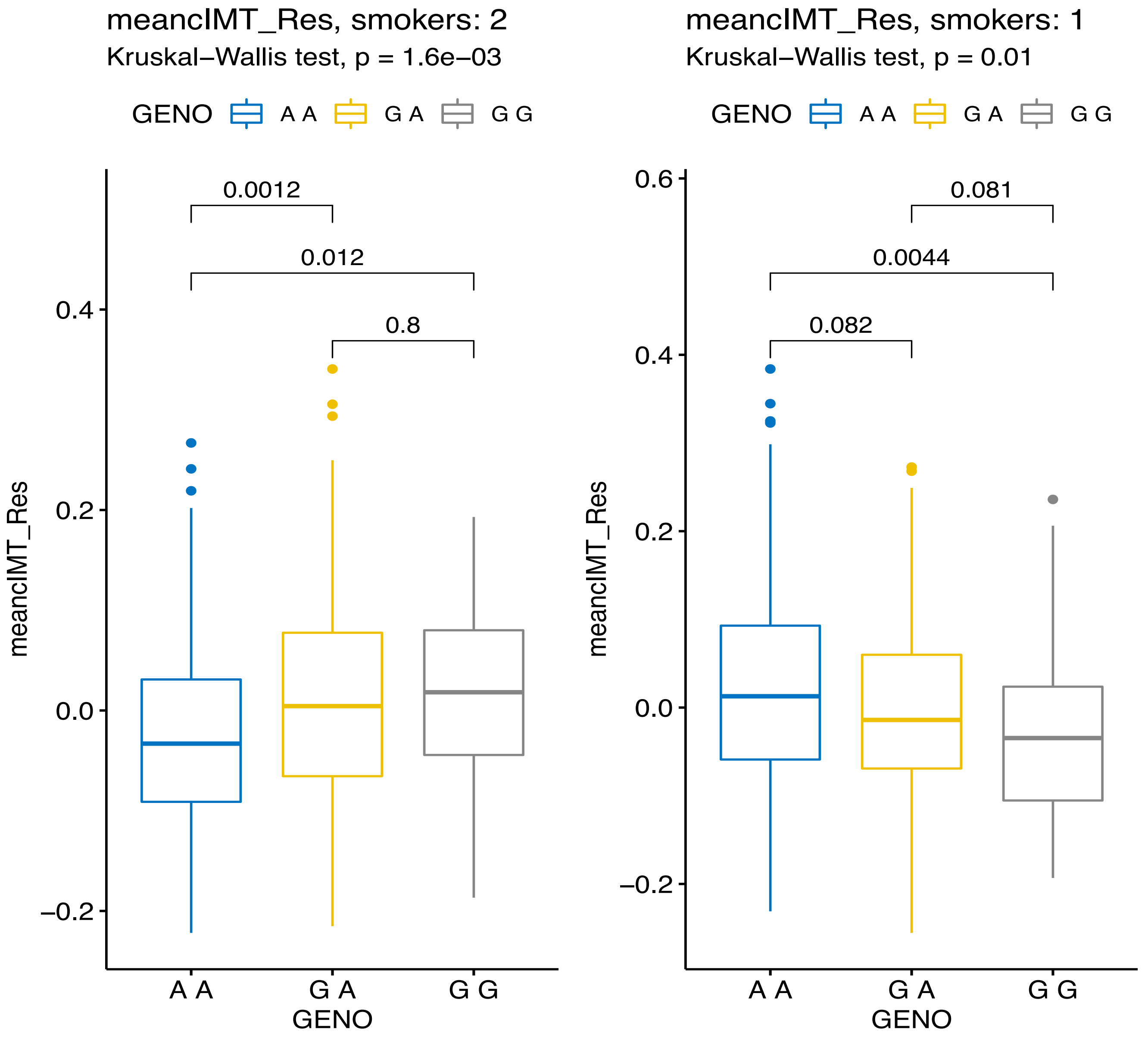

geno-rs10964835

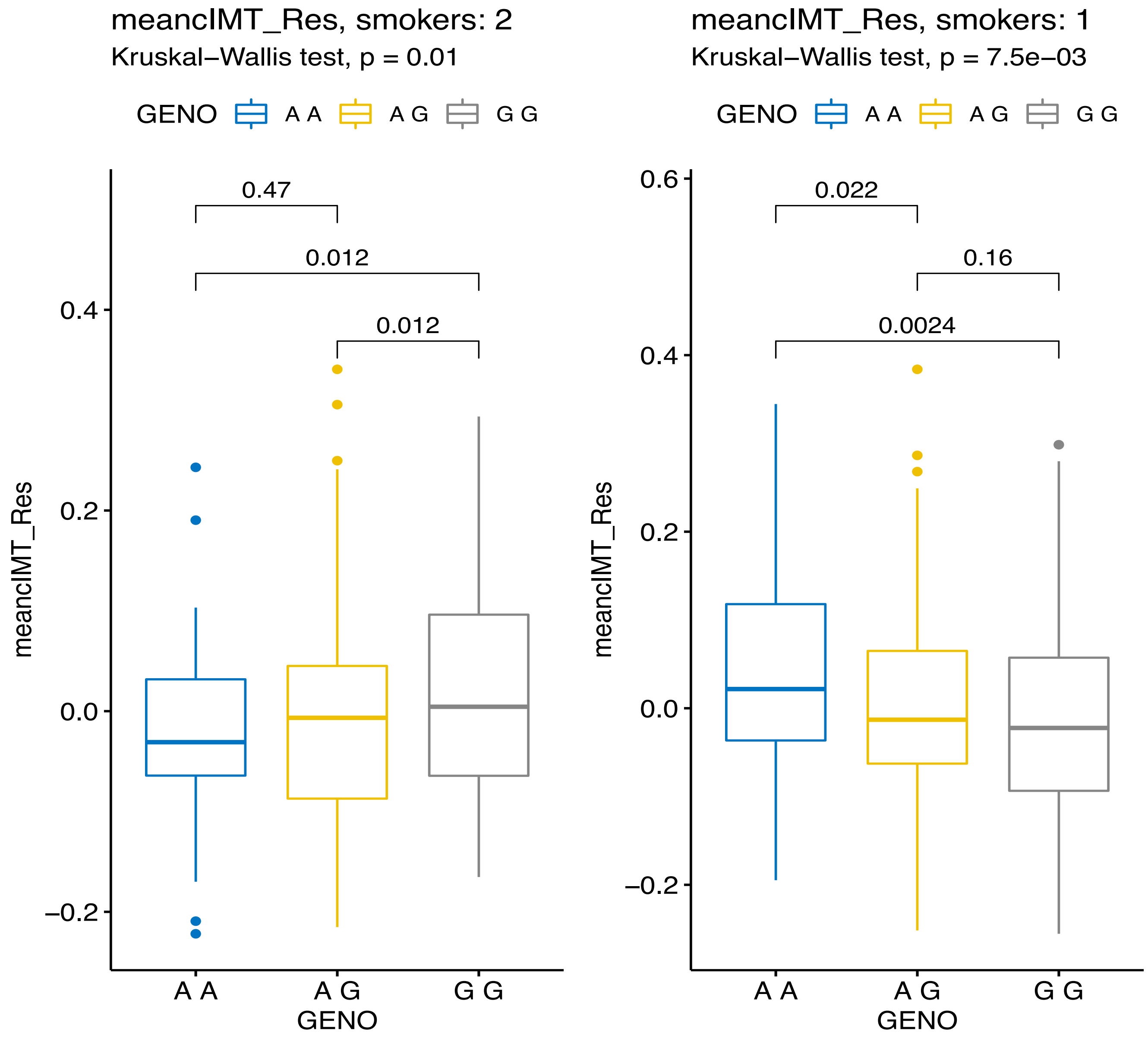

geno-rs11043125

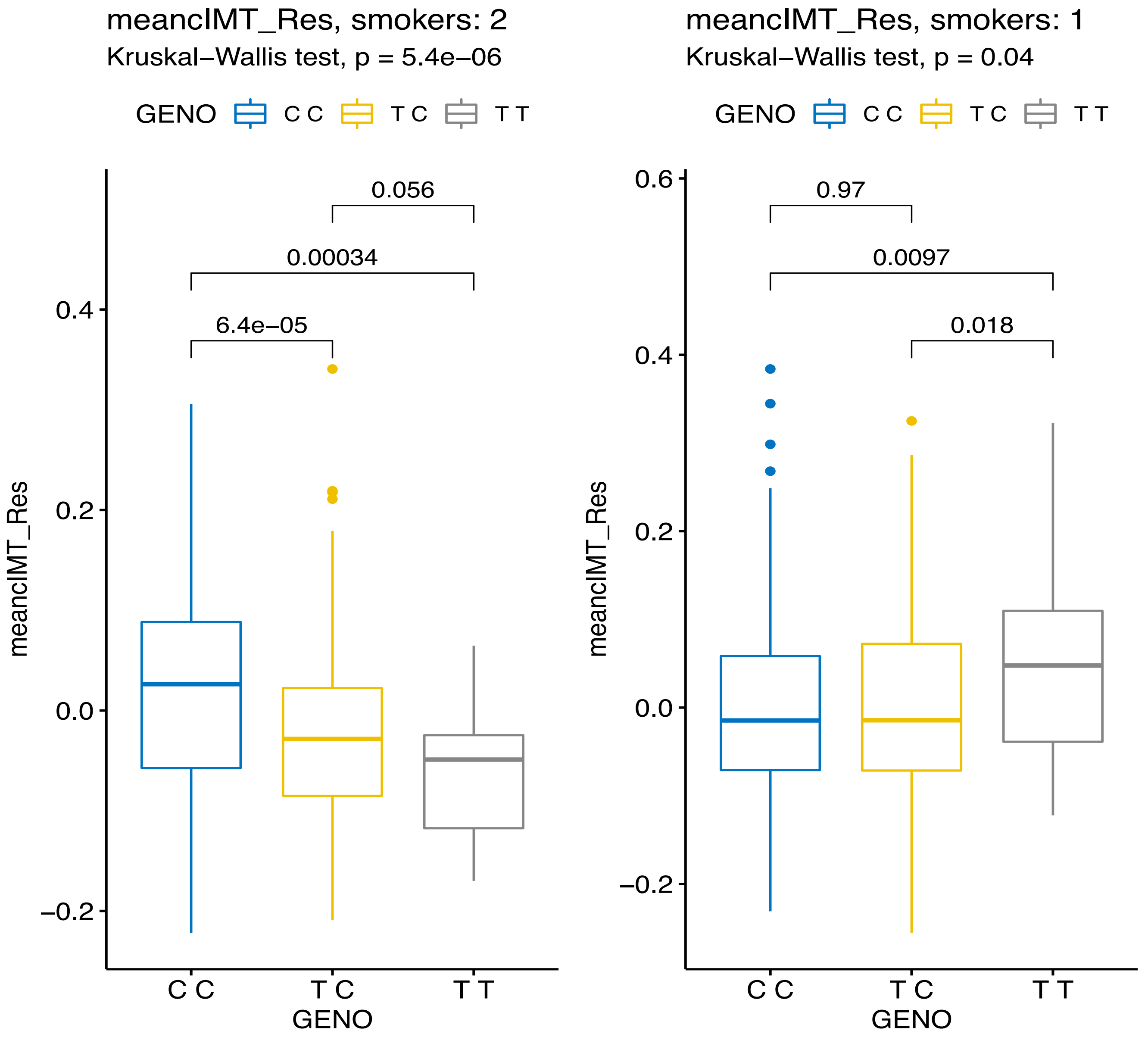

geno-rs11067175

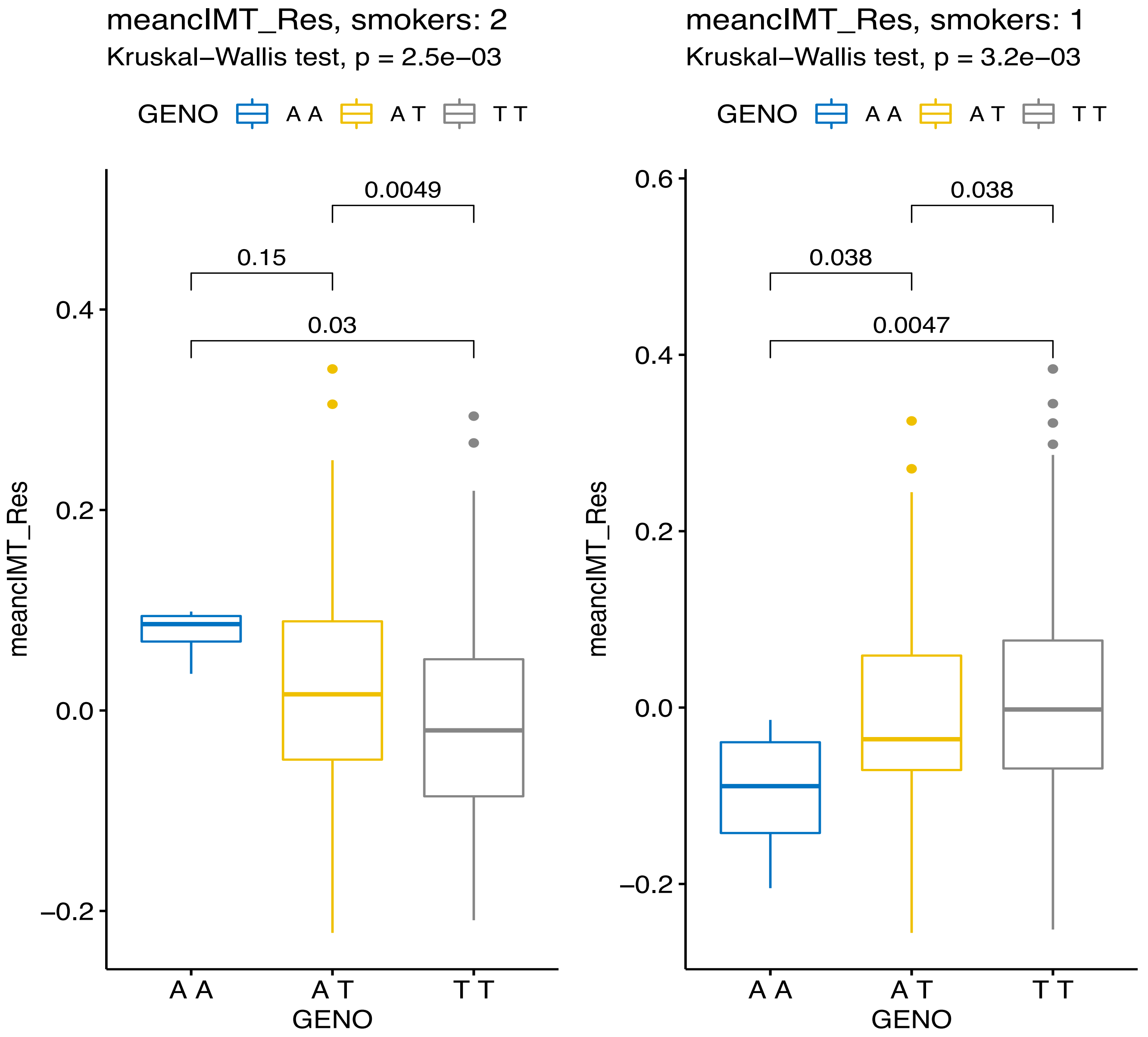

geno-rs11158732

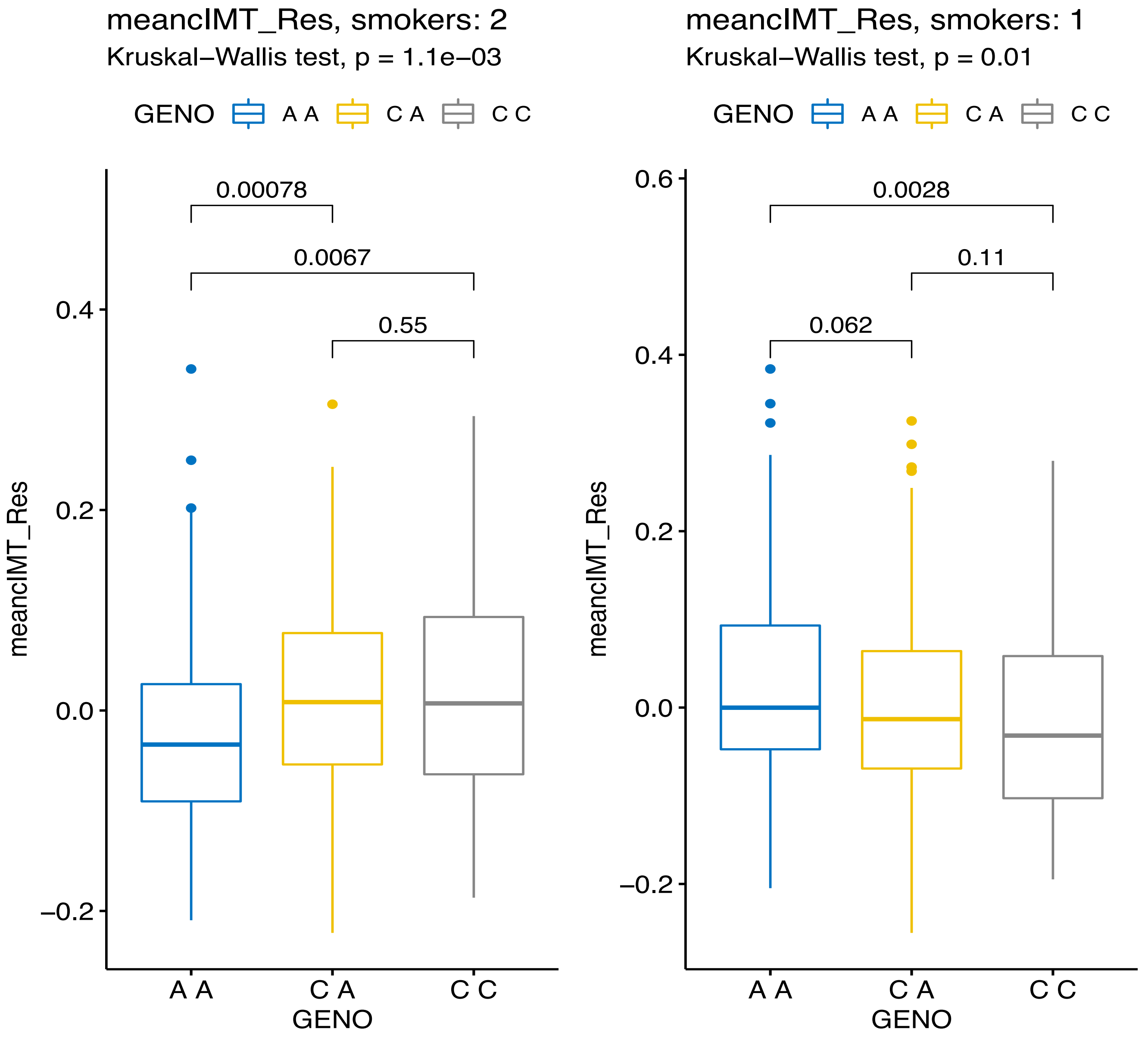

geno-rs11793060

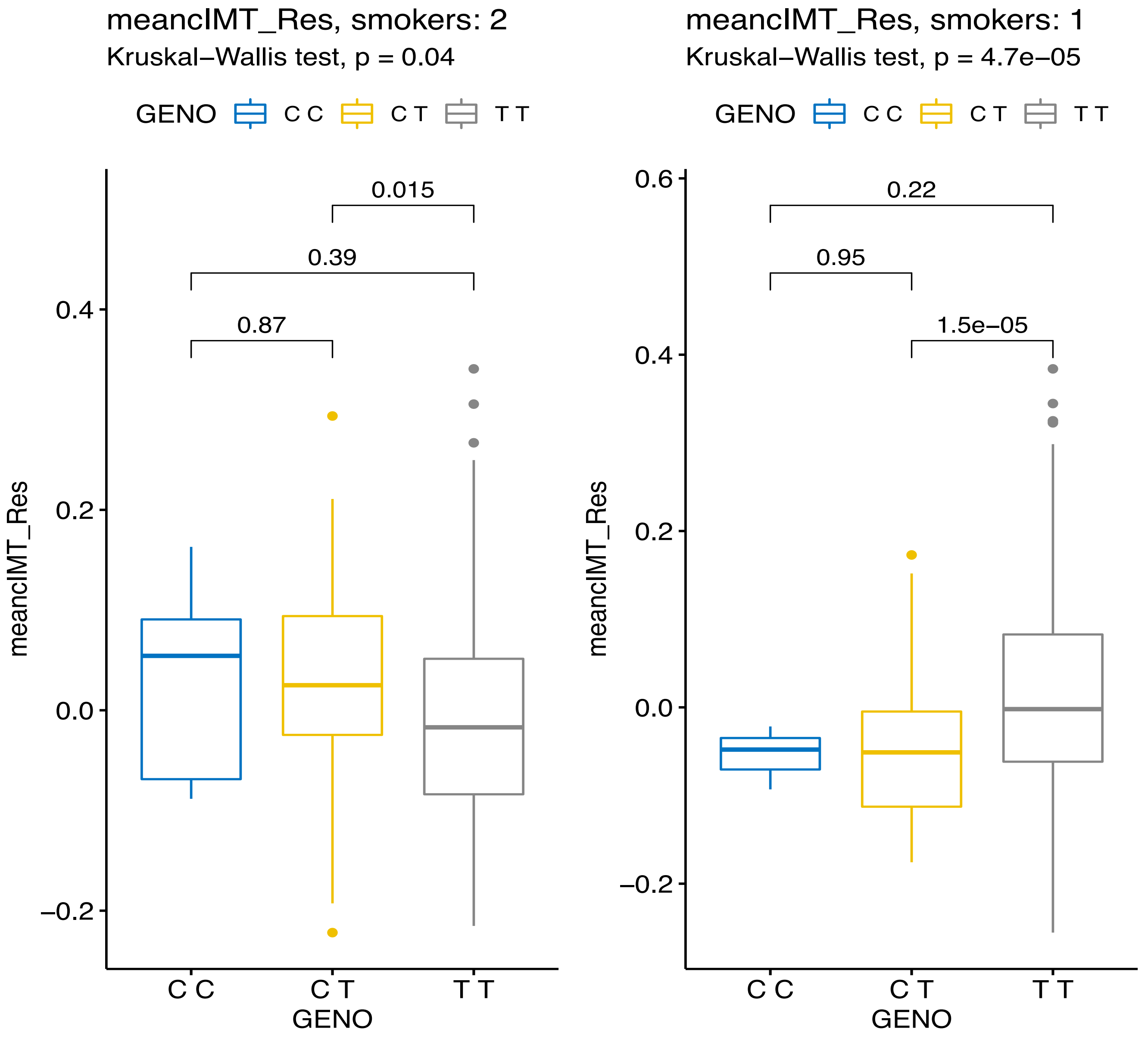

geno-rs12444312

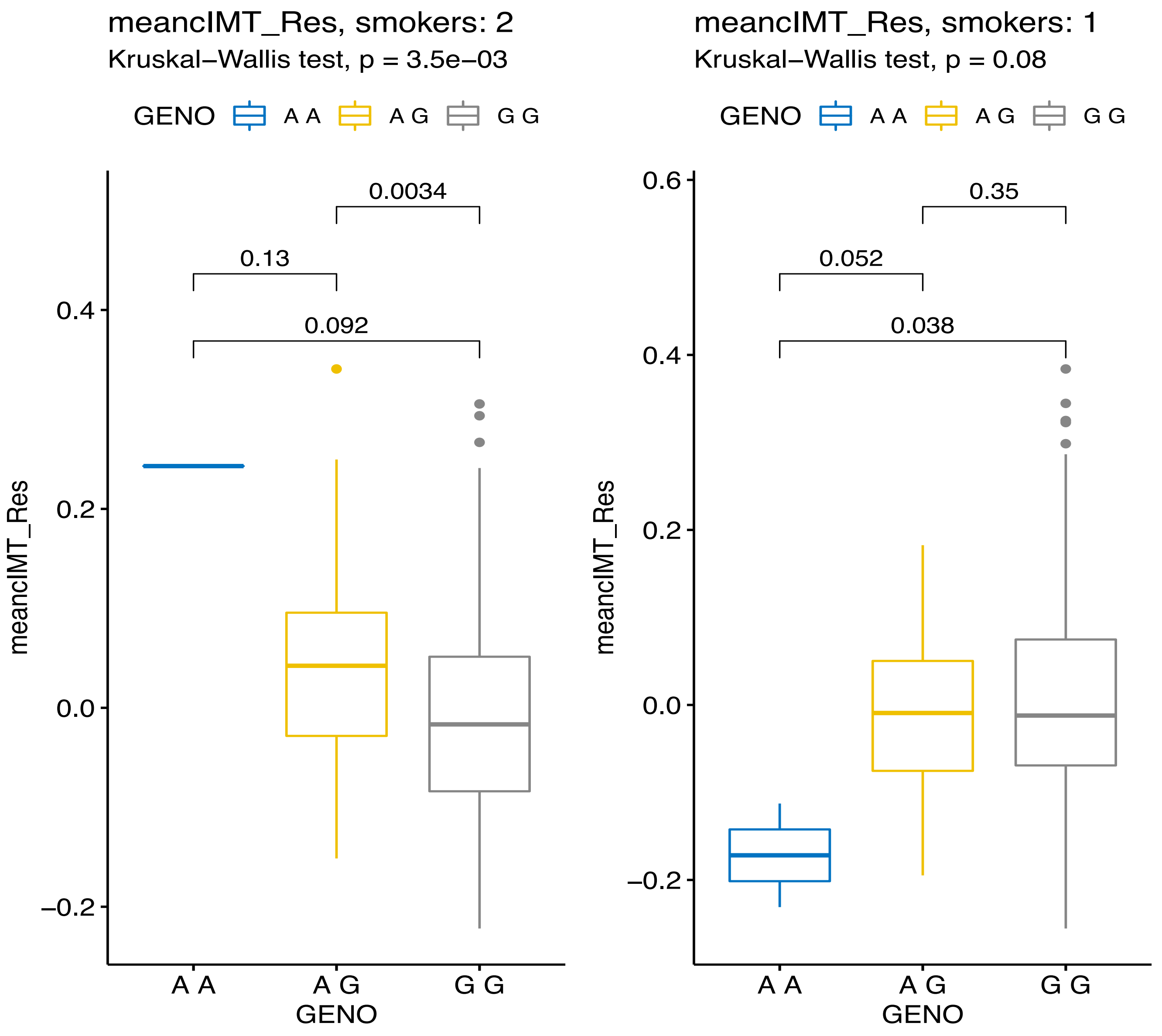

geno-rs12743305

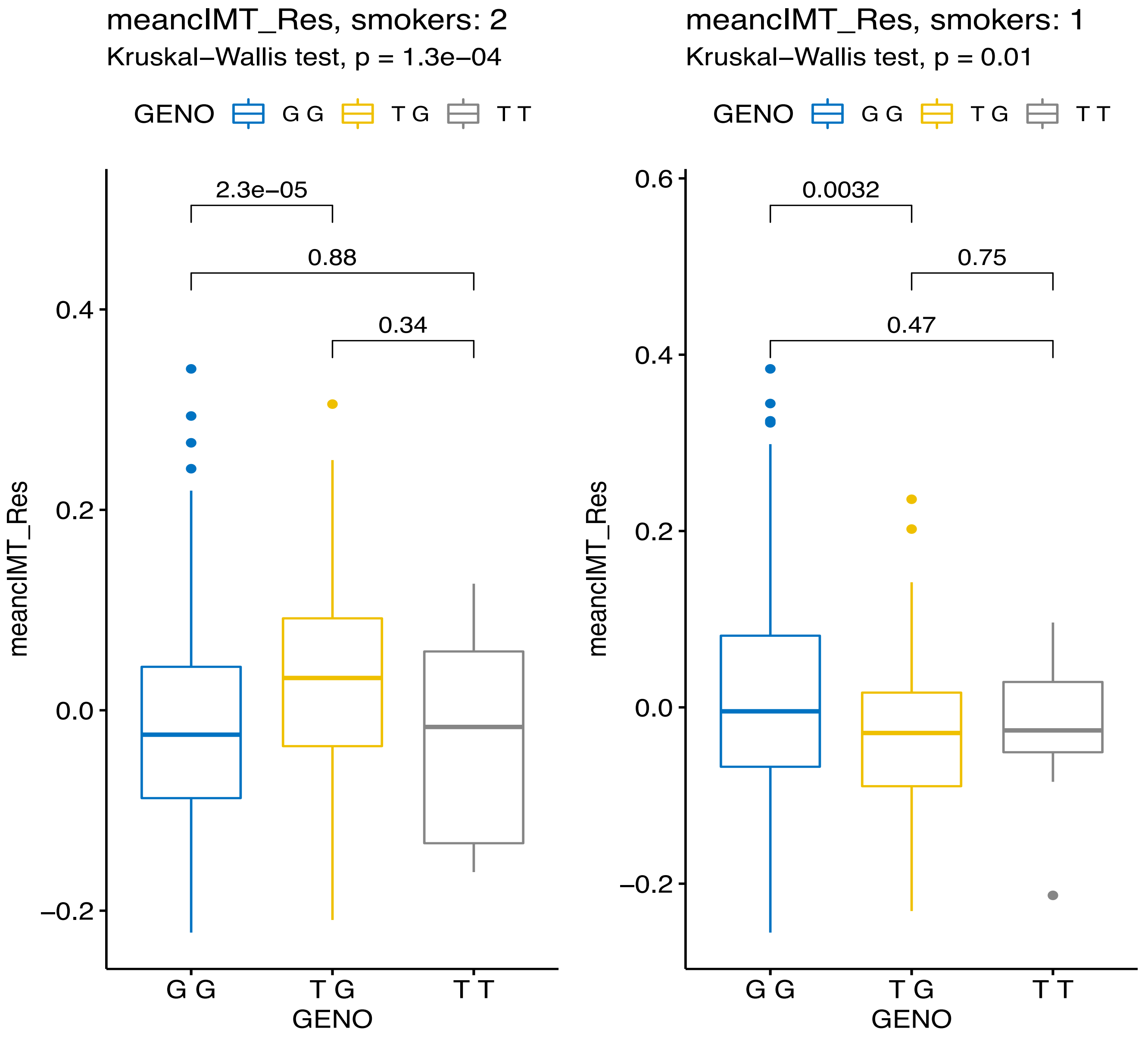

geno-rs28695976

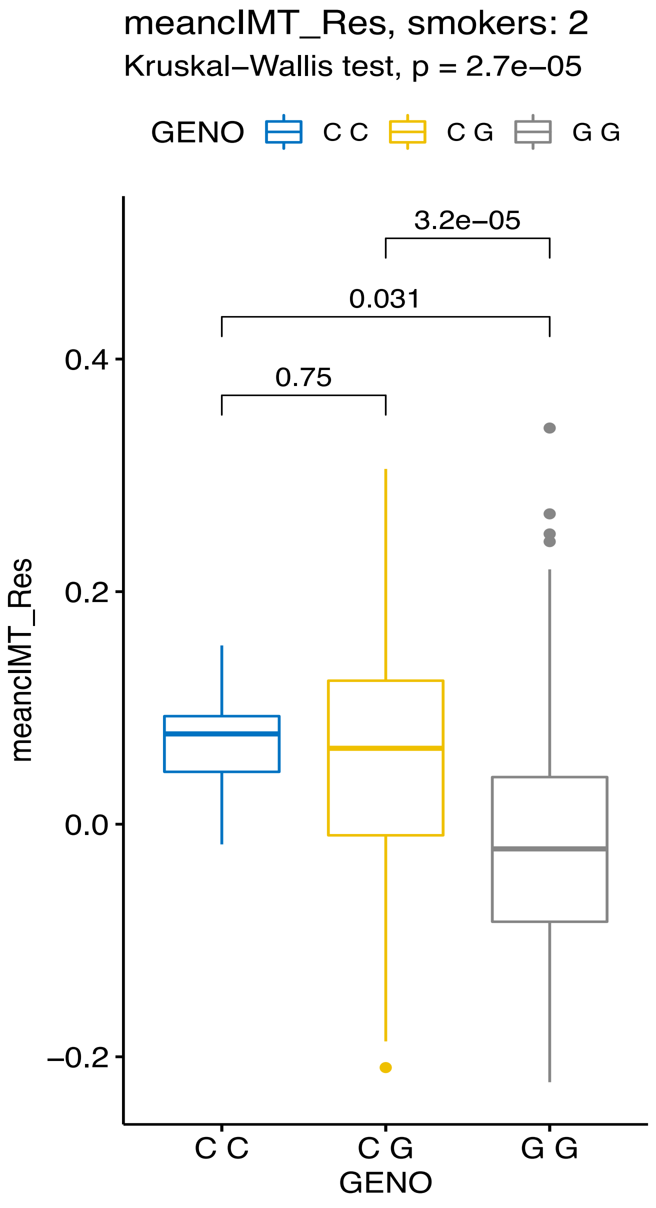

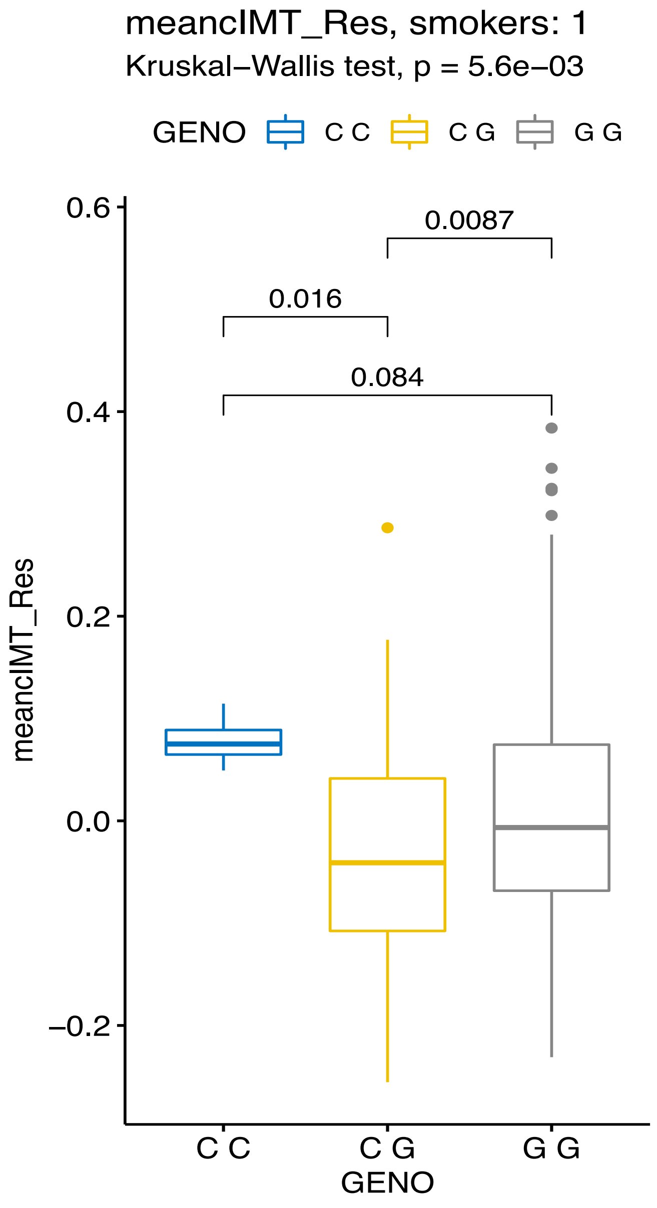

geno-rs58190827.pdf

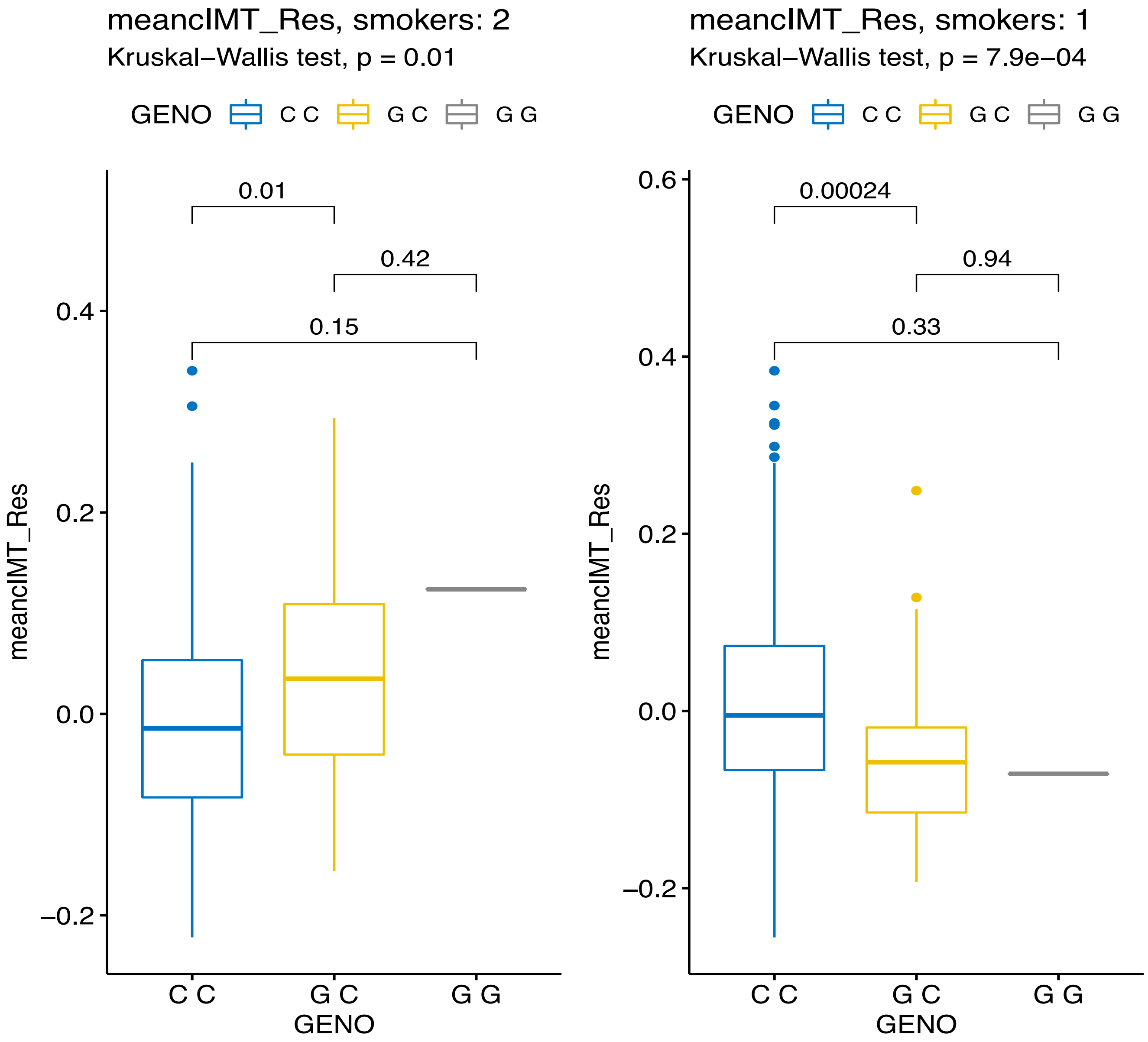

geno-rs116720528

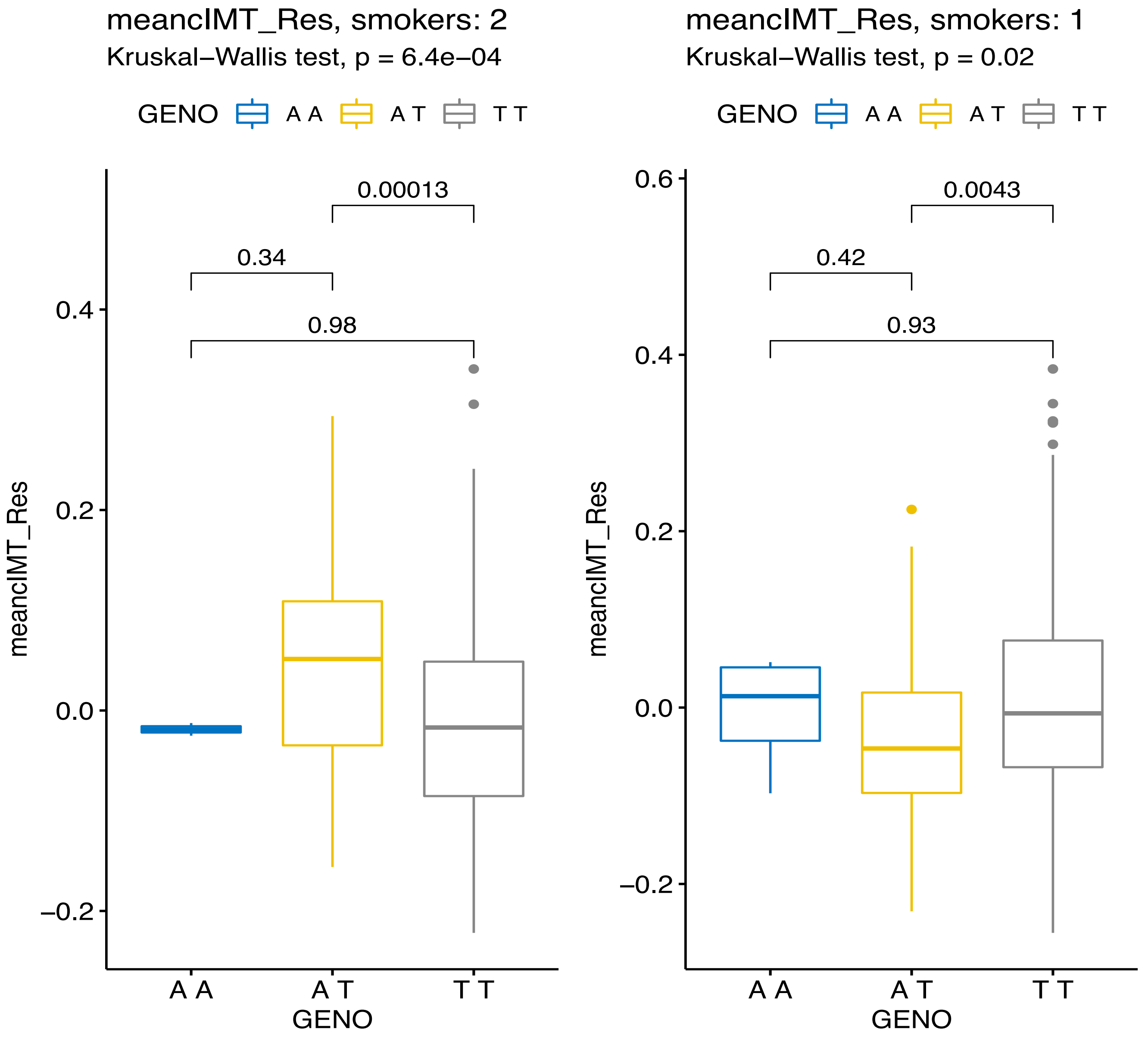

Supplementary Figure 2c:

Genotypes plots of selected SNPs (p-values<1E-05) in the combined sample, showing distibutions of mean cIMT residuals in somkers and non-smokers groups

geno-rs1192824

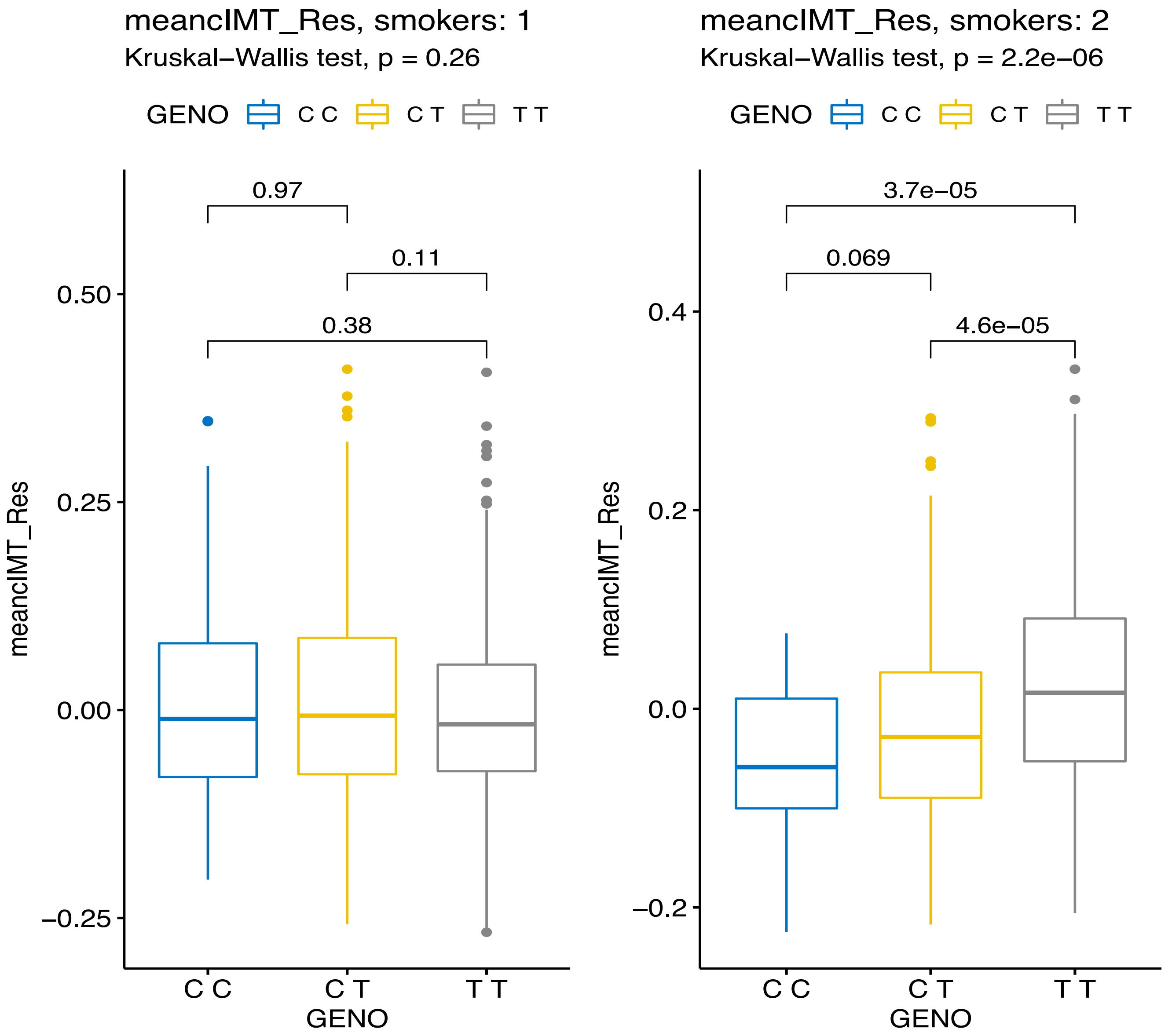

geno-rs1546993

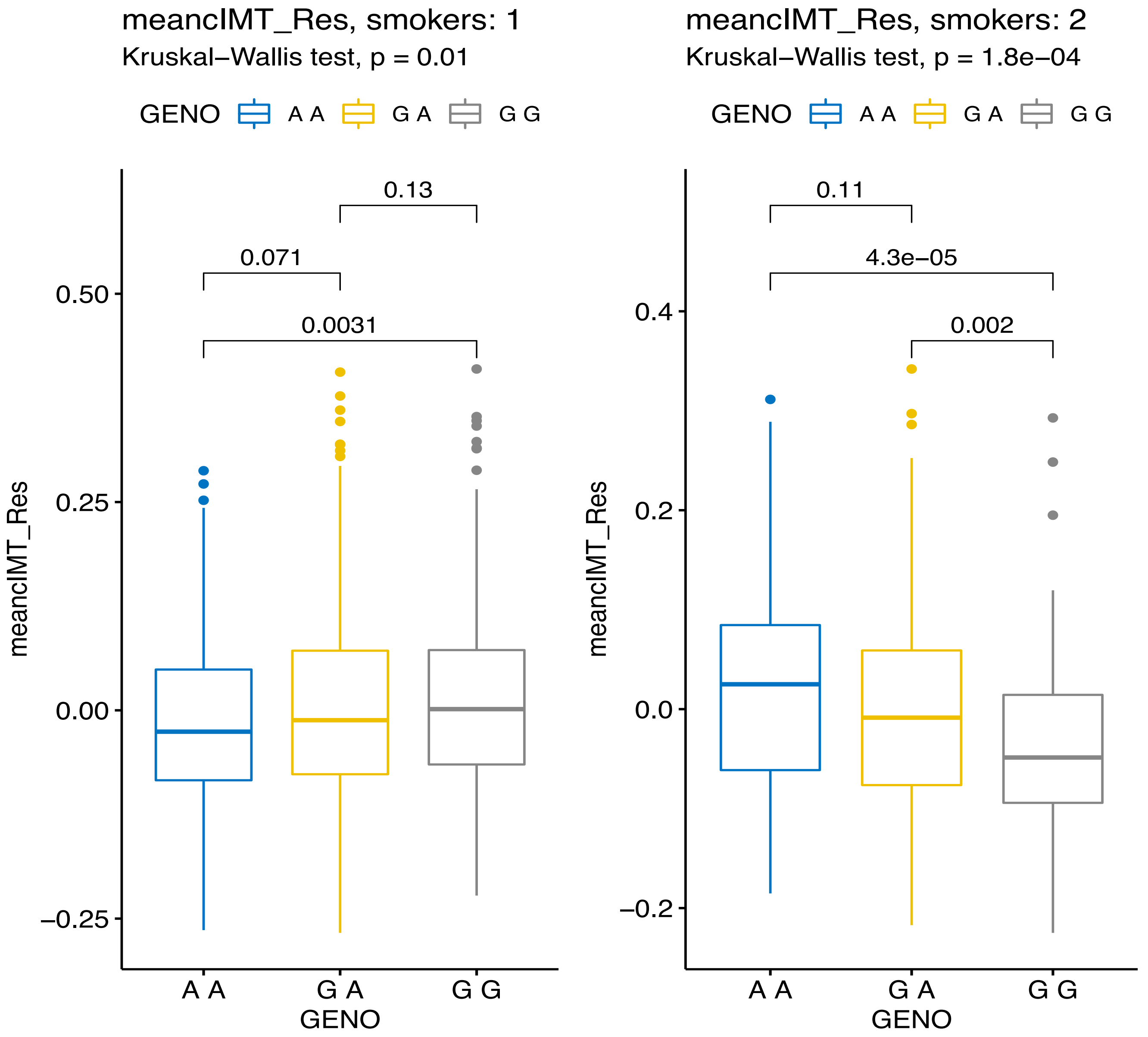

geno-rs1939309

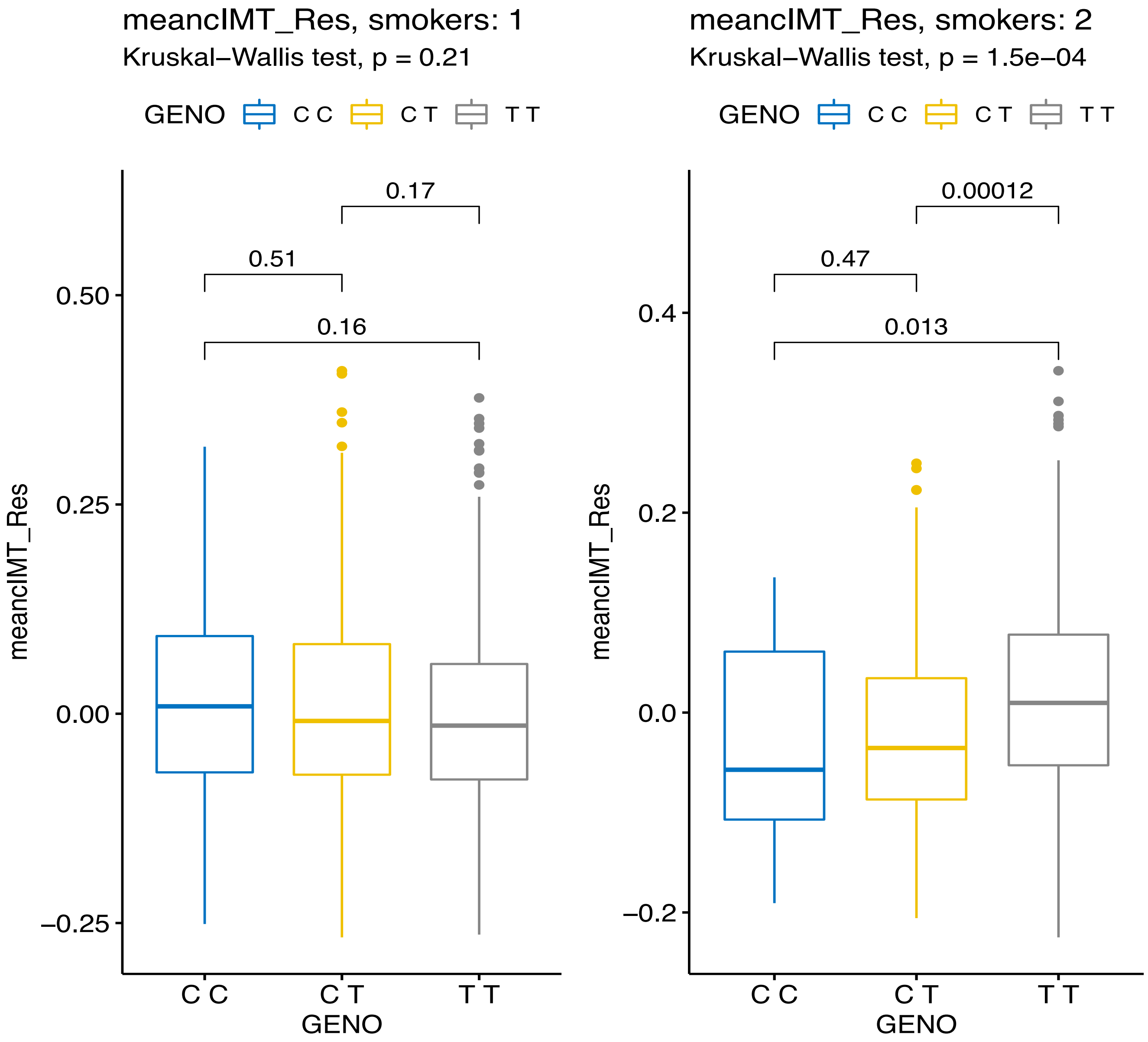

geno-rs4791039

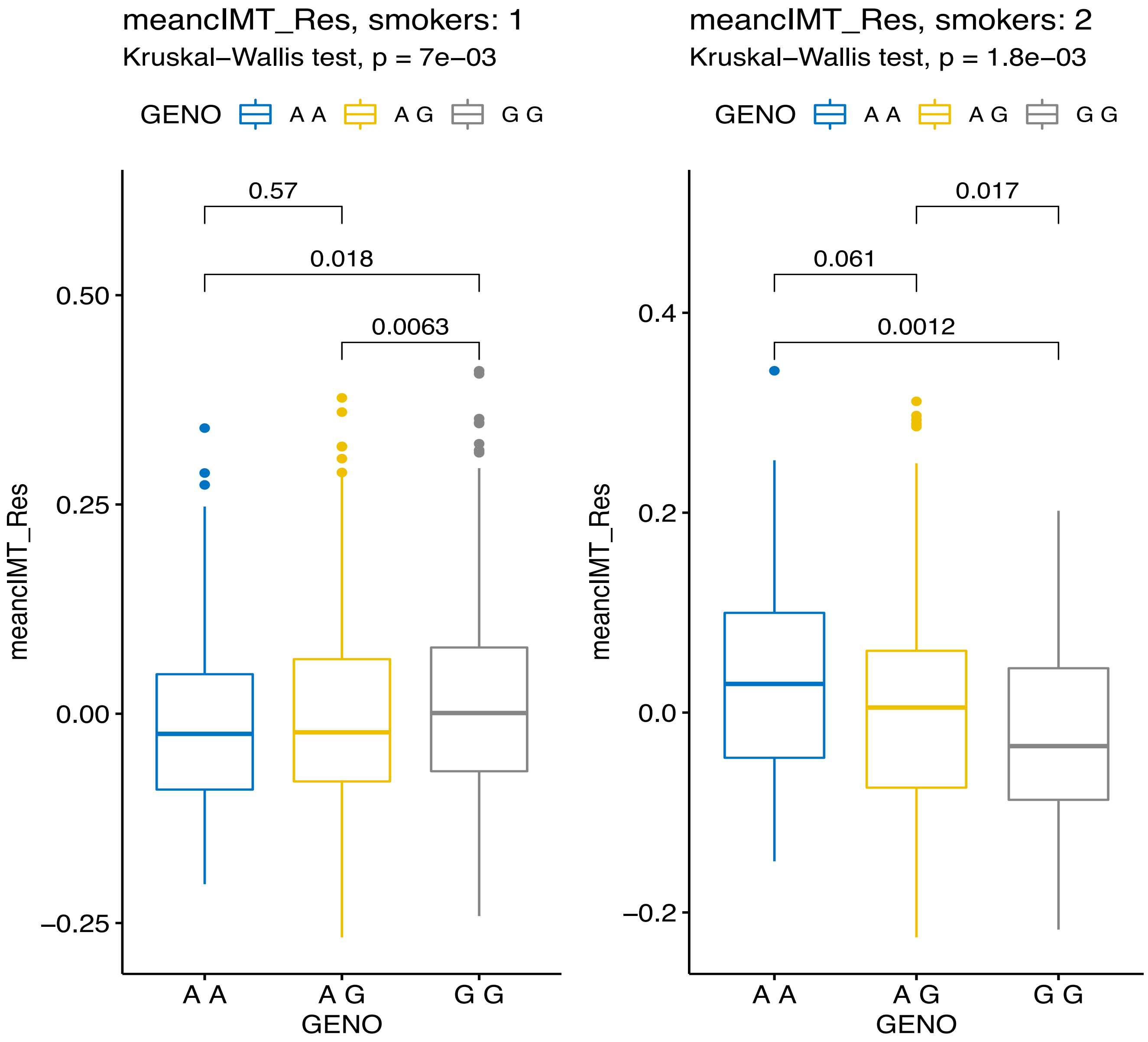

geno-rs6685095

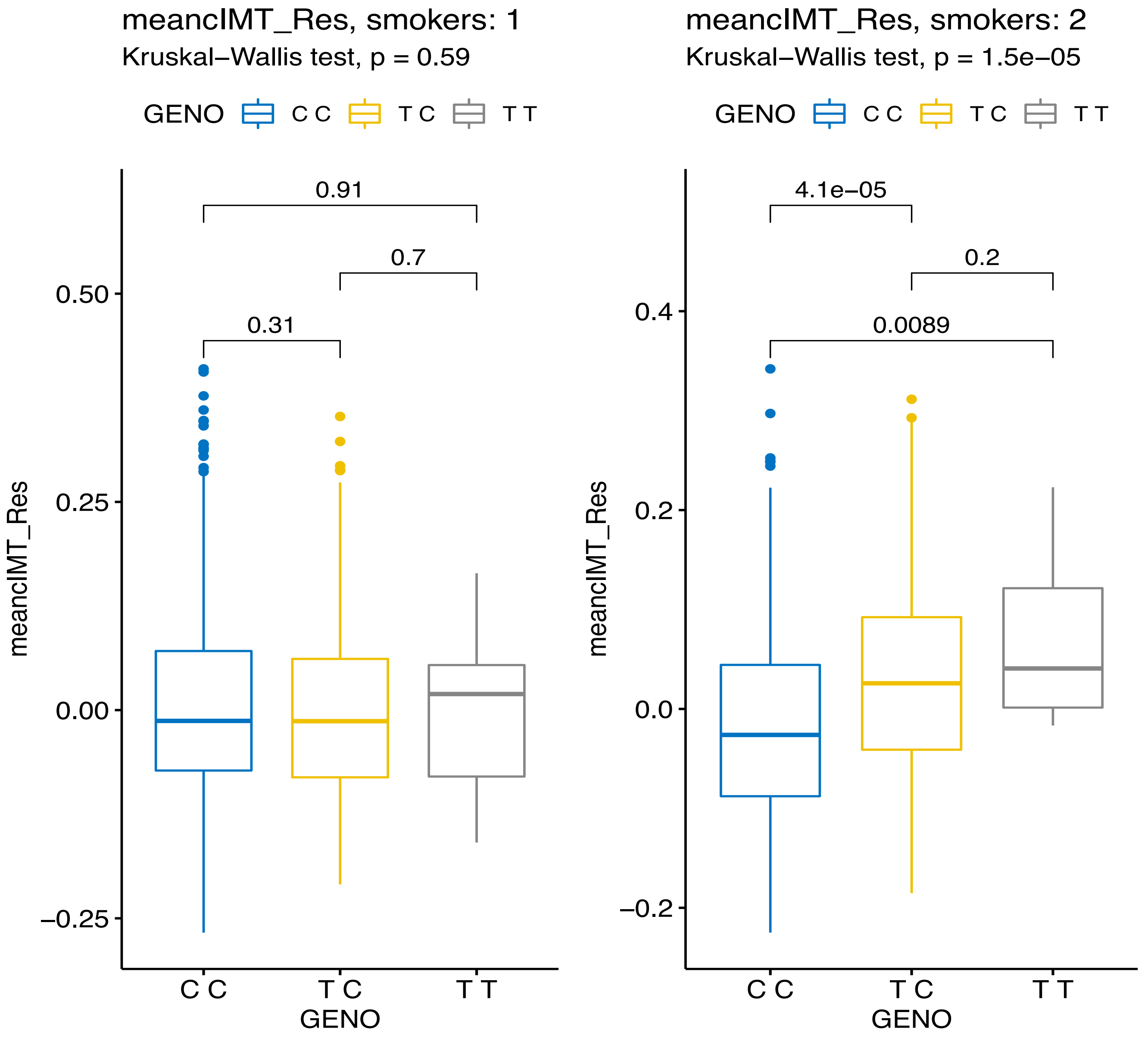

geno-rs9531489

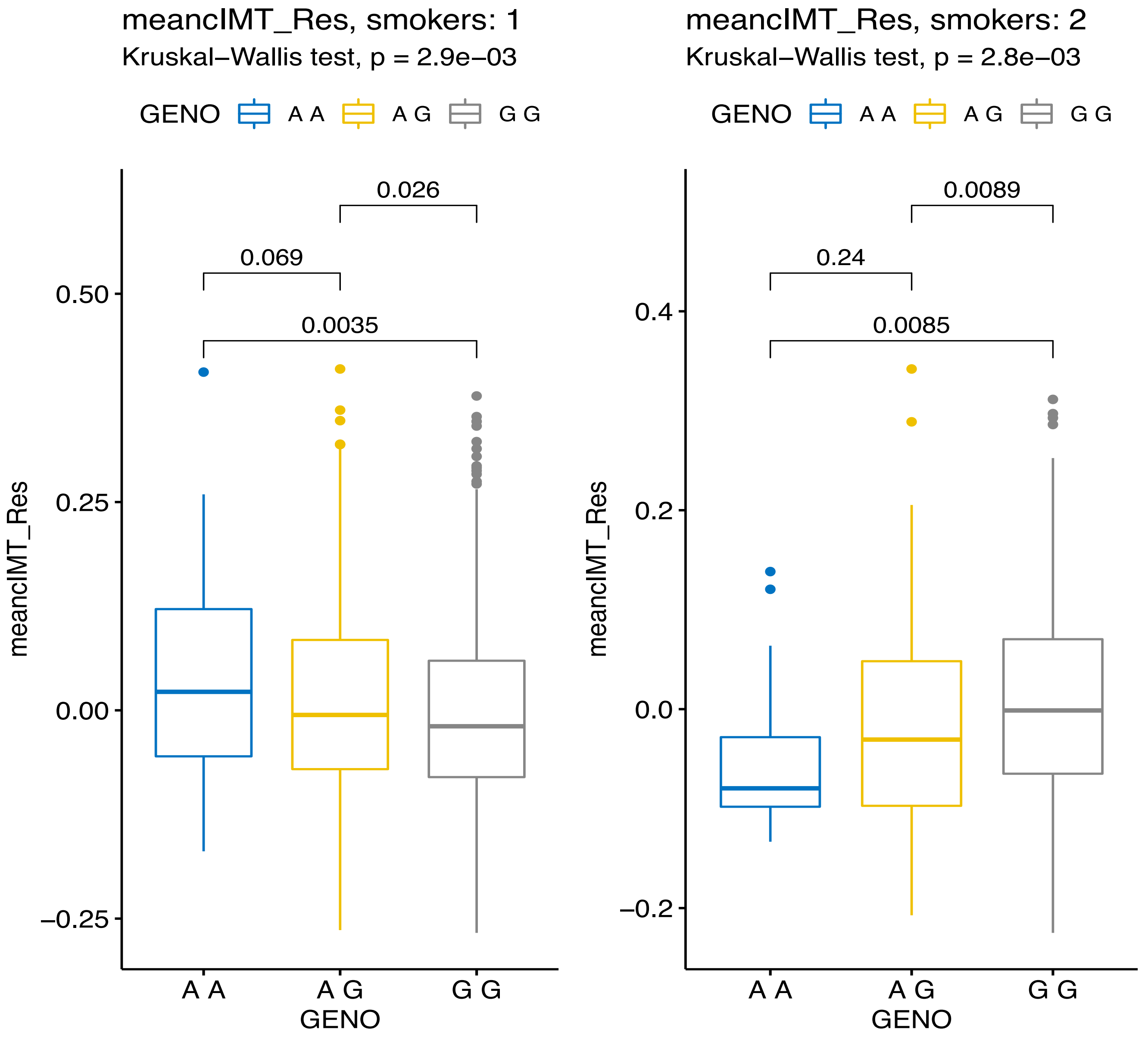

geno-rs9546479

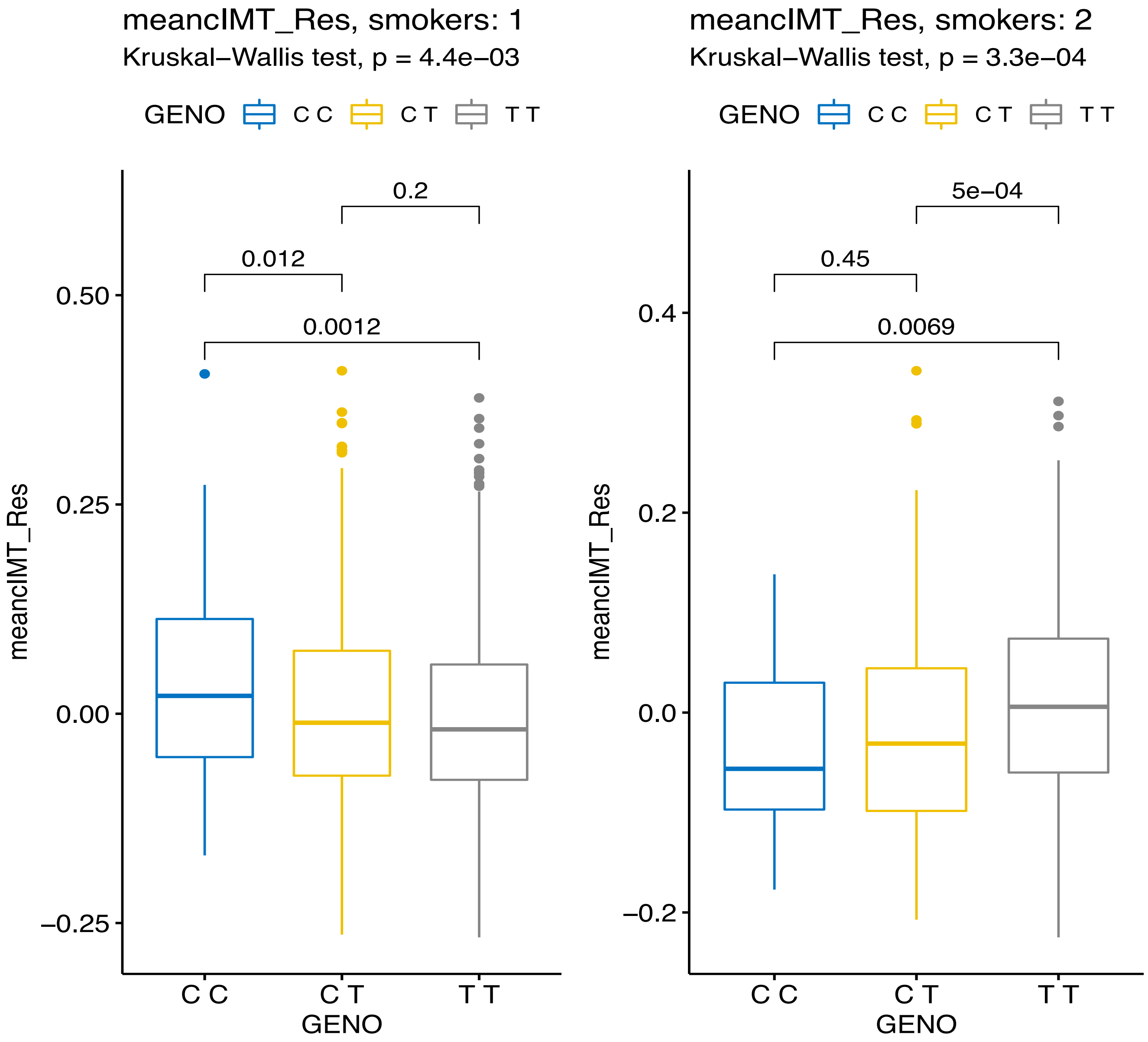

geno-rs9974620

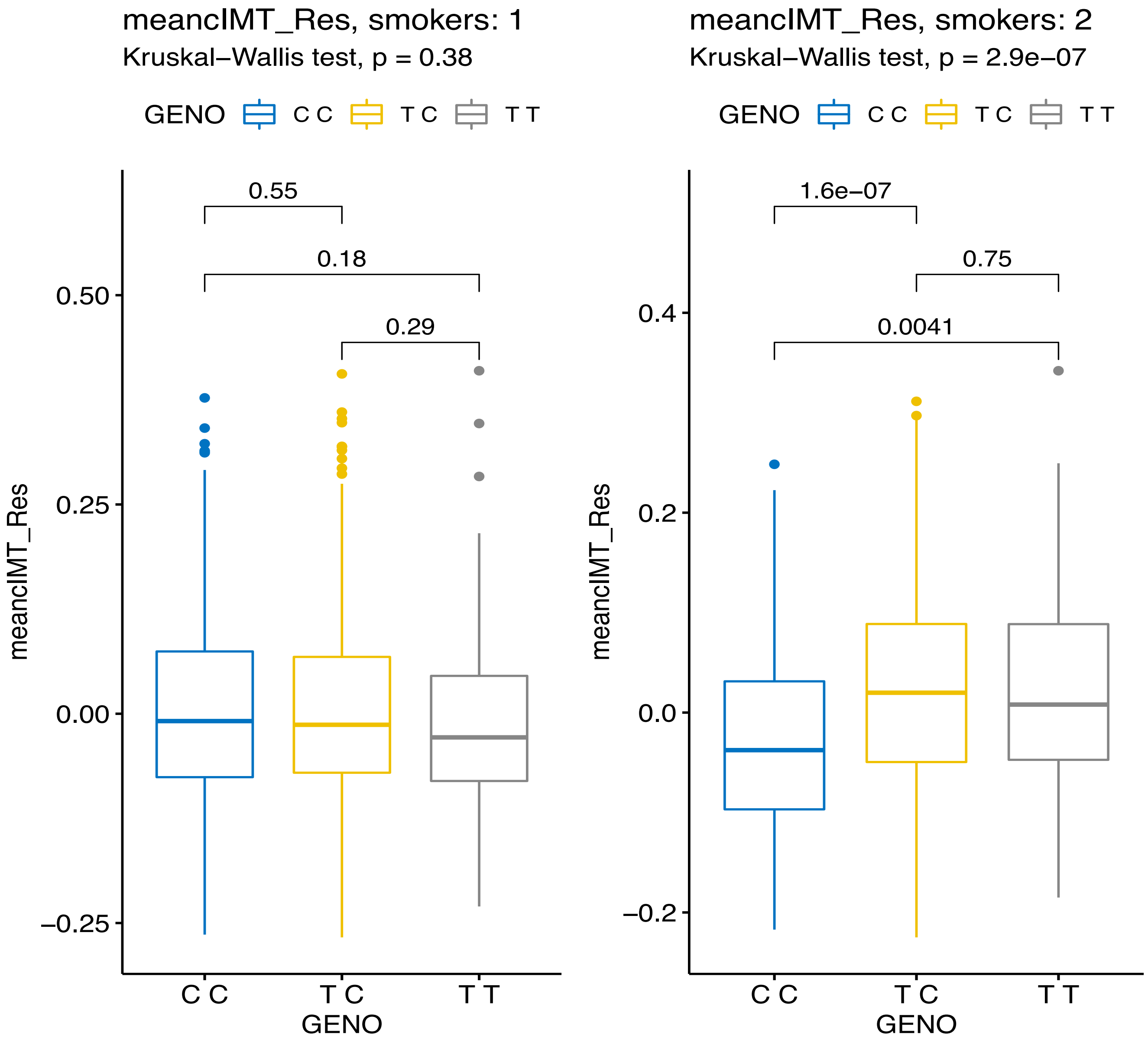

geno-rs11695675

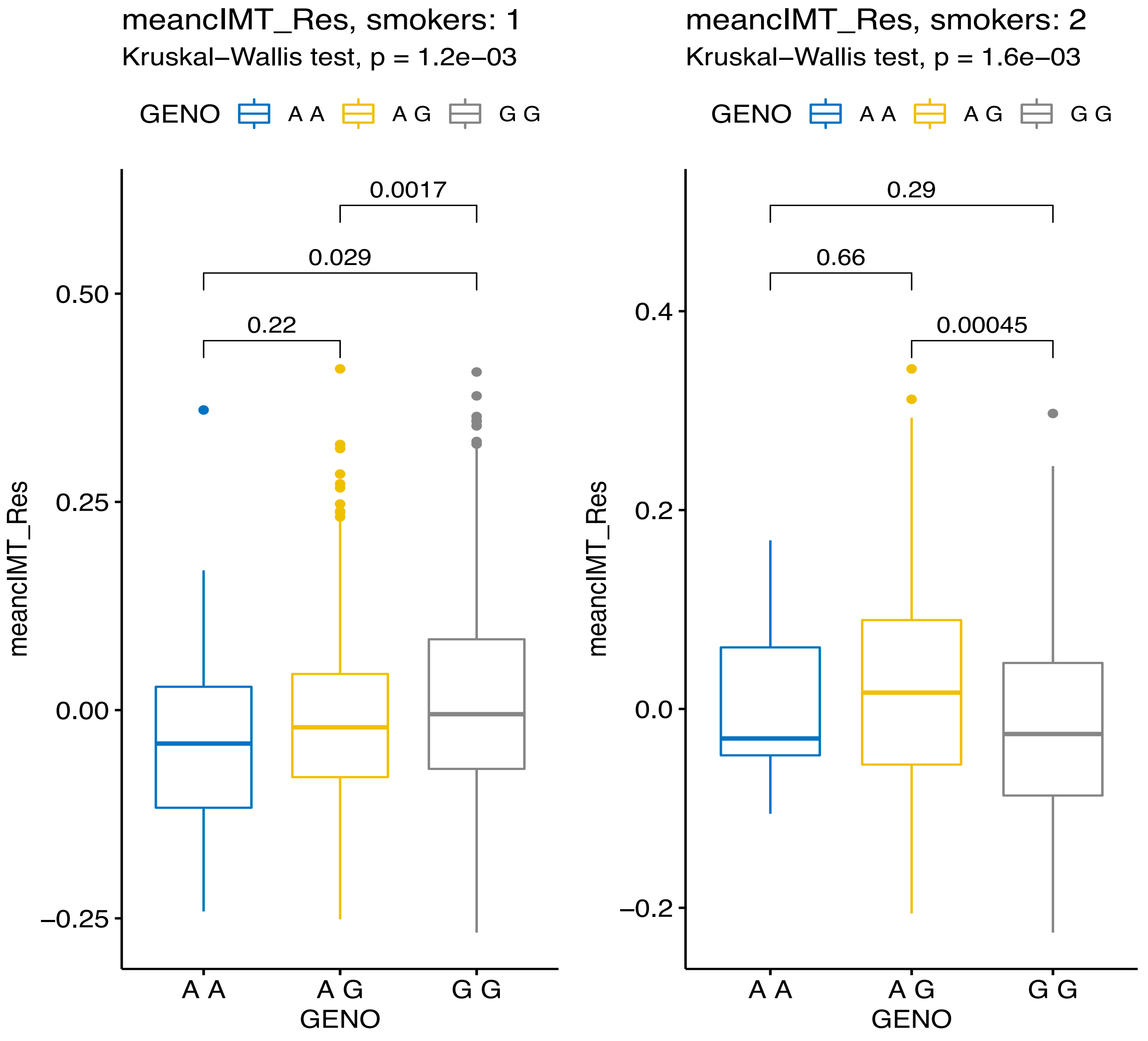

geno-rs12444312

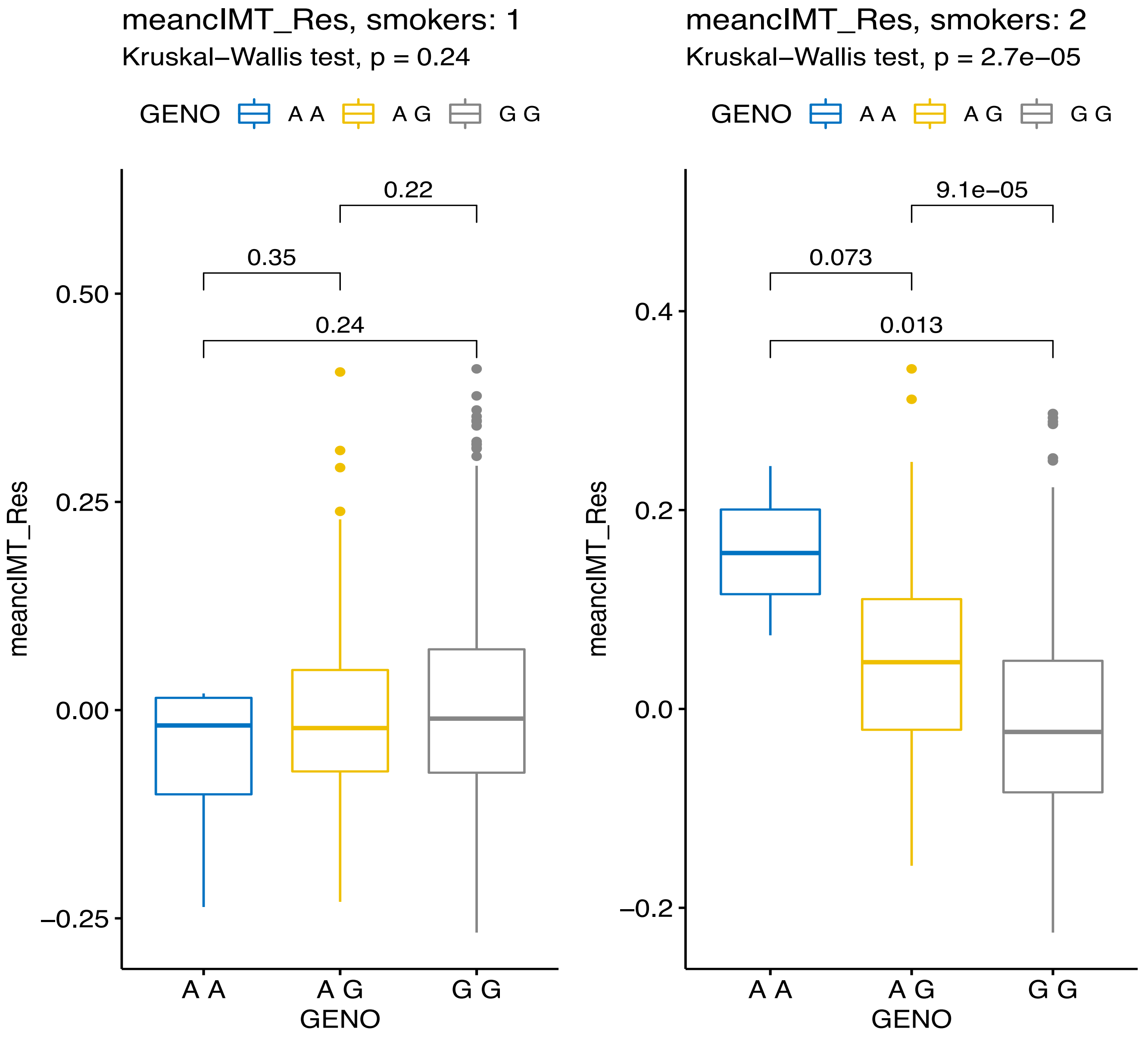

geno-rs62475193

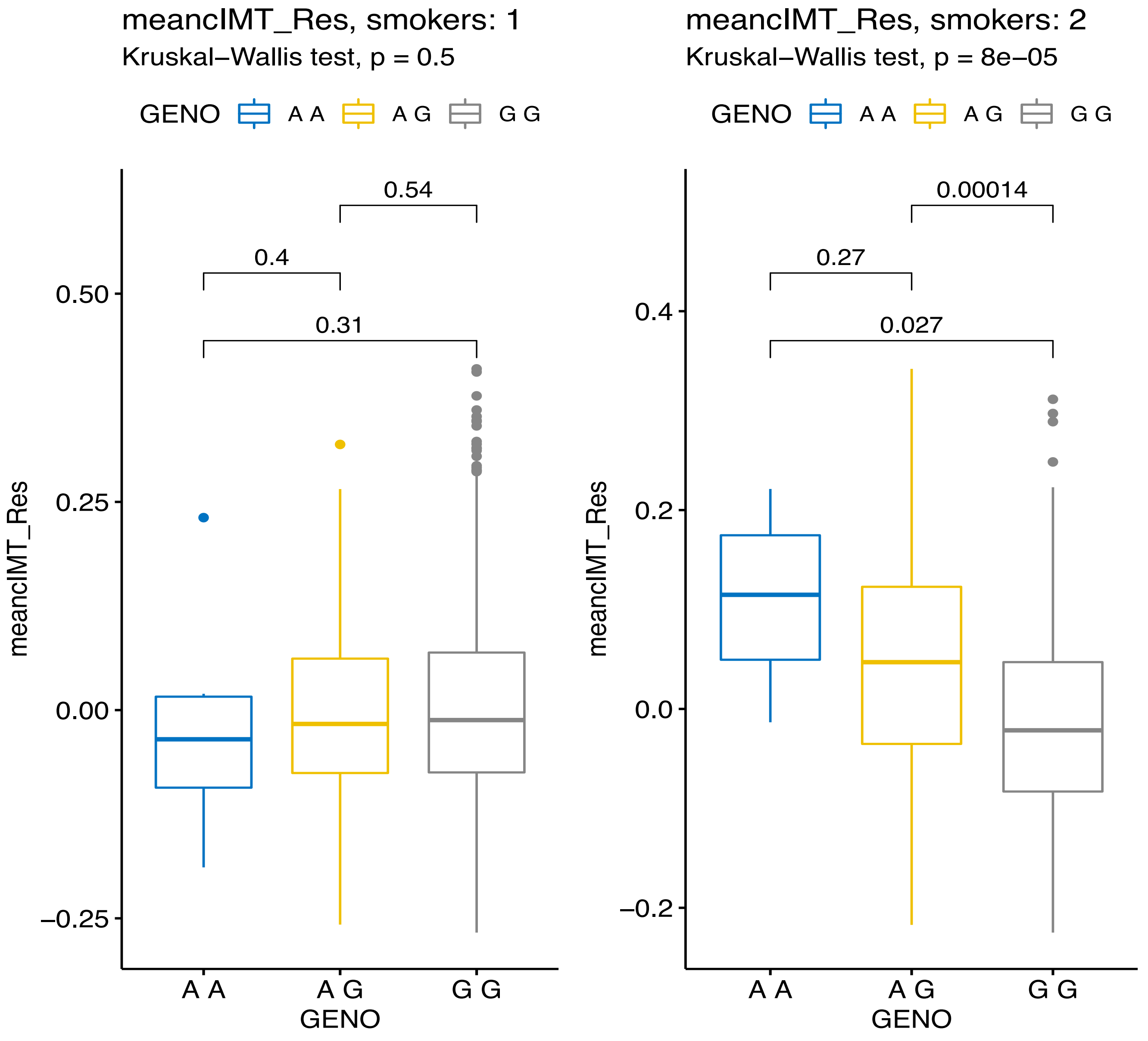

geno-rs74509340

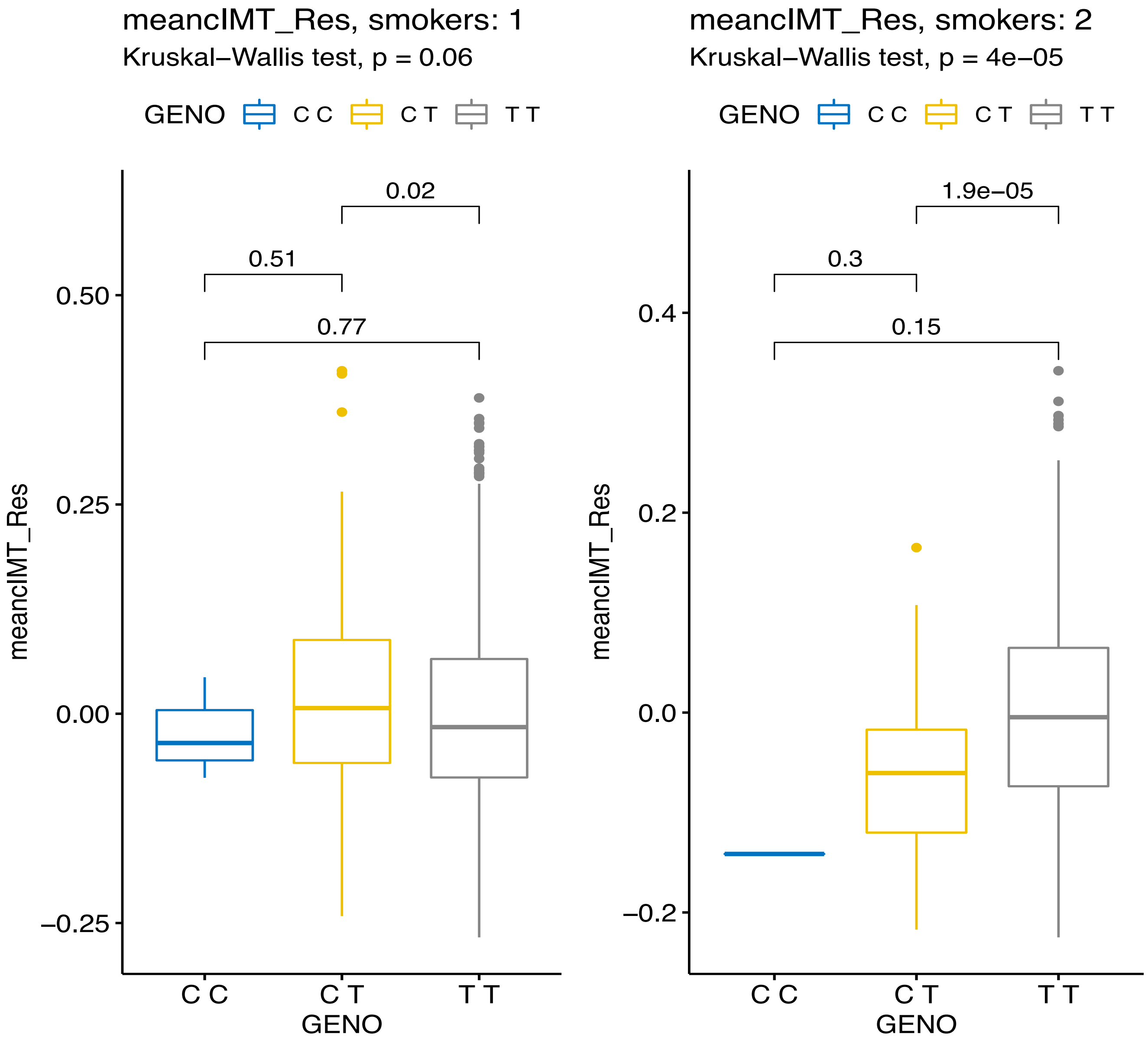

geno-rs74668330

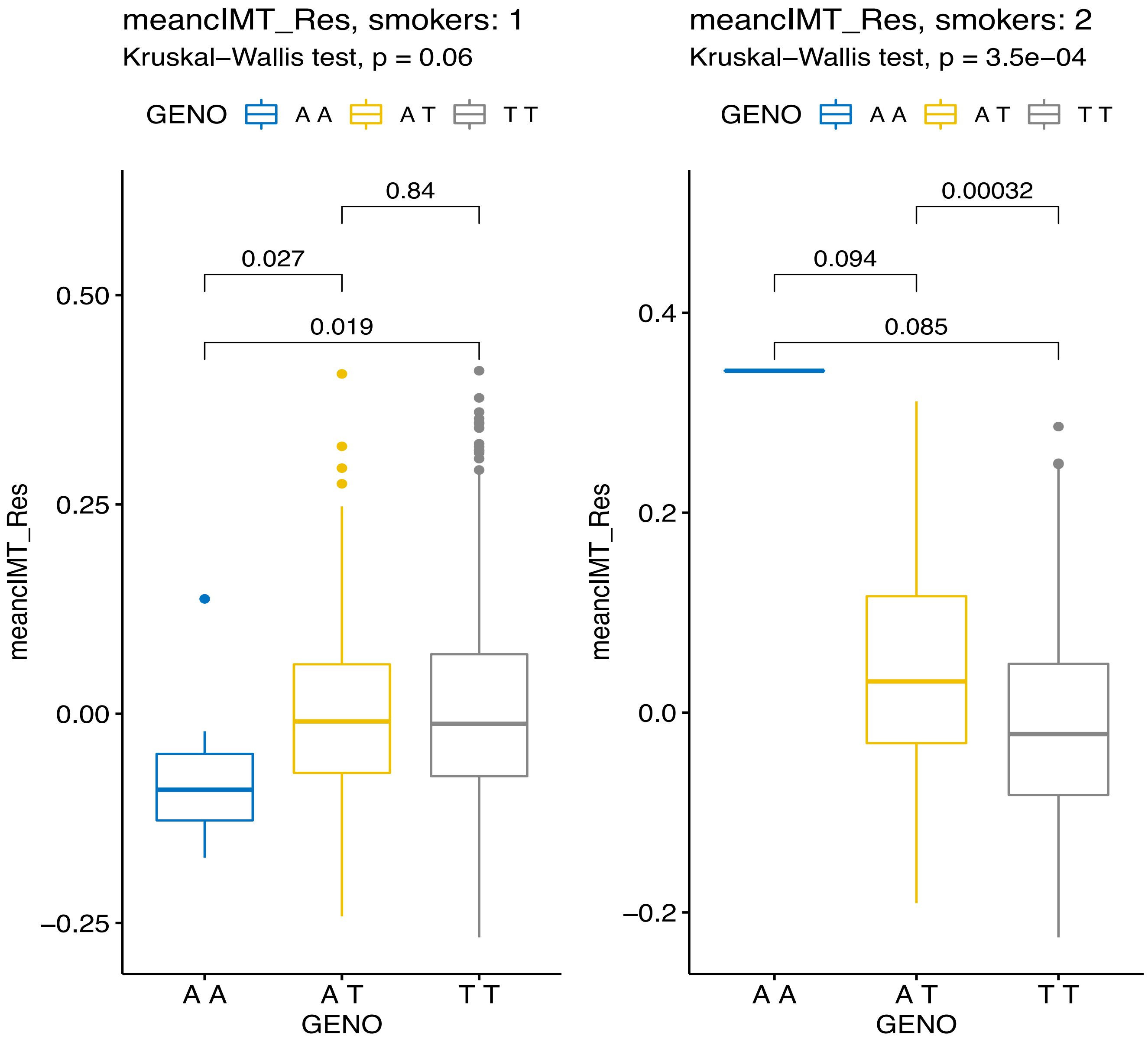

geno-rs76169119

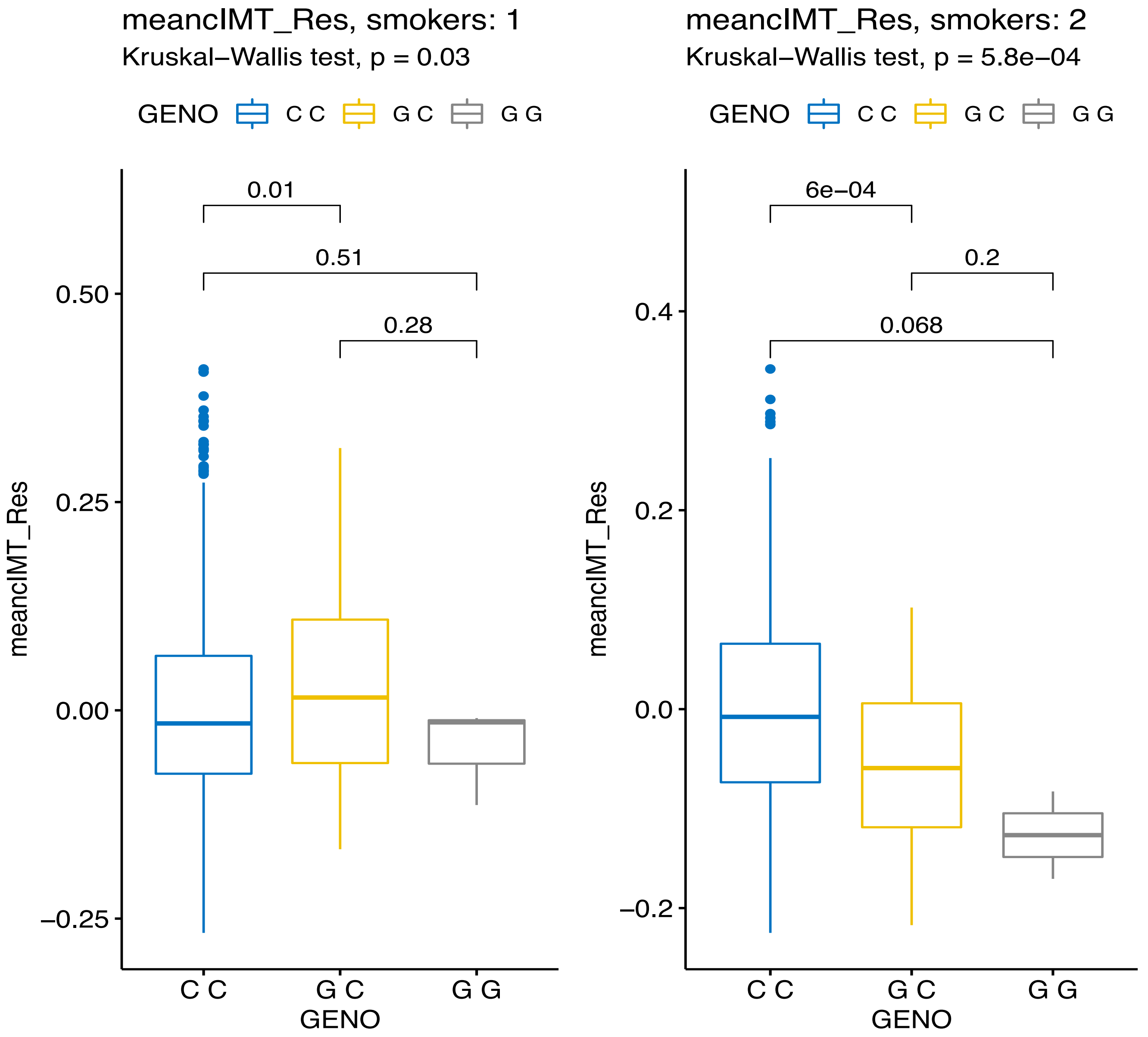

geno-rs77461169

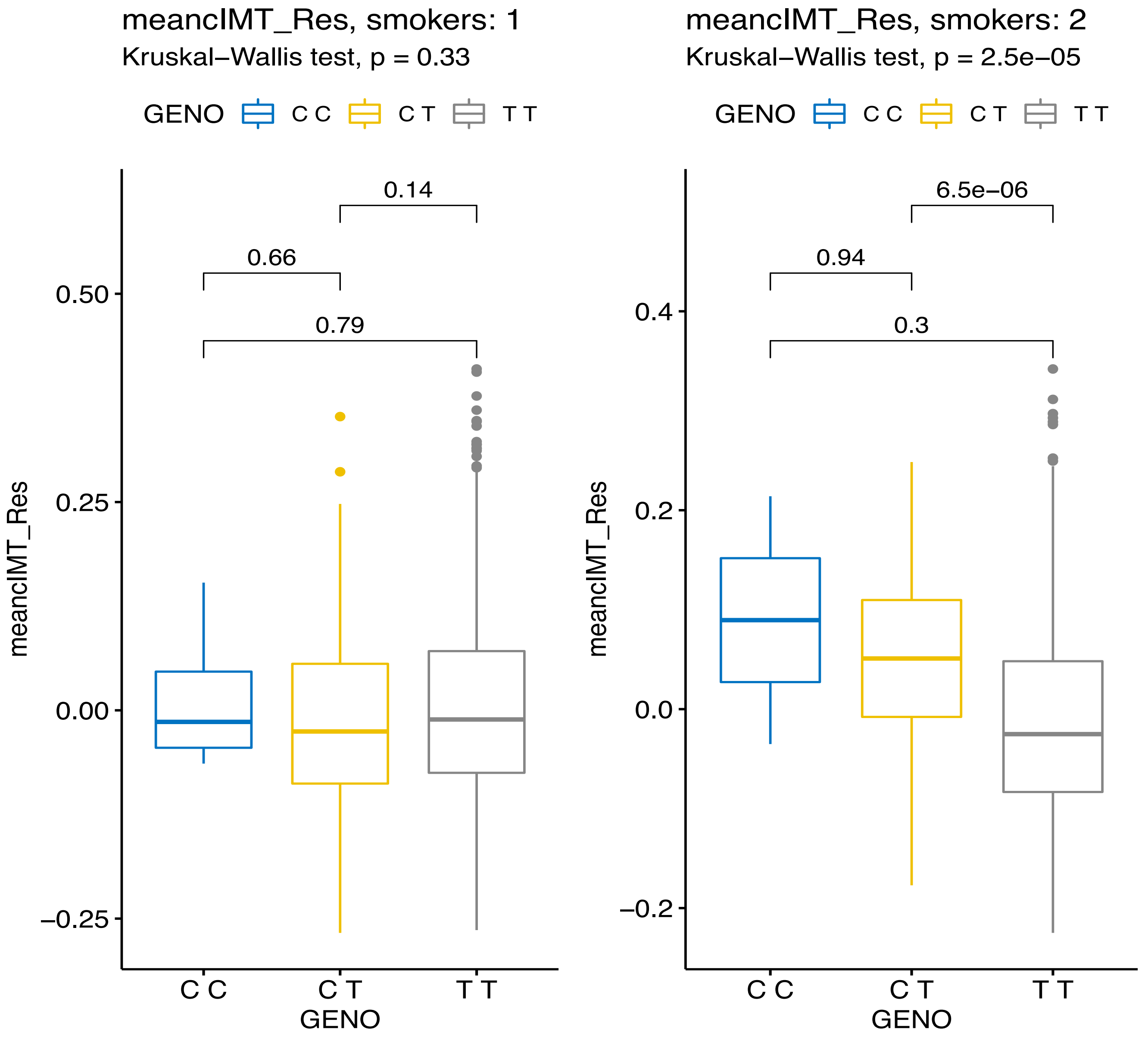

geno-rs77655815

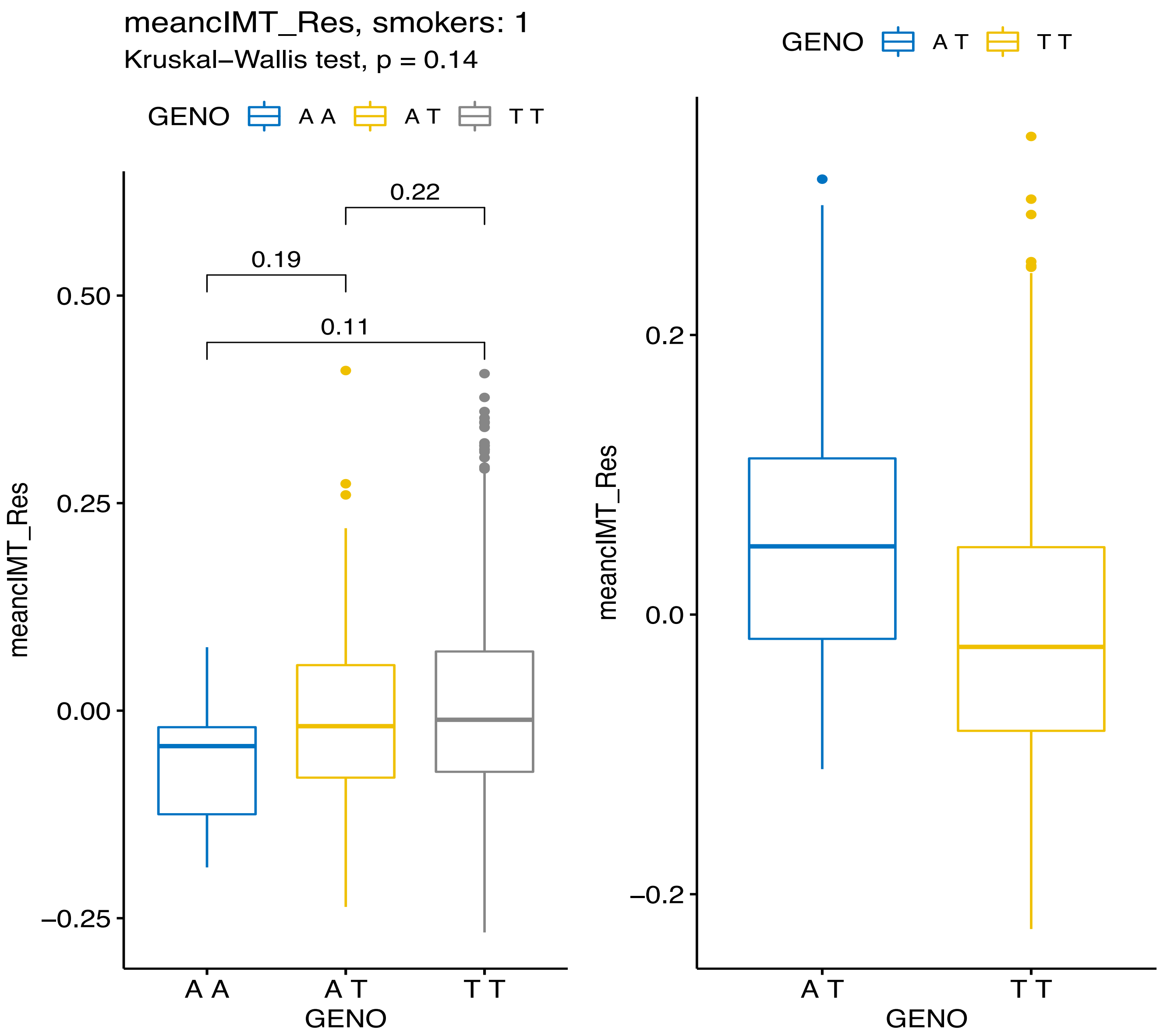

geno-rs112148169

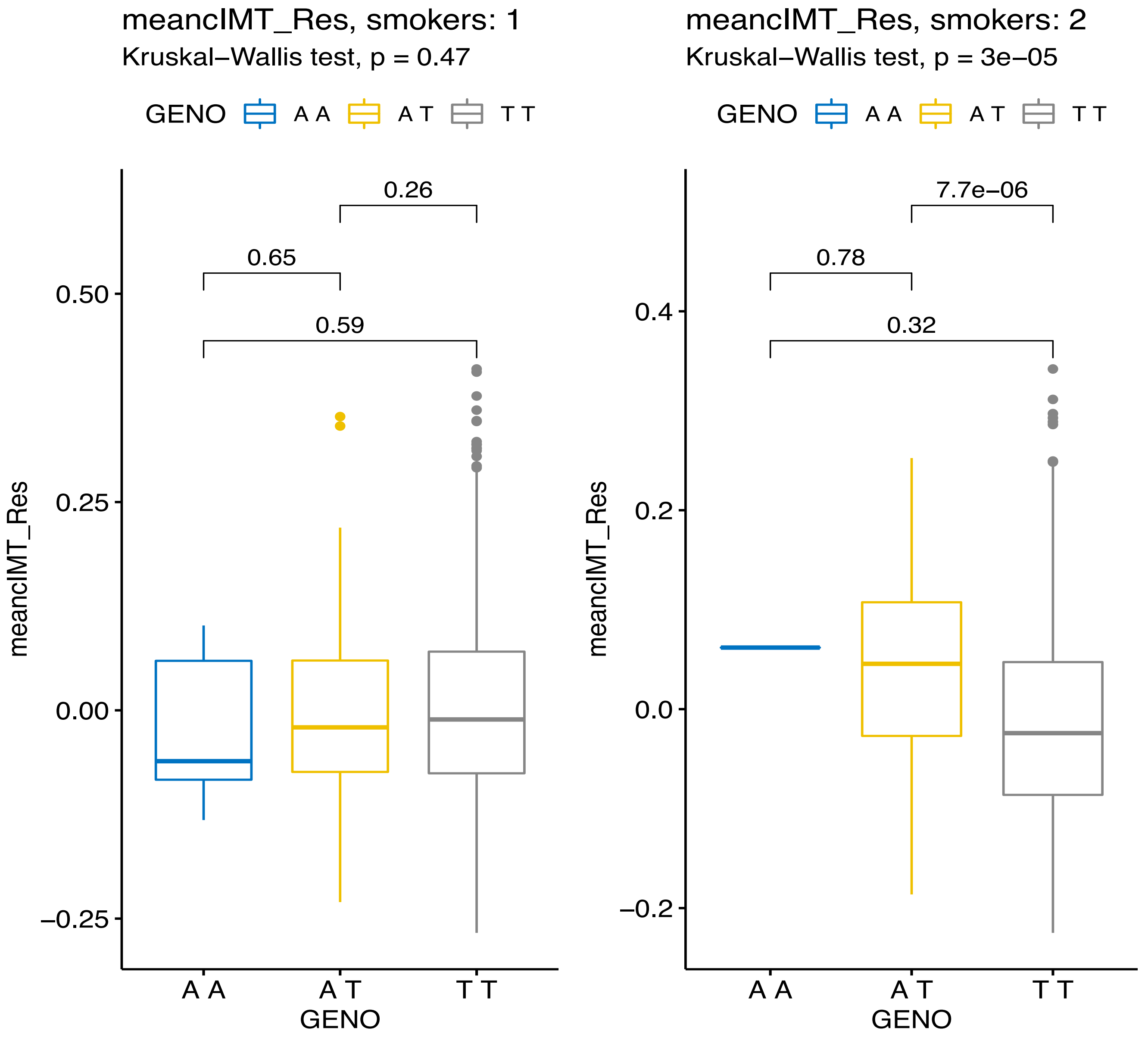

geno-rs551160836

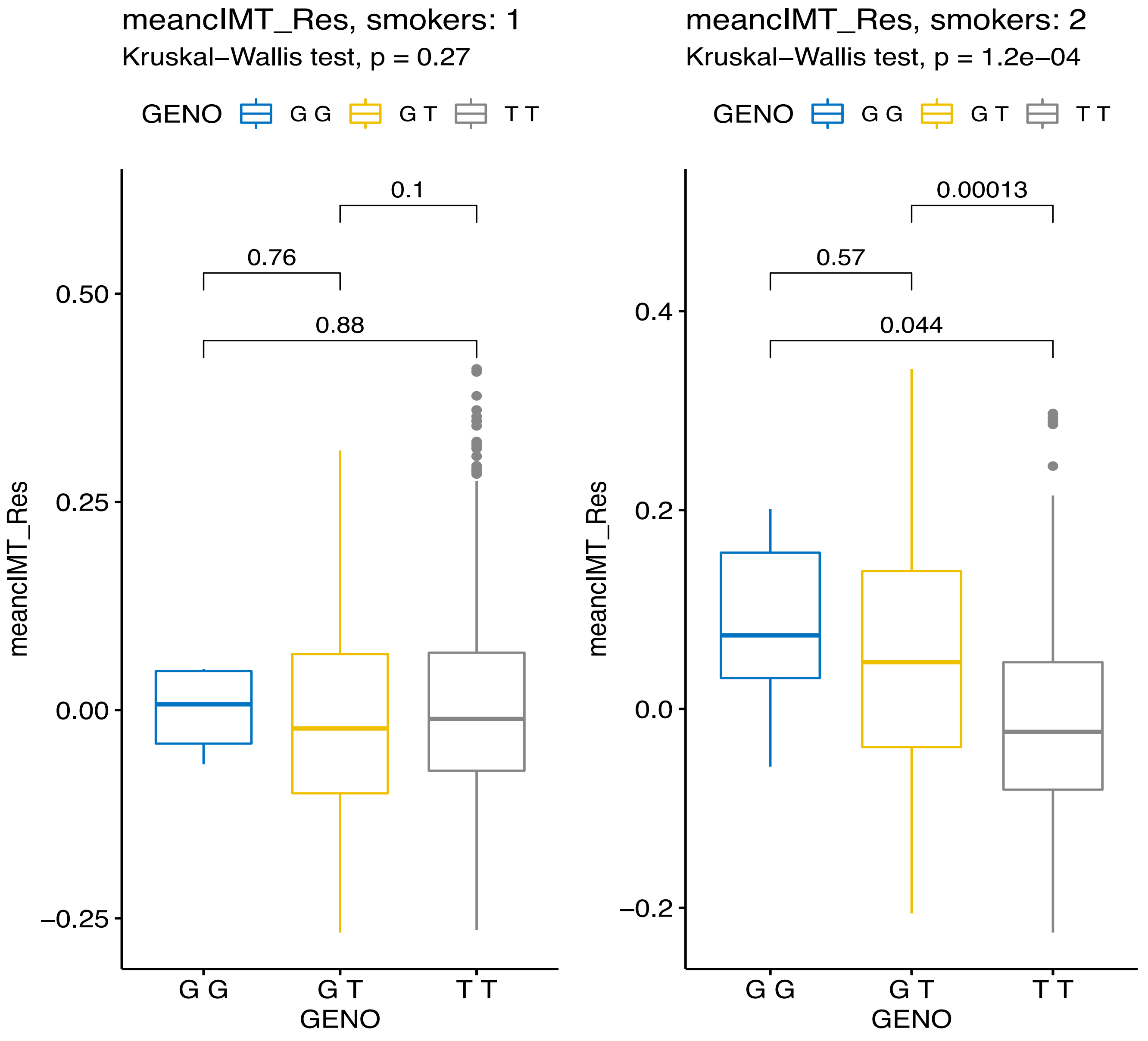

Supplement: Supplementary file 3 [file Image_2.pdf]
