# Supplementary material for: Novel and Known Gene-Smoking Interactions With cIMT Identified as Potential Drivers for Atherosclerosis Risk in West-African Populations of the AWI-Gen Study
Source: Front Genet. 2020 Feb 7;10:1354. doi: 10.3389/fgene.2019.01354 (PMC7025492; doi:10.3389/fgene.2019.01354)

Supplementary Figure 3 a: Circos plots showing genes on chromosomes that were linked to risk (P<1E-05) loci in the GWAS of Nanoro sample (blue regions) by eQTL mapping (green lines connecting an eQTL SNP to its associated gene) and/or chromatin interactions (orange lines connecting two interacting regions) and showed evidence of interaction across two independent genomic risk loci. Genes implicated by eQTLs are in green, by chromatin interactions are in orange, and by both eQTLs and chromatin interactions are in red. The outer layer shows a Manhattan plot containing the $-\log_{10}$-transformed two-tailed *P* value of each SNP from the GWAS meta-analysis (of linear and logistic regression statistics), with genome-wide significant SNPs colored according to LD patterns with the lead SNP. LD values has been generated from African samples from 1000 GP.

circos_chr1

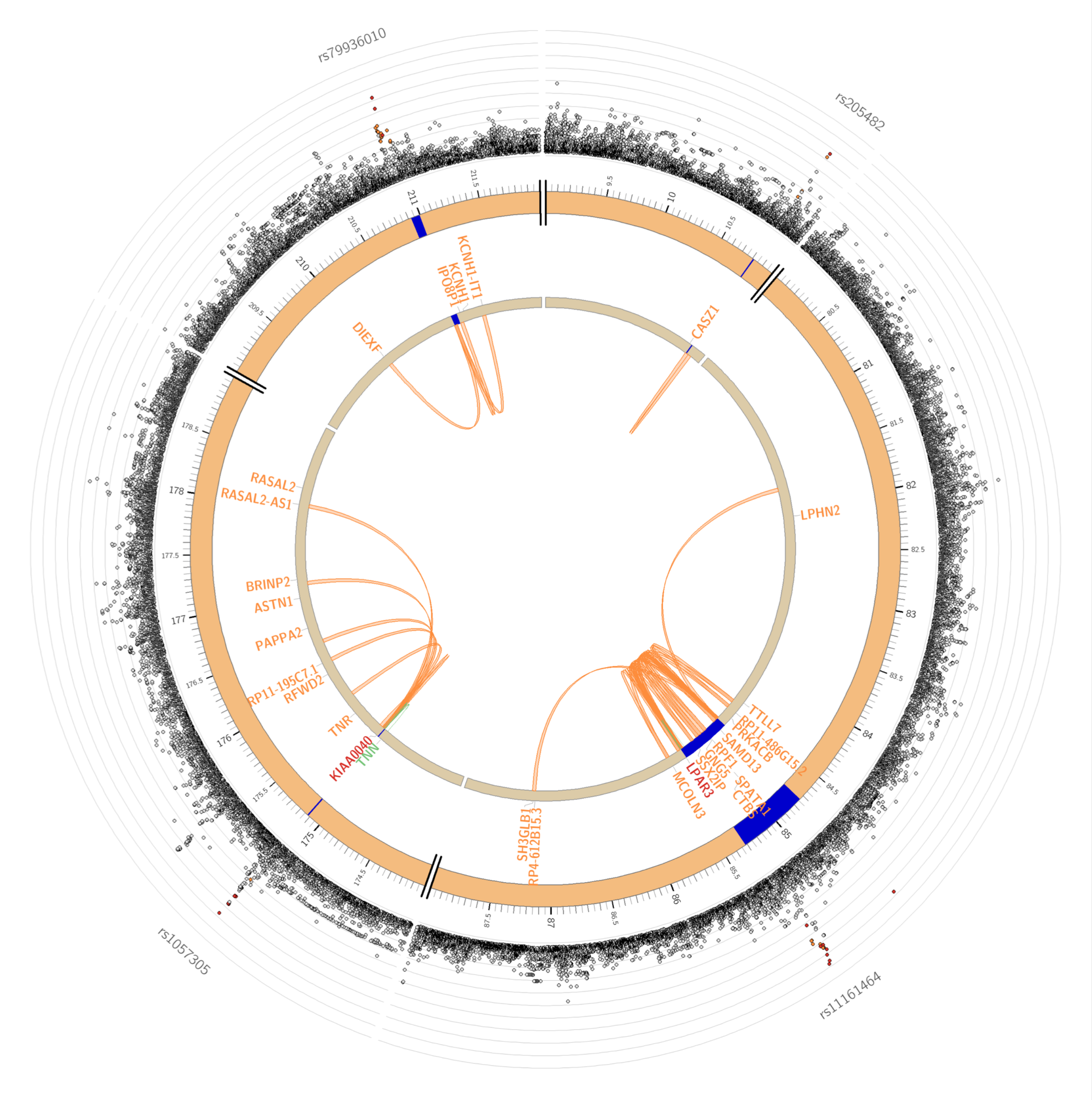

rs79936010
rs205482
rs1057305
rs11161464
KCNH1-IT1
KCNH1
IPO8P1
DIEXF
CASZ1
RASAL2
RASAL2-AS1
LPHN2
BRINP2
ASTN1
PAPPA2
RP11-195C7.1
RFWD2
TNR
KIAA0040
TNN
SH3GLB1
RP4-612B15.3
TTLL7
PRKACB
SAMD13
LPAR3
MCOLN3

circos_chr2

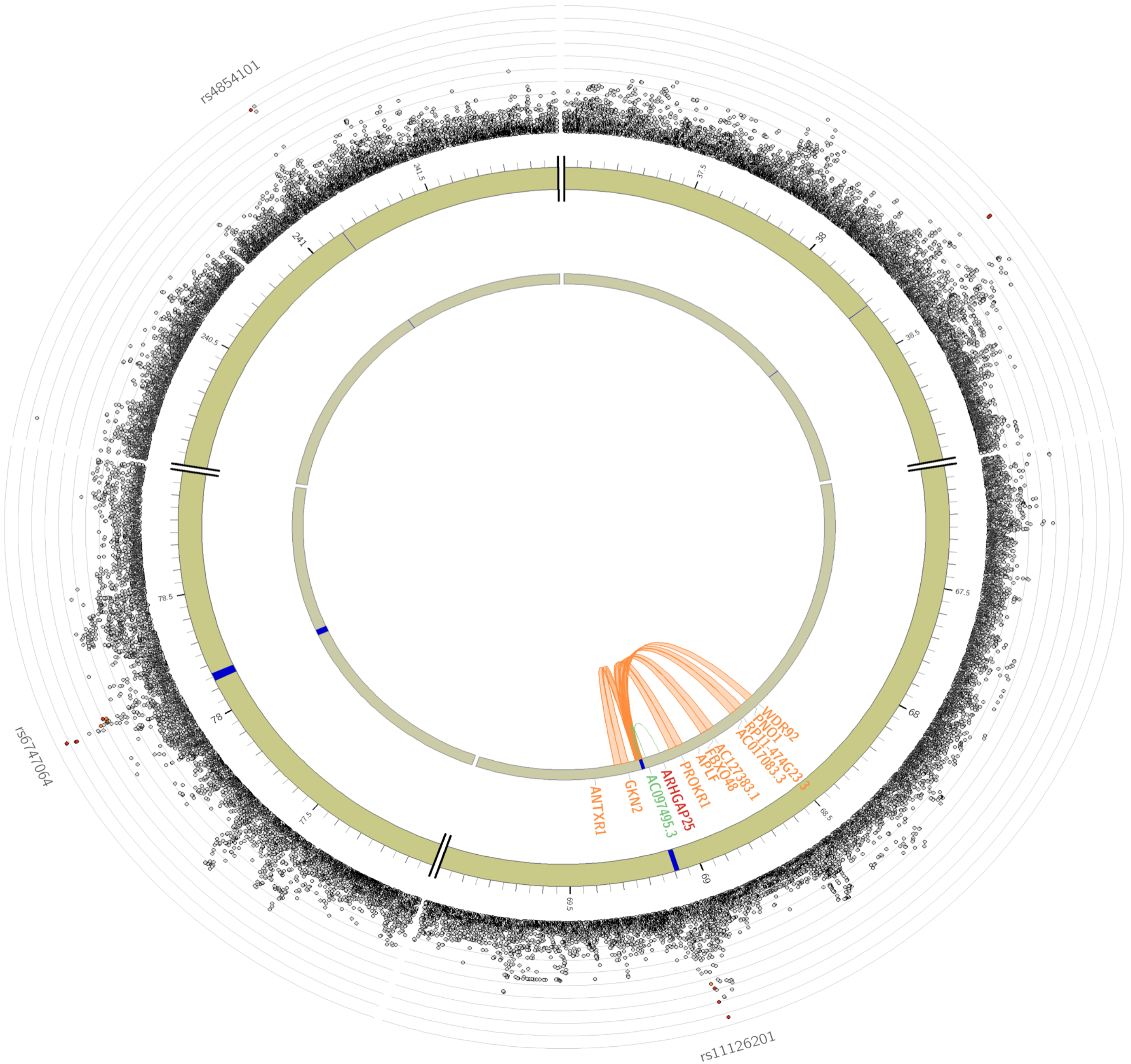
rs4854101
rs6747064
rs11126201
WDR92
PNO1
RP11-474G23
AC017083.3
AC127383.1
FBXO48
APLF
PROKR1
ARHGAP25
AC097495.3
GKN2
ANTXR1

circos_chr3

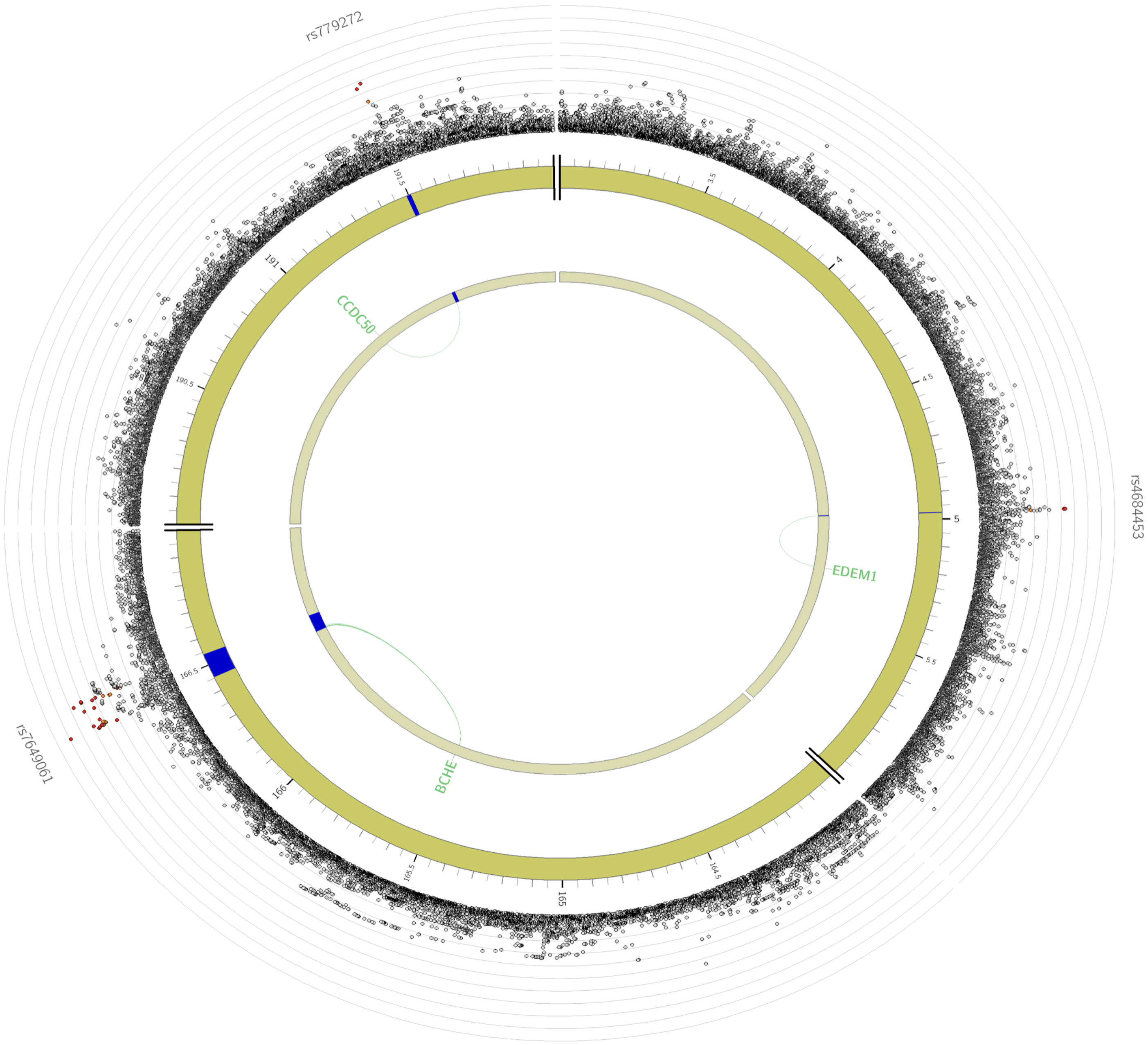

rs779272
rs4684453
rs7649061
CCDC50
EDEM1
BCHE
191.5
191
190.5
166.5
166
165.5
165
164.5
3.5
4
4.5
5
5.5

circos_chr4

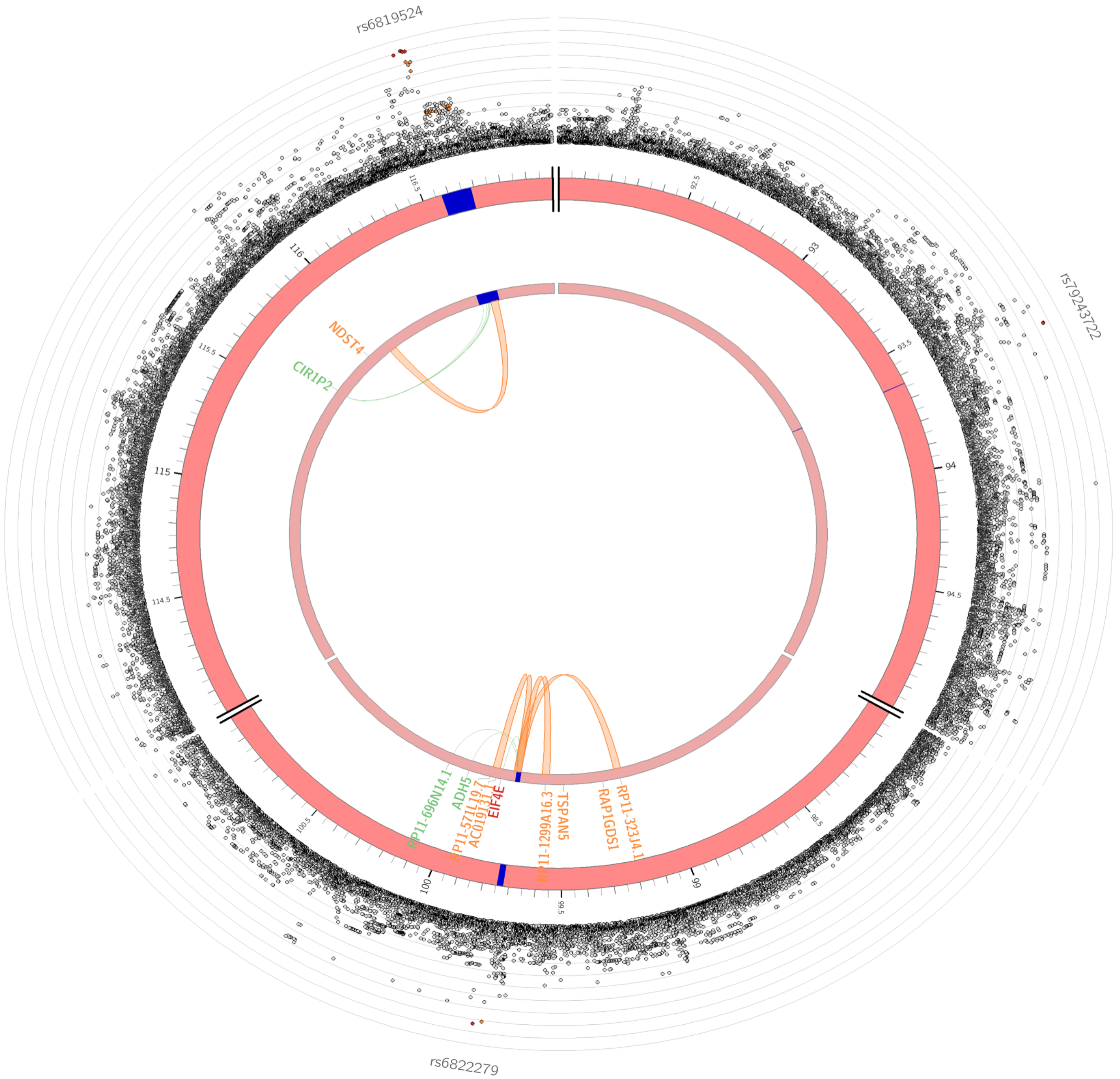

rs6819524
rs79243722
rs6822279
NDST4
CIR1P2
RP11-696N14.1
ADH5
RP11-571L19.7
AC019131.1
EIF4E
RP11-1299A16.3
TSPAN5
RAP1GDS1
RP11-323I4.1
116.5
116
115.5
115
114.5
92.5
93
93.5
94
94.5
100.5
100
99.5
99
98.5

circos_chr5

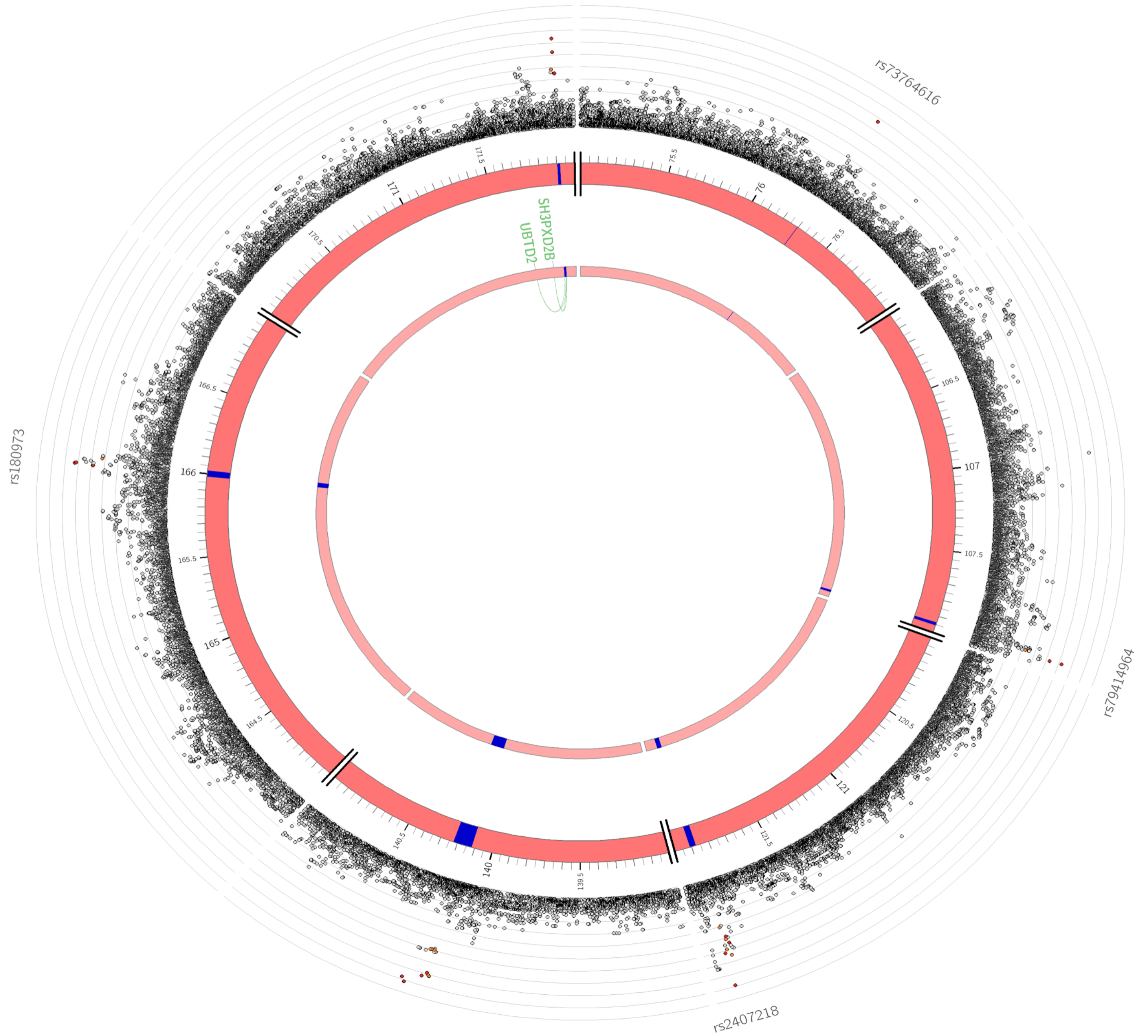
rs73764616
rs79414964
rs2407218
rs180973
SH3PXD2B
UBTD2
171.5
171
170.5
166.5
166
165.5
165
164.5
140.5
140
139.5
121.5
121
120.5
107.5
107
106.5
76.5
76
75.5

circos chr6

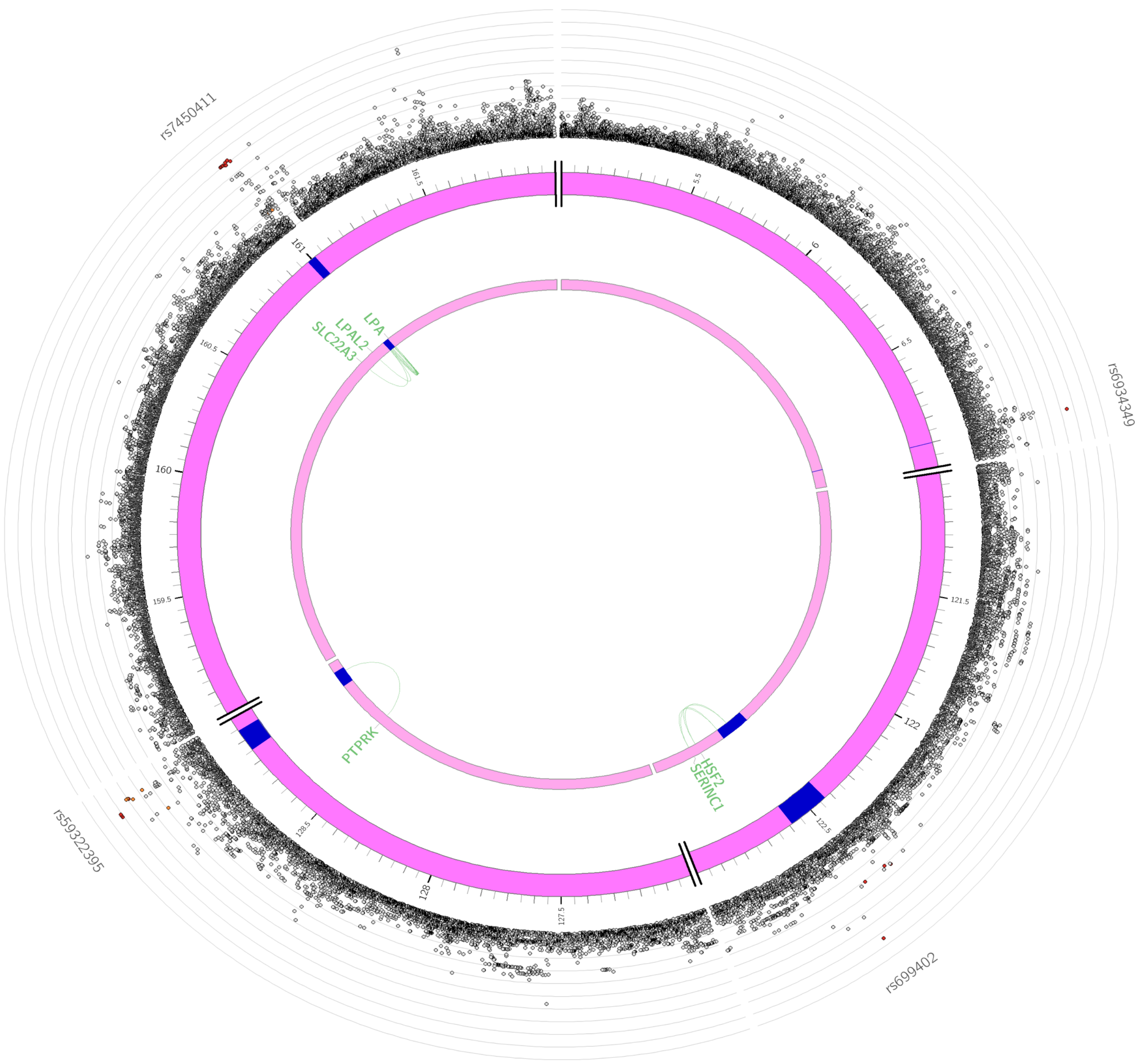
rs7450411
rs693349
rs59322395
rs699402
LPA
LPAL2
SLC22A3
PTPRK
HSF2
SERINC1
161.5
161
160.5
160
159.5
128.5
128
127.5
5.5
6
6.5
121.5
122
122.5

circos_chr7

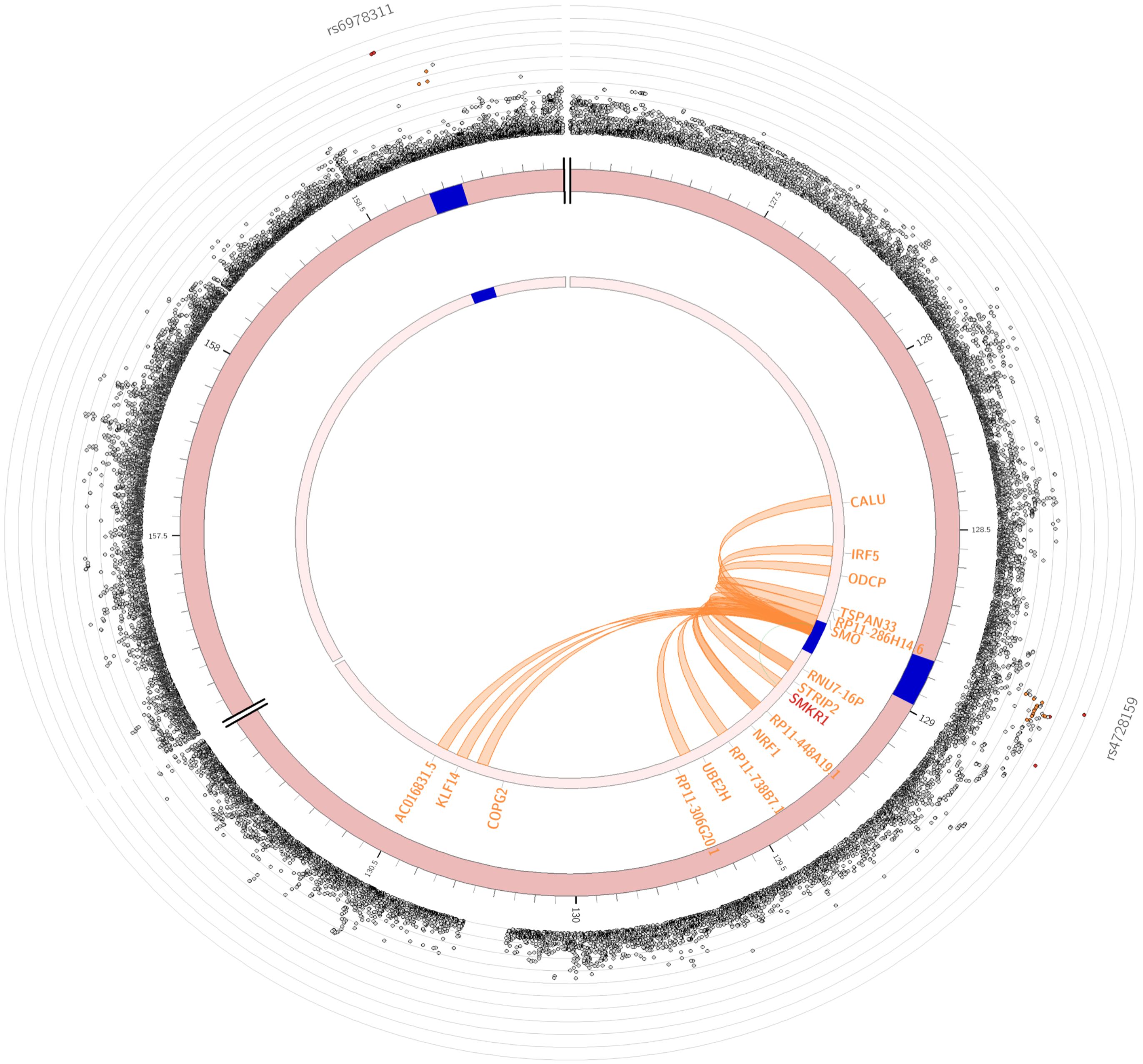

rs6978311
rs4728159
CALU
IRF5
ODCP
TSPAN33
RP11-286H14.6
SMO
RNU7-16P
STRIP2
SMKR1
RP11-448A19.1
NRF1
RP11-738B7.1
UBE2H
RP11-306G20.1
COPG2
KLF14
AC016831.5
127.5
128
128.5
129
129.5
130
130.5
157.5
158
158.5

circos_chr8

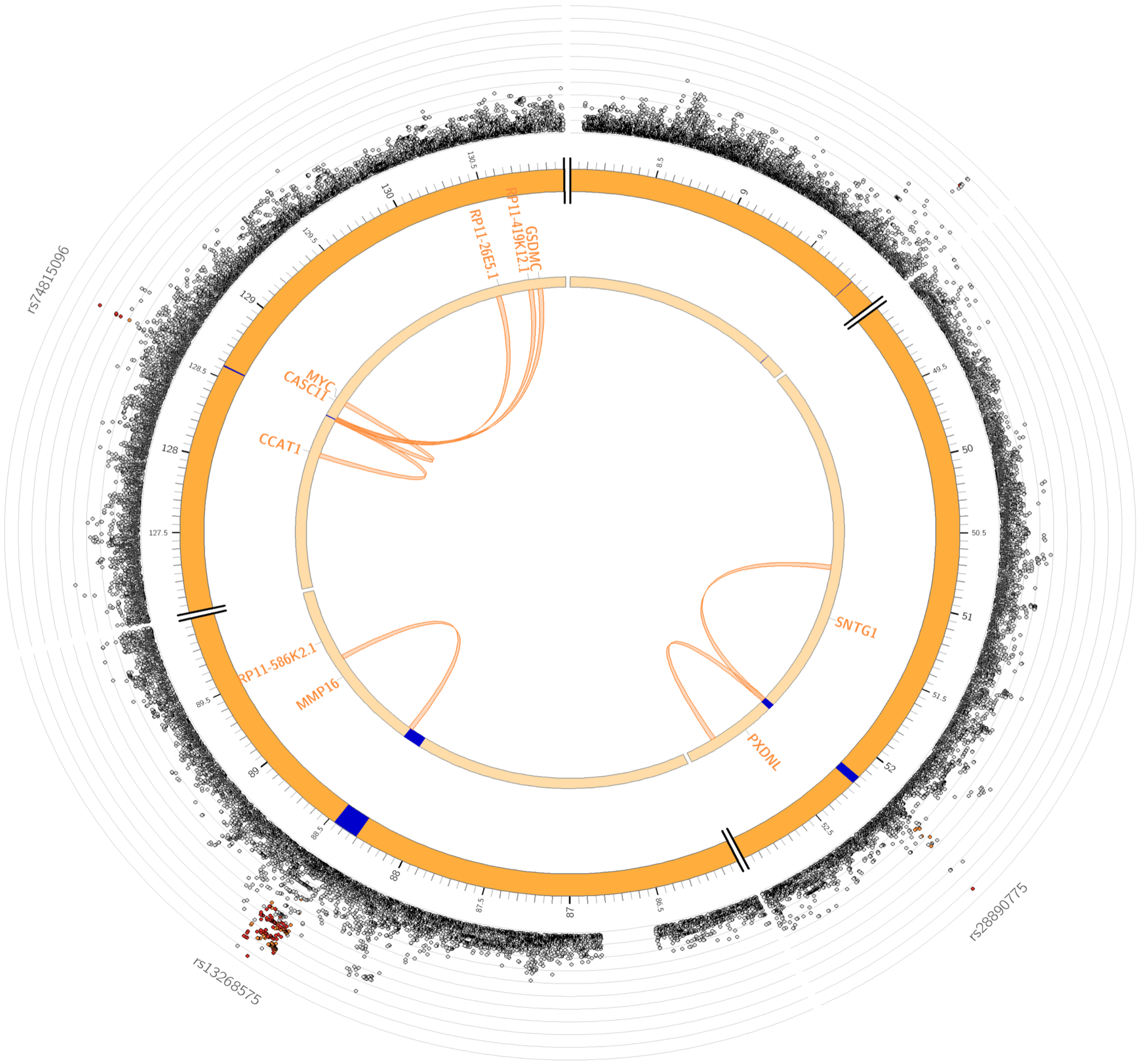

rs74815096
rs13268575
rs28890775
GSDMC
RP11-419K12.1
RP11-26E5.1
MYC
CASC11
CCAT1
RP11-586K2.1
MMP16
SNTG1
PXDNL

circos_chr9

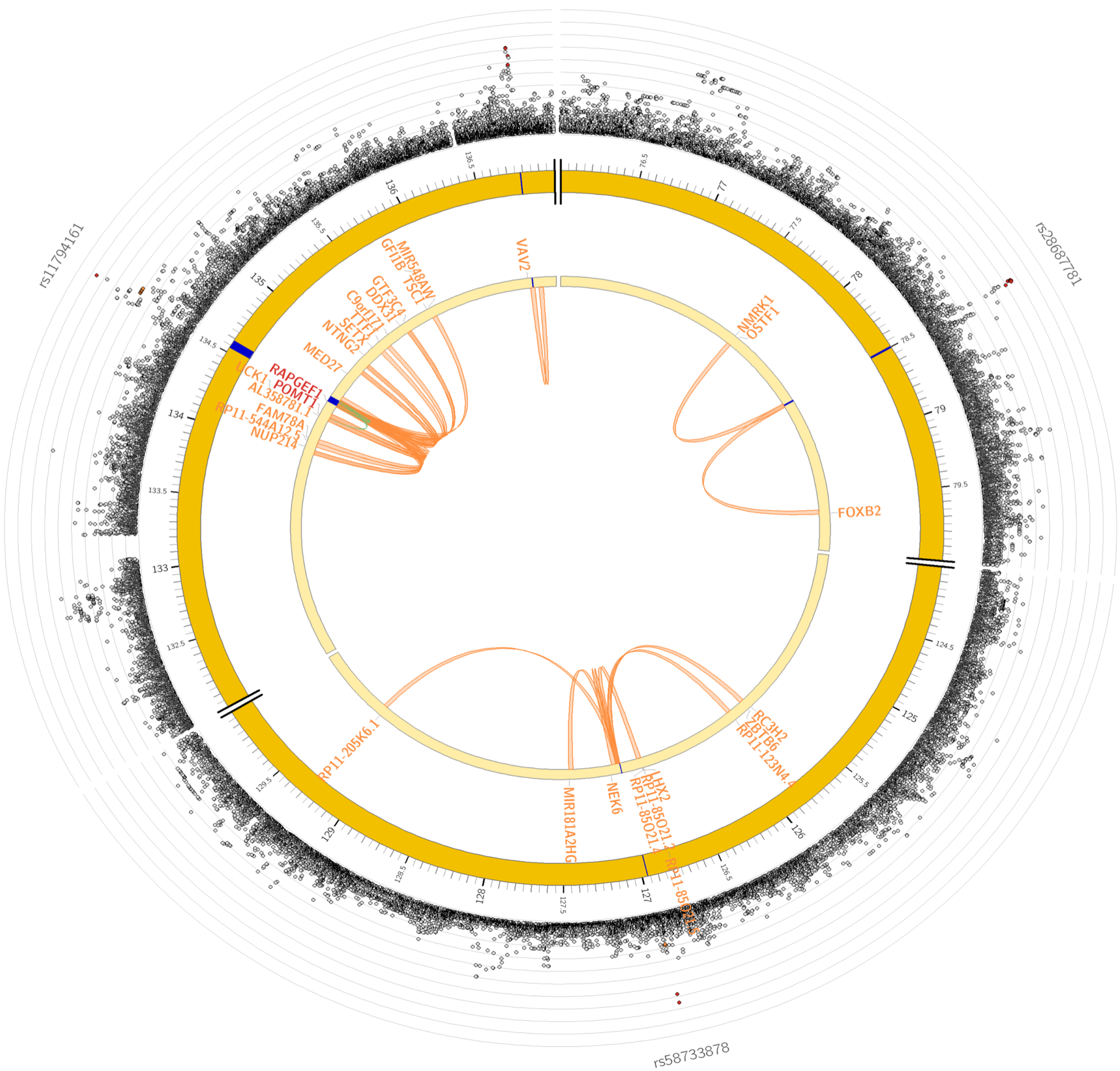
rs11794161
rs28687781
rs58733878
VAV2
NMRK1
OSTF1
FOXB2
MED27
NTNG2
SETX
GTF3C4
DDX31
TSC1
GFI1B
RAPGEF1
POMT1
AL358781.1
FAM78A
NUP214
RP11-205K6.1
MIR181A2HG
NEK6
LHX2
RC3H2
ZBTB6

circos_chr10

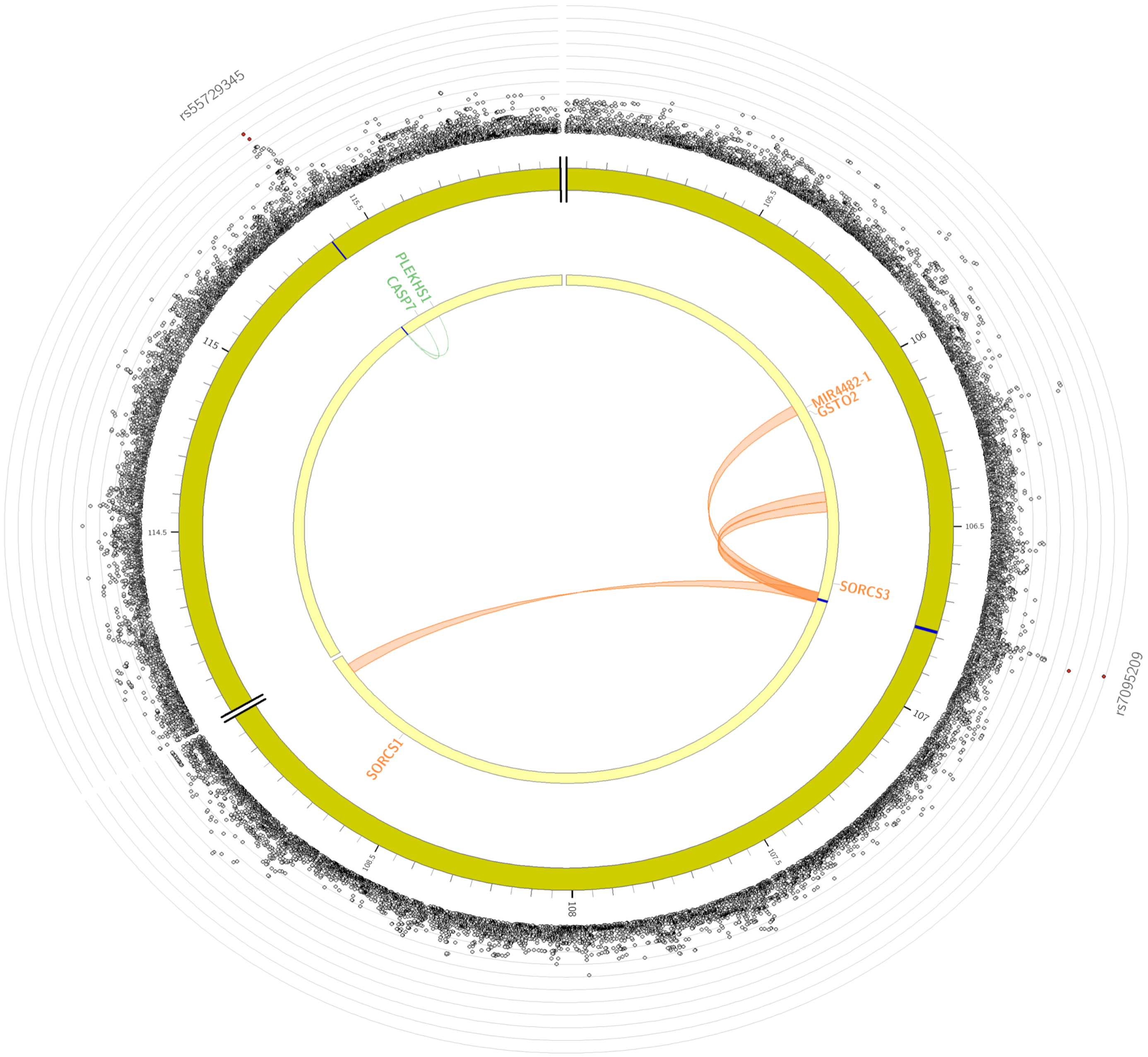

circos_chr11

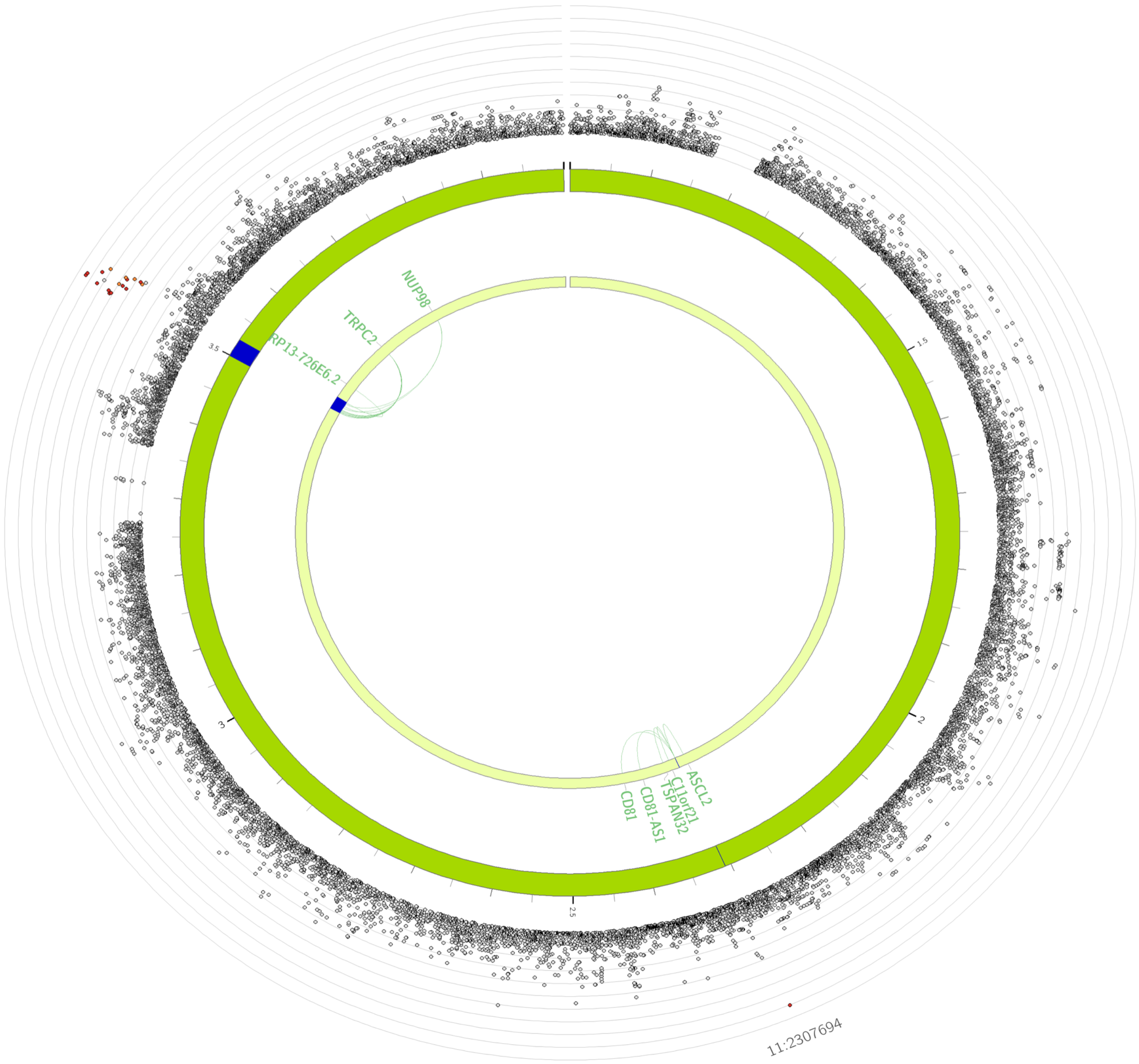

circos_chr12

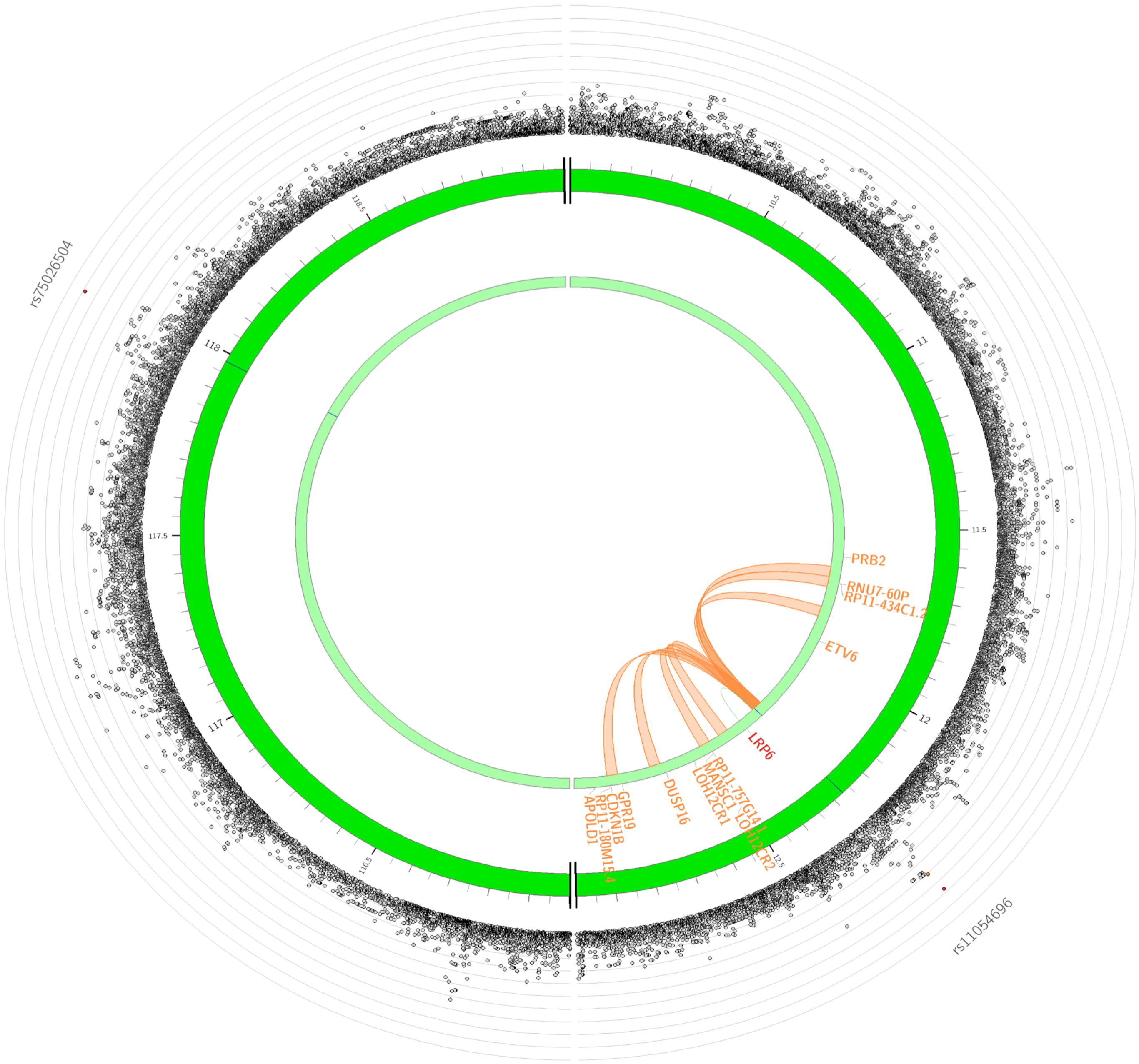

circos_chr13

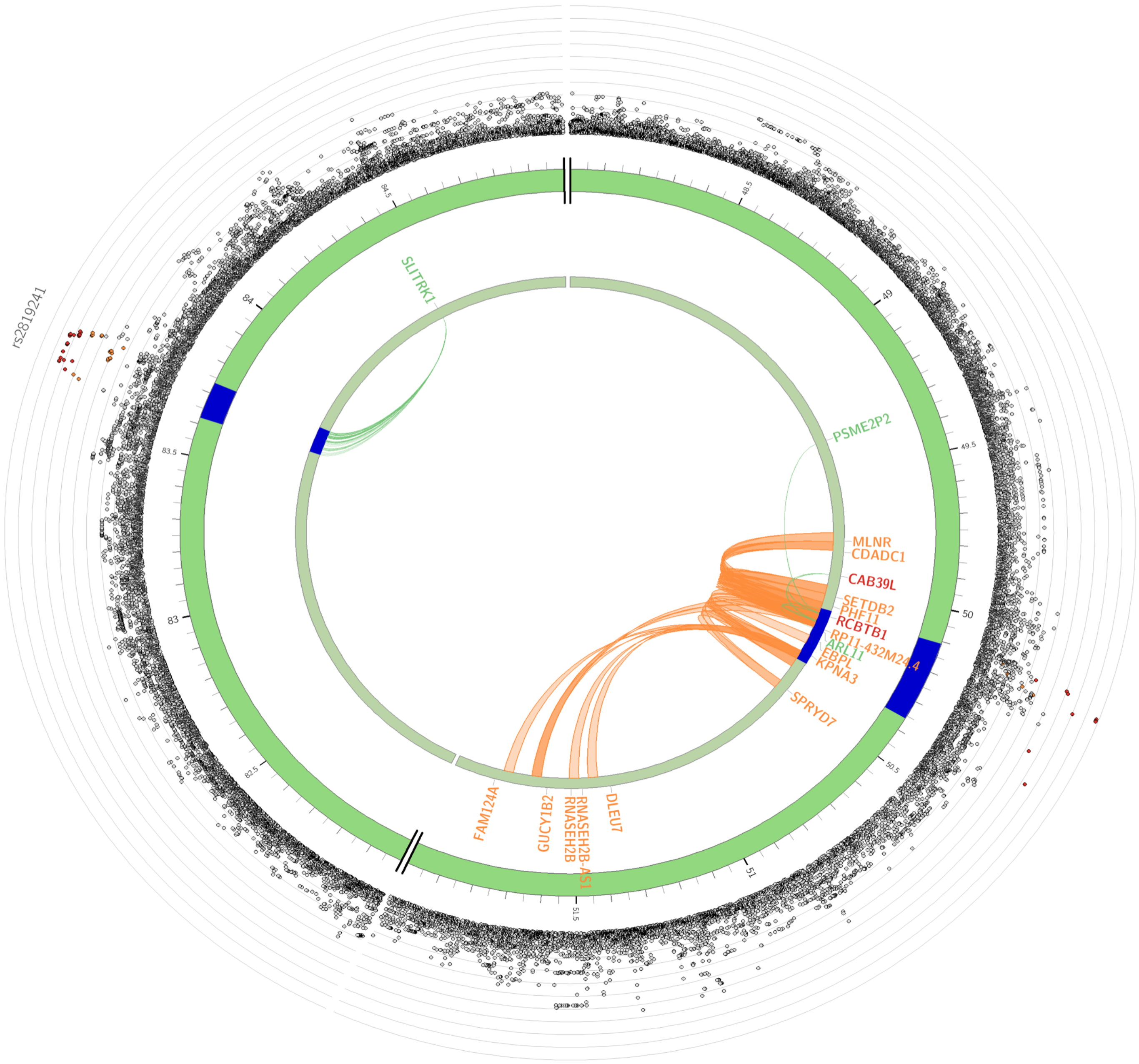

circos_chr14

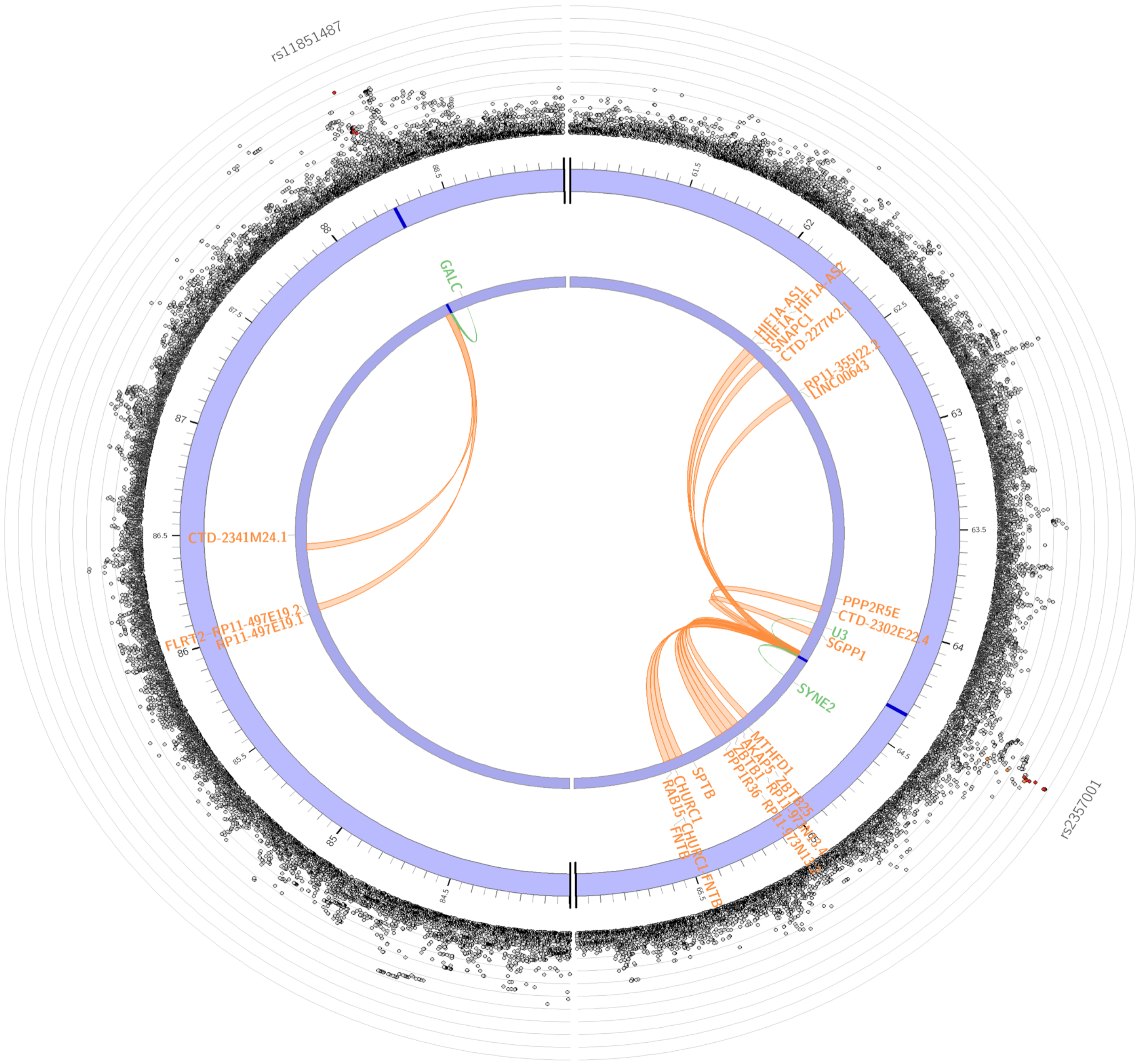

circos_chr16

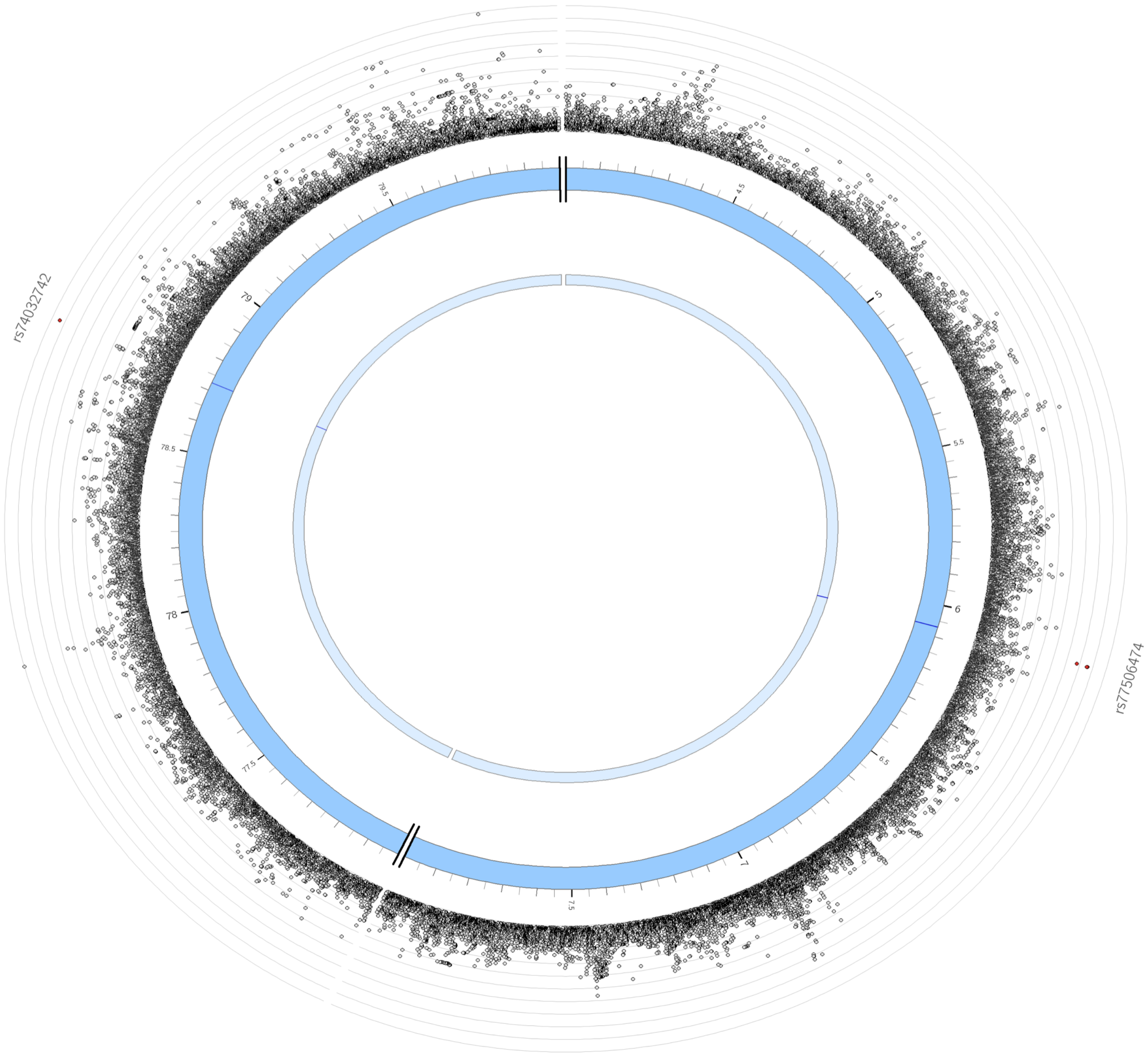

circos_chr18

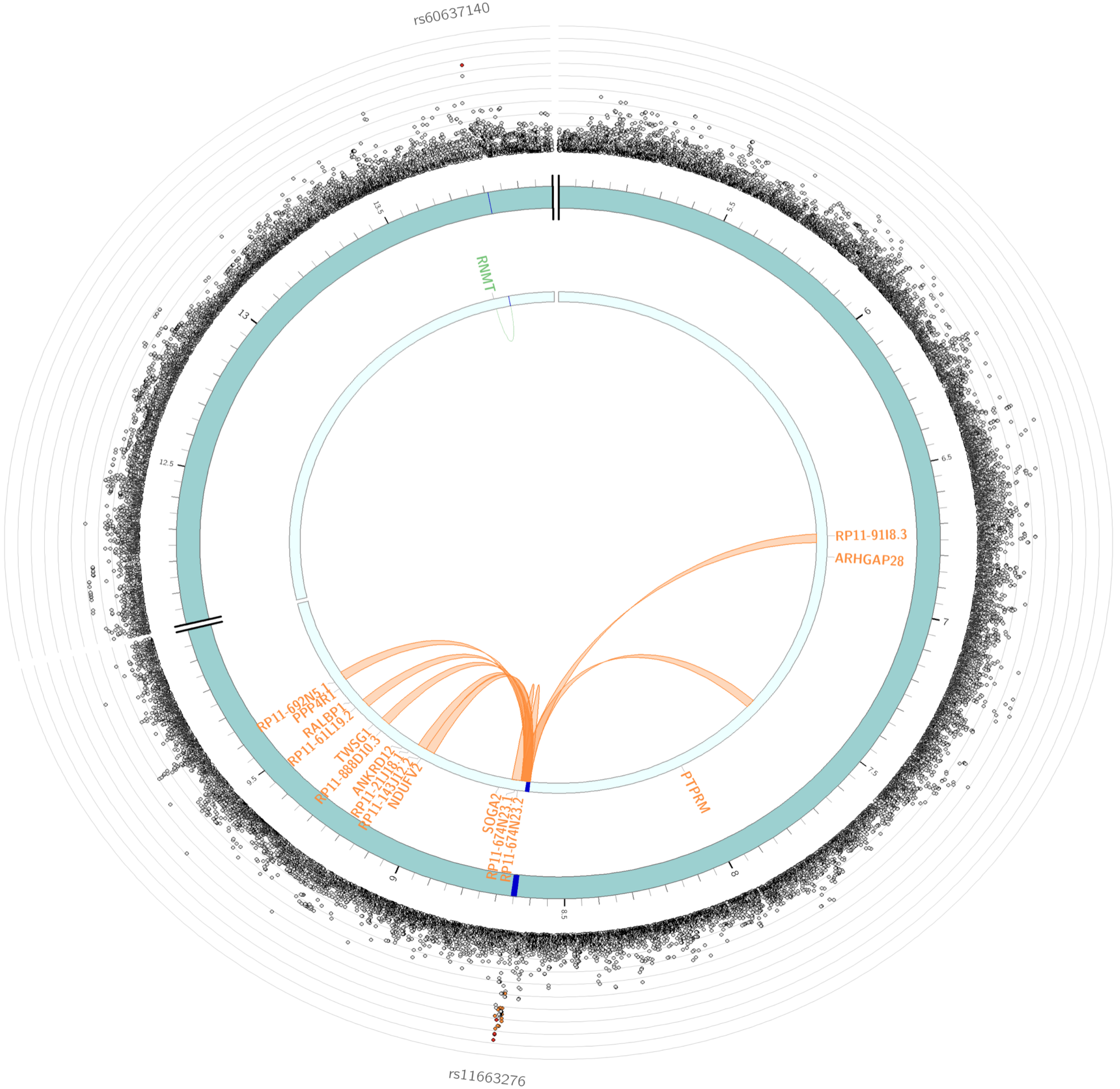

circos_chr19

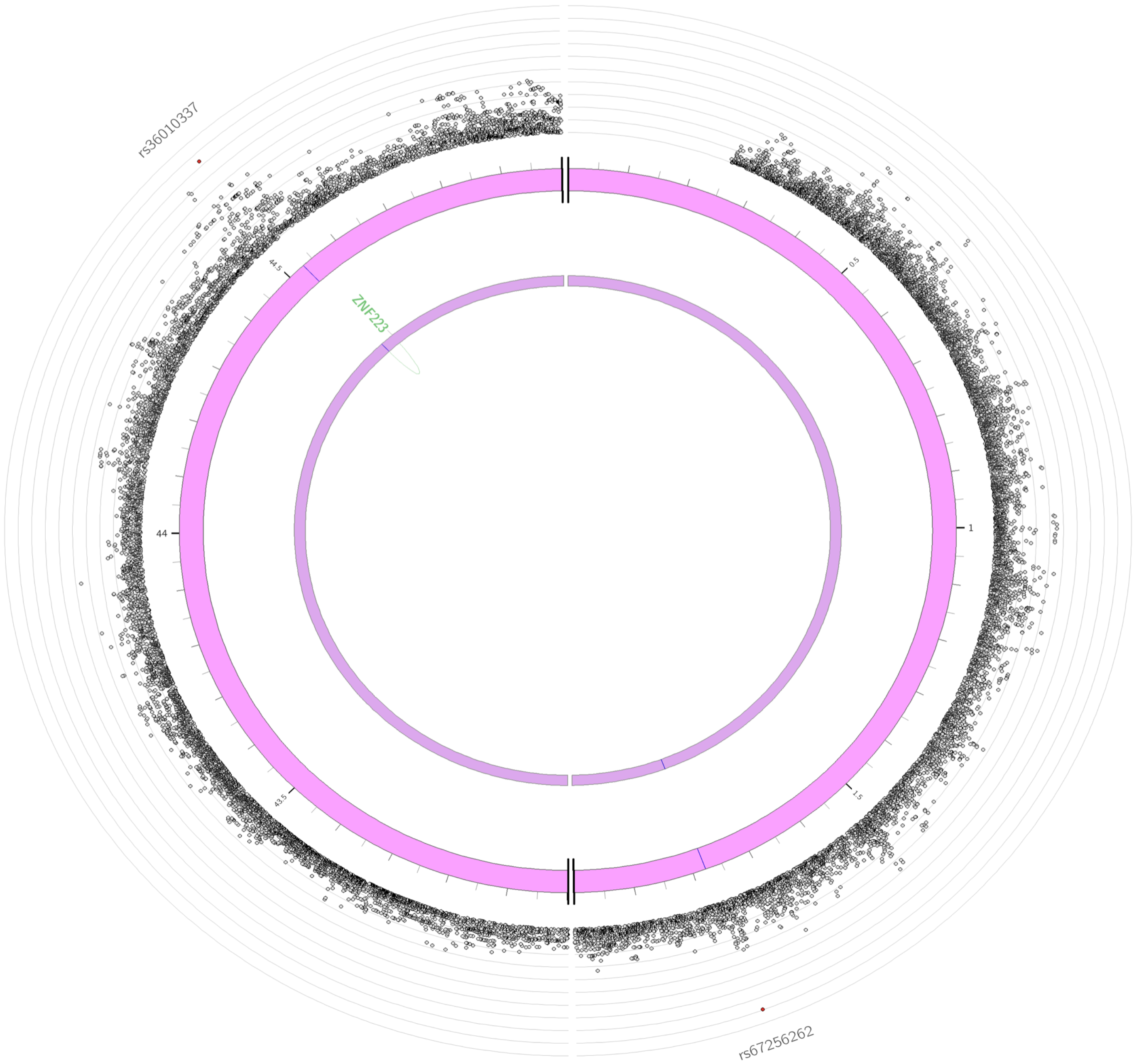

circos_chr20

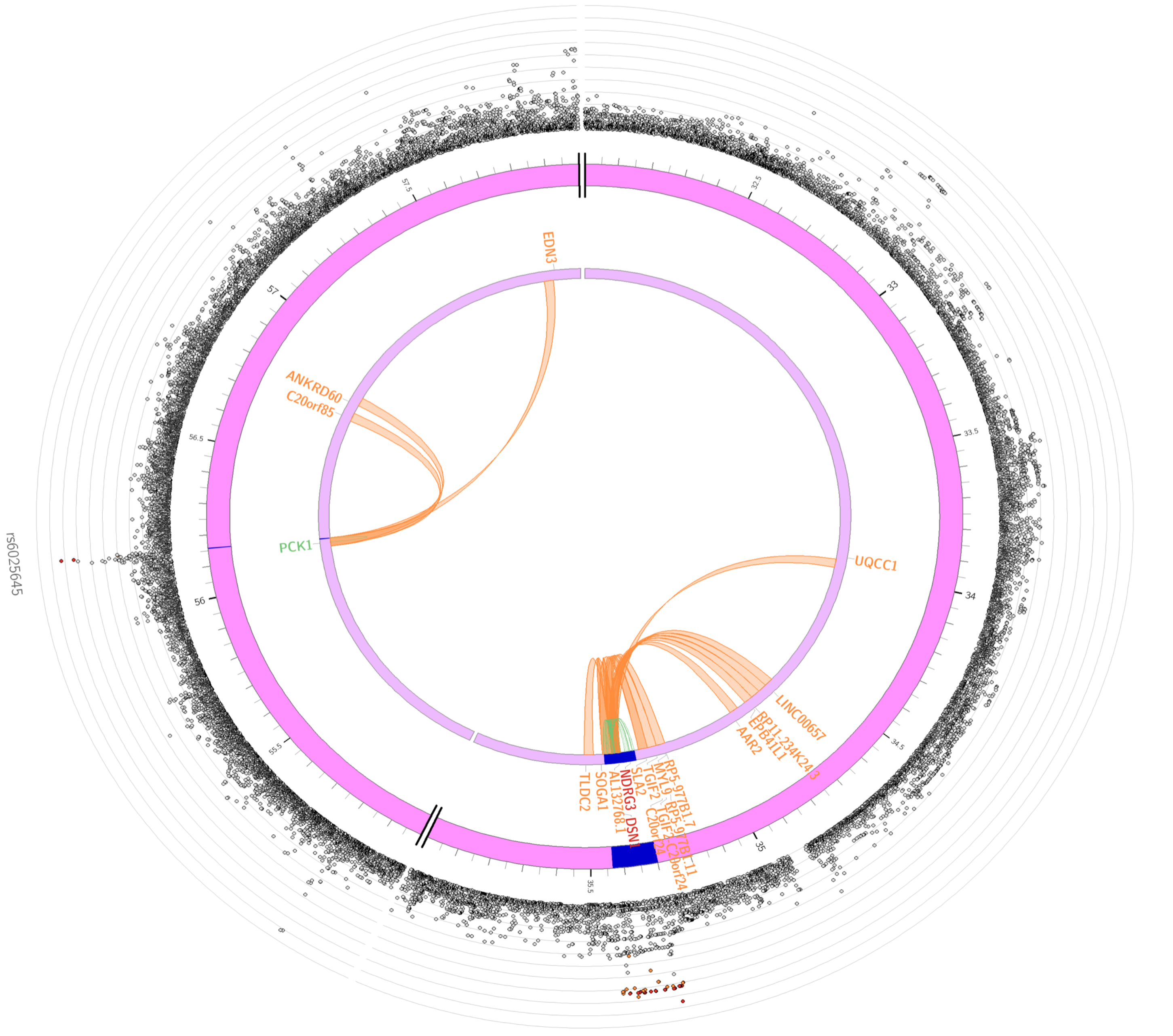

circos_chr21

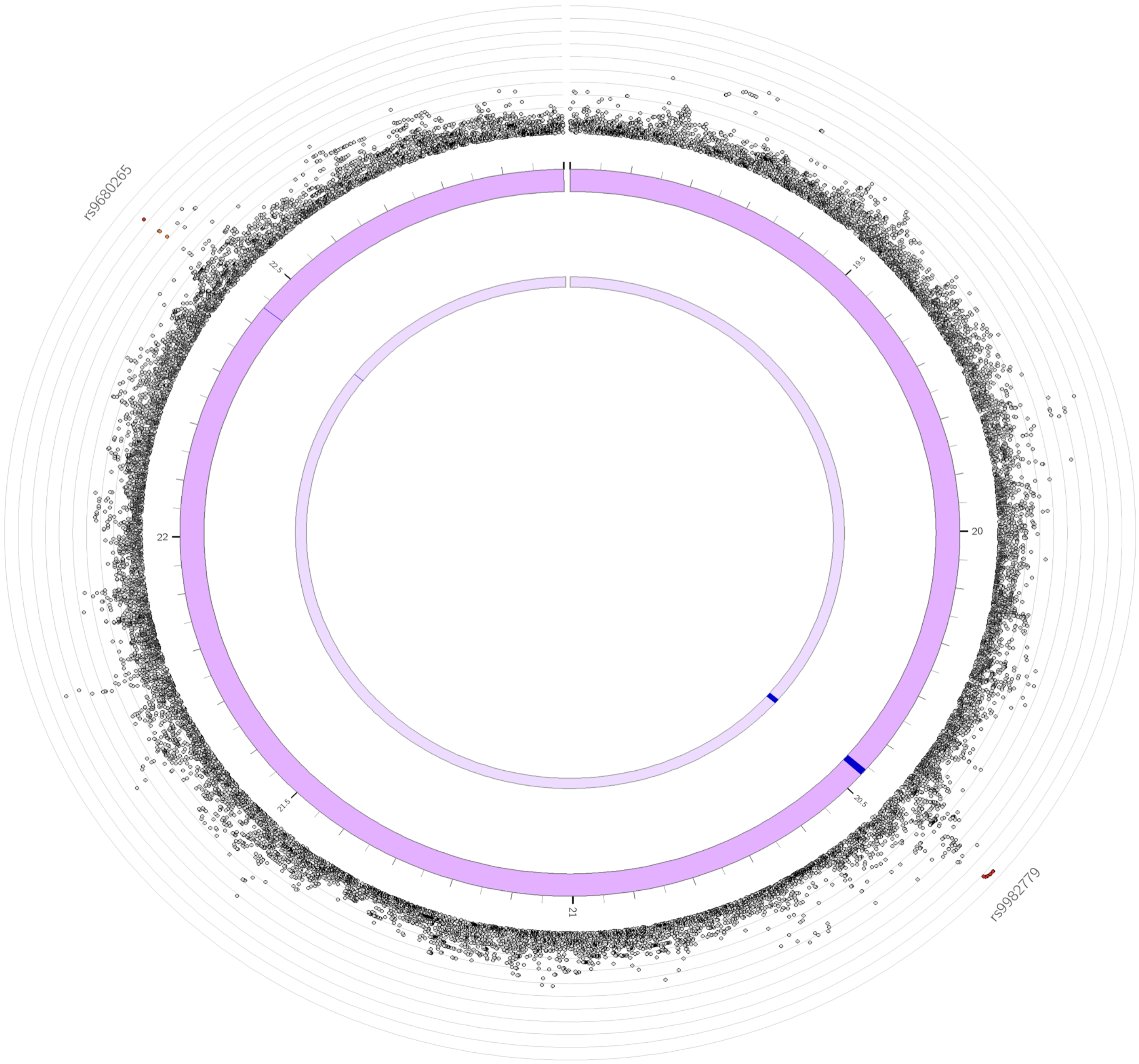

circos_chr22

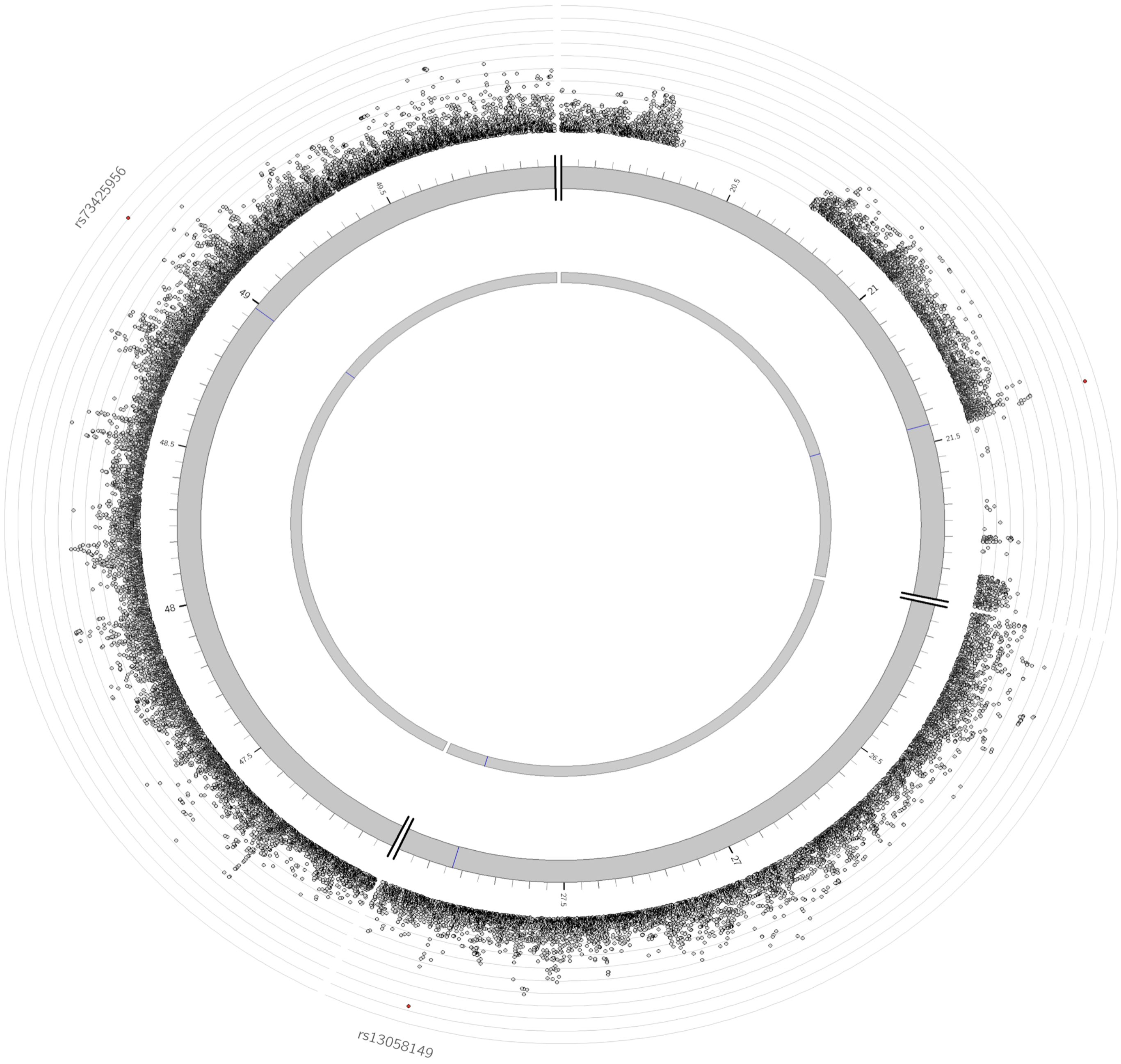

Supplementary Figure 3 b: Circos plots showing genes on chromosomes that were linked to risk (P<1E-05) loci in the GWAS of Navrongo sample (blue regions) by eQTL mapping (green lines connecting an eQTL SNP to its associated gene) and/or chromatin interactions (orange lines connecting two interacting regions) and showed evidence of interaction across two independent genomic risk loci. Genes implicated by eQTLs are in green, by chromatin interactions are in orange, and by both eQTLs and chromatin interactions are in red. The outer layer shows a Manhattan plot containing the $-\log_{10}$-transformed two-tailed *P* value of each SNP from the GWAS meta-analysis (of linear and logistic regression statistics), with genome-wide significant SNPs colored according to LD patterns with the lead SNP. LD values has been generated from African samples from 1000 GP.

circos_chr1

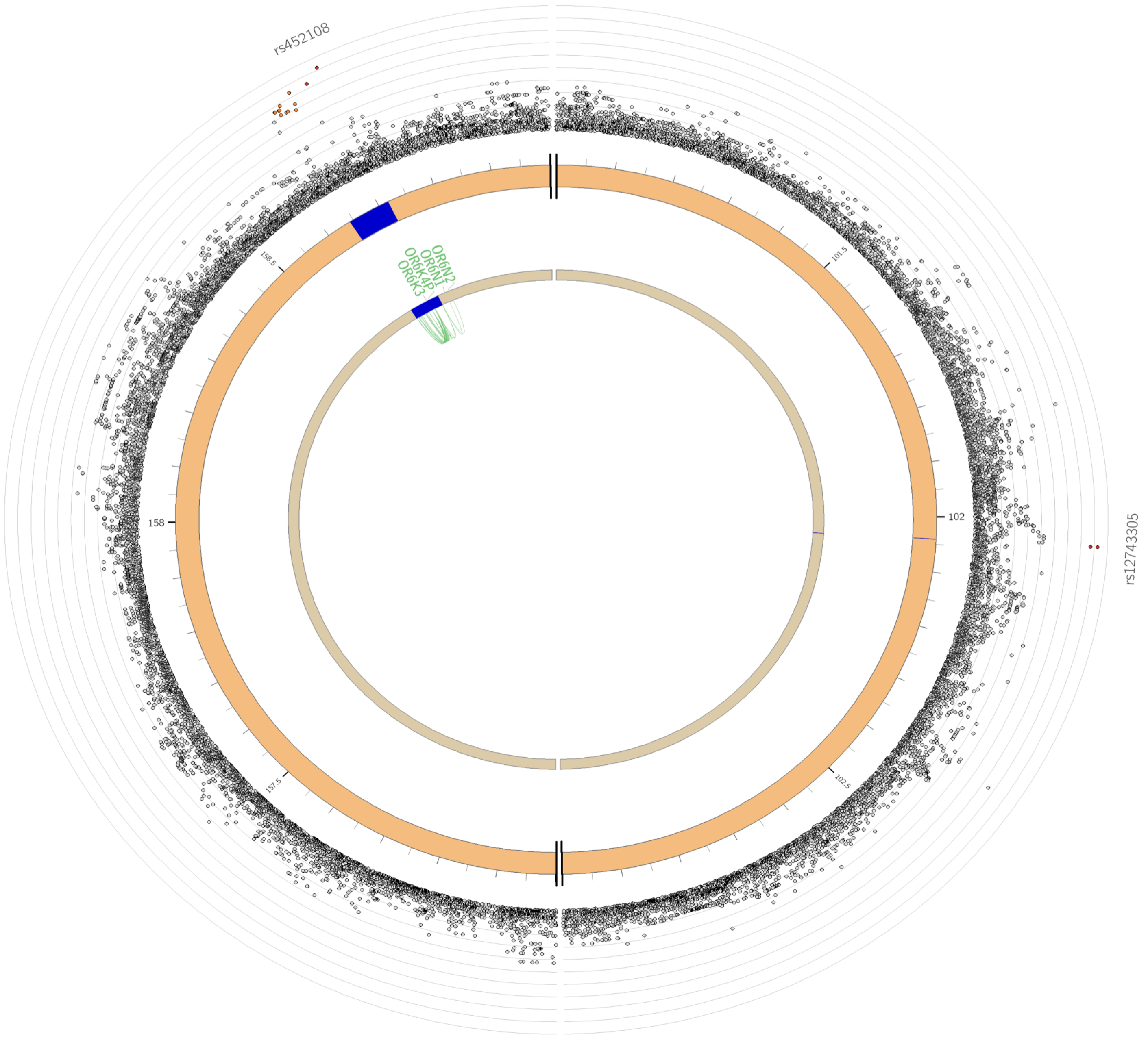

rs452108
rs12743305
158.5
158
157.5
101.5
102
102.5
OR6N2
OR6N1
OR6K4P
OR6K3

circos_chr2

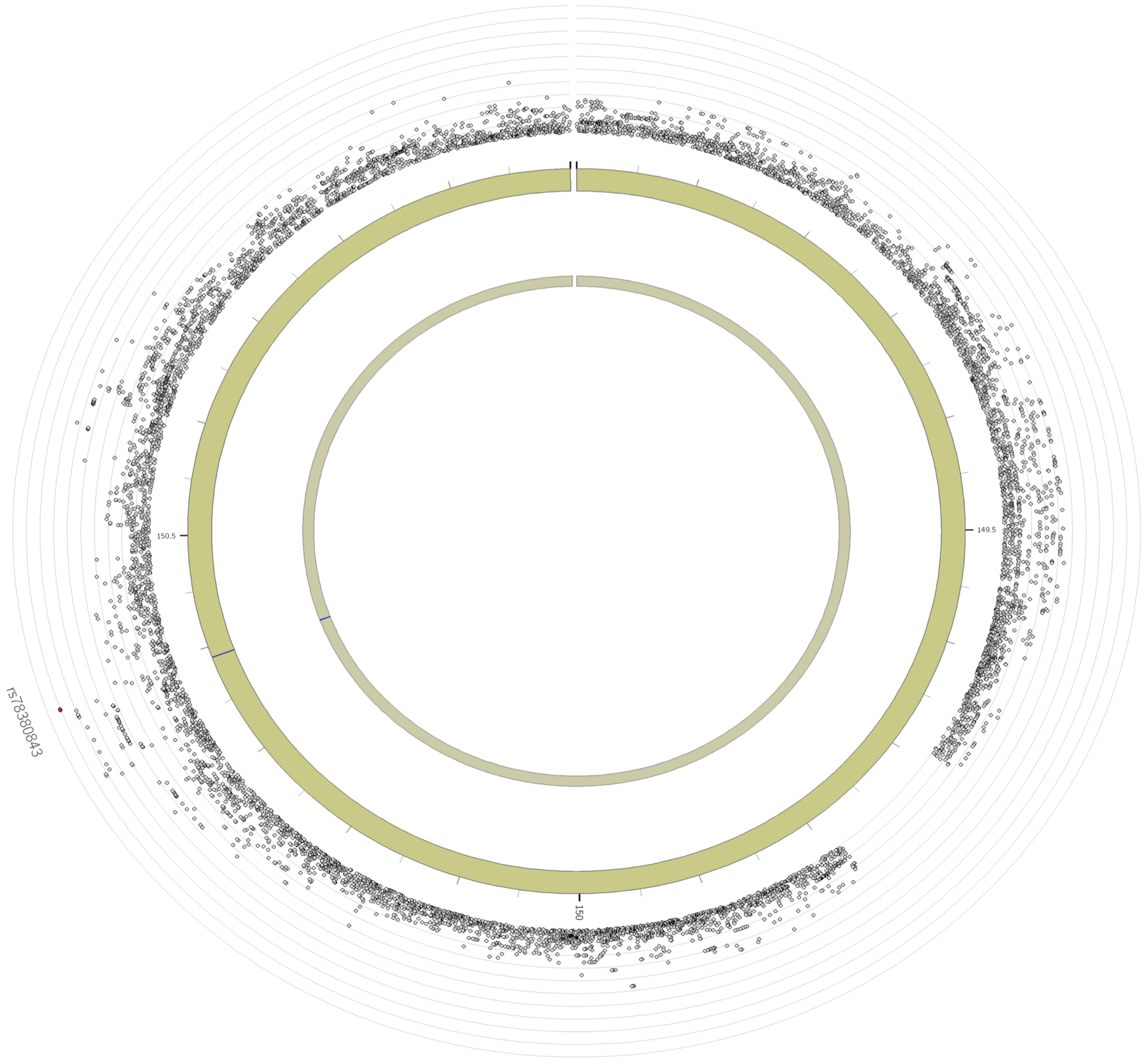

circos_chr4

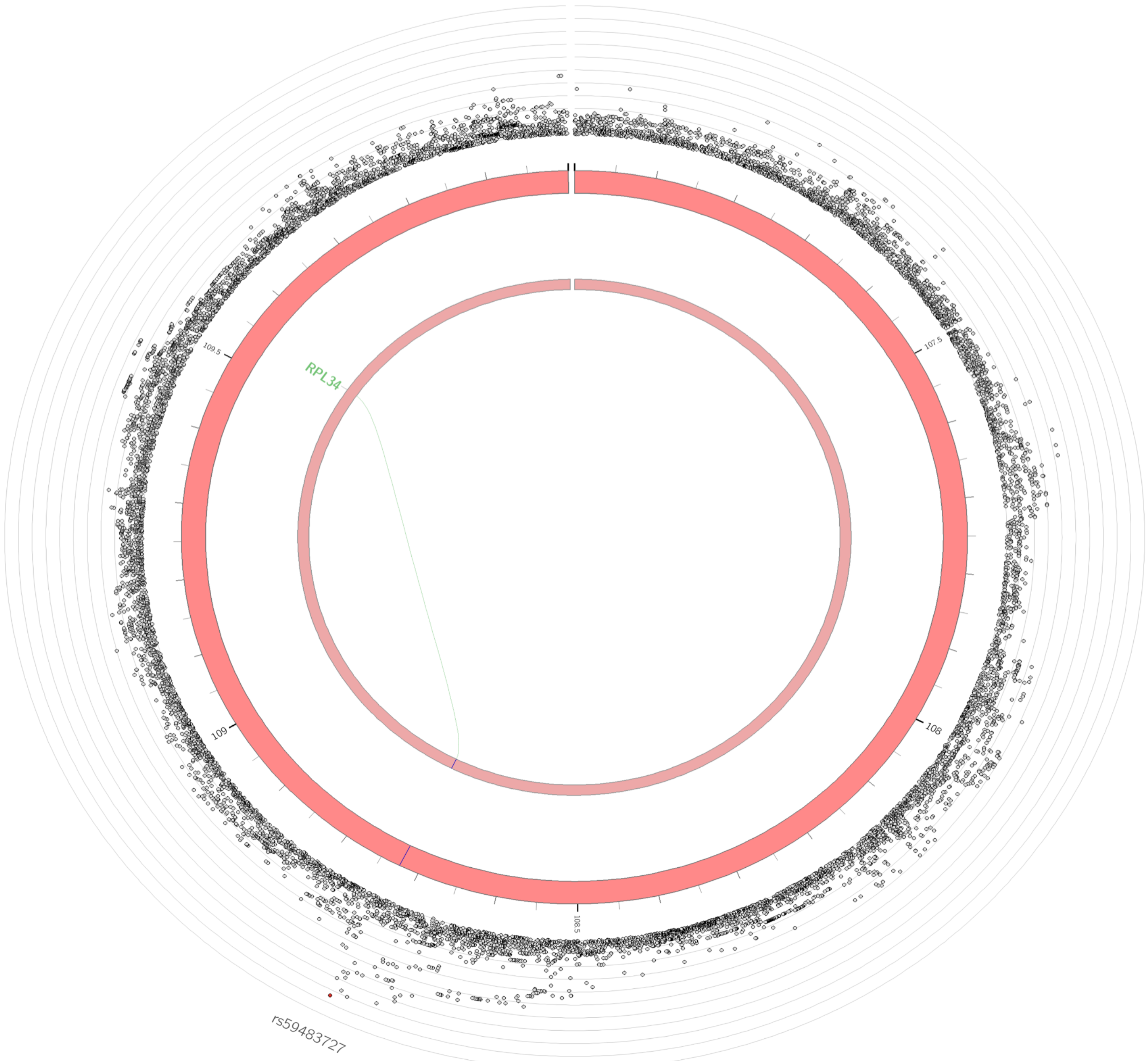

circos_chr5

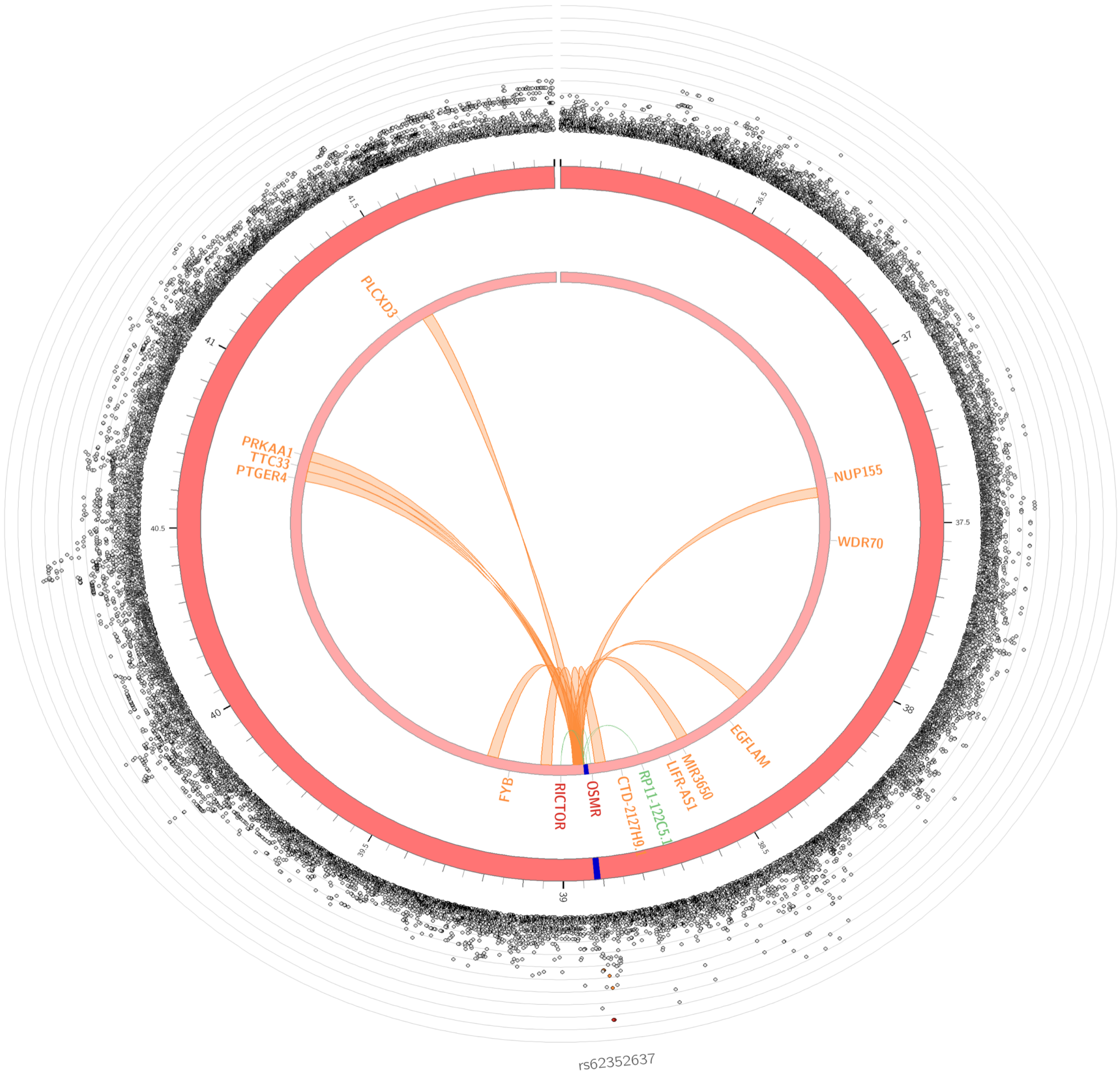
PLCXD3
PRKAA1
TTC33
PTGER4
NUP155
WDR70
EGFLAM
MIR3650
LIFR-AS1
FYB
RICTOR
OSMR
41.5
41
40.5
40
39.5
39
36.5
37
37.5
38
38.5
rs62352637

circos_chr6

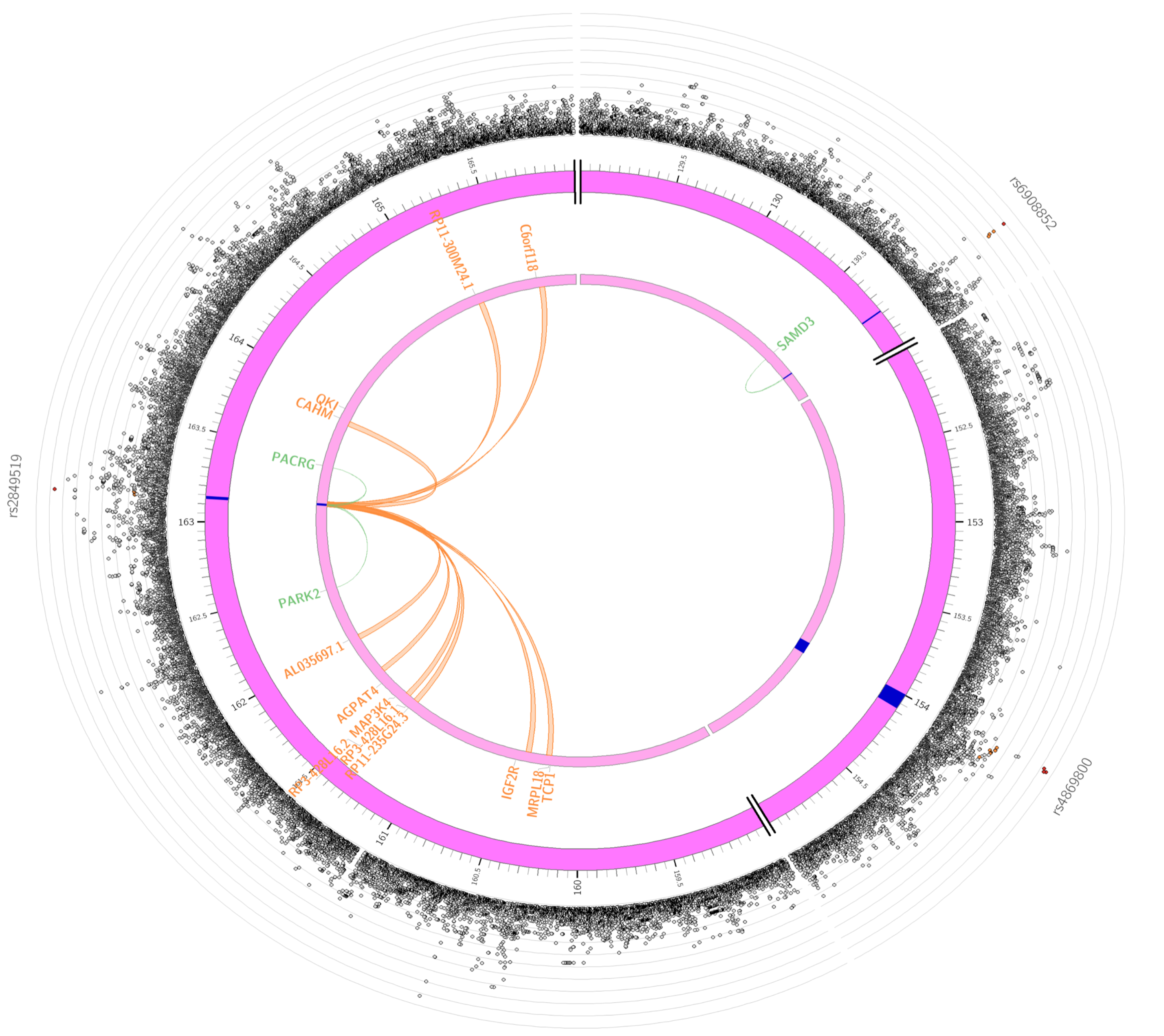

rs6908852
rs4869800
rs2849519
C6orf118
RP11-300M24.1
SAMD3
QKI
CAHM
PACRG
PARK2
AL035697.1
AGPAT4
MAP3K4
IGF2R
MRPL18
TCP1
129.5
130
130.5
152.5
153
153.5
154
154.5
159.5
160
160.5
161
162
162.5
163
163.5
164
164.5
165
165.5

circos_chr7

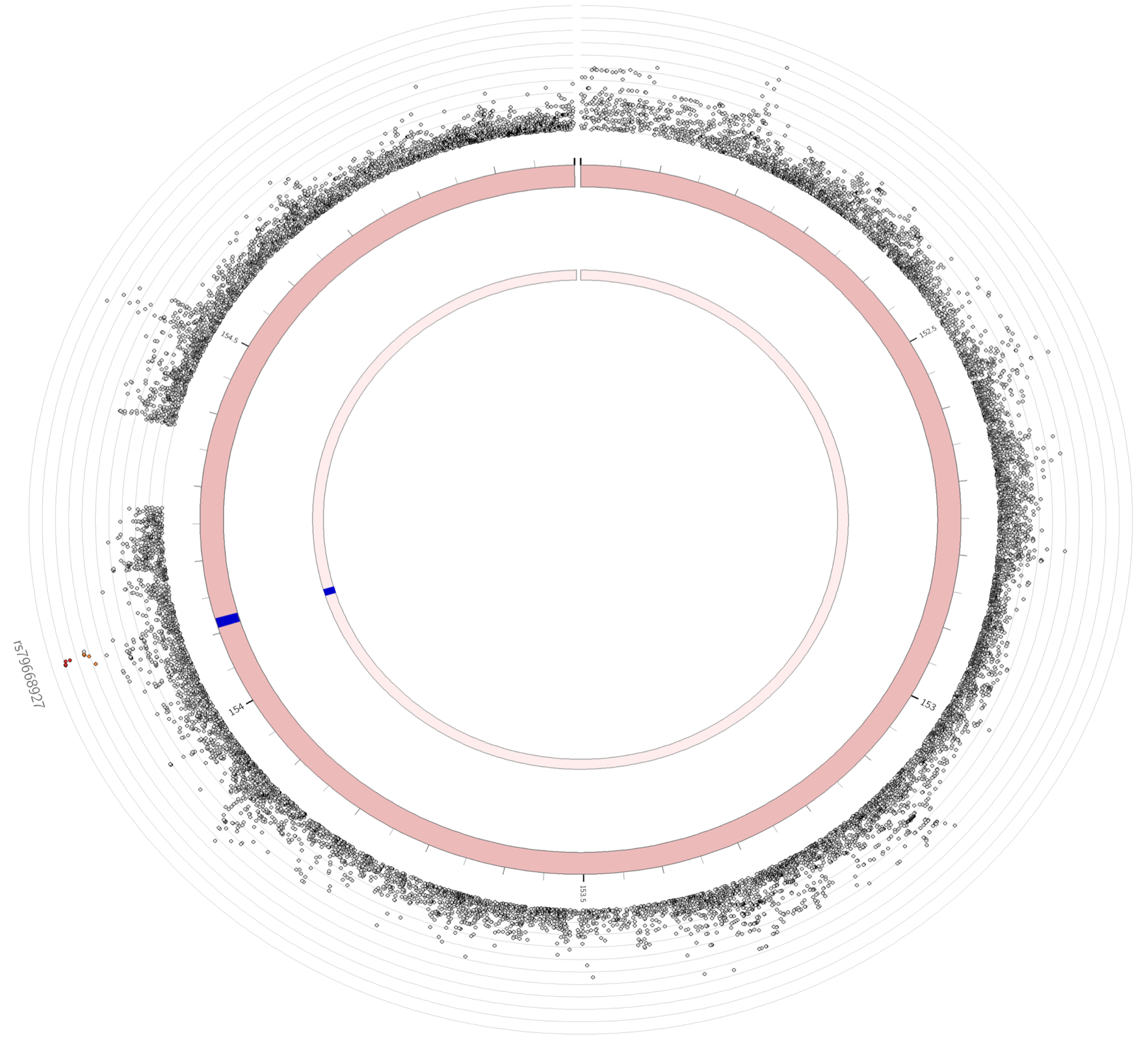

circos_chr9

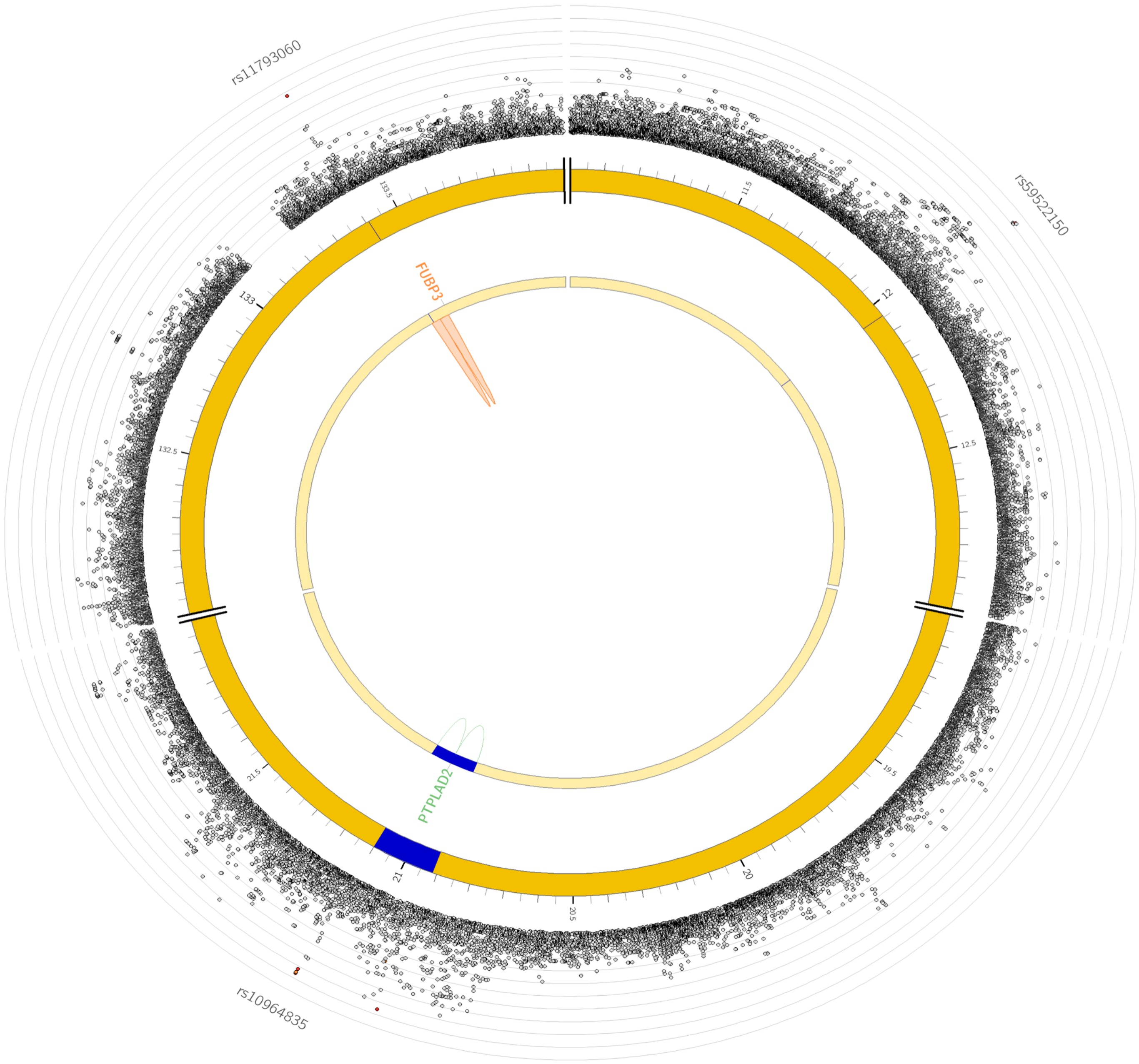
rs11793060
rs59522150
rs10964835
FUBP3
PTPLAD2
133.5
133
132.5
11.5
12
12.5
19.5
20
20.5
21
21.5

circos_chr11

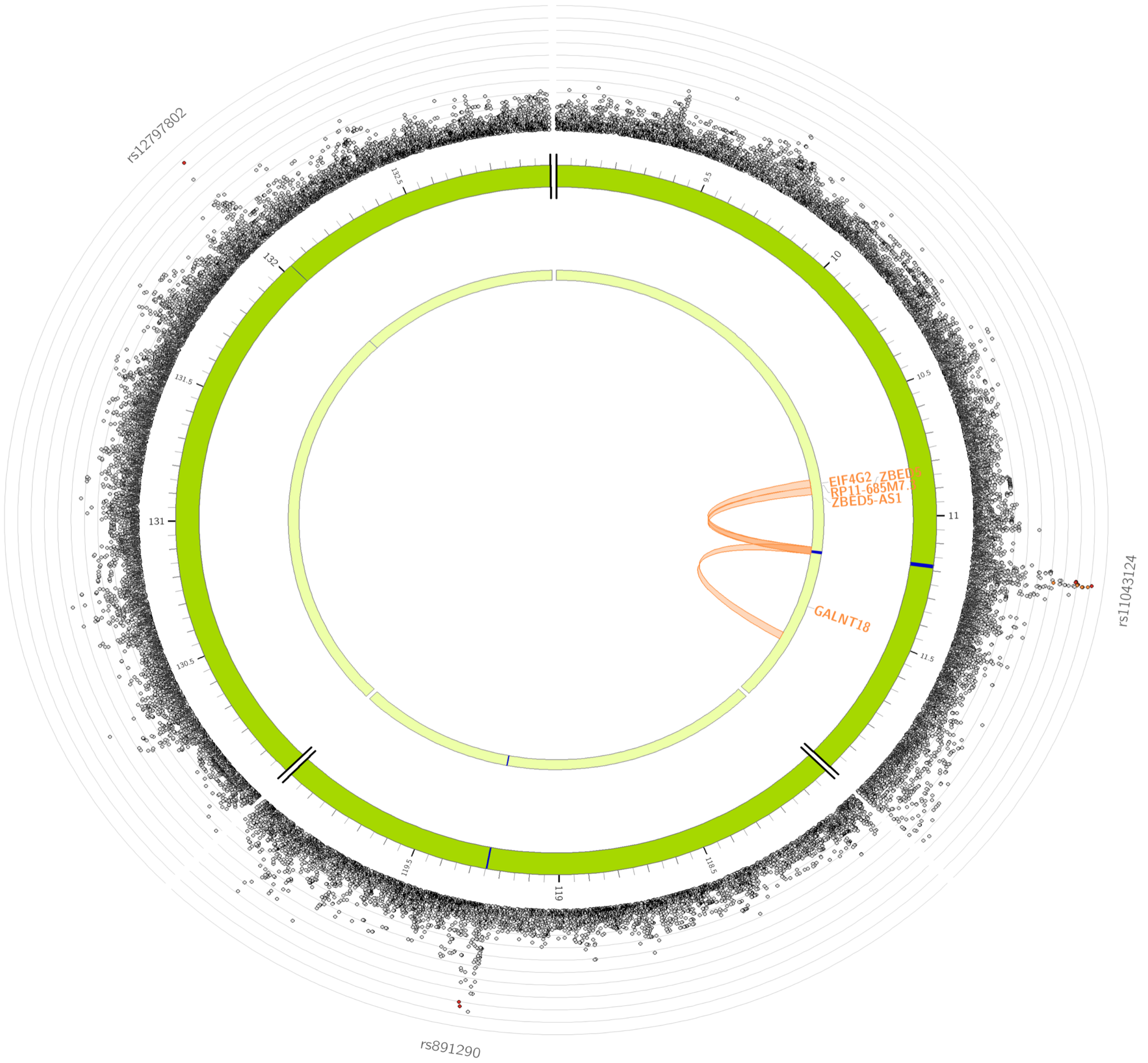

circos_chr12

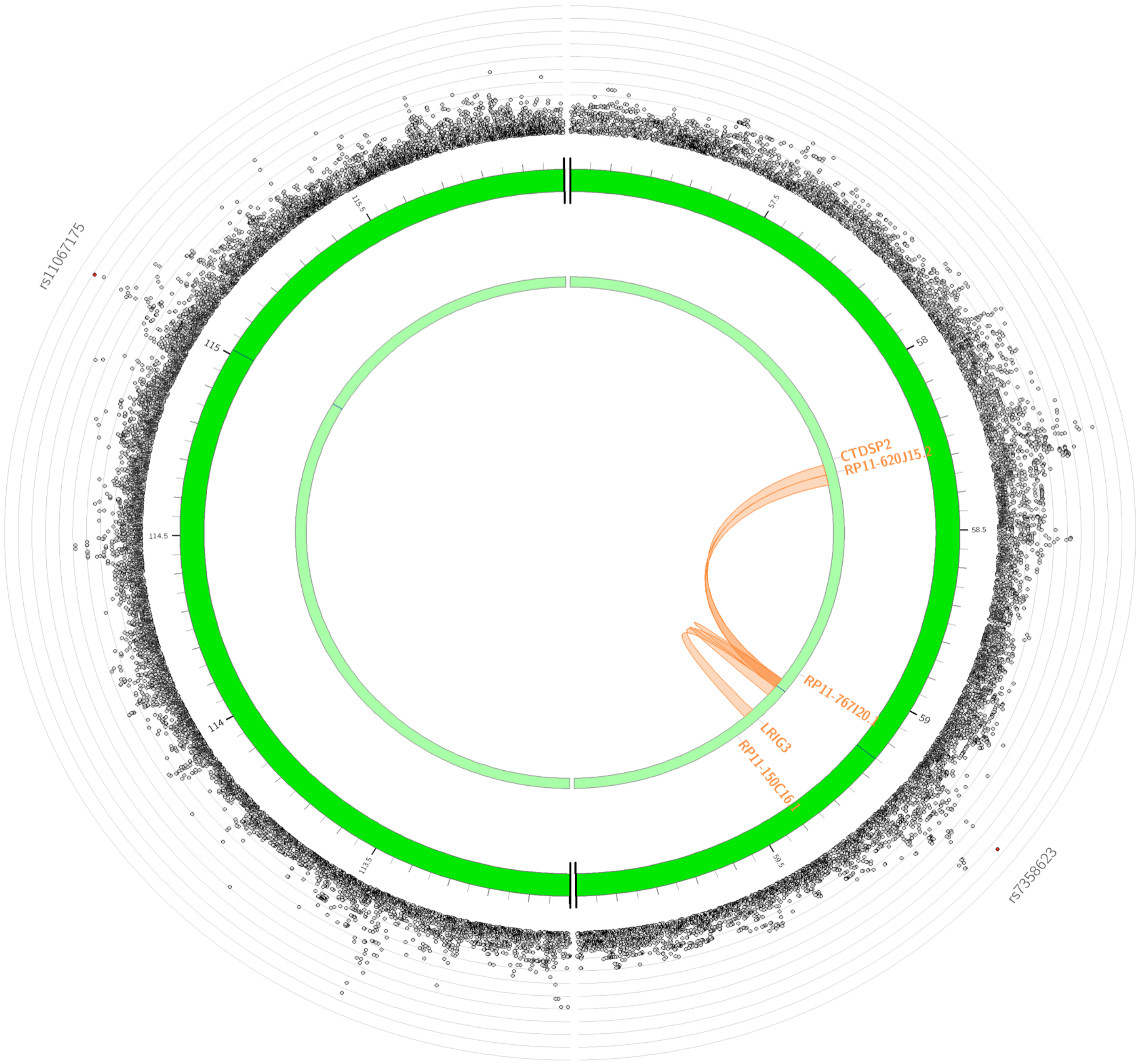

circos_chr14

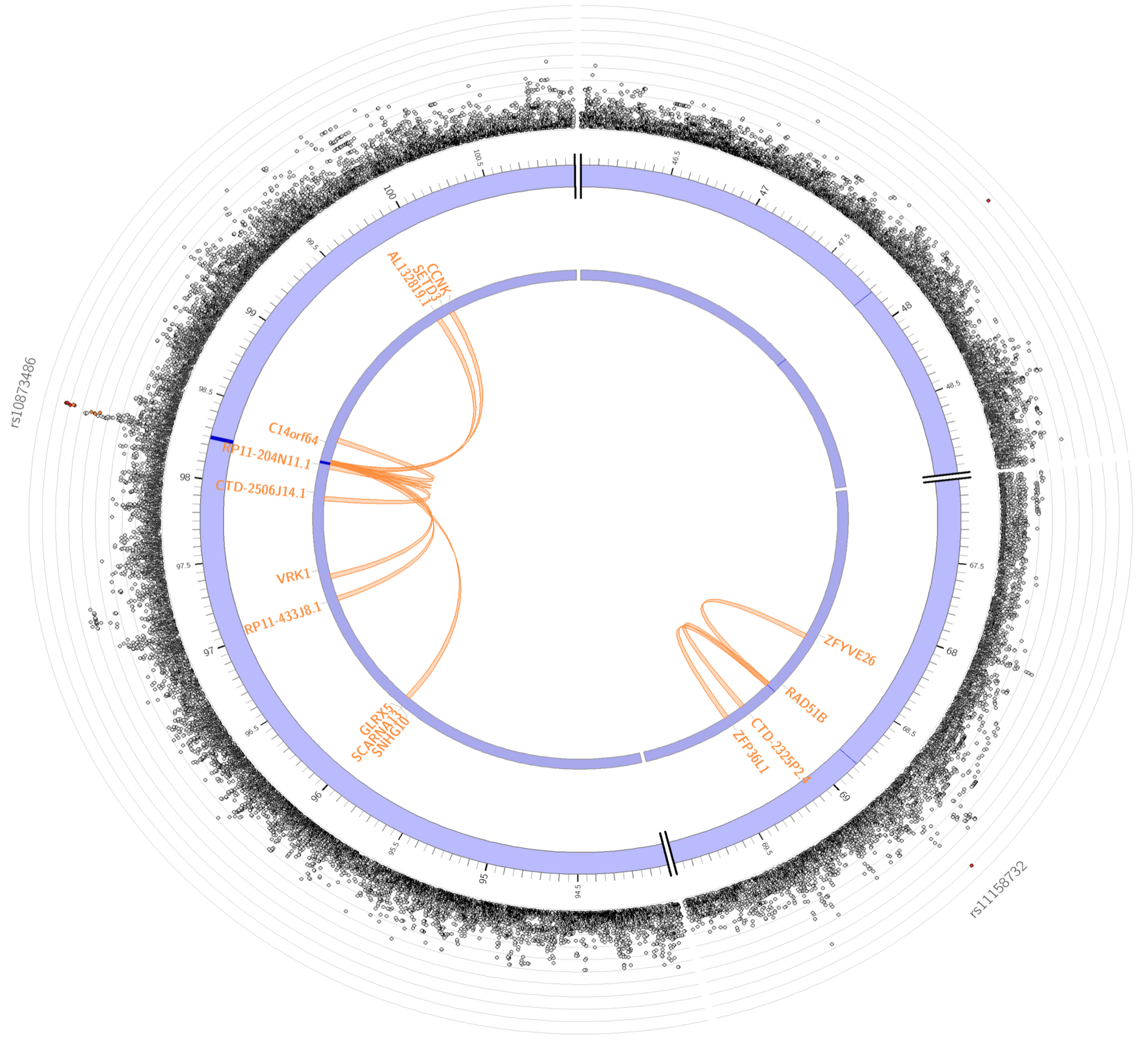

circos_chr17

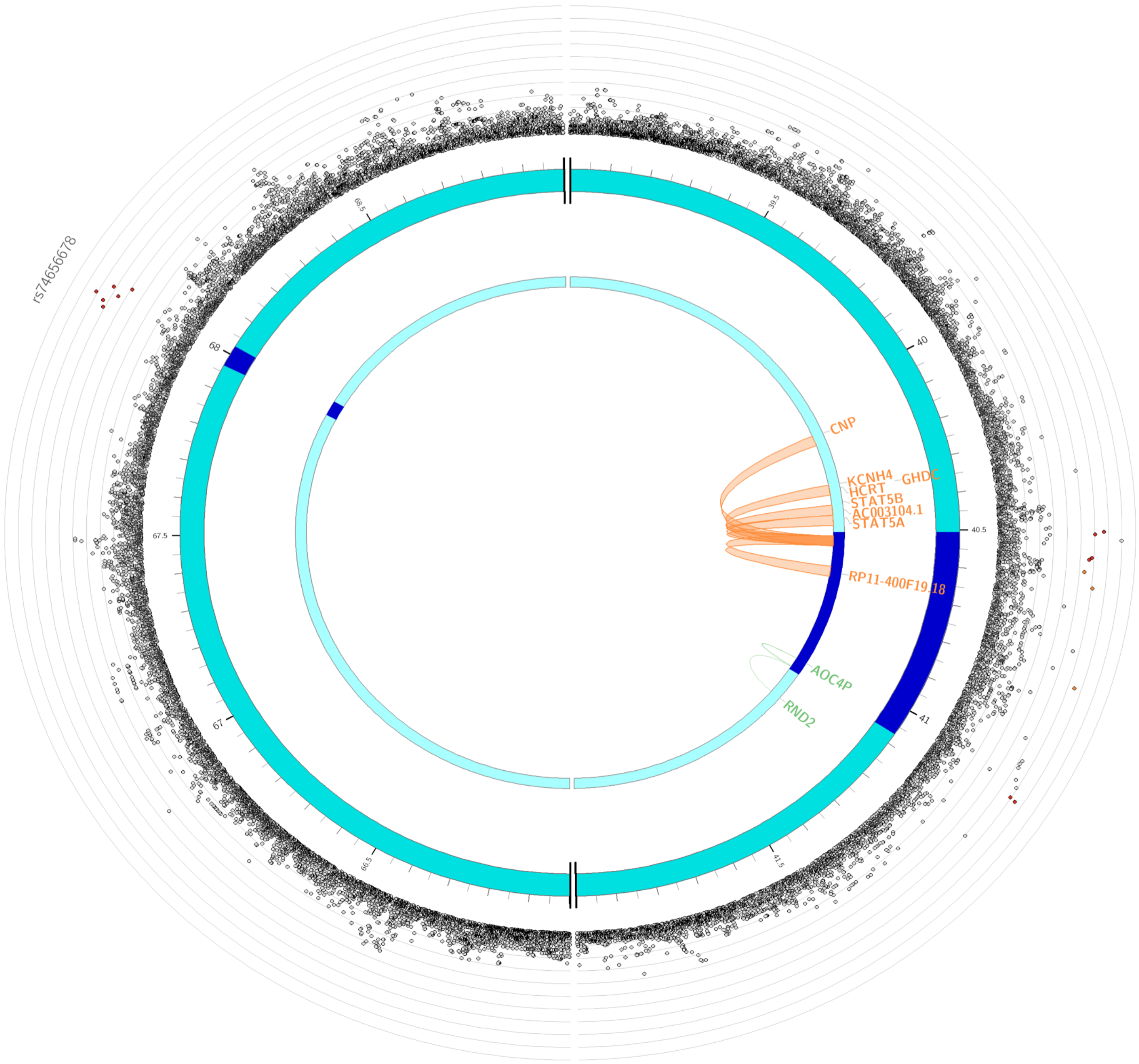

circos_chr19

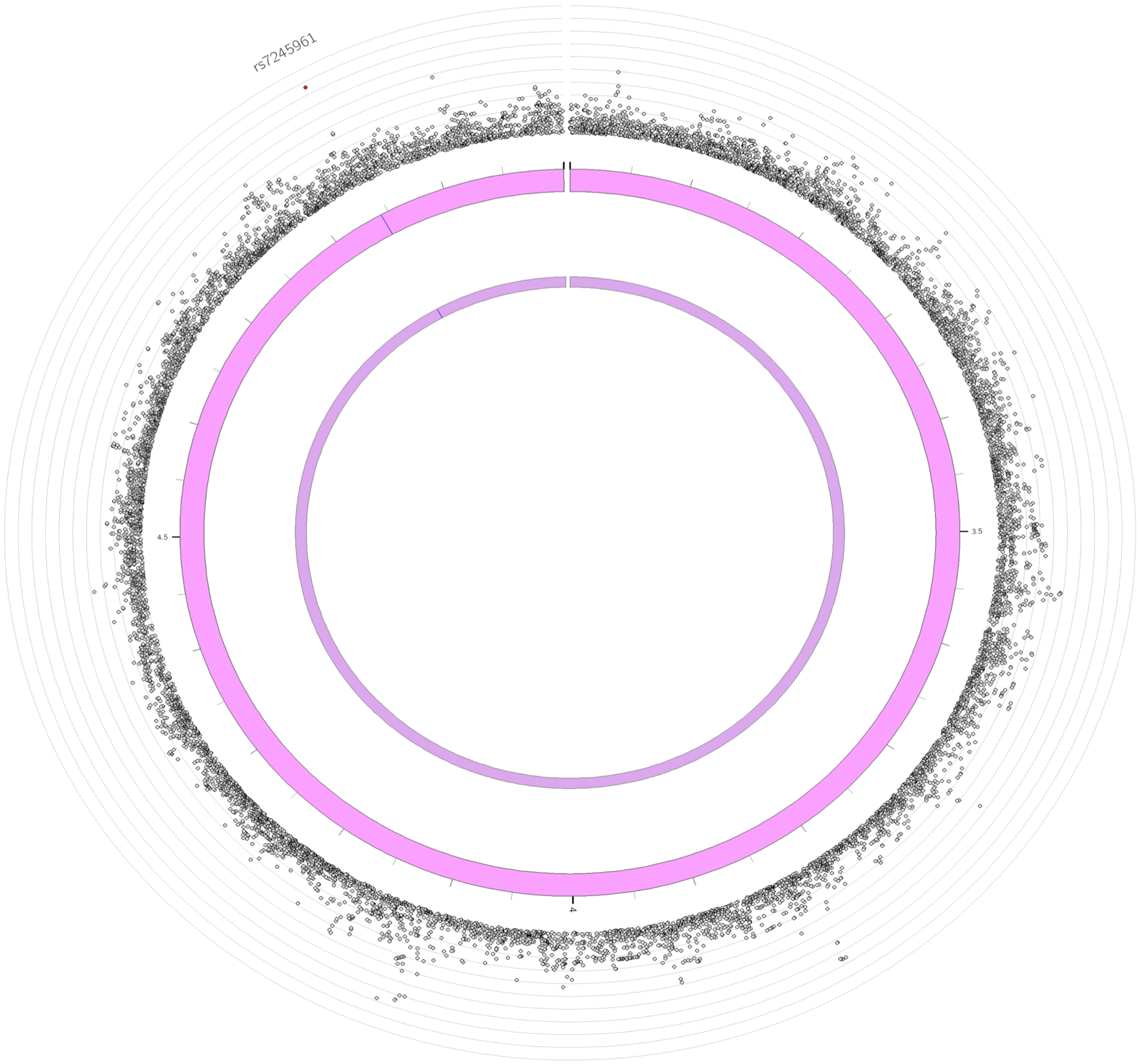

circos_chr20

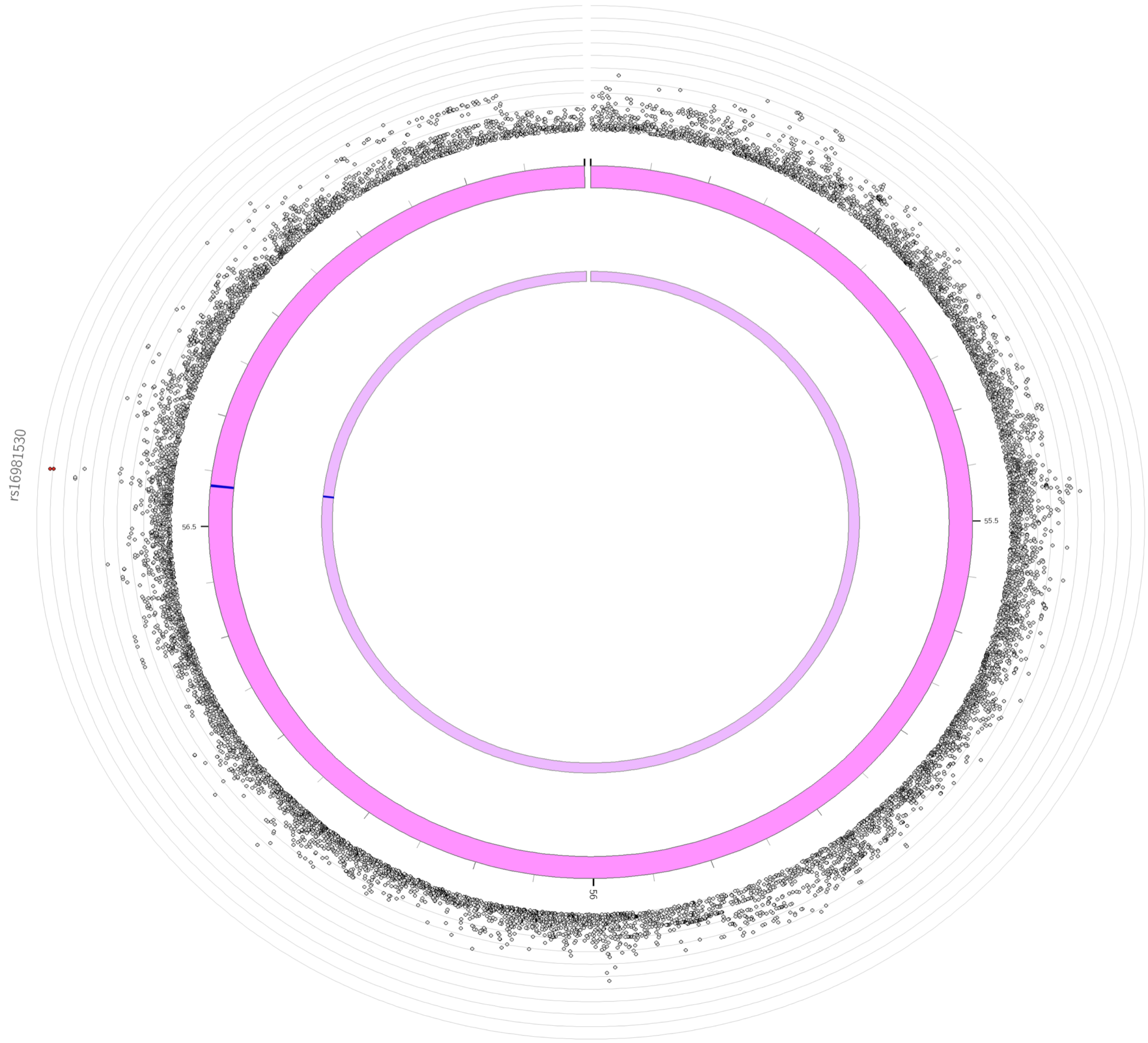

Supplementary Figure 3 c: Circos plots showing genes on chromosomes that were linked to risk (P<1E-05) loci in the GWAS of Combined sample (blue regions) by eQTL mapping (green lines connecting an eQTL SNP to its associated gene) and/or chromatin interactions (orange lines connecting two interacting regions) and showed evidence of interaction across two independent genomic risk loci. Genes implicated by eQTLs are in green, by chromatin interactions are in orange, and by both eQTLs and chromatin interactions are in red. The outer layer shows a Manhattan plot containing the $-\log_{10}$-transformed two-tailed *P* value of each SNP from the GWAS meta-analysis (of linear and logistic regression statistics), with genome-wide significant SNPs colored according to LD patterns with the lead SNP. LD values has been generated from African samples from 1000 GP.

circos_chr1

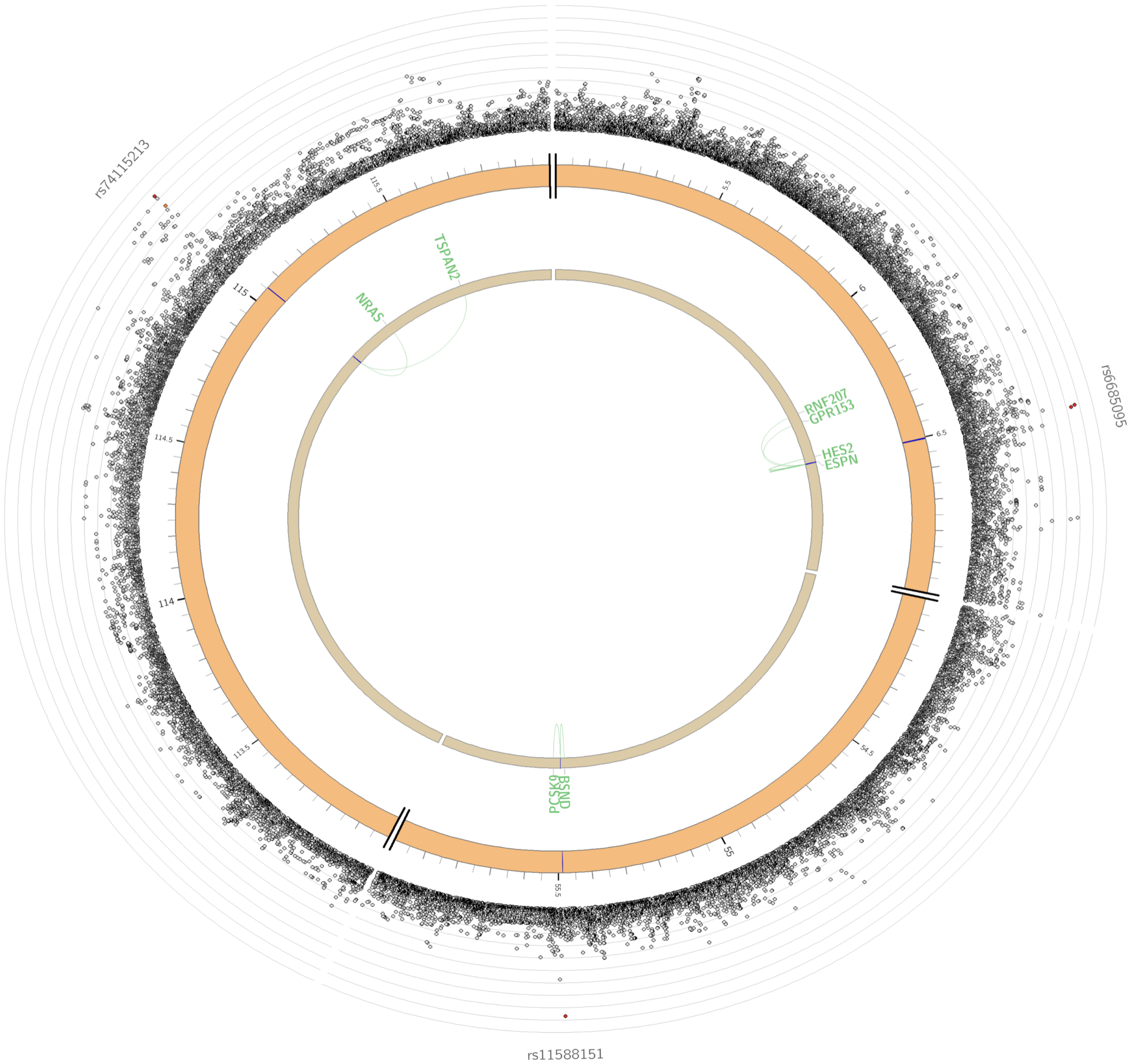

rs74115213
rs6685095
rs11588151
TSPAN2
NRAS
RNF207
GPR153
HES2
ESPN
PCSK9
BSND
115.5
115
114.5
114
113.5
5.5
6
6.5
54.5
55
55.5

circos_chr2

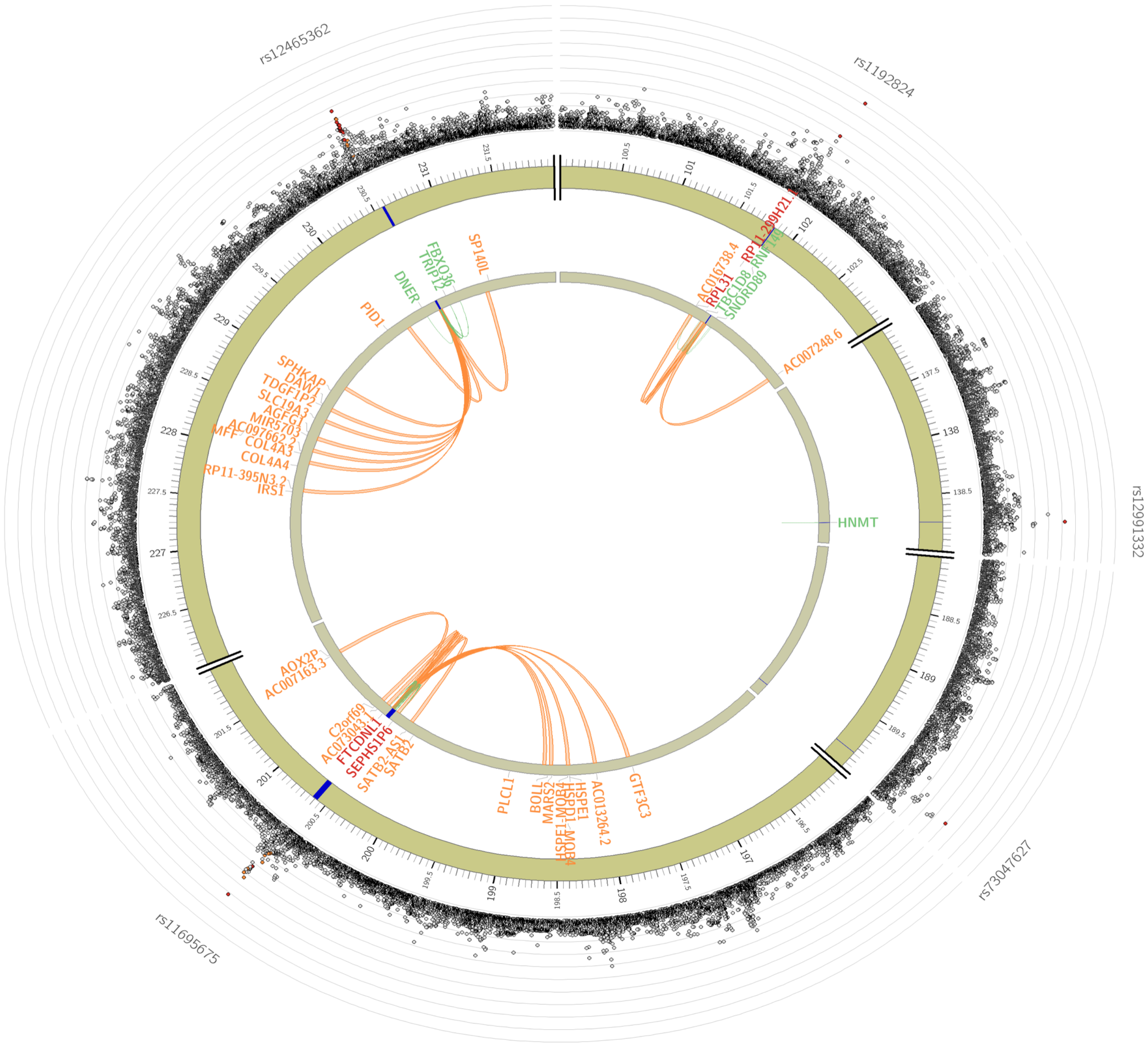
rs12465362
rs1192824
rs12991332
rs73047627
rs11695675
SP140L
FBXO36
TRIP12
DNER
PID1
SPHKAP
DAW1
TDGF1P2
SLC19A3
AGFG1
MIR5703
AC097662.2
MFF
COL4A3
COL4A4
RP11-395N3.2
IRS1
AC016738.4
RPL31
TBC1D8
SNORD89
AC007248.6
HNMT
AOX2P
AC007163.3
C2orf69
FTCDNL1
SEPHS1P6
SATB2-AS1
SATB2
PLCL1
BOLL
MARS2
HSPE1
AC013264.2
GTF3C3

circos_chr3

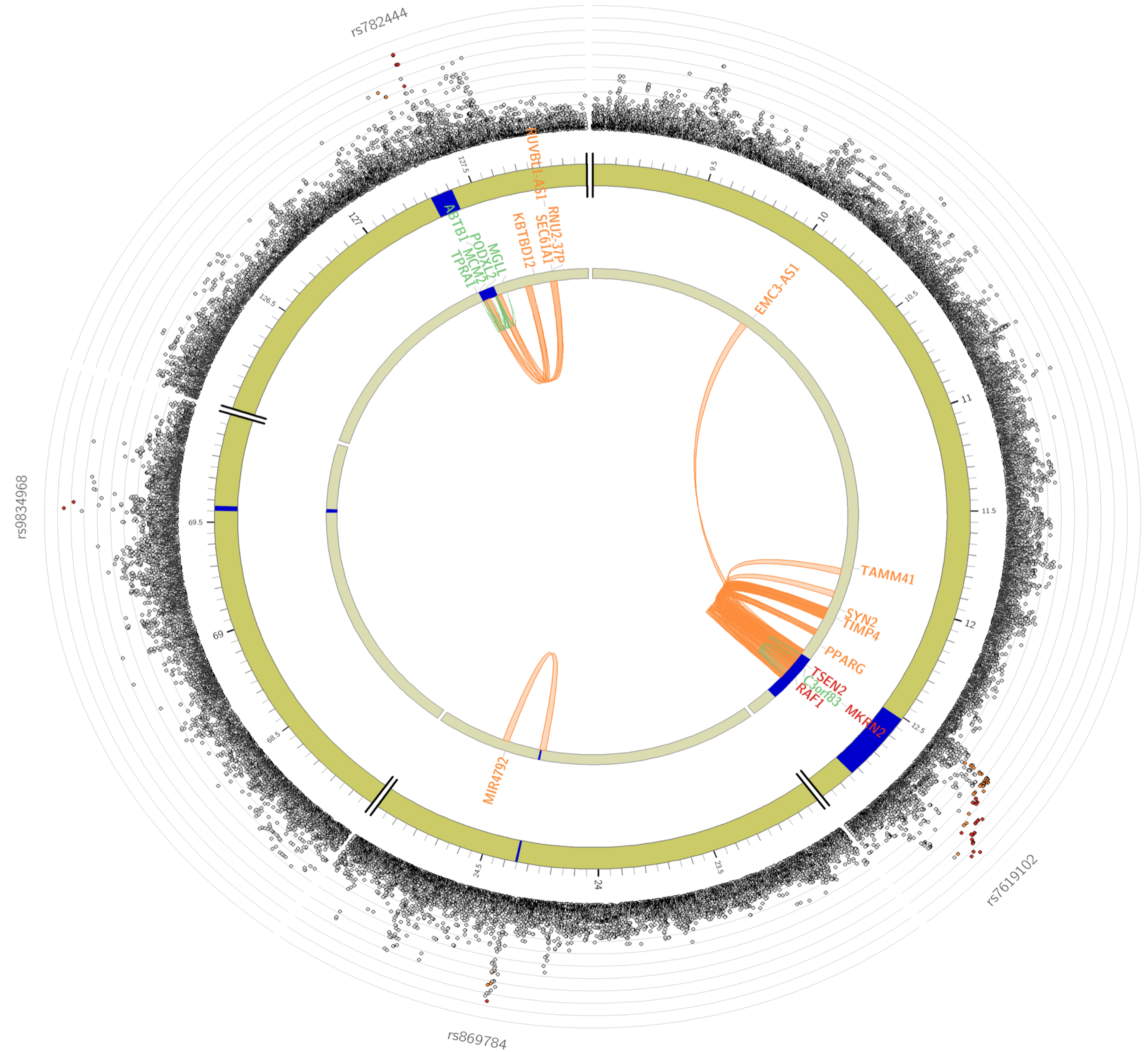

rs782444
rs9834968
rs869784
rs7619102
EMC3-AS1
TAMM41
SYN2
TIMP4
PPARG
TSEN2
C3orf83
RAF1
MKRN2
MIR4792
KBTBD12
SEC61A1
RNU2-37P
RUVBL1-AS1
MGLL
PODXL2
MCM2
TPRA1
ABTB1

circos_chr4

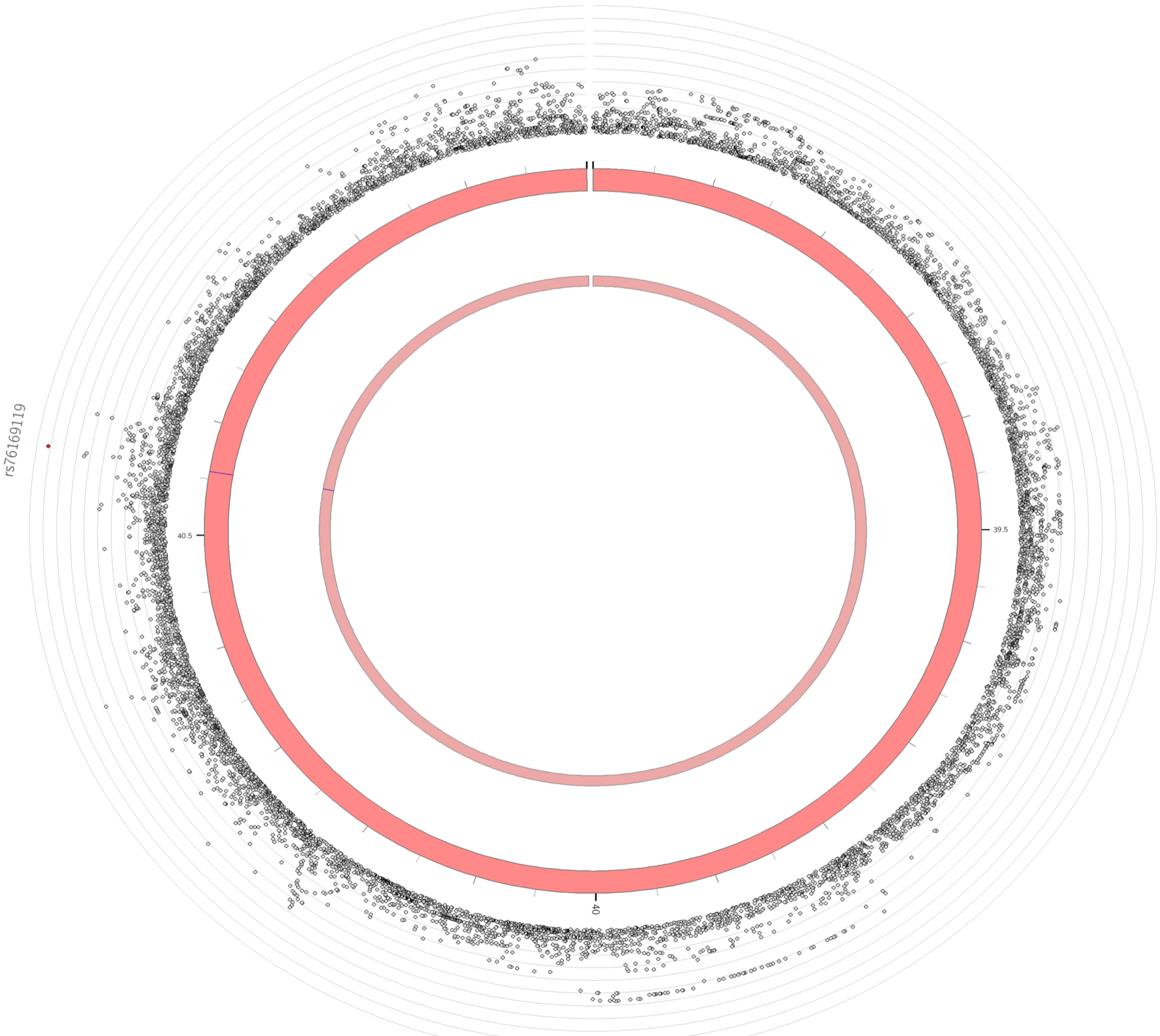

circos_chr5

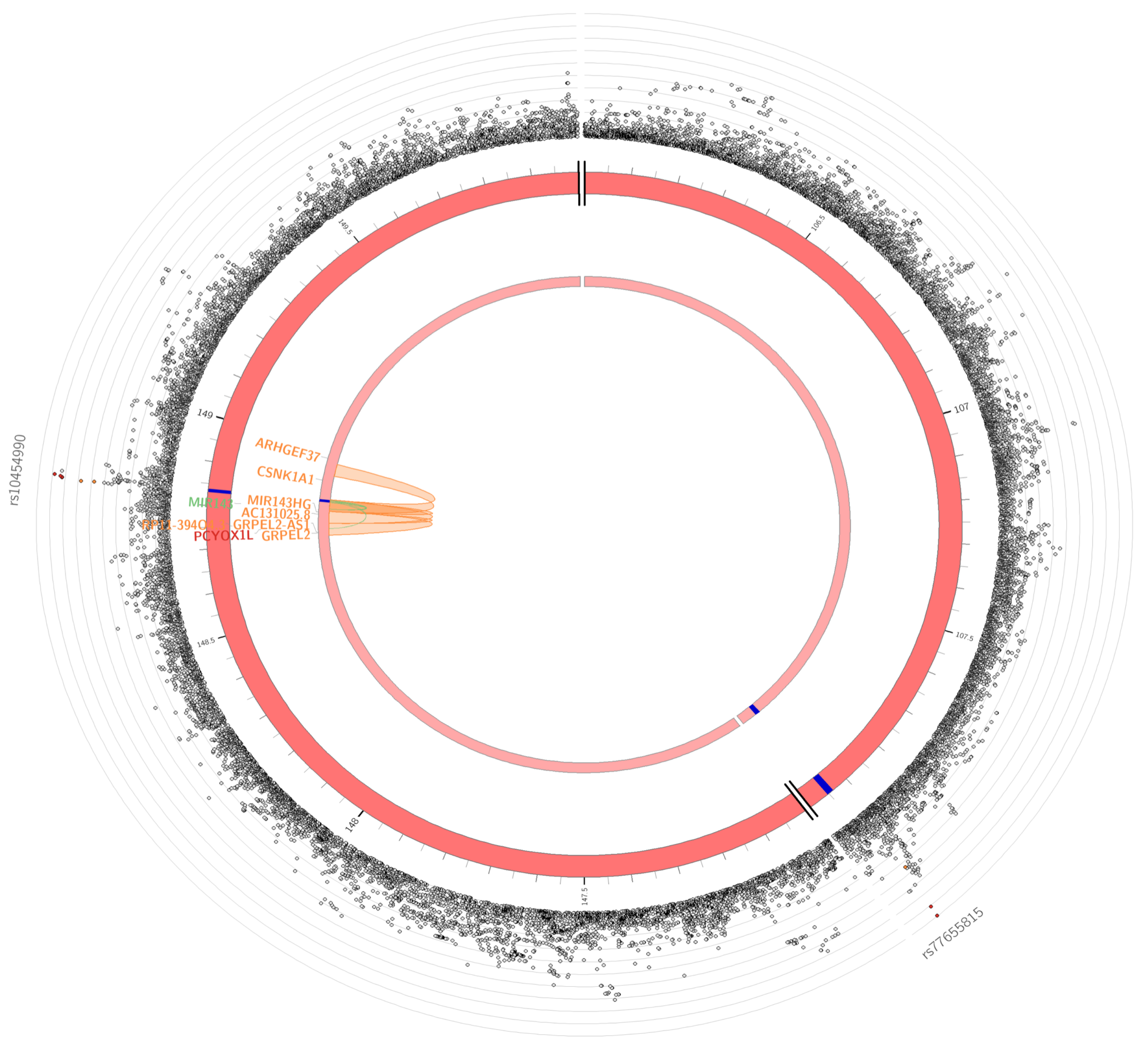
rs10454990
rs77655815
ARHGEF37
CSNK1A1
MIR143
MIR143HG
AC131025.8
GRPEL2-AS1
GRPEL2
PCYOX1L
106.5
107
107.5
147.5
148
148.5
149
149.5

circos_chr6

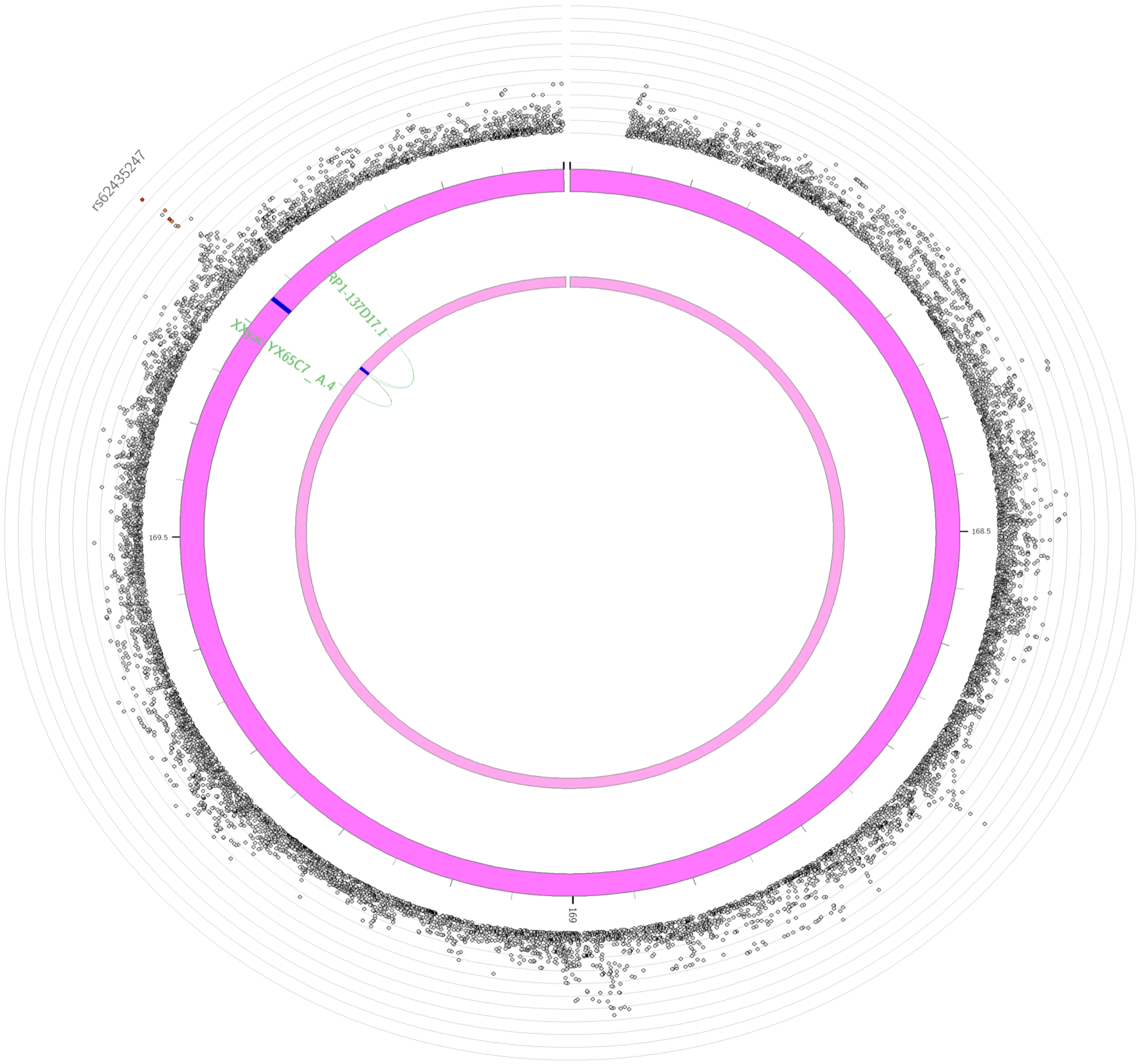

circos_chr7

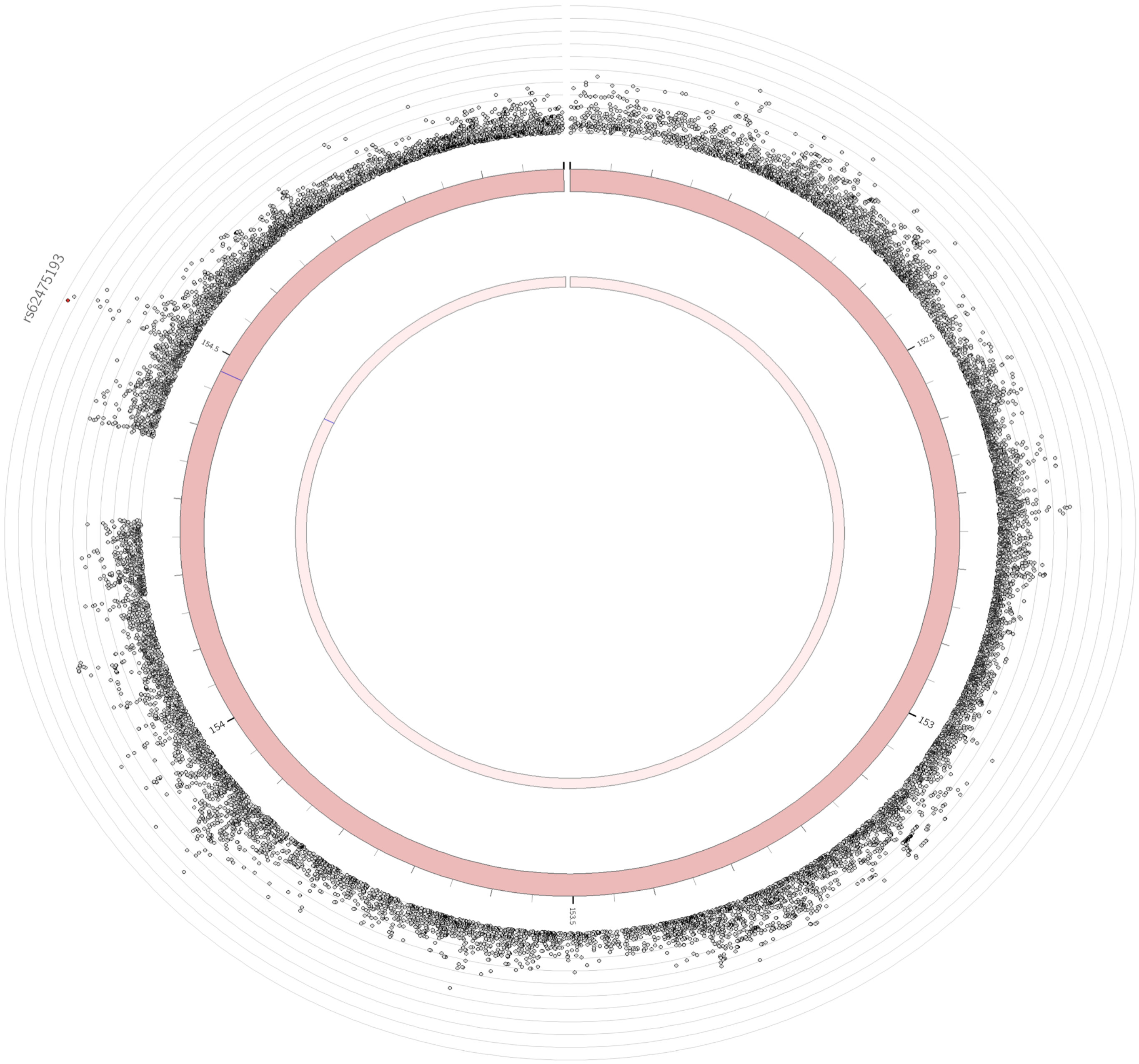

circos_chr8

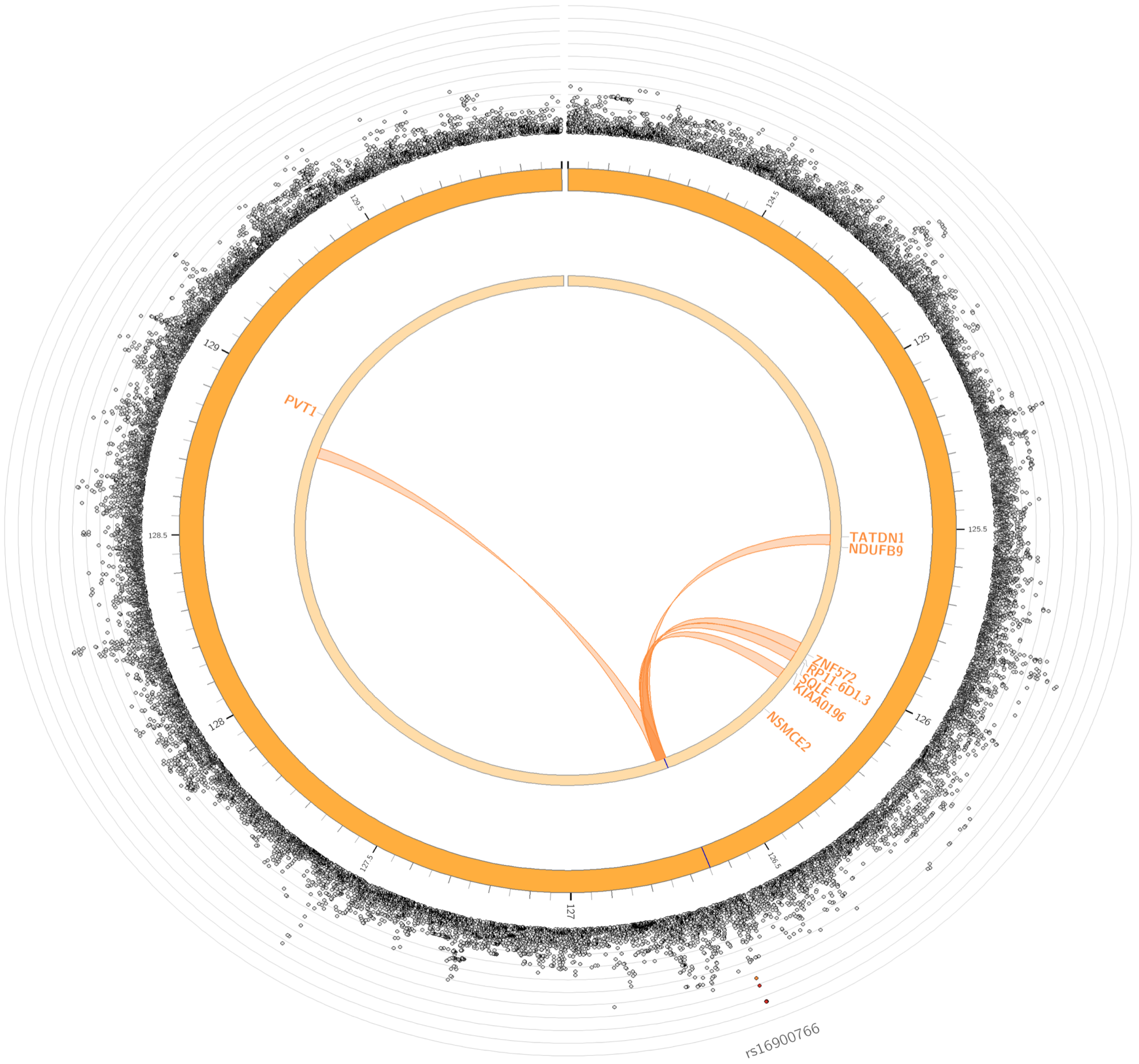

124.5
125
125.5
126
126.5
127
127.5
128
128.5
129
129.5
PVT1
TATDN1
NDUFB9
ZNF572
RP11-6D1.3
SQLE
KIAA0196
NSMCE2
rs16900766

circos_chr10

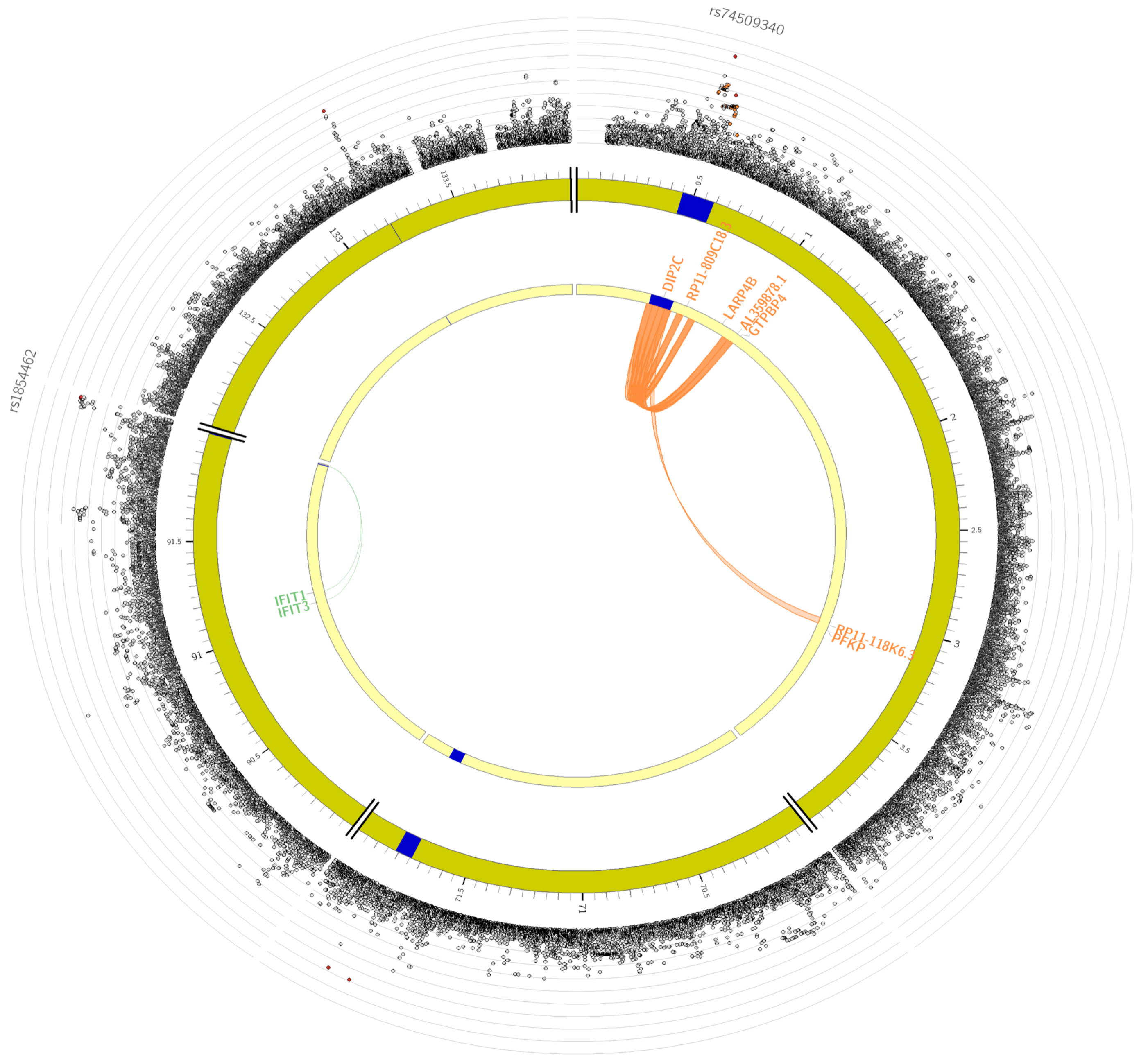

circos_chr11

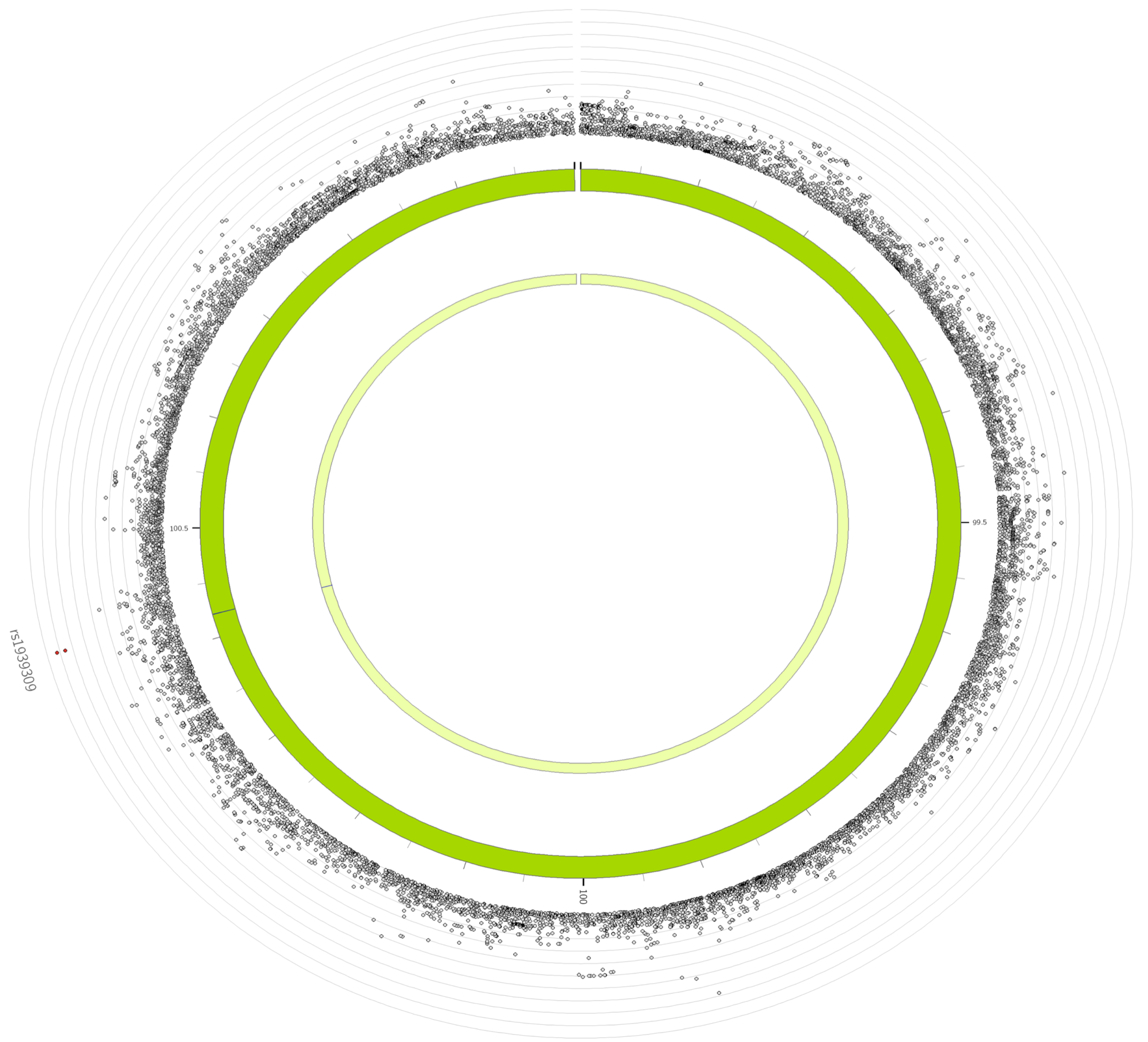

circos_chr13

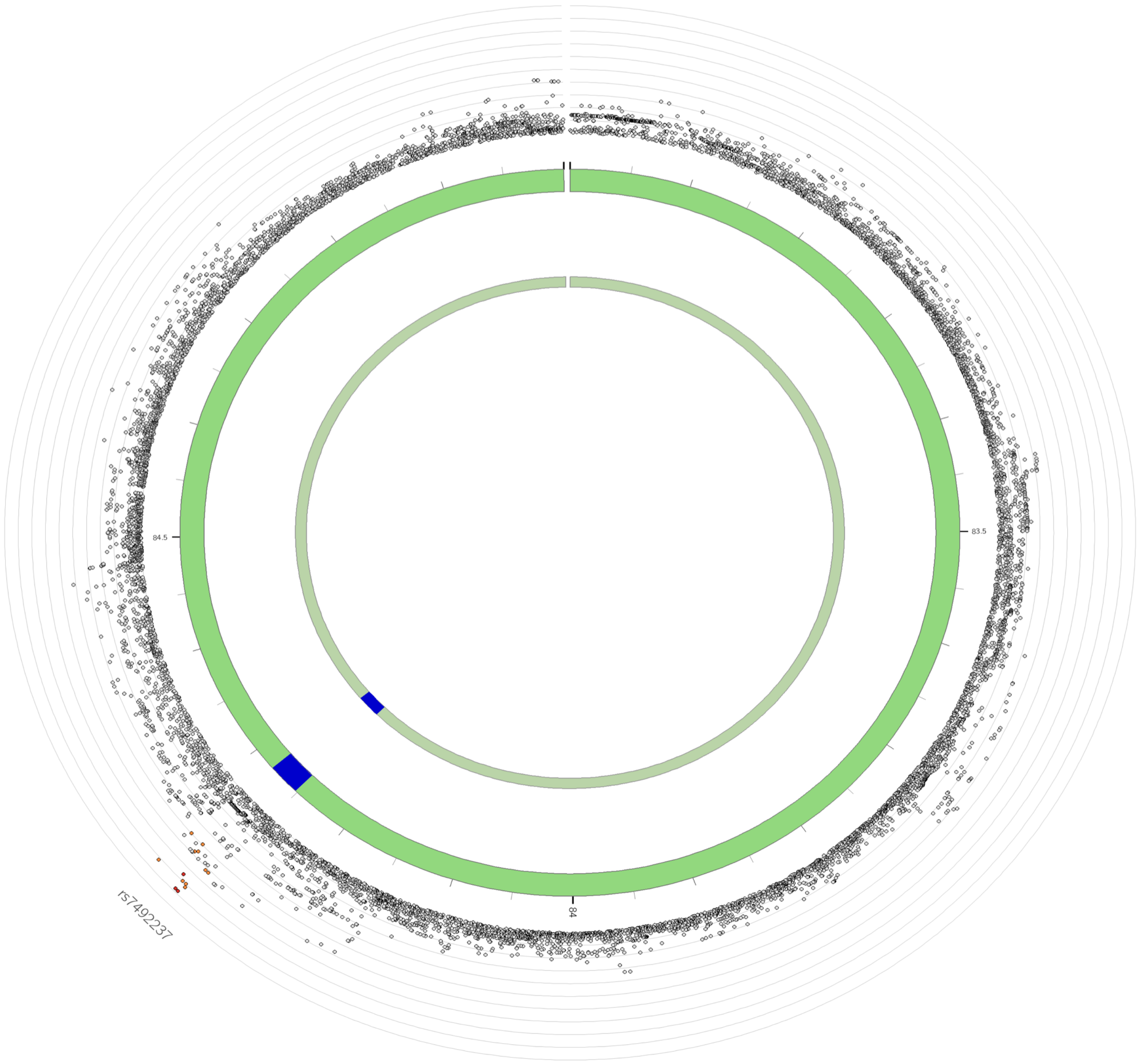

circos_chr14

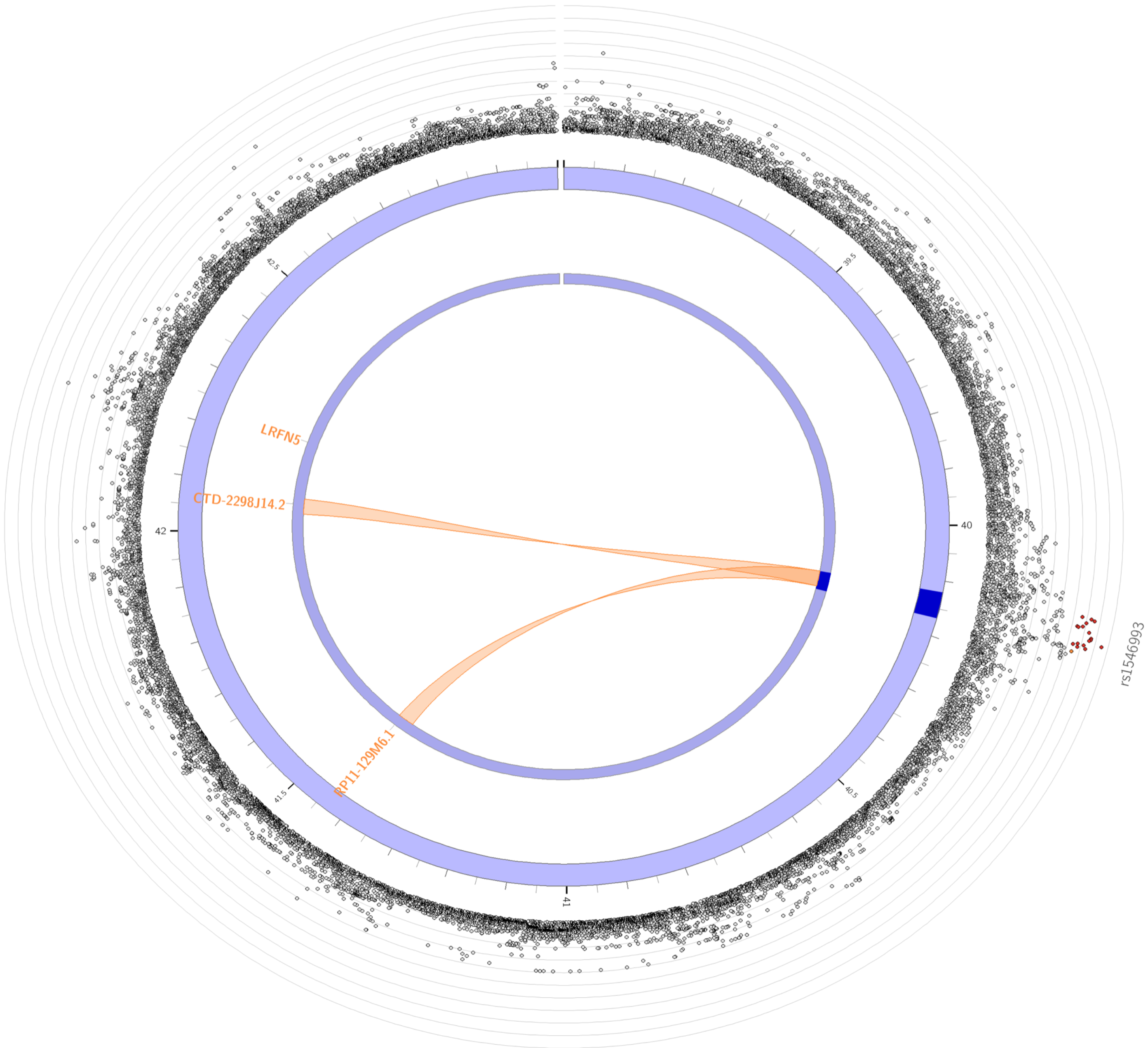

circos_chr16

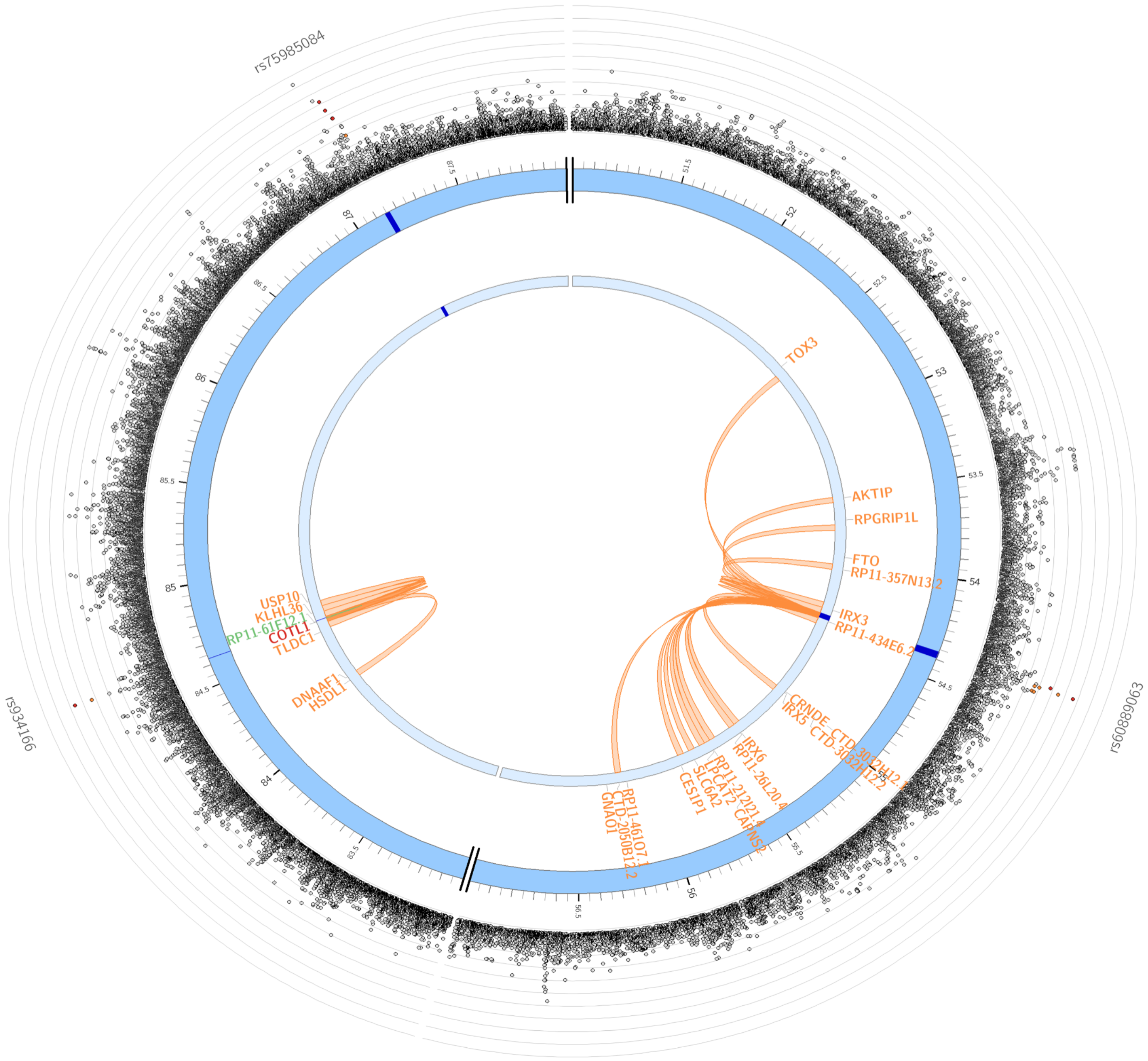

circos_chr17

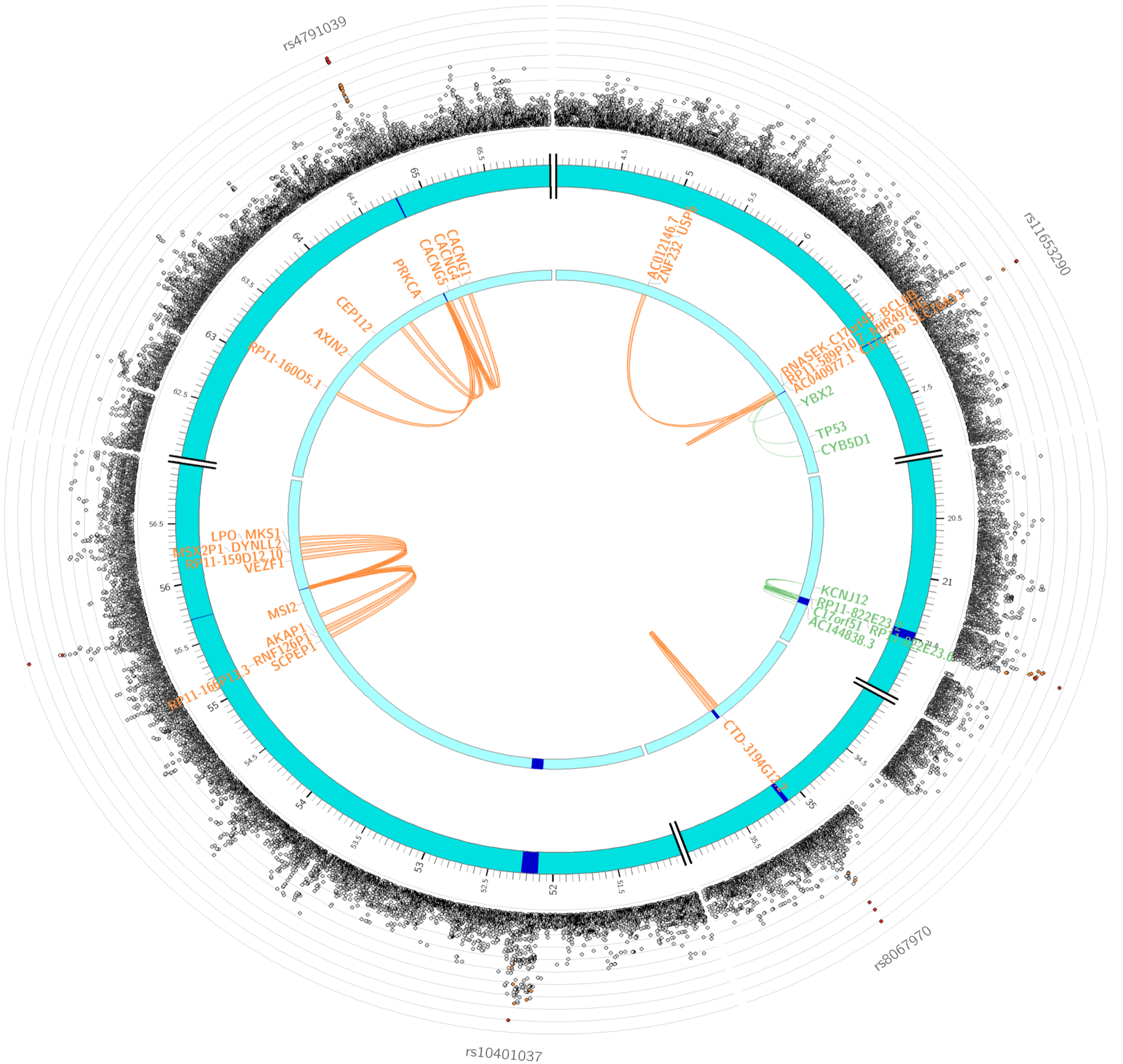

circos_chr18

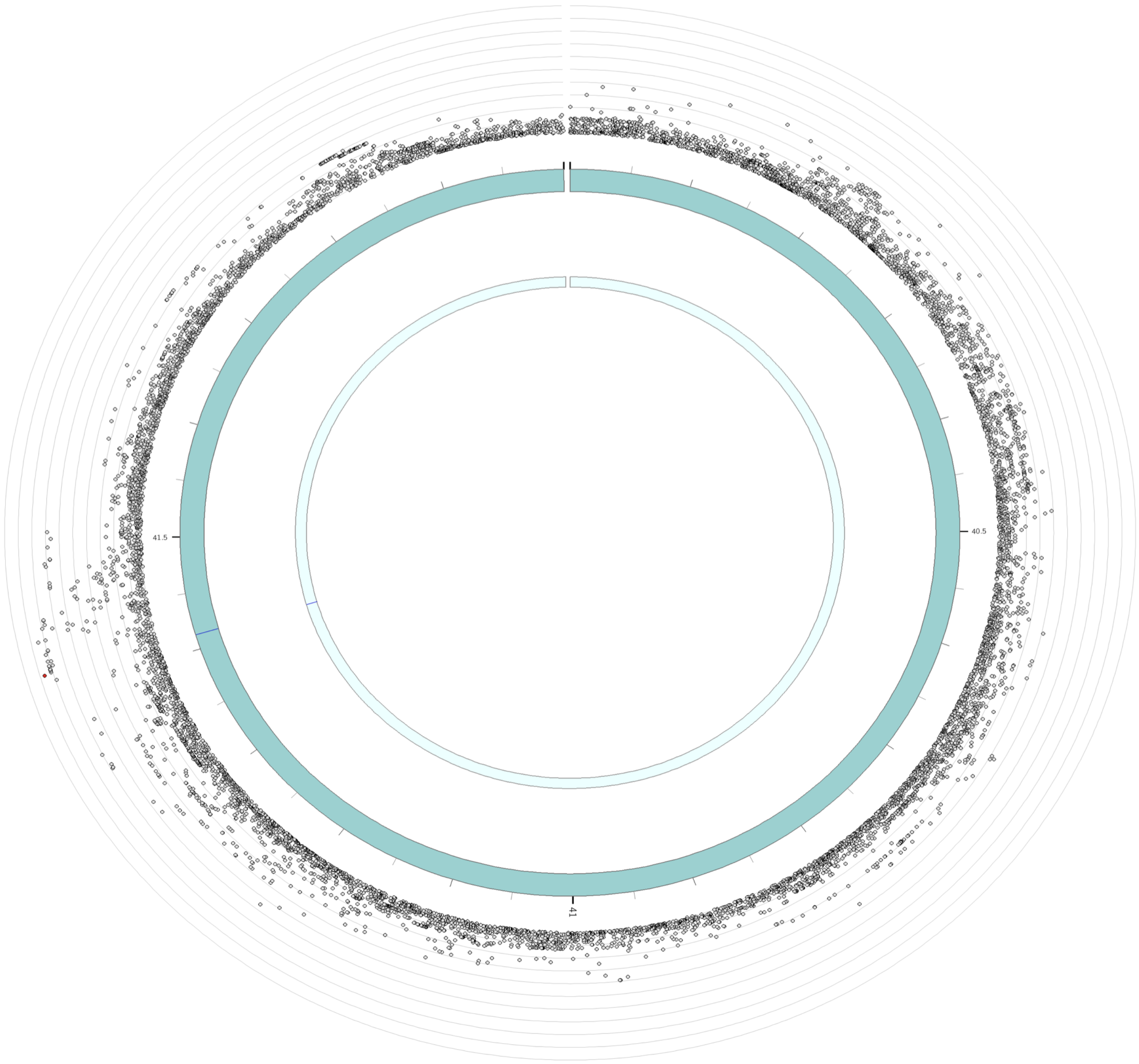

circos_chr19

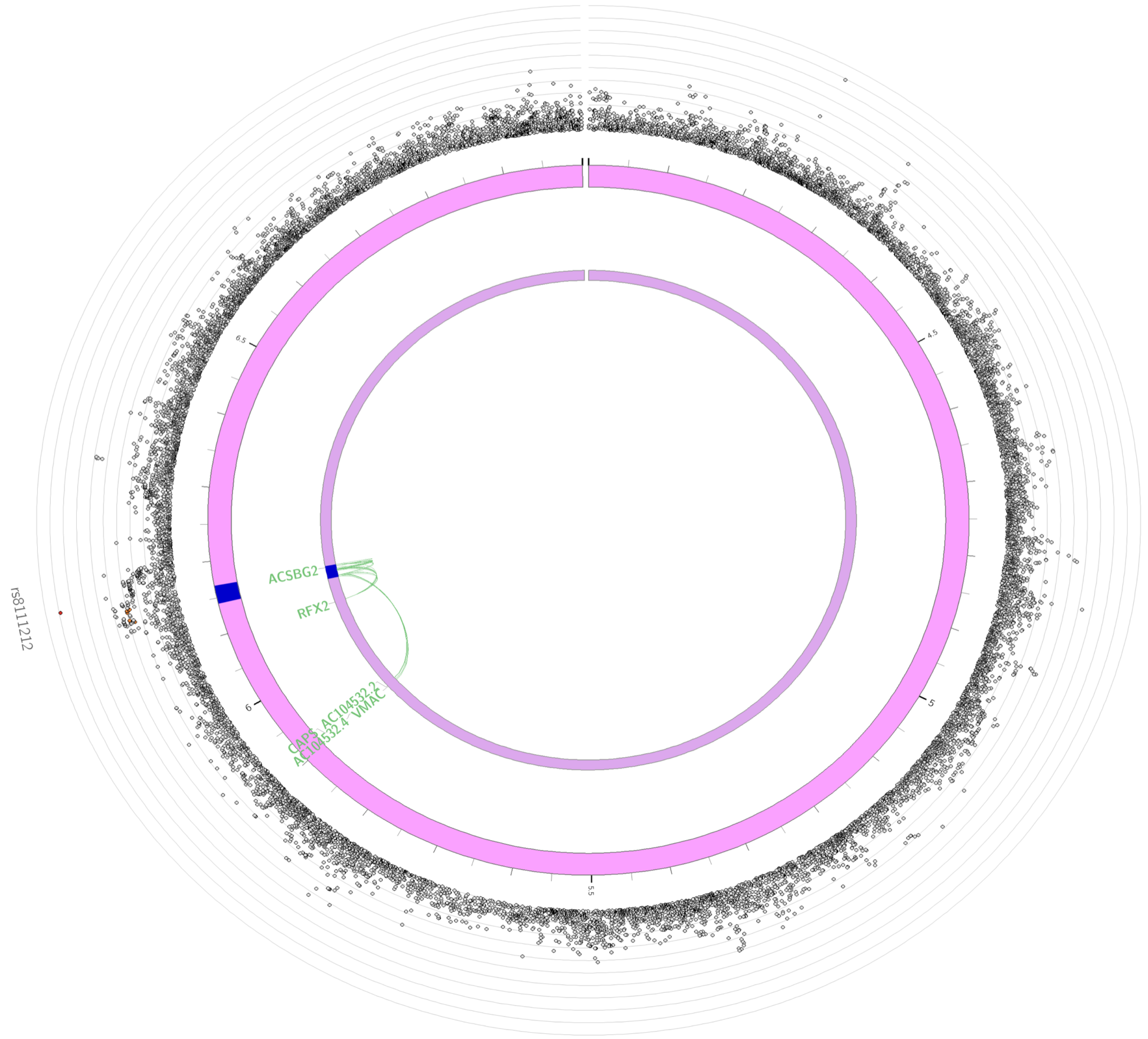

circos_chr20

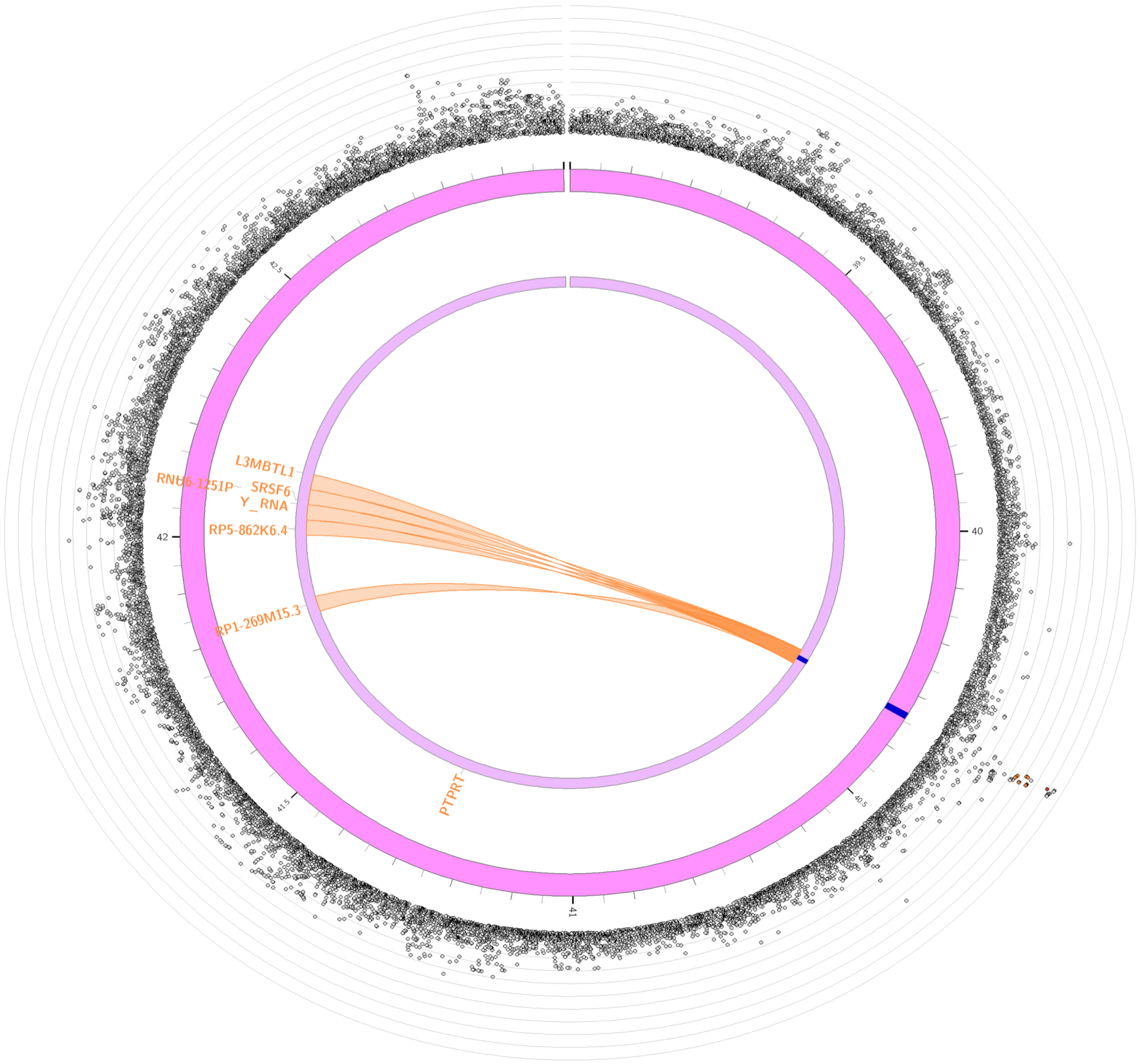

circos_chr21

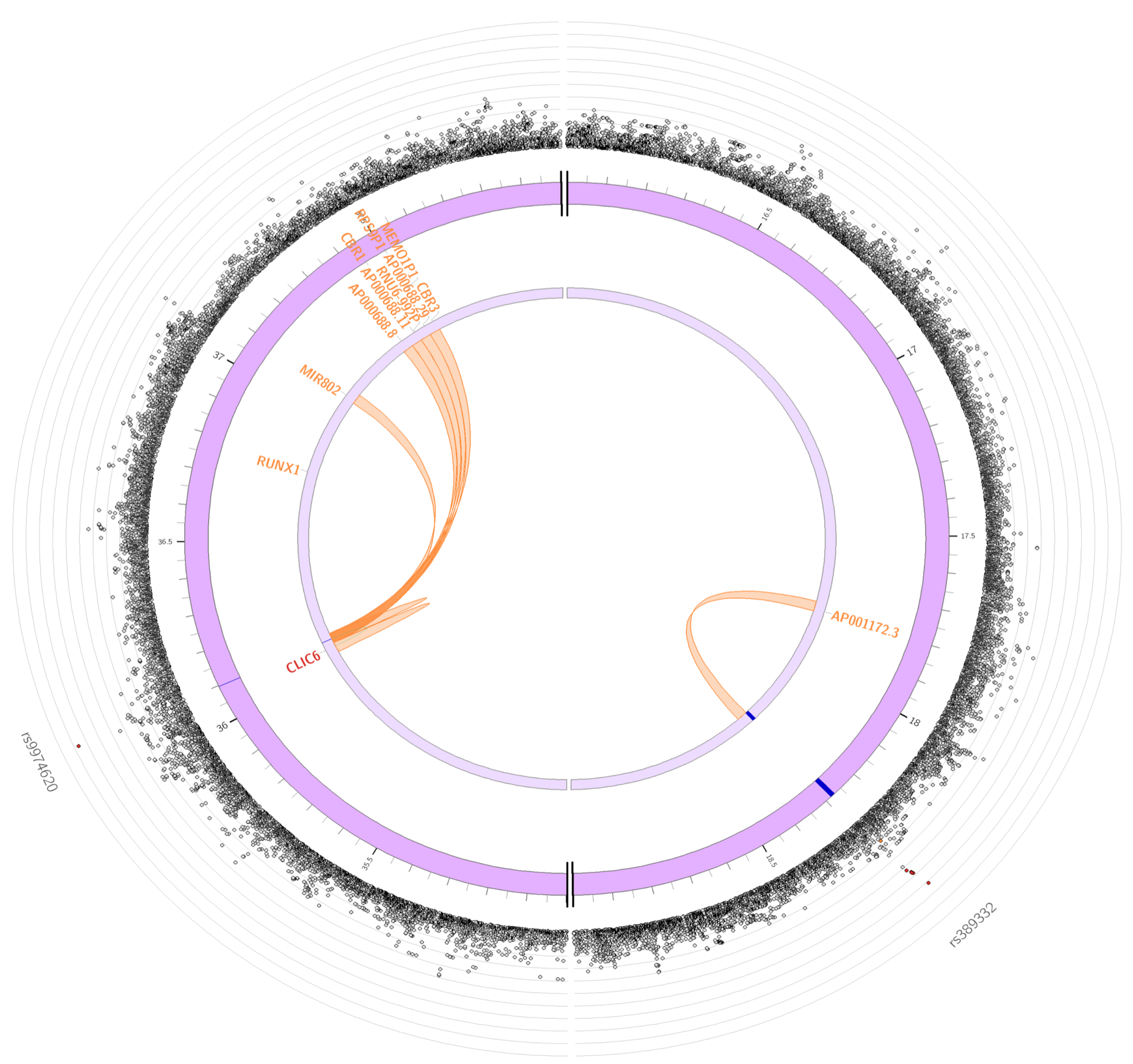

Supplement: Supplementary file 4 [file Image_3.pdf]
